# Identification, replication and characterization of epigenetic remodelling in the aging genome: a cross population analysis

**DOI:** 10.1038/s41598-017-08346-7

**Published:** 2017-08-15

**Authors:** Shuxia Li, Lene Christiansen, Kaare Christensen, Torben A. Kruse, Paul Redmond, Riccardo E. Marioni, Ian J. Deary, Qihua Tan

**Affiliations:** 1 Department of Clinical Research, Unit of Human Genetics, University of Southern Denmark, Odense, Denmark; 2 Department of Public Health, Epidemiology, Biostatistics and Biodemography, University of Southern Denmark, Odense, Denmark; 3 Department of Psychology, University of Edinburgh, Edinburgh, United Kingdom; 4 Centre for Genomic and Experimental Medicine, University of Edinburgh, Edinburgh, United Kingdom; 5 Centre for Cognitive Ageing and Cognitive Epidemiology, University of Edinburgh, Edinburgh, United Kingdom; 6 Queensland Brain Institute, The University of Queensland, Queensland, Australia

## Abstract

Aging is a complex biological process regulated by multiple cellular pathways and molecular mechanisms including epigenetics. Using genome-wide DNA methylation data measured in a large collection of Scottish old individuals, we performed discovery association analysis to identify age-methylated CpGs and replicated them in two independent Danish cohorts. The double-replicated CpGs were characterized by distribution over gene regions and location in relation to CpG islands. The replicated CpGs were further characterized by involvement in biological pathways to study their functional implications in aging. We identified 67,604 age-associated CpG sites reaching genome-wide significance of FWER <0.05, 86% demethylated with increasing age. Double-replication resulted in 5,168 CpGs (39% age-methylated and 61% age-demethylated) which were characterized by high concentration of age-methylated CpGs at 1stExon and TSS200 and a dominant pattern of age-demethylated CpGs at other gene regions, and by overwhelming age-related methylation in CpG islands and demethylation at shore/shelf and open sea. The differential distribution patterns over gene regions for methylated and demethylated CpGs both relate to reduced gene activity during aging. Pathway analysis showed that age-dependent methylations were especially involved in cellular signalling activities while demethylations particularly linked to functions of the extracellular matrix, all implicated in the aging process and age-related disease risk.

## Introduction

Current development in genomic analysis technologies such as the DNA microarrays and next generation sequencing (NGS) has enabled in-depth analysis of the epigenome. In the literature, age and aging associated epigenetic profiles have been detected by the epigenome-wide association studies (EWAS) using cross-sectional and longitudinal designs, respectively^1–6^. These studies have reported a considerable number of genomic sites differentially methylated over different ages, and during aging within the same individuals. Based on the significant CpG sites from EWAS, biological pathway analysis revealed important functional pathways concerning cell-cell signalling, synaptic transmission and multiple signalling pathways that overlap across studies^3, 5^. Although these were mainly obtained from the DNA methylome of whole blood corrected for cell composition, the identified pathways could reflect the generic aging-related epigenetic changes that are not tissue specific^5^.

Aging is a complex biological process that involves numerous changes at various levels and in different organ systems from molecular modification to the functional regulation of systems through multiple biological mechanisms including epigenetics^7, 8^. As a reflection of this, most of the epigenetic association analyses of human aging have reported relatively large numbers of differentially regulated sites^4–6^. While the reported findings could reflect extensive involvement of epigenetic modification during the aging process, careful validation of the findings are especially necessary both for reducing false discovery and for better characterizing the epigenetic changes accompanying human aging.

Based on genome-wide DNA methylation measurements from a large collection of blood samples from older people, we conducted a EWAS on aging to look for CpG sites differentially methylated with increasing age using a double validation scheme of independent samples. The double-replicated CpGs were characterized by grouping across genomic regions and by investigating their functional pathways implicated in the dynamic patterns of age-related epigenetic modifications.

## Results

### EWAS and replication analysis

**Figure 1:**
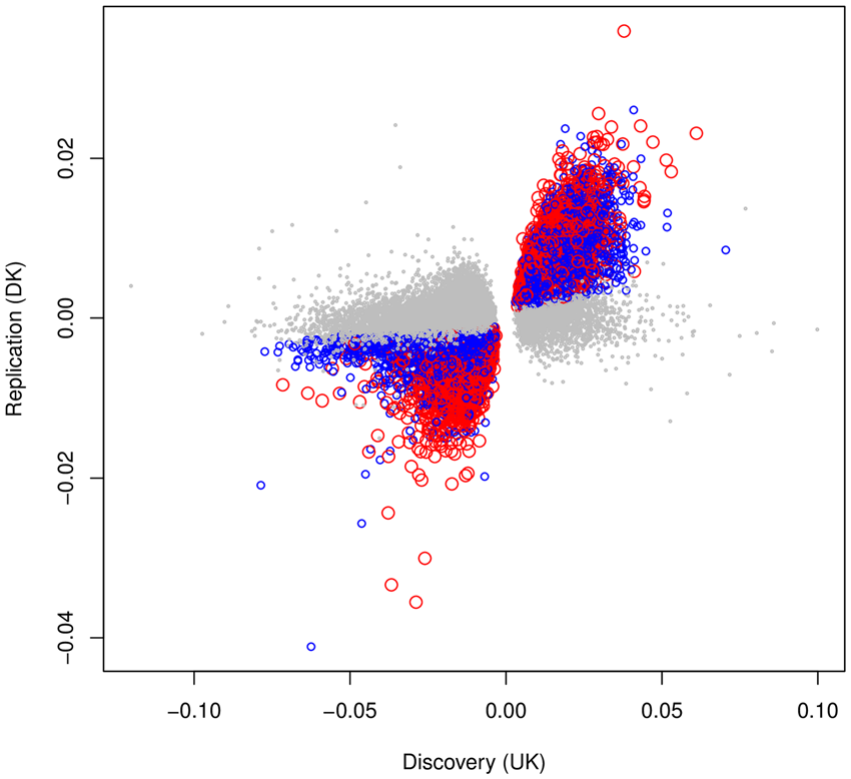
Replication results for the 67,604 significant CpGs identified in the discovery stage. The CpGs coloured with blue and red are the 19,768 CpGs replicated by the Danish cross-sectional twin samples. The red dots represent specifically the 5,168 CpGs double replicated by both Danish cross-sectional and longitudinal samples.

Following the procedure described in Methods section, the EWAS on LBC samples identified a total of 67,604 CpGs with FWER < 0.05. Among them, 9,688 CpGs (14%) were age-methylated and 57,916 CpGs (86%) were age-demethylated. The results show a predominant pattern of demethylation with increasing age in the significant CpGs. Replication analysis of these CpGs in the Danish cross-sectional twin samples found 19,768 CpGs showing same direction of change (increase or decrease) over age with p < 0.05, a replication rate of 29.2%. Replication using the Danish longitudinal twins resulted in 7,238 CpGs with same direction of age pattern and p value < 0.05, a replication rate of 10.7%. Interestingly, among the CpGs replicated in the Danish longitudinal twin samples, 5,168 overlap with the 19,768 CpGs replicated in the cross-sectional samples, an overlapping rate of 70.4%. Among the double-replicated 5,168 CpGs, 2,028 CpGs (39%) were age-methylated and 3,140 (61%) were age-demethylated, again exhibiting a prevailing pattern of demethylation with age (see Supplementary Table S1). In Fig. 1, we show the replication results by plotting regression coefficients of the 67,604 significant CpGs from the discovery LBC samples (the horizontal axis) against corresponding coefficients from the cross-sectional validation samples (the vertical axis) with replicated CpGs marked blue. On top of that, we further display the double replicated CpGs with red colour. The double-replicated CpGs tend to be scattered further away from the unreplicated CpGs coloured by light grey suggesting increased reliability. In the subsequent analysis, we focused on the 5,168 double-replicated CpGs.

### Distribution of age-related CpG sites over gene region

**Figure 2:**
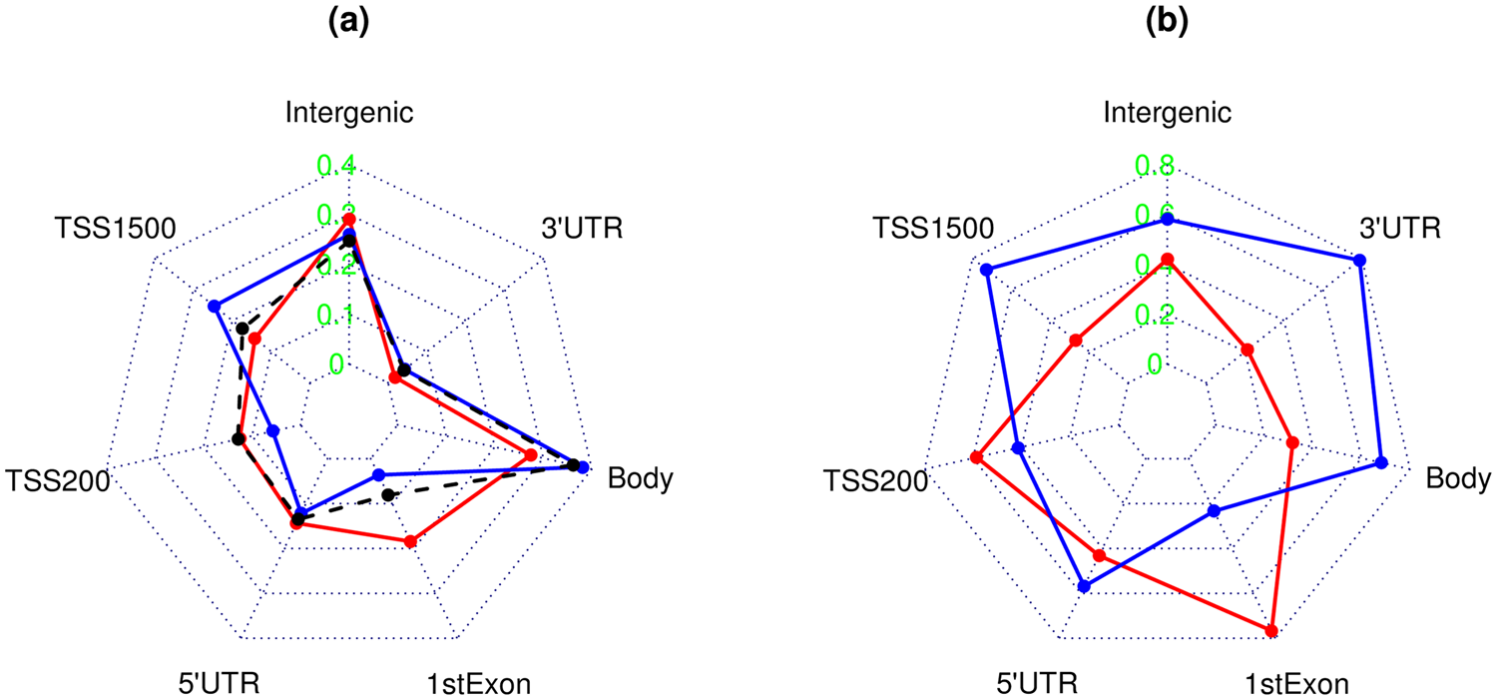
Starplots showing (**a**) proportionality of gene regions for the age-methylated (red) and demethylation (blue) CpGs; and (**b**) region-specific proportion of age-methylated (red) and demethylated (blue) CpGs in all double-replicated CpGs in the gene region.

The double-replicated 5,168 CpGs were first divided over gene regions separately for CpGs that displayed increased and decreased methylation patterns with age. The proportions of CpGs by gene region in the age-methylated or demethylated groups (columns 3 and 6 in Table 1) were then compared to the corresponding proportions for all CpGs in the whole array (column 2 in Table 1) to see if some gene regions were over-represented in each of the two groups. Figure 2a displays the proportion of CpGs by gene region in the groups of increased (red curve) and decreased (blue curve) methylation with the dashed curve representing proportion in the overall array. It is clear that the age-dependent DNA methylation is more frequent at 1stExon (18.44% vs 8.09%) but less frequent in the gene body (27.42% vs 36.09%). In contrast, the age-associated demethylation happens more frequently at the region covering −200 to −1500 nt upstream of the transcription start site (TSS) (TSS1500) (24.65% vs 17.34%) but less frequently at the region up to −200 nt upstream of TSS (TSS200) (5.73% vs 12.87%) and 1stExon (3.63% vs 8.09%). The differential patterns are supported by the very high statistical significance in Table 1.

**Table 1:** Proportionality of gene regions for all CpGs of the array and in age-methylated (gain) and demethylated (loss) CpGs.

Gene region	Whole array	Gain	χ^2^	P value	Loss	χ^2^	P value
Intergenic	0.25	0.29	21.27	3.99E-06	0.26	2.82	9.33E-02
TSS1500	0.17	0.14	13.93	1.90E-04	0.25	114.38	1.08E-26
TSS200	0.13	0.13	0.22	6.40E-01	0.06	142.40	7.93E-33
5′UTR	0.14	0.14	1.18	2.77E-01	0.12	4.41	3.57E-02
1stExon	0.08	0.18	286.57	2.78E-64	0.04	83.66	5.89E-20
Body	0.36	0.27	66.57	3.38E-16	0.38	5.10	2.39E-02
3′UTR	0.04	0.02	26.65	2.44E-07	0.04	0.28	6.00E-01

In addition to comparing the relative proportions of CpGs by gene region, we also compared the absolute proportions of age-dependent methylation and demethylation in the double-replicated CpGs at each gene region (Table 2). Most of the gene regions had more demethylated than methylated CpGs except for TSS200 and 1stExon. This is easily seen in Fig. 2b where most of the red curve for age-methylated CpGs is inside the blue curve for demethylated CpGs excluding TSS200 and 1stExon. The differential pattern is again extremely significant as indicated by p values in Table 2.

**Table 2:** Proportion of age-methylated (gain) and demethylated (loss) CpG in each region.

Gene region	Replicated CpGs	Gain	%	Loss	%	χ^2^	P value
Intergenic	1405	590	0.42	815	0.58	71.42	2.88E-17
TSS1500	1062	288	0.27	774	0.73	442.98	2.43E-98
TSS200	434	254	0.59	180	0.41	24.56	7.21E-07
5′UTR	674	291	0.43	383	0.57	24.57	7.16E-07
1stExon	488	374	0.77	114	0.23	274.92	9.60E-62
Body	1753	556	0.32	1197	0.68	467.31	1.23E-103
3′UTR	170	36	0.21	134	0.79	110.69	6.90E-26

### Distribution of age-related CpG sites in relation to CpG island (CGI)

**Figure 3:**
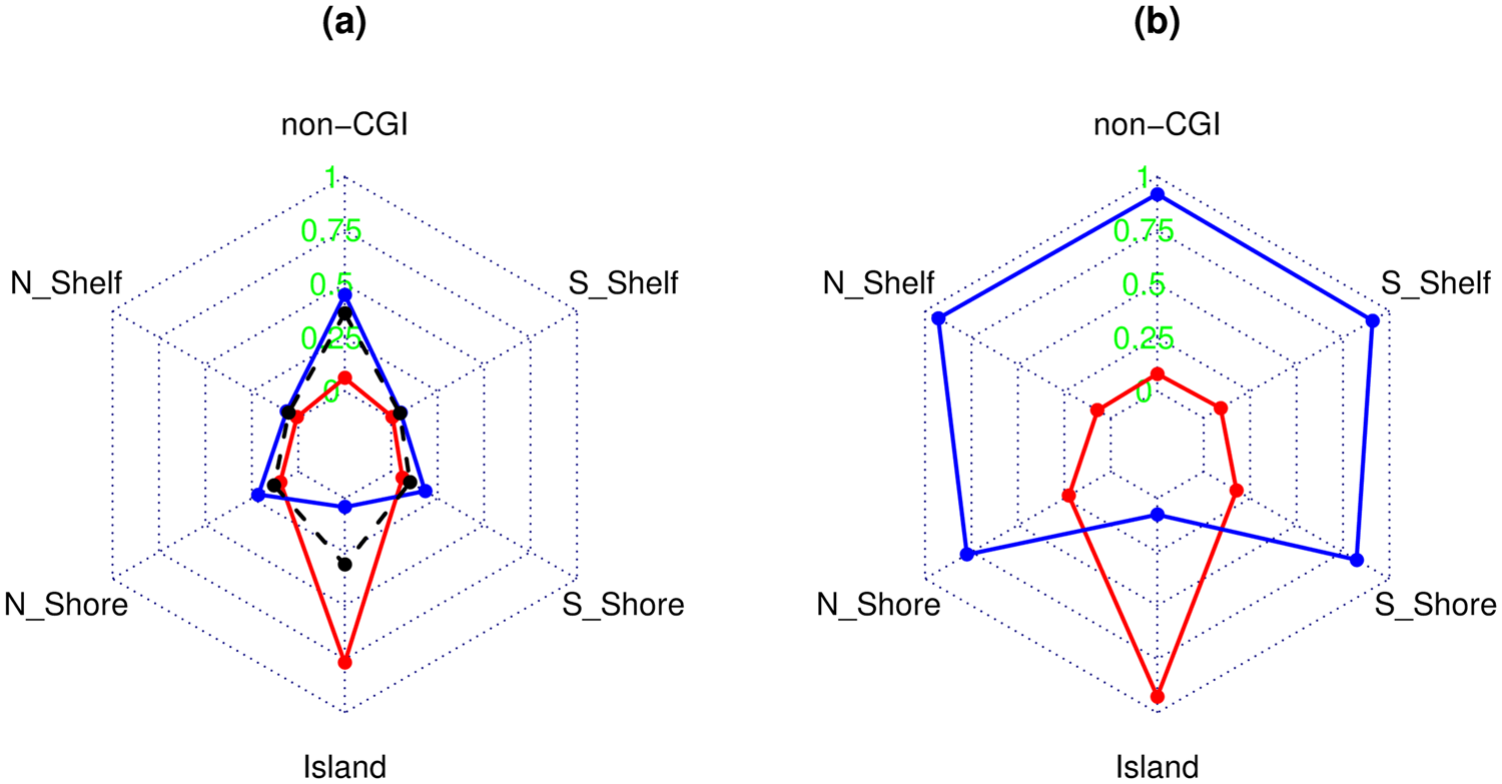
Starplots showing (**a**) proportionality of locations in relation to CpG island for the age-methylation (red) and demethylation (blue) CpGs; and (**b**) location-specific proportion of age-methylated (red) and demethylated (blue) CpGs in all double-replicated CpGs in the location.

Similar to the characterization by gene region, we next grouped the age-associated CpGs by their relation to CGI. We started with comparing the relative proportions of CpGs at their locations to CGI (island, shore, shelf, non-CGI) with the corresponding proportions for all CpGs in the whole array (Table 3, Fig. 3a). As shown by Fig. 3a, the age-methylated CpGs are predominantly distributed to the island (76.63% vs 30.89%) and rarely to the non-CGI region (6.11% vs 36.38%). On the contrary, the age-demethylated CpGs are more frequently distributed over all locations except the island. Comparison of the absolute proportions of age-dependent methylation and demethylation in significant CpGs at each location is presented in Table 4 and Fig. 3b. The significant CpGs at the island are mostly age-methylated (92.39%) while the rest of locations are mainly occupied by age-associated demethylation.

**Table 3:** Proportionality of locations to CGI for all CpGs of the array and in age-methylated (gain) and demethylated (loss) CpGs.

Relation to CGI	Whole array	Gain	χ^2^	P value	Loss	χ^2^	P value
non-CGI	0.36	0.06	793.97	3.99E-06	0.45	101.92	5.79E-24
N_Shelf	0.05	0.01	79.07	5.99E-19	0.06	7.89	4.97E-03
N_Shore	0.13	0.10	19.05	1.27E-05	0.21	197.75	6.48E-45
Island	0.31	0.77	1963.21	0	0.04	1056.14	1.12E-231
S_Shore	0.10	0.06	36.31	1.68E-09	0.18	221.97	3.36E-50
S_Shelf	0.05	0.01	66.05	4.41E-16	0.05	1.47	2.25E-01

**Table 4:** Proportion of age-methylated (gain) and demethylated (loss) CpG by location to CGI.

Relation to CGI	Replicated CpGs	Gain	%	Loss	%	χ^2^	P value
non-CGI	1536	124	0.08	1412	0.92	2156.73	0
N_Shelf	211	15	0.07	196	0.93	307.11	9.31E-69
N_Shore	869	196	0.23	673	0.77	521.46	2.03E-115
Island	1682	1554	0.92	128	0.08	2414.54	0
S_Shore	695	123	0.18	572	0.82	577.57	1.27E-127
S_Shelf	175	16	0.09	159	0.91	230.45	4.77E-52

**Figure 4:**
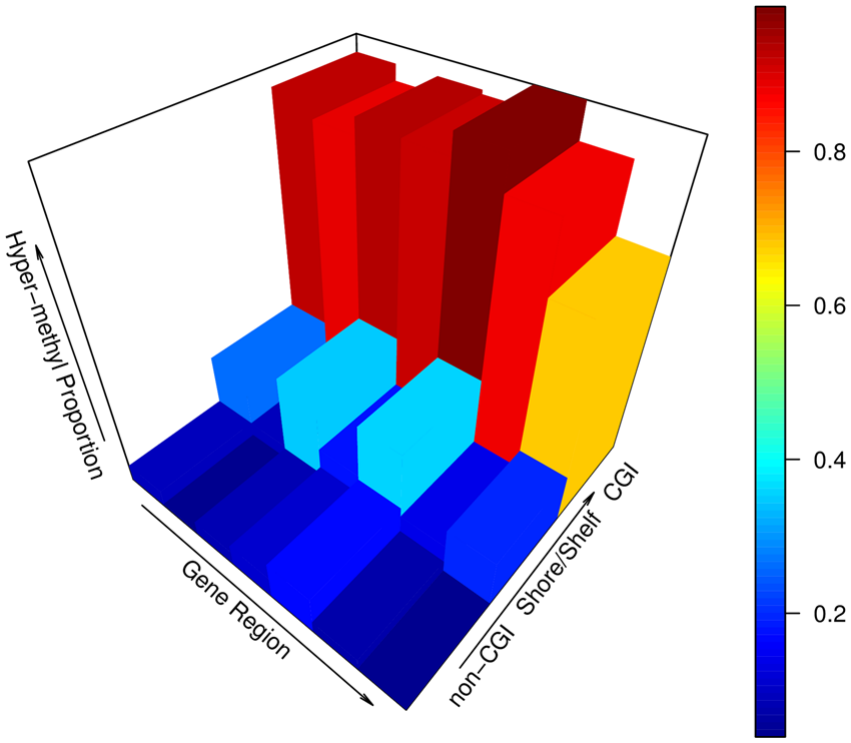
A 3-dimensional histogram showing the proportion of age-methylated CpGs at each combination of gene regions (intergenic region, TSS1500, TSS200, 5′UTR, 1stExon, gene body, 3′UTR) and locations to CpG island (from open sea or non-CGI to shore/shelf and to CGI). It is clearly shown that methylation level at each gene region is mainly driven by age-associated ﻿methylation (gain) at CGI.

After separately examining the differential methylation patterns by gene region and by relative location to CGI, we further divided the 5,168 CpGs by both gene region and CGI location and compared the proportions of age-associated methylation and demethylation at each combination to examine the methylation patterns across gene regions for a given CGI location or across CGI locations (Table 5). Table 5 shows that increased methylation is consistently observed in very high proportion at CGIs in all gene regions and in low proportion (thus high proportion of demethylation) at shore/shelf and non-CGI. This is visualized in Fig. 4 where it is shown that the proportion of age-methylated CpGs is mainly determined by relative location of the CpG sites to CGI with only minor variation across gene regions. Inspired by Fig. 4, we plot the proportion of age-methylated CpGs at each gene region together with proportion of CpGs located at the island from each gene region in Supplementary Figure S1. It is shown that the proportion of age-methylated CpGs by gene region is closely correlated with the proportion of CpGs residing at CGI in the region (correlation coefficient 0.995) again indicating that the differential methylation pattern across gene regions is highly related to region-specific composition of CpGs at CGI, shore/shelf or open sea.

**Table 5:** Proportion of age-methylated CpGs in double-replicated CpGs by combination of gene region and relation to CGI.

Gene region	Non-CGI				Shore/Shelf				CGI			
	Gain	Loss	%	P value	Gain	Loss	%	P value	Gain	Loss	%	P value
Intergenic	423	27	0.94	7.97E-153	118	327	0.27	3.41E-44	49	461	0.10	4.41E-146
TSS1500	221	26	0.89	3.05E-68	57	542	0.10	4.09E-172	10	206	0.05	1.49E-78
TSS200	206	13	0.94	3.41E-75	39	72	0.35	1.74E-05	9	95	0.09	4.53E-32
5′UTR	224	17	0.93	1.43E-78	48	216	0.18	7.22E-48	19	150	0.11	2.09E-45
1stExon	333	4	0.99	7.15E-141	29	51	0.36	8.99E-04	12	59	0.17	1.16E-14
Body	413	51	0.89	3.54E-124	100	630	0.14	9.44E-169	43	516	0.08	2.33E-175
3′UTR	20	9	0.69	8.64E-03	13	52	0.20	2.64E-11	3	73	0.04	4.40E-29

### Pathway analysis

The 5,168 double-replicated CpGs in Supplementary Table S1 were grouped into age-methylated (2,028) and demethylated (3,140) CpGs which were annotated to 578 and 1,051 nearest genes respectively. The lists of linked genes were submitted to the GSEA website to look for canonical pathways in MSigDB over-represented by each gene list. Large numbers of pathways were identified from which the top 50 are presented in Supplementary Table S2 for age-methylated CpGs and Supplementary Table S3 for age-demethylated CpGs. It is interesting to see that, among the top 10 gene sets in the two tables, only 1 gene set overlaps, NABA matrisome. The top significant pathways in Supplementary Table S2 are dominated by cellular signalling activities involving, for example, G protein-coupled receptors (GPCRs) while in Supplementary Table S3 by gene activities mainly involving the extracellular matrix (ECM).

## Discussion

Based on a relatively large collection of samples from older people and using a double replication strategy, we have identified 5,618 CpGs that changed their white blood cell methylation profiles in the aging cohorts with majority of them demethylated over increasing age. The significantly higher proportion of demethylation (60.76%) than methylation (39.24%) (p < 2.2e-16) among the double-replicated CpGs is not surprising as global decline in DNA methylation level of the methylome is the predominant event in aging^9^. The excessive pattern of demethylation over methylation with aging has also been reported as 64% by Johansson *et al*.^10^ using 421 individuals and as 54% by Marttila *et al*.^4^ using 143 individuals, with the former very close to our estimate.

Characterization of the double replicated CpGs by their distribution over gene regions was done first by examining the relative and absolute proportions of methylated and demethylated CpGs at each gene region. From the Tables 1 and 2 and Fig. 2, it is clear that there are excessive age-related gains in methylation at TSS200 and 1stExon and the rest of gene regions are characterized by loss of methylation. In the literature, it has been shown that increased methylation surrounding TSS is associated with gene inactivation^11^ and also methylation of the 1stExon is linked to gene silencing^12^. On the other hand, demethylation in the gene body is associated with suppression of gene expression^13^. Taking together, the differential distributions of both methylated and demethylated CpGs over gene region can all be related to the reduced gene activity during aging.

Our estimates of relative proportions (Tables 1 and 3, Figs 2a and 3a) revealed similar patterns of distribution of differentially methylated sites across gene regions as described by Marttila *et al*.^4^. The consistent findings suggest that age-associated DNA methylations are differentially distributed over gene regions as compared with the total CpGs carried by the microarray used in the studies. The differential distribution of age-associated methylation and demethylation across gene regions (Tables 1 and 2, Fig. 2) and over locations to CpG island (Tables 3 and 4, Fig. 3) could implicate different biological functions. It is interesting to see that, our analysis revealed, indeed, different functional clusters or gene sets over-represented by genes linked to age-methylated and demethylated CpGs (see Supplementary Tables S2 and S3). In Supplementary Table S2, the top significant canonical pathways are nearly all related to signalling pathways such as GPCR (G-protein coupled-receptors) signalling widely involved in overall physiological functions and pathological processes as well as progression of Alzheimer’s disease^14^. Cell-cell signalling was also reported by Florath *et al*.^3^, Marttila *et al*.^4^ and Tan *et al*.^5^ in their top significant pathways regulated by age-associated increase in DNA methylation. Interestingly, in an early microarray study, Tan *et al*.^15^ compared genome-wide gene expression between grandparents and grandchildren in the CEPH Utah families and reported cell-cell signalling and cell communication pathways as the top-most significant pathways enriched by differentially expressed genes dominated by decreased expression pattern with aging. Given the high concentration of age-methylated CpGs at gene promotor region that silences gene activity, the finding in this study could provide epigenetic evidence on the significant age-associated impairment in cellular signalling transduction during the aging process.

Although demethylation is found more frequently than methylation in the aging methylome, functional annotation of age-associated demethylation sites identified by Johansson *et al*.^10^ and Marttila *et al*.^4^ failed to show any significant enrichment for specific functional pathways. Based on the double replicated CpGs, our pathway analysis on genes linked to the significantly demethylated CpG sites identified a large number of gene-sets with the topmost ones unanimously involving genes encoding extracellular matrix (ECM) and ECM-associated proteins (see Supplementary Table S3). The ECM is a collection of molecules (sugars, proteins, etc.) that function not only as a scaffold but also as a place where signals affecting cell migration and differentiation originate. The ECM has been a central topic of discussion in aging studies^16^. It is associated with numerous age-related diseases and dysfunctions, most of them involving elasticity and strength of connective tissue, cartilage, bone, blood vessels, and skin^17^. In fact, the role of ECM goes beyond structural properties of tissue. For example, degradation of ECM in heart tissues could impact the heart’s electrical conduction system, a probable contributor to the increased prevalence of arrhythmias and similar issues with advancing age^18^. In this sense, the top significant pathways from both age-methylated and demethylated CpGs overlap in regard to cell signalling. This is further evidenced by the gene set NABA_MATRISOME which appears as top-most significant gene set in both Supplementary Tables S2 and S3.

Based on genome-wide DNA methylation data measured over ages, Horvath^19^ and Hannum *et al*.^20^ have selected multiple age predictive CpG sites for defining DNA methylation age (DNAm age). It is interesting that among the 71 CpGs in the list from Hannum *et al*.^20^ or the Hannum list, 54 (76.06%) are present in our 5,168 double-replicated CpGs, while of the 353 CpGs in the list from Horvath^19^, only 32 (9.07%) overlapped. This large difference in the overlapping rates can be due to the fact that the Horvath list was developed from relatively younger samples (mean age 43 years) while the mean age of the samples from Hannum *et al*.^20^ was more than 20 years older which is closer to the age ranges covered in our discovery and replication samples. This evidence further shows that our current study is featured by discovery, validation and characterization of DNA methylation patterns associated with the aging process in the older population.

Finally, we want to point out that, although high overlapping rates had been found in the replication analysis, the number of the double-replicated CpGs could have been considerably reduced by the relatively small sizes of the replication samples. Some of the truly significant CpGs from the discovery stage could have failed in the replication stage purely due to the fact that the latter did not have enough statistical power to confirm their significance. Under this situation, the double-replicated CpGs could have been biased towards CpGs with large effect sizes as indicated by the red spots in Fig. 1.

In conclusion, profiling of the aging methylome in combination with a double-replication strategy identified 5,168 significantly age-associated CpGs with majority of them being demethylated with increasing age. The detected CpGs are characterized by high concentration of gain in methylation at 1stExon and TSS200 and a dominant pattern of loss of methylation at other gene regions and by overwhelming age-associated methylation in CpG island and demethylation at shore/shelf and open sea. Biological pathway analysis showed that age-related increase in DNA methylation is especially involved in cellular signalling activities while age-dependent demethylation is particularly related to functions of the extracellular matrix, both provide clues to epigenetic remodelling behind the aging process and age-related diseases.

## Methods

### The discovery samples

The discovery analysis was based on samples collected from the Lothian Birth Cohorts (LBC) consisting of individuals born in 1921 and 1936 who resided in the Lothian region of Scotland at ages about 79 years (born 1921, 515 individuals) and 70 years (born 1936, 1,005 individuals), respectively^21^. A total of 2,460 whole blood samples were collected from the 1,520 participants during follow-up periods of about 10 years for the 1921 birth cohort, and about 6 years for the 1936 birth cohort. After sample level quality control (QC) on DNA methylation data measured on whole blood using the Illumina Infinium Human DNA methylation 450 K array, 2,195 samples from 1,366 individuals remained. DNA methylation measurements were available for 485,512 CpG sites from the array. We removed sites on sex chromosomes leaving 473,864 autosomal CpGs for subsequent analysis. The LBC data have been deposited to the European Genome-phenome Archive (https://www.ebi.ac.uk/ega/home) with accession number EGAS00001000910.

Ethics permission for the LBC1921 was obtained from the Lothian Research Ethics Committee (Wave 1: LREC/1998/4/183). Ethics permission for the LBC1936 was obtained from the Multi-Center Research Ethics Committee for Scotland (Wave 1: MREC/01/0/56), the Lothian Research Ethics Committee (Wave 1: LREC/2003/2/29). Written informed consent was obtained from all subjects. The study was conducted in accordance with the principles of the Helsinki Declaration.

### The validation samples

#### The cross-sectional Danish twins

The cross-sectional sample for validation consisted of 150 pairs of identical twins aged 30 to 74 years^22^. The sample can be divided into two age groups, i.e. a young group aged from 30–37 years (154 samples), and an old group aged 57–74 years (146 samples) with a gap of 20 years between the two groups. DNA methylation was measured using the same array platform as in discovery samples. Both raw and processed DNA methylation data for the cross-sectional samples have been deposited to the NCBI GEO database (http://www.ncbi.nlm.nih.gov/geo) under accession number GSE61496.

The study was approved by The Regional Scientific Ethical Committees for Southern Denmark (S-20090033) and conducted in accordance with the Helsinki II declaration. Informed consent was obtained from all subjects.

#### The longitudinal Danish twins

The longitudinal sample for validation included of 86 elderly Danish twins (18 monozygotic or MZ pairs; 25 dizygotic or DZ pairs) collected by the Longitudinal Study of Aging Danish Twins (LSADT) initiated in 1995^5, 23^. The project collected like-sex twin pairs born in Denmark for longitudinal assessment of aging-related phenotypes. The participants were born before 1923 with age ranging from 73–82 years in 1997 when first blood samples were taken. The second blood samples were drawn in 2007 after a ten-year follow-up. Genome-wide DNA methylation was measured using the same platform as in the discovery samples (i.e. Illumina Infinium Human DNA methylation 450 K beadchip). The raw and processed DNA methylation data have been deposited to the NCBI GEO database http://www.ncbi.nlm.nih.gov/geo under accession number GSE73115.

The study was approved by the Danish Scientific Ethics Committees and conducted in accordance with the Helsinki II declaration. Informed consent was obtained from all subjects.

### Estimating and adjusting cell composition

It has been shown that cell composition in the whole blood can change as a result of aging^2^ and it is thus critical to account for cellular heterogeneity in epigenetic studies of aging^24^. Based on the downloaded β-value for DNA methylation percentage for LBC, we estimated blood cell composition in each individual sample for 6 blood cell types: CD8T, CD4T, natural killer cell, B cell, monocyte, and granulocyte using the R package *celltypes450* (https://github.com/brentp/celltypes450). The package estimates cell-type composition in 450 K data using Houseman’s method^25^. Cell compositions for the Danish validation samples were estimated by applying the R package *minfi* (http://bioconductor.org/packages/release/bioc/html/minfi.html) to measured signals derived from the green and red channels^26^.

### Data analysis

The age-dependent DNA methylation patterns were analysed by fitting a regression model to each CpG site regressing methylation measurement on individual’s age at sampling. Before fitting the model, DNA methylation percentage, i.e. the β-value, was transformed into M-value using logit transformation to ensure normal or approximately normal distribution. Besides age, the regression model also included the estimated blood cell composition for adjustment. Considering that the LBC samples contained repeated measurements over follow-ups, statistical significance of age-dependent association of DNA methylation was assessed empirically using computer permutation that randomized age at observation to generate a null distribution of random p values. In our analysis, the computer permutation was combined with correction for multiple testing by estimating family-wise error rate (FWER). To do that, for every random EWAS performed on age permuted data, a minimum p value was recorded. A null distribution of minimum p values was created based on K (we used K = 300) random EWAS. FWER for each CpG was estimated as $\mathrm{FWER}=\frac{{\sum }_{i=1}^{K}I[{\rm{Pvalue}}({\rm{perm}})({\rm{i}}) < \mathrm{Pvalue}\,({\rm{obs}})]}{K}$.

The analyses of validation data were done by fitting mixed effect models as described in Tan *et al*.^5^. All statistical analyses were performed using the free R software (https://www.r-project.org) and related packages.

### Pathway analysis

The identified significant CpGs (FWER<0.05) were annotated to nearest genes and evaluated for over-representation of gene-sets or pathways in the Molecular Signatures Database (MSigDB). The over-representation analysis compares a reference set of genes to a test gene-set using the hypergeometric test. The probability of finding X > k significant genes in a particular gene-set or pathway can be calculated using the hypergeometric distribution, i.e. $$p(X > k)=1-\sum _{r=0}^{k}(\begin{array}{c}m\\ r\end{array})(\begin{array}{c}N-m\\ n-r\end{array})/(\begin{array}{c}N\\ n\end{array})$$, where N is the number of genes annotated to CpGs on the 450 K array, m is the number of genes annotated to the significant CpGs, n is the number of genes in the particular gene-set or pathway, and k is the number of genes that are both linked to significant CpGs and present in the particular gene-set or pathway. The analysis was performed using the analytical tool provided by Gene-Set Enrichment Analysis (GSEA) (http://www.broadinstitute.org/gsea/index.jsp).

## Electronic supplementary material

Supplementary information
