# Supplementary material for: Identification, replication and characterization of epigenetic remodelling in the aging genome: a cross population analysis
Source: Sci Rep. 2017 Aug 15;7:8183. doi: 10.1038/s41598-017-08346-7 (PMC5557932; doi:10.1038/s41598-017-08346-7)
Supplement: Supplementary file 1 — Supplementary information [file 41598_2017_8346_MOESM1_ESM.pdf]

Supplementary information

# Identification, replication and characterization of epigenetic remodeling in the aging genome: a cross population analysis

Shuxia Li[1], Lene Christiansen[2], Kaare Christensen[2], Torben A. Kruse[1], Paul Redmond[3], Riccardo E. Marioni[4,5,6], Ian J. Deary[3,5], Qihua Tan[1,2,*]

1. Unit of Human Genetics, Department of Clinical Research, University of Southern Denmark, Odense, Denmark
2. Epidemiology, Biostatistics and Biodemography, Department of Public Health, University of Southern Denmark, Odense, Denmark
3. Department of Psychology, University of Edinburgh, Edinburgh, United Kingdom
4. Centre for Genomic & Experimental Medicine, University of Edinburgh, Edinburgh, United Kingdom
5. Centre for Cognitive Ageing and Cognitive Epidemiology, University of Edinburgh, Edinburgh, United Kingdom
6. Queensland Brain Institute, The University of Queensland, Australia

# Supplementary Table S1. List of double-replicated CpG sites

| CpG | RefGene_Name | RefGene_Group | Relation_to_CGI | Coefficient | p value |
|---|---|---|---|---|---|
| cg00001747 | NA | NA | Island | 2.20E-02 | 3.13E-06 |
| cg00007076 | RRS1;RRS1 | 1stExon;3'UTR | N_Shore | -1.62E-02 | 2.44E-16 |
| cg00008033 | ZNF613;ZNF613 | TSS1500;TSS1500 | NA | 1.10E-02 | 8.23E-08 |
| cg00008036 | FAM163A | TSS1500 | Island | 8.74E-03 | 3.46E-06 |
| cg00008629 | ROD1;ROD1;ROD1 | Body;Body;5'UTR | N_Shore | -3.44E-02 | 9.58E-15 |
| cg00010853 | KIAA1949;KIAA1949 | Body;1stExon | N_Shore | -1.68E-02 | 4.40E-16 |
| cg00012576 | GPC6 | Body | NA | -1.14E-02 | 2.56E-11 |
| cg00018128 | TBR1 | Body | S_Shore | 1.24E-02 | 2.14E-07 |
| cg00019759 | NA | NA | N_Shore | 8.12E-03 | 1.11E-08 |
| cg00020991 | CBR4 | Body | N_Shore | -2.22E-02 | 1.49E-26 |
| cg00025981 | C13orf15 | TSS1500 | Island | 2.01E-02 | 1.49E-05 |
| cg00027083 | EPB41L3 | 5'UTR | Island | 2.92E-02 | 4.48E-05 |
| cg00028135 | ZBTB20;ZBTB20;ZBT | 5'UTR;5'UTR;TSS15( | NA | -1.92E-02 | 7.41E-14 |
| cg00030420 | SLC25A2 | TSS200 | Island | 1.24E-02 | 1.77E-06 |
| cg00034076 | RALYL;RALYL;RALYL; | 5'UTR;TSS1500;5'U | N_Shore | 2.72E-02 | 1.32E-08 |
| cg00034468 | ACTA1 | Body | Island | 1.04E-02 | 1.03E-10 |
| cg00036732 | LZTS2 | 3'UTR | S_Shore | -9.95E-03 | 8.78E-09 |
| cg00036871 | OBSL1;INHA | TSS1500;TSS200 | S_Shore | -3.88E-03 | 1.87E-05 |
| cg00037441 | RFX6;RFX6 | 5'UTR;1stExon | Island | 6.75E-03 | 2.57E-05 |
| cg00038020 | NA | NA | NA | -2.06E-02 | 4.87E-15 |
| cg00040575 | MICAL3;MICAL3;MI( | 5'UTR;5'UTR;5'UTR | N_Shore | -1.07E-02 | 6.18E-14 |
| cg00048759 | STAG3;GPC2 | TSS200;TSS1500 | Island | 8.90E-03 | 6.65E-07 |
| cg00049323 | LOC25845 | Body | N_Shore | -1.01E-02 | 3.64E-05 |
| cg00053851 | NA | NA | N_Shore | -1.75E-02 | 4.85E-13 |
| cg00055679 | NA | NA | Island | 8.10E-03 | 1.61E-06 |
| cg00057272 | ZFAT | TSS1500 | S_Shore | -1.39E-02 | 1.24E-05 |
| cg00059225 | GLRA1;GLRA1;GLRA | 1stExon;1stExon;5'U | Island | 9.71E-03 | 4.02E-13 |
| cg00060836 | SNHG6 | Body | N_Shelf | -1.89E-02 | 5.67E-09 |
| cg00062437 | NA | NA | N_Shore | -4.19E-03 | 7.54E-05 |
| cg00063748 | PRDM16;PRDM16 | 3'UTR;3'UTR | S_Shore | -9.25E-03 | 4.41E-08 |
| cg00065935 | STXBP5L;STXBP5L | 1stExon;5'UTR | Island | 1.01E-02 | 8.52E-07 |
| cg00068433 | ARHGEF19 | TSS1500 | N_Shelf | -1.21E-02 | 5.01E-12 |
| cg00069242 | JUB | 1stExon | Island | 1.17E-02 | 2.48E-07 |
| cg00073460 | ZC3H12D | TSS1500 | NA | -1.23E-02 | 9.31E-07 |
| cg00073837 | NA | NA | Island | 9.96E-03 | 5.02E-07 |
| cg00076555 | BSN | 3'UTR | NA | -6.81E-03 | 2.58E-06 |
| cg00086710 | PPM1K | 5'UTR | N_Shore | -1.61E-02 | 1.99E-07 |
| cg00090737 | KNDC1 | Body | S_Shore | -8.44E-03 | 1.34E-05 |
| cg00094518 | KLF14 | 1stExon | Island | 1.75E-02 | 3.68E-06 |
| cg00101260 | NA | NA | NA | -1.41E-02 | 6.95E-16 |
| cg00103778 | NA | NA | N_Shore | -1.34E-02 | 4.79E-10 |
| cg00105628 | NA | NA | Island | 1.11E-02 | 4.18E-05 |
| cg00108098 | SEMA5B | Body | N_Shelf | -7.59E-03 | 1.75E-06 |
| cg00109764 | NA | NA | NA | -8.26E-03 | 2.28E-11 |
| cg00114784 | NA | NA | NA | -7.70E-03 | 4.52E-07 |
| cg00127150 | ZNF280B | 5'UTR | N_Shore | -1.53E-02 | 4.11E-14 |
| cg00134210 | FAM107B | Body | N_Shore | -8.61E-03 | 7.40E-05 |

| | | | | | |
|---|---|---|---|---|---|
| cg00144186 | FJX1 | 1stExon | Island | 7.59E-03 | 3.75E-05 |
| cg00154846 | C1orf86 | Body | S_Shelf | -7.08E-03 | 6.43E-07 |
| cg00168942 | GJD4;GJD4 | 1stExon;5'UTR | N_Shelf | -1.66E-02 | 1.96E-18 |
| cg00173659 | NA | NA | Island | 1.04E-02 | 1.66E-05 |
| cg00174179 | RHOA;TCTA | TSS1500;Body | S_Shore | -5.25E-03 | 3.25E-05 |
| cg00174851 | AKR1B1 | Body | N_Shore | -9.51E-03 | 1.31E-09 |
| cg00174992 | NOSTRIN;NOSTRIN;I | 5'UTR;5'UTR;1stExo | NA | -1.25E-02 | 1.25E-15 |
| cg00178850 | EDARADD;EDARADE | Body;Body | S_Shore | -1.32E-02 | 6.20E-07 |
| cg00178928 | NA | NA | N_Shore | -6.06E-03 | 7.09E-07 |
| cg00182727 | MMP9 | Body | Island | 6.48E-03 | 1.39E-09 |
| cg00187299 | C10orf27 | TSS1500 | NA | -1.03E-02 | 2.23E-07 |
| cg00193668 | HINT2 | Body | N_Shore | -1.41E-02 | 6.99E-19 |
| cg00199059 | TP53RK | Body | N_Shore | -1.43E-02 | 1.33E-08 |
| cg00206490 | ANKRD11 | 5'UTR | NA | 8.34E-03 | 1.90E-05 |
| cg00214791 | NA | NA | S_Shore | 1.52E-02 | 3.44E-08 |
| cg00232105 | NA | NA | Island | -8.25E-03 | 6.90E-09 |
| cg00232500 | FREM1 | TSS1500 | NA | -8.97E-03 | 2.20E-07 |
| cg00237475 | LBXCOR1 | Body | Island | 6.53E-03 | 1.26E-06 |
| cg00237876 | CD300LG;CD300LG;( | TSS1500;TSS1500;T | NA | -1.24E-02 | 1.03E-06 |
| cg00238349 | WDR51A;WDR51A;\ | Body;Body;5'UTR | N_Shore | -7.39E-03 | 1.11E-08 |
| cg00240880 | WISP2 | TSS1500 | NA | -7.62E-03 | 4.76E-05 |
| cg00244299 | NA | NA | NA | -1.20E-02 | 5.18E-08 |
| cg00244776 | VARS2;VARS2;VARS: | Body;5'UTR;Body | S_Shore | -9.98E-03 | 8.52E-09 |
| cg00247557 | LOC642597;LOC642 | 1stExon;5'UTR | Island | 8.28E-03 | 1.78E-05 |
| cg00250645 | NA | NA | NA | -2.21E-02 | 3.29E-06 |
| cg00251125 | OBSCN;OBSCN | TSS1500;TSS1500 | NA | -8.13E-03 | 9.29E-08 |
| cg00252813 | GAPDH | TSS1500 | N_Shore | -6.29E-03 | 7.81E-05 |
| cg00253564 | PLEKHG5;PLEKHG5;I | Body;Body;TSS150C | S_Shore | -9.67E-03 | 3.81E-11 |
| cg00266920 | ST8SIA3 | Body | Island | 1.17E-02 | 3.30E-12 |
| cg00277397 | CALN1;CALN1 | 5'UTR;Body | N_Shore | -1.29E-02 | 2.81E-12 |
| cg00280235 | NRCAM;NRCAM;NR( | 1stExon;5'UTR;Body | NA | -2.50E-02 | 2.07E-05 |
| cg00283986 | NA | NA | S_Shelf | -1.19E-02 | 1.86E-09 |
| cg00292135 | C7orf13;RNF32 | Body;TSS1500 | Island | 1.73E-02 | 1.82E-06 |
| cg00295572 | TP73 | TSS1500 | Island | 2.19E-02 | 9.04E-07 |
| cg00298951 | CMKLR1;CMKLR1;CI | TSS1500;TSS1500;T | NA | -6.47E-03 | 1.36E-05 |
| cg00300637 | AHRR | Body | N_Shore | -8.77E-03 | 5.94E-06 |
| cg00308665 | HTR2A;HTR2A | Body;Body | NA | -2.35E-02 | 1.40E-24 |
| cg00310412 | SEMA7A;SEMA7A;SI | Body;Body;5'UTR | N_Shore | -5.39E-03 | 8.86E-05 |
| cg00314660 | CCDC75;HEATR5B | 5'UTR;TSS1500 | S_Shore | -1.56E-02 | 1.76E-07 |
| cg00318320 | BOK | TSS200 | Island | 1.67E-02 | 1.21E-06 |
| cg00327072 | NEURL1B | Body | Island | 3.49E-02 | 9.19E-05 |
| cg00328870 | NA | NA | N_Shore | -8.51E-03 | 4.11E-09 |
| cg00329272 | PRDM16;PRDM16 | Body;Body | N_Shelf | -5.13E-03 | 4.25E-05 |
| cg00332048 | HDAC9;HDAC9;HDA | TSS1500;TSS1500;T | NA | -1.30E-02 | 9.03E-11 |
| cg00332882 | LOC284788;LOC284 | Body;Body | NA | -8.86E-03 | 4.44E-06 |
| cg00334063 | ZIC4;ZIC4;ZIC4;ZIC4; | 3'UTR;Body;3'UTR;E | N_Shelf | 2.52E-02 | 2.21E-10 |
| cg00336320 | PNPLA1;PNPLA1;PN | 5'UTR;Body;5'UTR | NA | 1.72E-02 | 1.41E-11 |
| cg00337440 | PTK2B;PTK2B;PTK2E | TSS1500;TSS1500;5 | N_Shore | -7.00E-03 | 7.03E-05 |
| cg00339319 | CAPN2;CAPN2 | TSS1500;Body | N_Shore | -5.96E-03 | 5.11E-08 |
| cg00340855 | HLA-E | Body | S_Shore | -1.86E-02 | 7.94E-20 |

| | | | | | |
|---|---|---|---|---|---|
| cg00344900 | NA | NA | Island | 2.71E-02 | 1.15E-12 |
| cg00346208 | VWA5B1 | Body | Island | 2.32E-02 | 6.13E-07 |
| cg00350448 | ARAP1;ARAP1;ARAP | Body;TSS1500;TSS1 | N_Shelf | -6.00E-03 | 6.18E-05 |
| cg00351047 | ILVBL | Body | N_Shore | -6.87E-03 | 8.03E-07 |
| cg00376544 | FBXO39 | TSS1500 | Island | 2.59E-02 | 1.88E-06 |
| cg00379720 | NA | NA | Island | 2.20E-02 | 2.19E-07 |
| cg00384539 | PRDM14 | TSS200 | Island | 2.63E-02 | 1.66E-10 |
| cg00384847 | VPS13D;VPS13D | 5'UTR;5'UTR | S_Shore | -9.13E-03 | 4.94E-10 |
| cg00387658 | CASS4;CASS4;CASS4 | TSS1500;TSS1500;T | NA | -1.56E-02 | 1.43E-21 |
| cg00392257 | ISG20L2 | 1stExon | N_Shore | -1.31E-02 | 4.03E-17 |
| cg00394180 | GDF6 | Body | N_Shelf | 8.18E-03 | 2.91E-05 |
| cg00396667 | PITX1 | 3'UTR | Island | 2.08E-02 | 2.20E-05 |
| cg00397986 | GATA4 | TSS200 | Island | 1.30E-02 | 5.95E-05 |
| cg00399175 | FAM59B | TSS1500 | Island | 9.45E-03 | 1.50E-05 |
| cg00399483 | DCC | Body | Island | 8.88E-03 | 6.18E-07 |
| cg00404053 | RASAL2;RASAL2 | Body;Body | NA | -3.41E-02 | 1.15E-07 |
| cg00410422 | HBXIP | TSS1500 | S_Shore | -1.46E-02 | 3.73E-11 |
| cg00415704 | RGMA;RGMA | Body;5'UTR | N_Shore | -1.02E-02 | 5.38E-13 |
| cg00417323 | NA | NA | S_Shelf | -2.01E-02 | 4.88E-12 |
| cg00432049 | ULBP3 | TSS1500 | S_Shore | -1.03E-02 | 2.04E-06 |
| cg00439658 | GRIN2C | Body | Island | 1.16E-02 | 7.76E-14 |
| cg00439733 | OR6B2 | TSS1500 | NA | -9.46E-03 | 8.45E-07 |
| cg00448560 | ZNF423 | Body | N_Shore | -1.50E-02 | 2.26E-16 |
| cg00451635 | EMP2 | TSS1500 | S_Shore | -7.49E-03 | 2.56E-08 |
| cg00456672 | C2CD4A | TSS1500 | N_Shore | -2.20E-02 | 1.65E-17 |
| cg00458337 | SP2 | TSS1500 | N_Shore | -1.48E-02 | 6.10E-08 |
| cg00458949 | NA | NA | N_Shelf | -8.84E-03 | 1.04E-09 |
| cg00463859 | TCEA3 | Body | N_Shore | -7.85E-03 | 1.15E-06 |
| cg00466500 | CDH22 | Body | Island | 6.40E-03 | 4.25E-05 |
| cg00481951 | SST | Body | N_Shore | 1.75E-02 | 7.03E-15 |
| cg00484358 | ALX3 | Body | Island | 5.81E-03 | 2.78E-08 |
| cg00495775 | HOXD11 | TSS200 | Island | 7.72E-03 | 5.81E-06 |
| cg00497251 | RNPEPL1 | 5'UTR | S_Shore | -6.47E-03 | 2.75E-05 |
| cg00500989 | SMG5;TMEM79;TM | TSS1500;Body;TSS1 | NA | -1.55E-02 | 9.26E-06 |
| cg00503840 | DLX5 | Body | Island | 6.76E-03 | 3.18E-06 |
| cg00511674 | NA | NA | Island | 1.44E-02 | 1.46E-08 |
| cg00515457 | NA | NA | Island | 1.75E-02 | 1.16E-08 |
| cg00519069 | CD97;CD97;CD97 | TSS1500;TSS1500;T | NA | -1.19E-02 | 4.68E-08 |
| cg00521239 | PLEC1;PLEC1;PLEC1; | Body;Body;Body;Bo | N_Shore | -1.15E-02 | 2.47E-09 |
| cg00524486 | MUC5B | TSS200 | N_Shelf | -7.11E-03 | 6.21E-05 |
| cg00548708 | NA | NA | Island | 1.26E-02 | 8.22E-06 |
| cg00551679 | FOXF1 | 3'UTR | N_Shore | 1.49E-02 | 4.90E-09 |
| cg00552235 | NA | NA | NA | 2.66E-02 | 2.67E-13 |
| cg00552892 | SLC23A3;SLC23A3;S | TSS1500;TSS1500;T | NA | -1.55E-02 | 3.33E-08 |
| cg00553487 | GRIK5 | TSS1500 | NA | -1.23E-02 | 5.55E-13 |
| cg00557175 | KATNAL2 | Body | NA | -1.61E-02 | 2.88E-06 |
| cg00566516 | TSPAN33 | TSS1500 | N_Shore | -1.39E-02 | 7.06E-07 |
| cg00574412 | ABHD15 | Body | N_Shore | -6.72E-03 | 1.92E-07 |
| cg00574740 | LMO7 | Body | Island | 8.28E-03 | 3.51E-06 |
| cg00575672 | NA | NA | NA | -1.36E-02 | 1.42E-11 |

| | | | | | |
|---|---|---|---|---|---|
| cg00575674 | NA | NA | NA | -1.81E-02 | 3.97E-07 |
| cg00578032 | NA | NA | Island | 9.15E-03 | 1.64E-05 |
| cg00579520 | TMEM132C | Body | Island | 7.79E-03 | 1.49E-05 |
| cg00582628 | RGS20 | 1stExon | NA | -1.67E-02 | 6.56E-19 |
| cg00584238 | LOC153328 | Body | Island | 1.56E-02 | 4.27E-05 |
| cg00590036 | TMEM181 | TSS200 | Island | 8.05E-03 | 7.62E-06 |
| cg00590324 | TCF21;TCF21 | Body;Body | Island | 1.91E-02 | 3.48E-09 |
| cg00602811 | ZEB2;ZEB2;ZEB2 | TSS1500;TSS1500;T | N_Shelf | -1.00E-02 | 6.88E-06 |
| cg00609948 | NDFIP1 | TSS1500 | N_Shore | -7.68E-03 | 8.94E-06 |
| cg00612633 | NA | NA | N_Shore | -1.47E-02 | 6.83E-17 |
| cg00613752 | GPR6;GPR6 | 1stExon;5'UTR | Island | 1.14E-02 | 5.43E-10 |
| cg00620824 | HLA-C | TSS1500 | S_Shore | -2.26E-02 | 1.69E-08 |
| cg00621943 | LY6G5C | Body | N_Shelf | -1.06E-02 | 4.71E-07 |
| cg00622010 | AQP1 | TSS1500 | NA | -7.41E-03 | 5.39E-06 |
| cg00636527 | GRK6;GRK6;GRK6 | Body;Body;Body | N_Shore | -4.95E-03 | 8.59E-05 |
| cg00636737 | TCEA2;TCEA2 | Body;5'UTR | S_Shore | -8.88E-03 | 7.79E-07 |
| cg00637477 | DNAJC5 | TSS1500 | Island | 9.21E-03 | 2.98E-07 |
| cg00656728 | GUCA2B | TSS1500 | NA | -7.47E-03 | 6.97E-09 |
| cg00657460 | TCF4;TCF4 | Body;Body | NA | -7.81E-03 | 1.03E-05 |
| cg00661018 | KIAA1688 | Body | N_Shore | -7.26E-03 | 3.28E-06 |
| cg00661347 | COL13A1;COL13A1;( | Body;Body;Body;Bo | NA | -5.48E-03 | 8.03E-06 |
| cg00664416 | MANEAL;MANEAL;N | Body;Body;5'UTR;1: | S_Shore | 1.67E-02 | 1.08E-12 |
| cg00667751 | NA | NA | Island | 9.96E-03 | 4.74E-06 |
| cg00671600 | NA | NA | S_Shelf | -7.40E-03 | 8.39E-05 |
| cg00675878 | BCR;BCR | TSS1500;TSS1500 | N_Shore | -5.63E-03 | 5.36E-07 |
| cg00676833 | NA | NA | N_Shore | -5.71E-03 | 1.35E-06 |
| cg00677334 | NA | NA | NA | -1.78E-02 | 5.41E-15 |
| cg00683332 | NA | NA | Island | 1.57E-02 | 1.99E-05 |
| cg00684075 | NA | NA | NA | -7.18E-03 | 2.17E-08 |
| cg00687714 | CRYM;CRYM | Body;Body | NA | -2.05E-02 | 9.44E-09 |
| cg00688447 | NA | NA | Island | 2.25E-02 | 6.17E-07 |
| cg00688963 | THRB;THRB;THRB;TI | 1stExon;1stExon;5'U | Island | 2.86E-02 | 4.39E-07 |
| cg00689213 | REG1P | TSS1500 | NA | -1.18E-02 | 5.35E-05 |
| cg00690195 | NA | NA | NA | -7.20E-03 | 1.57E-06 |
| cg00695112 | TJP2;TJP2;TJP2;TJP2 | Body;Body;Body;Bo | N_Shore | -1.20E-02 | 6.96E-11 |
| cg00699993 | GRIA2;GRIA2;GRIA2 | TSS200;TSS1500;TS: | Island | 2.60E-02 | 6.40E-07 |
| cg00703513 | NA | NA | N_Shore | 6.81E-03 | 5.91E-06 |
| cg00708603 | NA | NA | NA | -9.69E-03 | 3.83E-09 |
| cg00714309 | RAB3B | Body | NA | -1.53E-02 | 2.70E-11 |
| cg00723017 | FER1L6 | 5'UTR | NA | -9.98E-03 | 1.77E-08 |
| cg00731185 | NA | NA | Island | 1.09E-02 | 6.17E-08 |
| cg00731683 | LTB;LTB | 3'UTR;3'UTR | Island | -7.41E-03 | 9.67E-05 |
| cg00731691 | LOC388428 | Body | NA | -1.25E-02 | 5.20E-12 |
| cg00733493 | NACAD | Body | N_Shore | -1.11E-02 | 4.99E-15 |
| cg00741624 | KIAA1409 | 5'UTR | Island | 7.19E-03 | 9.68E-07 |
| cg00745389 | NA | NA | Island | 1.30E-02 | 2.70E-11 |
| cg00748589 | NA | NA | Island | 1.13E-02 | 8.00E-13 |
| cg00753885 | NA | NA | S_Shore | -1.33E-02 | 2.02E-16 |
| cg00755588 | C9orf172 | TSS1500 | N_Shore | -1.04E-02 | 2.55E-15 |
| cg00764612 | C1orf51 | TSS200 | S_Shore | 1.21E-02 | 5.13E-12 |

| | | | | | |
|---|---|---|---|---|---|
| cg00774437 | NA | NA | NA | -1.15E-02 | 9.36E-10 |
| cg00776080 | TENC1;TENC1 | 5'UTR;TSS1500 | S_Shore | -1.39E-02 | 4.07E-10 |
| cg00779065 | NA | NA | S_Shelf | 1.05E-02 | 4.00E-06 |
| cg00783877 | NA | NA | NA | -2.12E-02 | 5.37E-06 |
| cg00791074 | MTHFD1L | TSS1500 | N_Shore | -1.67E-02 | 2.80E-07 |
| cg00792966 | SOX4 | 1stExon | Island | 1.34E-02 | 1.80E-06 |
| cg00804078 | DDO;DDO | TSS200;TSS200 | NA | -1.22E-02 | 7.42E-07 |
| cg00808170 | PCDHGA4;PCDHGA1 | Body;Body;Body;Bo | Island | 8.53E-03 | 9.39E-05 |
| cg00812438 | NA | NA | N_Shore | 1.44E-02 | 3.84E-05 |
| cg00812833 | PLD5 | 5'UTR | Island | 1.39E-02 | 7.08E-06 |
| cg00818680 | TMCO7 | Body | S_Shelf | -2.84E-02 | 2.15E-09 |
| cg00819338 | NA | NA | N_Shore | -6.67E-03 | 3.68E-06 |
| cg00824018 | INA | 1stExon | Island | 2.33E-02 | 1.51E-10 |
| cg00842161 | CHD8;CHD8;SNORD! | Body;Body;Body | NA | 9.10E-03 | 2.40E-05 |
| cg00842595 | C17orf97 | Body | Island | -2.04E-02 | 6.84E-19 |
| cg00848780 | GIPR | Body | Island | 4.34E-03 | 1.47E-06 |
| cg00851028 | NA | NA | NA | 5.89E-03 | 4.42E-05 |
| cg00855990 | PAX7;PAX7;PAX7 | Body;Body;Body | Island | 1.01E-02 | 1.32E-08 |
| cg00860372 | ZSWIM4 | Body | N_Shelf | -8.19E-03 | 1.06E-07 |
| cg00862117 | IQSEC3 | 1stExon | Island | 2.20E-02 | 1.52E-16 |
| cg00864666 | RAPGEF4;RAPGEF4 | Body;Body | NA | -1.79E-02 | 2.90E-26 |
| cg00872865 | NA | NA | NA | -1.01E-02 | 6.20E-06 |
| cg00874990 | NA | NA | Island | 8.93E-03 | 1.16E-06 |
| cg00875989 | NA | NA | Island | 9.11E-03 | 6.44E-11 |
| cg00876127 | BBS2 | TSS1500 | S_Shore | -1.57E-02 | 1.22E-12 |
| cg00876267 | NA | NA | N_Shore | -5.85E-03 | 8.96E-06 |
| cg00878605 | UNC13A | 3'UTR | Island | 9.76E-03 | 2.59E-08 |
| cg00884093 | CELSR1 | 1stExon | Island | 1.99E-02 | 7.65E-05 |
| cg00884334 | NA | NA | Island | 1.98E-02 | 1.16E-07 |
| cg00889398 | NA | NA | Island | 8.36E-03 | 6.61E-07 |
| cg00891995 | SPRR2C | Body | NA | -1.75E-02 | 6.11E-13 |
| cg00896068 | NA | NA | NA | -9.22E-03 | 6.40E-09 |
| cg00901765 | SPOCK3;SPOCK3 | 5'UTR;5'UTR | Island | 8.98E-03 | 7.93E-05 |
| cg00903099 | HTR5A | TSS200 | N_Shore | 9.28E-03 | 1.36E-05 |
| cg00909275 | ATXN2 | Body | N_Shore | 1.13E-02 | 2.81E-05 |
| cg00910067 | SLC7A10 | TSS1500 | Island | 8.48E-03 | 1.88E-05 |
| cg00911351 | PCDHGA4;PCDHGA6 | Body;Body;Body;Bo | Island | 9.27E-03 | 3.57E-05 |
| cg00923744 | GALNT2 | Body | S_Shore | -1.06E-02 | 4.83E-05 |
| cg00926285 | PARP14 | TSS1500 | N_Shore | -2.54E-02 | 5.42E-07 |
| cg00934037 | CDC14A;CDC14A;CD | Body;Body;Body | NA | 1.01E-02 | 4.26E-05 |
| cg00947744 | NCAPH2;LMF2;NCAI | TSS1500;Body;TSS1 | N_Shore | -8.99E-03 | 2.29E-12 |
| cg00949753 | NA | NA | N_Shore | 8.29E-03 | 4.45E-08 |
| cg00951599 | NA | NA | NA | -7.94E-03 | 1.56E-05 |
| cg00957665 | TRIM8 | Body | S_Shore | -5.41E-03 | 2.76E-05 |
| cg00957901 | LIMCH1;LIMCH1;LIN | TSS200;Body;Body;I | NA | -1.92E-02 | 5.96E-08 |
| cg00962755 | PHLDA3 | TSS1500 | S_Shore | 1.18E-02 | 2.96E-05 |
| cg00982136 | CLK1;CLK1;CLK1;CLK | Body;Body;Body;Bo | N_Shelf | -2.63E-02 | 3.98E-08 |
| cg00988548 | NA | NA | NA | -1.14E-02 | 1.95E-07 |
| cg00989492 | NA | NA | Island | 2.61E-02 | 5.03E-05 |
| cg00989853 | IRF6;IRF6 | 5'UTR;1stExon | Island | 2.87E-02 | 1.26E-05 |

| | | | | | |
|---|---|---|---|---|---|
| cg00994250 | PLEKHG1 | Body | NA | -1.74E-02 | 1.22E-09 |
| cg00995854 | CD5L | Body | NA | -8.34E-03 | 1.31E-05 |
| cg01001286 | NA | NA | NA | -1.02E-02 | 5.34E-05 |
| cg01002529 | NA | NA | Island | 8.26E-03 | 5.46E-07 |
| cg01002636 | RBM11 | TSS1500 | N_Shore | -1.59E-02 | 3.64E-06 |
| cg01007828 | CDH8 | TSS1500 | Island | 1.04E-02 | 2.13E-11 |
| cg01014224 | NA | NA | NA | -2.03E-02 | 2.32E-12 |
| cg01022345 | MIR770;MEG3;MEG | TSS1500;Body;Body | NA | -8.00E-03 | 1.51E-09 |
| cg01023751 | LRFN3 | TSS1500 | N_Shore | -1.29E-02 | 1.52E-06 |
| cg01026661 | FAM173B | Body | NA | -4.82E-02 | 5.06E-06 |
| cg01028796 | BICD2;BICD2 | Body;Body | N_Shore | -1.98E-02 | 8.26E-17 |
| cg01036351 | LOC200726 | TSS1500 | N_Shore | 1.00E-02 | 3.83E-10 |
| cg01040850 | MR1 | TSS1500 | NA | -1.56E-02 | 2.27E-19 |
| cg01041405 | SMPD3 | 5'UTR | NA | -2.54E-02 | 7.88E-05 |
| cg01054110 | NCOR2;NCOR2 | 5'UTR;5'UTR | Island | -2.84E-02 | 4.42E-15 |
| cg01055824 | CCDC88C | Body | NA | -1.51E-02 | 1.67E-05 |
| cg01062126 | SNRNP25;POLR3K | Body;TSS1500 | S_Shore | -1.15E-02 | 3.77E-07 |
| cg01066157 | SYNE1;SYNE1;SYNE1 | Body;Body;Body;Bo | NA | -1.57E-02 | 4.29E-06 |
| cg01075271 | NA | NA | Island | 1.78E-02 | 2.67E-09 |
| cg01077058 | NA | NA | NA | -2.34E-02 | 1.18E-12 |
| cg01078824 | HOXA10 | TSS200 | S_Shore | 6.03E-03 | 4.07E-05 |
| cg01081083 | SGSM1;SGSM1;SGSI | Body;Body;Body;Bo | S_Shore | -1.52E-02 | 1.99E-11 |
| cg01097384 | NA | NA | Island | 1.45E-02 | 6.87E-05 |
| cg01100175 | KIF5C | Body | S_Shore | 1.95E-02 | 6.14E-07 |
| cg01101873 | PRDM16;PRDM16 | Body;Body | NA | -1.20E-02 | 1.68E-11 |
| cg01102833 | WDR17;WDR17 | TSS1500;TSS1500 | N_Shore | -1.57E-02 | 1.50E-12 |
| cg01102854 | MARK2;MARK2;MAI | TSS1500;TSS1500;T | N_Shore | -8.08E-03 | 1.77E-11 |
| cg01103207 | TNS3 | 5'UTR | NA | -6.40E-03 | 1.27E-06 |
| cg01103827 | PSMG1;PSMG1 | Body;Body | N_Shore | -2.59E-02 | 1.78E-16 |
| cg01104208 | C14orf73 | TSS1500 | N_Shelf | -7.46E-03 | 3.34E-07 |
| cg01106338 | NA | NA | Island | 6.64E-03 | 5.86E-07 |
| cg01106926 | NA | NA | S_Shore | 9.92E-03 | 1.27E-10 |
| cg01108112 | NA | NA | N_Shore | -4.59E-03 | 8.95E-05 |
| cg01109279 | KBTBD11 | TSS1500 | N_Shore | -1.79E-02 | 4.99E-07 |
| cg01111041 | PPT2;PRRT1;PPT2 | TSS1500;TSS1500;T | N_Shore | -7.69E-03 | 7.90E-05 |
| cg01127300 | NA | NA | S_Shelf | -8.73E-03 | 1.35E-05 |
| cg01128109 | LRRC8B | TSS1500 | N_Shore | -1.51E-02 | 1.64E-17 |
| cg01133433 | NA | NA | N_Shore | 1.40E-02 | 2.96E-05 |
| cg01137047 | SERPINB6 | 5'UTR | N_Shore | -6.90E-03 | 6.54E-07 |
| cg01139880 | TELO2 | Body | S_Shore | -1.29E-02 | 3.40E-06 |
| cg01141413 | NA | NA | N_Shore | -6.34E-03 | 4.40E-06 |
| cg01141438 | LEPREL1;LEPREL1 | 5'UTR;Body | N_Shore | -8.16E-03 | 2.45E-07 |
| cg01142635 | NA | NA | Island | 1.59E-02 | 5.29E-07 |
| cg01142676 | FXYD2;FXYD2;FXYD2 | Body;TSS200;TSS20 | NA | -9.65E-03 | 1.63E-05 |
| cg01148073 | HSD17B2 | Body | NA | -1.04E-02 | 1.88E-08 |
| cg01150683 | NA | NA | Island | 1.01E-02 | 3.75E-06 |
| cg01153166 | NR2F2;NR2F2;NR2F | TSS1500;1stExon;B | Island | 1.12E-02 | 3.68E-09 |
| cg01153385 | NA | NA | NA | -8.66E-03 | 6.35E-09 |
| cg01155497 | PCDHA2;PCDHA1;P | Body;Body;Body;Bo | Island | 8.07E-03 | 1.73E-06 |
| cg01184975 | NA | NA | Island | 1.58E-02 | 6.55E-05 |

| | | | | | |
|---|---|---|---|---|---|
| cg01185921 | ABLIM1;ABLIM1 | TSS200;TSS200 | NA | -1.22E-02 | 4.62E-15 |
| cg01194538 | CYB5R3;CYB5R3;CYE | TSS1500;Body;TSS1 | N_Shore | -7.04E-03 | 3.21E-05 |
| cg01205267 | LRCH1;LRCH1;LRCH1 | TSS1500;TSS1500;T | N_Shore | -1.87E-02 | 1.55E-08 |
| cg01207684 | ADCY9 | Body | NA | -2.22E-02 | 1.83E-09 |
| cg01219000 | CXCL5 | TSS1500 | S_Shore | -8.20E-03 | 4.64E-06 |
| cg01222316 | HRNBP3 | 5'UTR | NA | -1.28E-02 | 7.85E-06 |
| cg01234420 | LOC150381 | Body | N_Shelf | -1.44E-02 | 2.25E-15 |
| cg01236063 | NA | NA | N_Shore | 1.24E-02 | 5.68E-08 |
| cg01239651 | PIK3R1;PIK3R1 | Body;TSS1500 | N_Shore | -1.22E-02 | 5.84E-09 |
| cg01243823 | NOD2 | Body | NA | -9.78E-03 | 2.38E-07 |
| cg01256539 | PRR16 | 5'UTR | S_Shore | -2.48E-02 | 1.87E-11 |
| cg01259782 | CRYM;NCRNA00169 | 5'UTR;Body | S_Shore | -2.30E-02 | 5.38E-08 |
| cg01261798 | DPYSL4 | TSS1500 | Island | 3.23E-02 | 3.22E-06 |
| cg01268590 | NA | NA | Island | 1.80E-02 | 2.47E-09 |
| cg01276475 | RIMS1 | TSS1500 | Island | 1.03E-02 | 3.22E-08 |
| cg01276536 | BAIAP2;BAIAP2;BAIA | Body;Body;Body;Bo | N_Shore | -8.42E-03 | 1.94E-07 |
| cg01278654 | C17orf73 | TSS1500 | NA | -1.11E-02 | 1.08E-09 |
| cg01281911 | FOXE3 | 1stExon | Island | 2.28E-02 | 4.23E-11 |
| cg01282174 | NA | NA | NA | -1.76E-02 | 2.45E-11 |
| cg01283246 | FBXL21;FBXL21 | 1stExon;5'UTR | Island | 3.74E-02 | 1.77E-06 |
| cg01287975 | TAC1;TAC1;TAC1;TA | TSS200;TSS200;TSS2 | Island | 1.58E-02 | 4.90E-10 |
| cg01290904 | EVC2;EVC2 | 5'UTR;Body | N_Shore | -9.88E-03 | 4.01E-08 |
| cg01293740 | CACNG8 | Body | S_Shelf | -1.01E-02 | 2.93E-07 |
| cg01294808 | IRX1 | Body | Island | 1.21E-02 | 1.60E-06 |
| cg01296360 | NA | NA | Island | 7.01E-03 | 1.45E-08 |
| cg01297756 | CLYBL | 3'UTR | Island | 8.87E-03 | 2.99E-09 |
| cg01302656 | C14orf23 | Body | Island | 7.65E-03 | 7.54E-05 |
| cg01314044 | NA | NA | S_Shore | -1.01E-02 | 4.05E-07 |
| cg01314574 | COL18A1 | Body | N_Shore | -1.04E-02 | 6.09E-10 |
| cg01329789 | TMBIM1;PNKD | TSS1500;Body | S_Shore | -1.51E-02 | 1.42E-10 |
| cg01337940 | FAT1 | Body | NA | -3.23E-02 | 5.18E-06 |
| cg01344420 | NA | NA | NA | -2.52E-02 | 9.59E-06 |
| cg01344797 | PSD | Body | S_Shelf | -6.83E-03 | 2.52E-05 |
| cg01346718 | CSNK1E;CSNK1E | 5'UTR;TSS1500 | S_Shore | -6.09E-03 | 9.20E-05 |
| cg01350680 | NA | NA | S_Shore | 8.87E-03 | 2.31E-06 |
| cg01352586 | NA | NA | Island | 1.13E-02 | 3.92E-10 |
| cg01360605 | NA | NA | NA | -1.03E-02 | 5.75E-06 |
| cg01368219 | CACNA2D3 | Body | NA | -1.26E-02 | 1.22E-12 |
| cg01372572 | ATP10A | Body | NA | -9.35E-03 | 1.53E-09 |
| cg01373819 | NA | NA | S_Shore | -1.05E-02 | 2.81E-05 |
| cg01376402 | NA | NA | Island | 7.42E-03 | 1.25E-05 |
| cg01376829 | IL1RL2 | Body | Island | 9.74E-03 | 5.55E-05 |
| cg01382110 | NA | NA | Island | 1.12E-02 | 3.74E-08 |
| cg01391297 | LOC645676;ASH1L | TSS1500;5'UTR | N_Shore | -7.81E-03 | 6.11E-05 |
| cg01394339 | KHDC1 | 5'UTR | N_Shore | -1.39E-02 | 2.00E-05 |
| cg01397325 | HTATIP2;HTATIP2;H | Body;Body;Body;Bo | S_Shore | -7.10E-03 | 1.91E-05 |
| cg01400822 | GPX7 | TSS1500 | N_Shore | -9.50E-03 | 2.28E-08 |
| cg01405404 | NA | NA | Island | 1.65E-02 | 2.97E-10 |
| cg01405582 | HLA-F;HLA-F;HLA-F | Body;Body;Body | S_Shore | -1.04E-02 | 5.50E-06 |
| cg01405761 | CLVS1;CLVS1 | 1stExon;5'UTR | NA | 1.20E-02 | 3.36E-10 |

| | | | | | |
|---|---|---|---|---|---|
| cg01407018 | NA | NA | NA | -2.34E-02 | 5.97E-05 |
| cg01407062 | PRC1;PRC1;PRC1 | TSS1500;TSS1500;T | S_Shore | -3.33E-02 | 4.48E-06 |
| cg01409985 | SRPRB | TSS1500 | N_Shore | -1.91E-02 | 5.24E-14 |
| cg01425188 | NA | NA | N_Shore | -3.11E-02 | 6.80E-06 |
| cg01429408 | NA | NA | N_Shelf | -1.35E-02 | 1.29E-12 |
| cg01437204 | PTPN7;PTPN7 | TSS1500;TSS1500 | NA | -1.12E-02 | 5.68E-08 |
| cg01442601 | NA | NA | Island | 9.08E-03 | 1.17E-06 |
| cg01444397 | PER3 | TSS1500 | Island | 3.38E-02 | 2.78E-08 |
| cg01449136 | NA | NA | Island | 8.28E-03 | 2.49E-08 |
| cg01458054 | PRIC285;PRIC285 | Body;TSS1500 | N_Shore | -9.43E-03 | 2.02E-06 |
| cg01461824 | NA | NA | NA | 7.10E-03 | 5.54E-05 |
| cg01464985 | ZNF512 | TSS1500 | N_Shore | -9.33E-03 | 1.01E-06 |
| cg01474257 | PYY | Body | Island | 6.56E-03 | 3.15E-07 |
| cg01475538 | RRAS2;RRAS2 | TSS1500;TSS1500 | S_Shore | -5.74E-03 | 1.23E-05 |
| cg01478482 | DNAH3;TMEM159 | Body;TSS1500 | N_Shore | -1.74E-02 | 4.59E-05 |
| cg01485797 | SNORA52;RPLP2 | TSS1500;Body | S_Shore | -1.23E-02 | 1.76E-06 |
| cg01486146 | NA | NA | Island | 1.41E-02 | 3.66E-13 |
| cg01486610 | RNF170;RNF170;RN | 5'UTR;Body;5'UTR;5 | N_Shelf | -4.69E-02 | 2.90E-11 |
| cg01489686 | FUT9 | TSS200 | N_Shore | 6.82E-03 | 1.56E-05 |
| cg01493685 | DOCK4 | Body | NA | -1.62E-02 | 2.90E-09 |
| cg01503065 | DCHS2;DCHS2 | 1stExon;1stExon | Island | 9.24E-03 | 1.03E-06 |
| cg01515534 | NA | NA | N_Shore | -8.95E-03 | 6.89E-09 |
| cg01515543 | LOC100129066 | TSS200 | NA | -8.69E-03 | 8.15E-07 |
| cg01517384 | HLA-B | Body | N_Shore | -1.59E-02 | 5.29E-06 |
| cg01521274 | NA | NA | NA | -1.89E-02 | 4.30E-20 |
| cg01527307 | NA | NA | NA | -1.14E-02 | 1.79E-05 |
| cg01528542 | NA | NA | N_Shelf | -2.08E-02 | 7.35E-11 |
| cg01542019 | TECR | Body | N_Shelf | -2.06E-02 | 2.85E-10 |
| cg01543603 | ANKRD11 | 5'UTR | NA | 8.89E-03 | 4.09E-05 |
| cg01546568 | CTNNA2;CTNNA2;CT | 1stExon;1stExon;5'U | S_Shore | 2.06E-02 | 7.41E-06 |
| cg01552919 | GAK | Body | S_Shore | -1.65E-02 | 1.32E-11 |
| cg01554963 | NXN | Body | NA | 8.94E-03 | 7.20E-05 |
| cg01560676 | ERGIC1 | TSS1500 | N_Shore | -9.39E-03 | 1.43E-07 |
| cg01560871 | C10orf27 | TSS1500 | NA | -9.51E-03 | 1.20E-08 |
| cg01564343 | TREML1 | Body | NA | -9.07E-03 | 2.54E-07 |
| cg01565703 | TRAF3;TRAF3;TRAF3 | 5'UTR;5'UTR;5'UTR | NA | -1.77E-02 | 9.61E-15 |
| cg01567672 | MXD4 | Body | N_Shore | -5.31E-03 | 8.69E-05 |
| cg01577760 | ALDH3B1;ALDH3B1 | 1stExon;5'UTR | NA | -1.11E-02 | 9.50E-05 |
| cg01581018 | ZIC4;ZIC4;ZIC4;ZIC4; | Body;TSS1500;Body | Island | 1.19E-02 | 5.57E-08 |
| cg01586952 | NA | NA | N_Shore | -7.30E-03 | 6.71E-09 |
| cg01588224 | JTB | TSS1500 | S_Shore | -1.99E-02 | 1.08E-22 |
| cg01591830 | NA | NA | S_Shelf | -1.08E-02 | 6.45E-09 |
| cg01592801 | KCNS2 | TSS1500 | Island | 8.67E-03 | 2.69E-05 |
| cg01593969 | MYH6 | 3'UTR | NA | -8.34E-03 | 3.50E-11 |
| cg01594949 | NRARP | TSS1500 | S_Shore | -9.36E-03 | 9.59E-10 |
| cg01598596 | BCL6 | TSS1500 | S_Shelf | -2.12E-02 | 6.62E-08 |
| cg01605783 | LOC284837 | TSS200 | NA | -7.07E-03 | 1.79E-06 |
| cg01620309 | ADAM11 | Body | N_Shore | -1.02E-02 | 4.16E-07 |
| cg01622018 | MMP28;MMP28 | TSS1500;TSS1500 | S_Shore | -7.11E-03 | 6.59E-06 |
| cg01622416 | GRIK1;GRIK1 | Body;Body | N_Shore | -1.82E-02 | 5.53E-12 |

| | | | | | |
|---|---|---|---|---|---|
| cg01622930 | NA | NA | S_Shore | -1.26E-02 | 2.08E-18 |
| cg01625087 | NA | NA | N_Shore | 1.74E-02 | 4.80E-11 |
| cg01627252 | CAPN14 | 3'UTR | NA | -7.23E-03 | 1.18E-08 |
| cg01644611 | OXT | TSS200 | Island | -1.67E-02 | 2.78E-06 |
| cg01657995 | C6orf48;C6orf48;SN | 5'UTR;5'UTR;Body | S_Shelf | -7.27E-03 | 7.59E-05 |
| cg01663970 | SCARB1;SCARB1 | Body;Body | Island | 7.61E-03 | 5.19E-05 |
| cg01666793 | CACNG8;MIR935 | Body;TSS200 | Island | 8.85E-03 | 1.12E-05 |
| cg01671681 | PLCH1 | 5'UTR | NA | -2.06E-02 | 1.88E-24 |
| cg01672322 | FOSL1 | 3'UTR | S_Shore | -6.76E-03 | 6.50E-06 |
| cg01676322 | ACBD4;ACBD4;ACBD | 5'UTR;5'UTR;5'UTR; | S_Shelf | -6.50E-03 | 1.93E-05 |
| cg01686975 | NA | NA | N_Shelf | -7.99E-03 | 1.28E-05 |
| cg01687680 | CBLN1 | 3'UTR | Island | 1.40E-02 | 7.15E-07 |
| cg01688536 | NA | NA | Island | 1.94E-02 | 1.83E-06 |
| cg01688688 | PCDHB18 | Body | N_Shore | 8.72E-03 | 1.12E-05 |
| cg01713950 | RCC2 | TSS1500 | S_Shore | -1.32E-02 | 9.68E-11 |
| cg01720722 | CDK18;CDK18;CDK1 | 5'UTR;5'UTR;5'UTR | NA | -7.72E-03 | 7.15E-05 |
| cg01722932 | MTNR1B | TSS200 | N_Shore | 6.10E-03 | 5.68E-08 |
| cg01729827 | CBLN4 | 1stExon | Island | 2.15E-02 | 4.02E-06 |
| cg01747664 | TRAPPC3 | Body | N_Shore | -1.80E-02 | 1.63E-11 |
| cg01749249 | TTBK1 | Body | N_Shore | -8.41E-03 | 2.50E-07 |
| cg01750611 | TRAF4 | Body | S_Shore | -7.00E-03 | 3.00E-08 |
| cg01753375 | DCTN1;DCTN1 | TSS200;TSS200 | NA | -6.65E-03 | 1.48E-06 |
| cg01759562 | NA | NA | NA | 9.78E-03 | 1.94E-05 |
| cg01763090 | OTUD7A | 3'UTR | N_Shore | 1.27E-02 | 5.28E-12 |
| cg01770755 | NA | NA | Island | 8.88E-03 | 9.41E-06 |
| cg01777643 | CNIH3 | Body | Island | 2.82E-02 | 5.94E-06 |
| cg01783650 | PRDM16;PRDM16 | Body;Body | Island | -5.74E-03 | 2.43E-05 |
| cg01804934 | HLA-DPA1 | Body | NA | -1.51E-02 | 5.92E-10 |
| cg01805215 | FBXL17 | Body | NA | -1.35E-02 | 9.13E-15 |
| cg01806921 | NA | NA | S_Shore | -6.22E-03 | 2.60E-06 |
| cg01812045 | DES | 1stExon | Island | 8.63E-03 | 7.53E-12 |
| cg01819137 | POU4F3 | Body | Island | 9.04E-03 | 1.30E-06 |
| cg01820374 | LAG3 | Body | N_Shore | -1.14E-02 | 2.35E-14 |
| cg01823925 | ADAM19 | Body | NA | -5.94E-03 | 3.67E-06 |
| cg01829241 | GPR78 | TSS200 | Island | 1.98E-02 | 6.87E-07 |
| cg01835021 | MSH5;MSH5;C6orf2 | Body;Body;TSS1500 | NA | -1.08E-02 | 2.02E-08 |
| cg01835725 | PCBP4 | TSS1500 | NA | 6.19E-03 | 5.50E-10 |
| cg01839396 | NA | NA | Island | 8.20E-03 | 2.17E-05 |
| cg01844642 | GPR62 | 1stExon | Island | 1.07E-02 | 1.11E-14 |
| cg01847889 | NA | NA | S_Shelf | -7.25E-03 | 2.02E-05 |
| cg01850269 | SIM1 | Body | N_Shore | 9.96E-03 | 1.16E-08 |
| cg01851378 | NA | NA | Island | 1.27E-02 | 4.16E-08 |
| cg01851672 | PPAP2A;PPAP2A;RN | Body;Body;Body | N_Shore | -1.25E-02 | 6.36E-10 |
| cg01851782 | NA | NA | S_Shore | -1.01E-02 | 2.92E-08 |
| cg01875106 | OPCML;OPCML | 3'UTR;3'UTR | NA | -1.47E-02 | 1.63E-10 |
| cg01881062 | KLHL21 | Body | N_Shore | -1.23E-02 | 7.68E-11 |
| cg01885635 | ZNF621;ZNF621 | TSS1500;TSS1500 | N_Shore | -8.23E-03 | 2.91E-08 |
| cg01886514 | NA | NA | N_Shore | 8.74E-03 | 4.48E-07 |
| cg01894846 | NA | NA | S_Shore | -9.14E-03 | 3.34E-10 |
| cg01897498 | ANKRD19 | Body | Island | 8.02E-03 | 1.50E-07 |

| | | | | | |
|---|---|---|---|---|---|
| cg01899253 | FLT1;FLT1;FLT1;FLT1 | TSS1500;TSS1500;T | S_Shore | -1.13E-02 | 2.40E-05 |
| cg01910639 | S100A6 | Body | N_Shore | -1.13E-02 | 1.11E-17 |
| cg01912921 | FOXC1 | 1stExon | Island | 2.05E-02 | 2.17E-05 |
| cg01921432 | BARHL2 | 1stExon | Island | 1.54E-02 | 1.69E-08 |
| cg01936370 | NA | NA | Island | 8.43E-03 | 5.92E-05 |
| cg01937055 | NA | NA | Island | 1.29E-02 | 4.48E-11 |
| cg01937212 | C6orf10 | Body | NA | -1.44E-02 | 1.02E-07 |
| cg01937780 | NA | NA | S_Shore | 1.45E-02 | 2.27E-09 |
| cg01940943 | C17orf71 | 1stExon | Island | 9.82E-03 | 2.07E-05 |
| cg01942816 | MIR589;FBXL18 | Body;Body | S_Shore | -1.36E-02 | 1.14E-11 |
| cg01942962 | GRIA2;GRIA2;GRIA2 | Body;Body;Body | Island | 1.67E-02 | 6.40E-07 |
| cg01957582 | C22orf24;YWHAH | TSS1500;Body | S_Shore | -9.66E-03 | 4.28E-07 |
| cg01959123 | NA | NA | N_Shore | -1.64E-02 | 8.58E-09 |
| cg01959730 | NA | NA | N_Shore | 1.60E-02 | 3.01E-06 |
| cg01960096 | ARHGEF11;ARHGEF1 | Body;Body | N_Shelf | -1.55E-02 | 3.05E-11 |
| cg01963933 | TEKT5 | Body | S_Shore | -1.19E-02 | 3.41E-10 |
| cg01970325 | NELF;NELF;NELF;NEI | TSS1500;TSS1500;T | S_Shore | -6.61E-03 | 1.42E-07 |
| cg01974375 | PI4KB | TSS1500 | N_Shore | -1.24E-02 | 2.81E-10 |
| cg01981760 | FTO;RPGRIP1L;RPGF | TSS1500;5'UTR;5'U | N_Shore | -7.64E-03 | 1.14E-05 |
| cg01983373 | DLK1 | Body | S_Shelf | -7.89E-03 | 3.22E-09 |
| cg01990878 | TEKT1 | TSS200 | NA | 1.19E-02 | 1.54E-05 |
| cg01997272 | NA | NA | Island | 2.24E-02 | 4.22E-07 |
| cg02005231 | DNAH3 | Body | NA | -9.96E-03 | 8.54E-06 |
| cg02010481 | JAZF1 | Body | N_Shore | -1.12E-02 | 1.47E-05 |
| cg02018902 | ANKRD34C | 5'UTR | Island | 1.53E-02 | 3.56E-13 |
| cg02030542 | VAC14 | Body | S_Shelf | -4.30E-03 | 5.15E-06 |
| cg02042310 | NA | NA | Island | 6.96E-03 | 1.28E-08 |
| cg02046143 | IGSF9B | Body | N_Shelf | -1.70E-02 | 5.14E-11 |
| cg02047661 | RRP9;PARP3;PARP3 | TSS1500;Body;5'UT | S_Shore | -8.34E-03 | 6.67E-07 |
| cg02053807 | C20orf166;C20orf20 | TSS1500;5'UTR | N_Shore | -5.88E-03 | 5.61E-05 |
| cg02064368 | NA | NA | NA | -1.57E-02 | 4.36E-05 |
| cg02067712 | NA | NA | N_Shore | 1.20E-02 | 3.17E-05 |
| cg02077766 | PTCRA | Body | N_Shore | -8.24E-03 | 1.03E-06 |
| cg02078690 | BSX | TSS200 | Island | 9.92E-03 | 9.91E-05 |
| cg02079111 | TGFBRAP1;TGFBRAF | Body;Body | Island | -1.16E-02 | 4.08E-05 |
| cg02079584 | CCDC33;CCDC33 | 1stExon;5'UTR | NA | -7.32E-03 | 1.27E-07 |
| cg02081052 | NA | NA | Island | 1.91E-02 | 2.46E-08 |
| cg02085953 | ARID5A | TSS1500 | N_Shore | -9.34E-03 | 4.27E-17 |
| cg02088996 | LOC285954 | Body | NA | -9.34E-03 | 2.17E-08 |
| cg02091100 | GUCA1A | TSS200 | NA | -1.04E-02 | 8.64E-10 |
| cg02097429 | NA | NA | N_Shelf | -1.61E-02 | 5.85E-13 |
| cg02106941 | NEFM;NEFM | TSS1500;1stExon | Island | 1.23E-02 | 5.60E-05 |
| cg02107824 | VWA2 | TSS1500 | N_Shore | -1.13E-02 | 5.09E-07 |
| cg02109699 | ADAMTS16 | Body | NA | -1.50E-02 | 9.09E-12 |
| cg02110858 | PLEC1;PLEC1;PLEC1 | Body;Body;TSS200 | Island | -1.41E-02 | 7.87E-06 |
| cg02119348 | A2BP1;A2BP1;A2BP | 5'UTR;1stExon;1stE | Island | 7.61E-03 | 8.13E-06 |
| cg02121547 | CLDN25 | TSS1500 | NA | -2.24E-02 | 3.32E-08 |
| cg02124499 | NA | NA | N_Shore | -7.28E-03 | 2.92E-08 |
| cg02130289 | TBCD | Body | NA | -9.97E-03 | 2.25E-06 |
| cg02146001 | GATA5 | TSS1500 | Island | 2.26E-02 | 1.16E-08 |

| | | | | | |
|---|---|---|---|---|---|
| cg02152233 | OPRD1 | Body | Island | 2.81E-02 | 4.91E-06 |
| cg02154186 | PNMA2;PNMA2 | 5'UTR;1stExon | Island | 1.17E-02 | 1.20E-11 |
| cg02156692 | AMZ1 | Body | NA | -1.71E-02 | 1.21E-13 |
| cg02164046 | SST;SST | 1stExon;5'UTR | Island | 2.45E-02 | 5.63E-13 |
| cg02170478 | PDGFRA | 5'UTR | S_Shore | -1.45E-02 | 4.53E-14 |
| cg02172150 | WBSCR17 | TSS200 | Island | 1.04E-02 | 9.09E-05 |
| cg02172819 | KCNMA1;KCNMA1;K | Body;Body;Body;Bo | NA | -9.15E-03 | 4.63E-10 |
| cg02173067 | NA | NA | NA | 3.10E-02 | 1.50E-07 |
| cg02187214 | FOXC2 | TSS1500 | Island | 1.39E-02 | 5.60E-06 |
| cg02188818 | TBC1D9 | Body | N_Shore | -1.62E-02 | 2.78E-12 |
| cg02189888 | NA | NA | Island | 1.05E-02 | 4.07E-09 |
| cg02203380 | C7orf41 | TSS1500 | N_Shore | -1.10E-02 | 3.58E-05 |
| cg02203881 | PLA2G4D | TSS200 | NA | -6.14E-03 | 4.82E-05 |
| cg02204637 | NA | NA | N_Shore | -3.18E-02 | 2.04E-16 |
| cg02210887 | GUCY1A3;GUCY1A3 | TSS1500;TSS1500;T | N_Shore | -1.37E-02 | 1.28E-06 |
| cg02210934 | NA | NA | N_Shore | -1.27E-02 | 1.62E-06 |
| cg02215357 | HNRNPA1L2;HNRNP | TSS1500;TSS1500 | N_Shore | -1.15E-02 | 3.04E-05 |
| cg02224314 | BCL11B;BCL11B | Body;Body | Island | 4.29E-02 | 7.97E-05 |
| cg02228185 | ASPA;ASPA | 1stExon;Body | NA | -3.16E-02 | 9.99E-09 |
| cg02229135 | MIR548F5;NBEA | Body;Body | Island | 1.08E-02 | 3.13E-06 |
| cg02239862 | SUSD5 | TSS200 | Island | 8.53E-03 | 6.47E-06 |
| cg02245004 | NA | NA | Island | 2.09E-02 | 2.96E-08 |
| cg02250594 | ONECUT2 | 1stExon | Island | 1.11E-02 | 3.46E-06 |
| cg02254885 | NA | NA | N_Shore | -7.19E-03 | 1.78E-07 |
| cg02255054 | NA | NA | Island | 8.81E-03 | 4.10E-07 |
| cg02256105 | NA | NA | N_Shore | -8.22E-03 | 3.37E-07 |
| cg02258201 | HRCT1 | TSS200 | NA | -9.20E-03 | 6.10E-07 |
| cg02272968 | TNXB | Body | NA | -1.06E-02 | 4.79E-07 |
| cg02272993 | NA | NA | Island | 8.46E-03 | 9.48E-05 |
| cg02275784 | DLG2 | Body | NA | -1.73E-02 | 9.54E-07 |
| cg02276274 | LRRN1 | TSS1500 | N_Shore | -6.59E-03 | 7.86E-06 |
| cg02281167 | TRIM15 | Body | Island | 6.25E-03 | 1.70E-05 |
| cg02281208 | GABRG3 | TSS1500 | Island | 1.66E-02 | 1.60E-07 |
| cg02286081 | HLA-DPB1 | 1stExon | NA | -1.74E-02 | 4.53E-09 |
| cg02286091 | BHMT;BHMT | 5'UTR;1stExon | NA | 1.93E-02 | 4.48E-08 |
| cg02291439 | PPIC | Body | N_Shore | -6.52E-03 | 3.82E-06 |
| cg02294055 | LOC100287216;SH3 | Body;1stExon | Island | 6.62E-03 | 1.44E-05 |
| cg02294302 | FOXD2;FOXD2 | 3'UTR;1stExon | S_Shore | -4.94E-03 | 1.00E-05 |
| cg02297967 | NA | NA | NA | -2.62E-02 | 7.24E-15 |
| cg02301528 | APOLD1;APOLD1 | Body;Body | N_Shore | -1.13E-02 | 9.05E-12 |
| cg02301740 | NA | NA | N_Shelf | -8.82E-03 | 4.73E-06 |
| cg02303505 | NA | NA | NA | -2.01E-02 | 1.06E-11 |
| cg02303677 | TACR2 | TSS1500 | NA | -1.19E-02 | 1.80E-06 |
| cg02305765 | NA | NA | N_Shore | 1.97E-02 | 5.22E-05 |
| cg02306162 | NA | NA | S_Shelf | -2.05E-02 | 2.00E-14 |
| cg02308706 | C17orf91;C17orf91; | Body;Body;Body;Bo | N_Shelf | -8.43E-03 | 1.05E-06 |
| cg02315732 | FTH1 | Body | N_Shelf | -1.97E-02 | 1.41E-17 |
| cg02318784 | NA | NA | Island | 9.79E-03 | 1.35E-08 |
| cg02318971 | NA | NA | Island | 9.59E-03 | 1.08E-09 |
| cg02323356 | SPEG | Body | Island | 1.08E-02 | 9.31E-11 |

| | | | | | |
|---|---|---|---|---|---|
| cg02332073 | TSGA13 | TSS1500 | NA | -8.52E-03 | 4.52E-06 |
| cg02334775 | ISG20 | Body | NA | -8.37E-03 | 4.54E-07 |
| cg02336521 | NA | NA | Island | 9.48E-03 | 2.28E-07 |
| cg02336827 | LIMD1 | TSS200 | S_Shore | -8.22E-03 | 3.13E-10 |
| cg02344113 | NA | NA | N_Shore | -1.43E-02 | 1.29E-18 |
| cg02346706 | NA | NA | NA | -8.26E-03 | 1.58E-08 |
| cg02352240 | NA | NA | Island | 9.83E-03 | 1.79E-07 |
| cg02352281 | NA | NA | NA | -1.58E-02 | 1.15E-08 |
| cg02356551 | NA | NA | NA | 2.25E-02 | 1.06E-06 |
| cg02360199 | NA | NA | Island | 9.90E-03 | 1.30E-09 |
| cg02362292 | DAGLA | Body | S_Shore | -7.30E-03 | 3.48E-09 |
| cg02383785 | NA | NA | Island | 1.31E-02 | 8.19E-17 |
| cg02387435 | NA | NA | N_Shore | -8.63E-03 | 9.26E-05 |
| cg02390209 | HS3ST4 | TSS1500 | Island | 8.63E-03 | 2.87E-08 |
| cg02393721 | C12orf39 | Body | N_Shore | -1.14E-02 | 9.01E-05 |
| cg02396116 | NA | NA | NA | -1.03E-02 | 8.63E-09 |
| cg02401556 | NA | NA | N_Shore | 7.64E-03 | 9.21E-05 |
| cg02402091 | ACSL5;ACSL5;ACSL5 | TSS1500;1stExon;5' | NA | -1.31E-02 | 1.26E-16 |
| cg02409351 | ALX1 | TSS200 | Island | 9.49E-03 | 2.73E-06 |
| cg02412616 | NA | NA | N_Shore | -9.10E-03 | 2.65E-06 |
| cg02423574 | CCDC153;CCDC153 | 1stExon;5'UTR | NA | -7.57E-03 | 7.05E-05 |
| cg02431562 | DUSP2 | Body | N_Shore | -6.97E-03 | 1.29E-05 |
| cg02446106 | ZNF805;ZNF805 | TSS1500;TSS1500 | N_Shore | 1.69E-02 | 8.03E-05 |
| cg02446869 | NA | NA | S_Shelf | -1.31E-02 | 1.52E-09 |
| cg02451443 | NA | NA | Island | -1.23E-02 | 1.07E-06 |
| cg02452732 | IFI35 | TSS200 | NA | -1.28E-02 | 1.48E-06 |
| cg02471940 | NA | NA | NA | -4.89E-03 | 1.43E-10 |
| cg02478589 | NA | NA | S_Shore | -1.04E-02 | 2.33E-08 |
| cg02479575 | MIR7-3;C19orf30 | TSS1500;Body | NA | 2.03E-02 | 4.40E-08 |
| cg02484127 | CCDC48 | Body | Island | -1.42E-02 | 6.77E-05 |
| cg02487453 | OSBP2 | Body | Island | 1.85E-02 | 3.50E-05 |
| cg02489219 | C2orf66 | TSS1500 | NA | -7.72E-03 | 3.24E-07 |
| cg02490185 | SEPN1;SEPN1 | Body;Body | NA | -7.48E-03 | 2.64E-07 |
| cg02494066 | AGRN | Body | Island | -1.10E-02 | 2.81E-06 |
| cg02498722 | FSCN1 | 1stExon | Island | 1.22E-02 | 1.01E-09 |
| cg02499768 | GLT8D1;SPCS1;GLT8 | TSS1500;TSS1500;5 | N_Shore | -1.86E-02 | 3.78E-09 |
| cg02506717 | CREB5 | TSS200 | NA | -1.39E-02 | 1.31E-12 |
| cg02512168 | ZNF205;MGC3771;Z | 5'UTR;Body;5'UTR;E | NA | -2.14E-02 | 3.64E-15 |
| cg02513379 | IL21R;IL21R | TSS200;5'UTR | NA | -1.31E-02 | 2.63E-08 |
| cg02519751 | ZIC1 | TSS1500 | N_Shore | 2.50E-02 | 1.45E-08 |
| cg02530321 | TTPAL;TTPAL | TSS1500;TSS1500 | N_Shore | -6.99E-03 | 3.84E-05 |
| cg02531437 | PRLHR | TSS200 | Island | 1.19E-02 | 1.11E-06 |
| cg02537108 | USP35 | 5'UTR | Island | 1.42E-02 | 5.71E-06 |
| cg02543159 | MPPED1 | 5'UTR | N_Shore | -7.43E-03 | 2.78E-06 |
| cg02543268 | ATP10B | 5'UTR | NA | -1.15E-02 | 3.11E-07 |
| cg02544257 | FUK | Body | S_Shelf | -9.65E-03 | 1.00E-11 |
| cg02546376 | NA | NA | NA | -1.39E-02 | 4.20E-16 |
| cg02553054 | SMARCD1;SMARCD1 | TSS1500;TSS1500 | N_Shore | -1.04E-02 | 8.16E-05 |
| cg02561482 | TFAP2B | 3'UTR | Island | 1.33E-02 | 1.01E-06 |
| cg02565062 | FLJ39609 | TSS1500 | S_Shore | -7.15E-03 | 3.38E-06 |

| | | | | | |
|---|---|---|---|---|---|
| cg02571042 | NA | NA | NA | -1.49E-02 | 5.17E-14 |
| cg02571636 | NA | NA | NA | 2.33E-02 | 1.45E-10 |
| cg02571816 | PPP1R14A | TSS1500 | Island | 2.78E-02 | 1.17E-11 |
| cg02573703 | SOX2OT;SOX2 | Body;TSS1500 | N_Shore | 8.71E-03 | 7.42E-05 |
| cg02596994 | HPD | TSS200 | NA | -6.60E-03 | 3.08E-06 |
| cg02607237 | NA | NA | N_Shelf | -1.25E-02 | 3.51E-08 |
| cg02610106 | FLT4;FLT4 | Body;Body | N_Shelf | -7.68E-03 | 2.54E-06 |
| cg02610600 | STX1A;STX1A | Body;Body | Island | 7.43E-03 | 4.70E-05 |
| cg02620013 | MLNR | 1stExon | Island | 8.98E-03 | 7.20E-08 |
| cg02627216 | IGFBP4 | TSS1500 | N_Shore | -4.29E-03 | 9.52E-05 |
| cg02628704 | NA | NA | NA | -1.34E-02 | 9.84E-09 |
| cg02629106 | PCDP1 | 5'UTR | Island | 1.35E-02 | 7.63E-07 |
| cg02631838 | HPCA | Body | Island | 1.28E-02 | 7.91E-06 |
| cg02639634 | RGS20;RGS20 | Body;TSS1500 | Island | 8.21E-03 | 1.18E-05 |
| cg02641539 | TM7SF4 | TSS1500 | NA | -6.80E-03 | 6.31E-07 |
| cg02646021 | NA | NA | Island | 8.17E-03 | 1.77E-05 |
| cg02646491 | KCNQ1DN | TSS1500 | Island | 1.65E-02 | 4.42E-06 |
| cg02650127 | NA | NA | NA | -1.77E-02 | 7.45E-12 |
| cg02650266 | NA | NA | Island | 3.18E-02 | 9.44E-23 |
| cg02656560 | NA | NA | NA | -8.13E-03 | 6.85E-07 |
| cg02657401 | NA | NA | N_Shore | 1.83E-02 | 9.52E-10 |
| cg02677946 | OPCML | Body | NA | 9.38E-03 | 5.29E-08 |
| cg02693210 | LOC285593 | Body | NA | 8.46E-03 | 2.73E-06 |
| cg02699218 | ANKRD43 | 1stExon | Island | 1.17E-02 | 2.94E-07 |
| cg02699829 | TRMT1;TRMT1;TRM | Body;Body;Body | S_Shore | -1.34E-02 | 1.68E-06 |
| cg02699915 | EXT2;EXT2 | TSS200;TSS200 | N_Shore | -1.55E-02 | 1.30E-07 |
| cg02714303 | LMO7;LMO7 | TSS200;Body | NA | -1.45E-02 | 4.37E-11 |
| cg02724490 | RP1L1 | 5'UTR | NA | -5.14E-03 | 3.88E-05 |
| cg02732067 | SNHG3-RCC1;RCC1;S | Body;TSS1500;Body | N_Shore | -1.63E-02 | 3.53E-10 |
| cg02745847 | IGF2BP1;IGF2BP1 | Body;Body | Island | 1.47E-02 | 8.91E-08 |
| cg02756107 | TRIM4;TRIM4 | TSS1500;TSS1500 | S_Shore | -1.17E-02 | 5.26E-07 |
| cg02758612 | NA | NA | Island | 3.27E-02 | 6.32E-09 |
| cg02759846 | CLEC11A | Body | Island | 8.61E-03 | 7.40E-06 |
| cg02760293 | PPP2R2B;PPP2R2B;I | 5'UTR;Body;5'UTR;T | S_Shore | -1.08E-02 | 1.91E-05 |
| cg02760555 | PLBD2;PLBD2 | 3'UTR;3'UTR | NA | -1.23E-02 | 4.58E-09 |
| cg02792322 | IL17D | Body | S_Shelf | -1.37E-02 | 1.10E-10 |
| cg02792538 | SLC16A8 | Body | Island | 7.91E-03 | 7.95E-09 |
| cg02797271 | GPR132 | TSS1500 | NA | -7.35E-03 | 6.73E-07 |
| cg02801786 | NA | NA | Island | 1.99E-02 | 3.55E-09 |
| cg02811067 | EEPD1 | Body | NA | 3.13E-02 | 1.54E-06 |
| cg02815374 | NA | NA | NA | -4.51E-02 | 6.52E-13 |
| cg02827278 | ORAI1 | TSS1500 | N_Shore | -1.78E-02 | 7.34E-13 |
| cg02837536 | AJAP1;AJAP1 | Body;Body | Island | 2.03E-02 | 1.02E-05 |
| cg02837876 | NA | NA | S_Shelf | -1.03E-02 | 2.44E-07 |
| cg02841912 | SYNE1;SYNE1 | 5'UTR;5'UTR | N_Shore | -1.39E-02 | 4.85E-05 |
| cg02842382 | HIBADH | Body | NA | -1.76E-02 | 1.55E-05 |
| cg02847140 | F3;F3 | 1stExon;5'UTR | Island | 3.27E-02 | 2.00E-05 |
| cg02851873 | ZMIZ1 | 5'UTR | NA | -9.80E-03 | 5.98E-11 |
| cg02853616 | NA | NA | Island | 9.75E-03 | 7.65E-05 |
| cg02868521 | PTPRN2;PTPRN2;PTI | Body;Body;Body | N_Shelf | -9.05E-03 | 7.19E-07 |

| | | | | | |
|---|---|---|---|---|---|
| cg02870946 | NFATC1;NFATC1;NF | 3'UTR;3'UTR;3'UTR; | Island | -1.08E-02 | 3.66E-07 |
| cg02872426 | DDO;DDO | TSS200;TSS200 | NA | -2.60E-02 | 1.12E-12 |
| cg02879159 | TRIM15 | Body | NA | -1.30E-02 | 3.35E-15 |
| cg02881274 | SLC17A7 | Body | Island | 9.86E-03 | 7.17E-08 |
| cg02885007 | HOXD9 | 1stExon | Island | 8.30E-03 | 4.63E-05 |
| cg02886188 | NA | NA | N_Shore | -1.08E-02 | 1.78E-06 |
| cg02891686 | SOD3 | Body | Island | 9.16E-03 | 7.39E-05 |
| cg02898293 | VSX1;VSX1 | Body;Body | Island | 9.61E-03 | 2.05E-11 |
| cg02903680 | PDE8A;PDE8A | Body;Body | S_Shore | -1.17E-02 | 1.10E-06 |
| cg02925222 | NLRP6 | Body | Island | 6.21E-03 | 6.42E-07 |
| cg02930200 | DNALI1 | TSS200 | N_Shore | 6.86E-03 | 3.38E-05 |
| cg02932314 | C17orf104 | TSS200 | Island | 7.45E-03 | 4.30E-05 |
| cg02932780 | VARS | Body | N_Shore | -1.61E-02 | 2.96E-17 |
| cg02933228 | CDC42BPG | Body | N_Shore | -7.21E-03 | 2.84E-06 |
| cg02938414 | NA | NA | S_Shore | 8.73E-03 | 1.83E-08 |
| cg02943290 | C1orf198;C1orf198 | TSS200;TSS1500 | S_Shore | -2.23E-02 | 1.44E-13 |
| cg02944084 | NA | NA | S_Shelf | -7.73E-03 | 3.89E-07 |
| cg02947125 | NA | NA | NA | -1.06E-02 | 2.14E-07 |
| cg02954056 | NA | NA | Island | 9.55E-03 | 1.85E-10 |
| cg02954903 | CCDC80;CCDC80;CC | 1stExon;5'UTR;1stE: | NA | -1.00E-02 | 2.64E-07 |
| cg02961101 | NA | NA | S_Shelf | -1.86E-02 | 1.06E-12 |
| cg02973171 | FAM181B | 1stExon | Island | 2.29E-02 | 2.16E-10 |
| cg02980604 | RASL10B | TSS1500 | N_Shore | -6.50E-03 | 3.47E-07 |
| cg02986451 | CDH20 | Body | NA | -9.18E-03 | 3.25E-09 |
| cg02991558 | NA | NA | NA | -8.01E-03 | 1.93E-06 |
| cg02993991 | ZBTB7A | 5'UTR | Island | -1.44E-02 | 3.09E-09 |
| cg02998394 | EIF4E;EIF4E;EIF4E | Body;Body;Body | N_Shore | -1.69E-02 | 5.33E-11 |
| cg03009030 | HLA-DPA1 | Body | NA | -1.61E-02 | 7.11E-08 |
| cg03010887 | NA | NA | NA | -7.37E-03 | 1.09E-05 |
| cg03013070 | PLCE1;PLCE1 | Body;Body | NA | -1.97E-02 | 1.01E-25 |
| cg03013488 | TNXB | Body | NA | -5.67E-03 | 8.70E-05 |
| cg03013817 | FSIP1 | 5'UTR | N_Shore | -1.28E-02 | 1.60E-06 |
| cg03016906 | NA | NA | N_Shore | 6.07E-03 | 1.36E-05 |
| cg03020208 | AQP5 | TSS1500 | Island | 8.58E-03 | 4.31E-09 |
| cg03022541 | DNAJB8 | TSS1500 | NA | -6.47E-03 | 2.73E-05 |
| cg03035167 | SPATS2L;SPATS2L;SI | Body;Body;Body;Bo | NA | -1.63E-02 | 2.71E-25 |
| cg03035495 | NA | NA | NA | -1.16E-02 | 3.10E-05 |
| cg03039990 | MIR770;MEG3;MEG | TSS1500;Body;Body | NA | -8.42E-03 | 9.51E-05 |
| cg03043157 | FHL5;FHL5 | 5'UTR;5'UTR | NA | -2.45E-02 | 4.57E-12 |
| cg03052182 | KIAA1949;KIAA1949 | Body;1stExon | N_Shore | -1.22E-02 | 7.81E-10 |
| cg03062944 | NA | NA | N_Shelf | -1.86E-02 | 7.17E-08 |
| cg03066577 | C3orf55;C3orf55;C3 | 5'UTR;5'UTR;1stExo | Island | 1.00E-02 | 1.32E-07 |
| cg03069737 | LRWD1;ALKBH4 | TSS1500;Body | N_Shore | -1.14E-02 | 4.59E-09 |
| cg03074925 | NA | NA | N_Shore | 8.30E-03 | 2.95E-08 |
| cg03075966 | GRK5 | Body | NA | -2.78E-02 | 2.85E-08 |
| cg03088990 | PLTP;PLTP | 5'UTR;5'UTR | N_Shore | 7.88E-03 | 4.01E-07 |
| cg03096347 | TUB | Body | NA | -9.35E-03 | 7.57E-05 |
| cg03103661 | NA | NA | NA | -1.51E-02 | 2.94E-15 |
| cg03124318 | USP30 | Body | S_Shore | -1.69E-02 | 3.54E-07 |
| cg03126058 | PRDM16;PRDM16 | Body;Body | NA | -1.38E-02 | 6.31E-07 |

| | | | | | |
|---|---|---|---|---|---|
| cg03131366 | NA | NA | NA | -9.51E-03 | 6.90E-10 |
| cg03132729 | RAP1GAP;RAP1GAP, | Body;5'UTR;5'UTR | N_Shore | -6.55E-03 | 3.62E-07 |
| cg03147185 | NCAPH | Body | NA | -1.00E-02 | 1.47E-15 |
| cg03149128 | NA | NA | Island | 1.18E-02 | 2.83E-05 |
| cg03149958 | NA | NA | NA | -1.76E-02 | 2.10E-11 |
| cg03155159 | NA | NA | NA | -1.24E-02 | 1.26E-05 |
| cg03158310 | SLC9A3R2;SLC9A3R2 | Body;Body | N_Shore | -1.48E-02 | 7.81E-06 |
| cg03158561 | RPL27A;SNORA45 | Body;TSS1500 | S_Shore | -1.44E-02 | 9.02E-14 |
| cg03161498 | NA | NA | NA | -8.28E-03 | 2.08E-07 |
| cg03162314 | CTU1 | 3'UTR | N_Shore | -9.80E-03 | 3.73E-08 |
| cg03171770 | NA | NA | Island | 2.52E-02 | 1.88E-11 |
| cg03176423 | LOC285830;LOC285 | Body;Body | NA | -5.59E-03 | 2.68E-05 |
| cg03184011 | NA | NA | N_Shore | -7.80E-03 | 2.70E-11 |
| cg03193472 | NA | NA | NA | -9.78E-03 | 5.36E-06 |
| cg03194226 | CLEC3B | TSS1500 | NA | -1.49E-02 | 1.51E-17 |
| cg03200166 | SYT7 | Body | Island | 1.60E-02 | 4.29E-07 |
| cg03204605 | AFAP1;AFAP1 | TSS200;TSS200 | Island | 1.26E-02 | 1.45E-05 |
| cg03206925 | EEPD1 | Body | NA | -1.03E-02 | 2.94E-08 |
| cg03214130 | NA | NA | NA | -7.09E-03 | 1.65E-05 |
| cg03223072 | ABLIM1;ABLIM1;ABI | Body;Body;Body | NA | -9.60E-03 | 1.77E-14 |
| cg03224418 | SAMD10;PRPF6 | TSS1500;TSS1500 | N_Shore | -1.23E-02 | 2.10E-12 |
| cg03237606 | TMEM26 | Body | N_Shore | 1.97E-02 | 4.39E-10 |
| cg03239580 | CYP21A2;CYP21A2 | TSS1500;TSS1500 | NA | -9.72E-03 | 7.42E-17 |
| cg03240059 | NA | NA | N_Shore | -1.45E-02 | 1.40E-13 |
| cg03249630 | ANKRD22 | TSS200 | NA | -1.64E-02 | 1.29E-10 |
| cg03259243 | NA | NA | Island | 1.43E-02 | 5.25E-09 |
| cg03262554 | NA | NA | Island | 1.22E-02 | 5.94E-11 |
| cg03269716 | SAMD11 | Body | N_Shore | -1.02E-02 | 2.03E-09 |
| cg03282312 | NA | NA | NA | -1.69E-02 | 4.71E-11 |
| cg03283486 | SMAD9;SMAD9 | 3'UTR;3'UTR | NA | -1.88E-02 | 2.90E-10 |
| cg03289906 | PPP1R9A;PPP1R9A;I | TSS1500;5'UTR;TSS1 | S_Shore | -1.70E-02 | 5.93E-18 |
| cg03290530 | NA | NA | NA | 1.54E-02 | 7.88E-05 |
| cg03299617 | KIF1A | 5'UTR | N_Shelf | -5.96E-03 | 5.40E-06 |
| cg03301200 | NA | NA | Island | 1.69E-02 | 1.07E-05 |
| cg03308038 | NA | NA | NA | -1.40E-02 | 6.18E-08 |
| cg03310838 | ZFPM1 | Body | NA | -1.37E-02 | 1.02E-06 |
| cg03319497 | NA | NA | Island | 7.92E-03 | 4.74E-05 |
| cg03322161 | NA | NA | Island | 1.59E-02 | 3.15E-06 |
| cg03323696 | PDE4D;PDE4D | Body;1stExon | Island | 2.02E-02 | 2.36E-07 |
| cg03325403 | CSMD2 | Body | S_Shore | -7.52E-03 | 2.04E-05 |
| cg03333330 | TFPI2 | TSS1500 | S_Shore | 1.03E-02 | 1.47E-06 |
| cg03335216 | ERRFI1 | TSS1500 | Island | 6.99E-03 | 2.92E-05 |
| cg03337035 | CEACAM6 | TSS1500 | NA | -8.18E-03 | 4.64E-10 |
| cg03342498 | TFAP2A;TFAP2A;TFA | Body;Body;Body | N_Shore | 9.33E-03 | 2.42E-06 |
| cg03344767 | PGD | Body | NA | 6.96E-03 | 3.12E-05 |
| cg03350900 | SOBP | Body | Island | 1.08E-02 | 1.82E-07 |
| cg03354340 | MARVELD3;MARVEI | TSS200;TSS200 | Island | 1.99E-02 | 7.12E-10 |
| cg03354992 | NA | NA | Island | 1.62E-02 | 1.03E-07 |
| cg03359540 | MYLK4 | 5'UTR | NA | -2.33E-02 | 1.75E-22 |
| cg03365733 | PRDM13 | Body | Island | 7.78E-03 | 1.07E-06 |

| | | | | | |
|---|---|---|---|---|---|
| cg03389653 | CBLN1 | TSS1500 | Island | 2.23E-02 | 2.06E-06 |
| cg03389926 | NA | NA | NA | -1.47E-02 | 3.13E-12 |
| cg03393602 | ABHD3 | TSS1500 | S_Shore | -1.81E-02 | 1.84E-08 |
| cg03394122 | MFAP2;MFAP2;MFA | TSS1500;5'UTR;5'U | S_Shore | -9.18E-03 | 2.30E-13 |
| cg03395188 | CTDSP1;MIR26B;CTI | Body;TSS1500;Body | S_Shore | -6.88E-03 | 1.97E-08 |
| cg03399905 | ANKRD34C | 5'UTR | Island | 1.33E-02 | 1.13E-10 |
| cg03420846 | PUM1;PUM1 | Body;Body | NA | -9.34E-03 | 1.91E-07 |
| cg03420881 | NA | NA | Island | 1.53E-02 | 7.48E-08 |
| cg03428981 | EPHB6 | TSS1500 | N_Shore | -1.14E-02 | 2.09E-05 |
| cg03434384 | PPFIBP1;PPFIBP1 | 5'UTR;5'UTR | NA | -7.41E-03 | 4.69E-05 |
| cg03437157 | NA | NA | S_Shore | 8.99E-03 | 1.83E-08 |
| cg03438552 | TAP2;TAP2 | Body;Body | N_Shore | -1.23E-02 | 1.70E-13 |
| cg03455990 | NA | NA | NA | -9.51E-03 | 2.27E-08 |
| cg03464229 | C7orf58;C7orf58 | Body;Body | NA | -1.10E-02 | 9.52E-05 |
| cg03468072 | NA | NA | Island | 8.16E-03 | 1.04E-05 |
| cg03473042 | WDR5 | TSS1500 | N_Shore | -5.71E-03 | 3.15E-05 |
| cg03473532 | MKLN1 | Body | N_Shelf | -2.33E-02 | 1.07E-17 |
| cg03474926 | RALGDS | Body | N_Shore | -5.71E-03 | 7.28E-11 |
| cg03482883 | MAPK8IP3;MAPK8IF | Body;Body | N_Shelf | 8.06E-03 | 9.27E-08 |
| cg03485669 | DLC1 | Body | NA | -1.71E-02 | 6.17E-18 |
| cg03495011 | NA | NA | S_Shelf | -9.42E-03 | 8.39E-05 |
| cg03497133 | NA | NA | Island | 7.47E-03 | 2.09E-06 |
| cg03502002 | GALR1;GALR1 | 1stExon;5'UTR | Island | 2.23E-02 | 6.92E-05 |
| cg03508063 | DLG4;ACADVL;ACAD | TSS1500;Body;Body | S_Shore | -5.09E-03 | 4.42E-05 |
| cg03509094 | EVI5L;EVI5L | Body;Body | Island | 1.19E-02 | 2.72E-11 |
| cg03512426 | NA | NA | Island | 7.11E-03 | 1.72E-05 |
| cg03515656 | IFT140;TMEM204 | Body;Body | S_Shore | -8.95E-03 | 3.82E-06 |
| cg03515663 | PTPRN2;PTPRN2;PTI | Body;Body;Body | Island | 7.08E-03 | 7.84E-05 |
| cg03517919 | KIF1B | Body | NA | -1.56E-02 | 6.80E-15 |
| cg03519967 | MAN1C1 | Body | NA | -1.59E-02 | 1.79E-20 |
| cg03522799 | NA | NA | Island | 5.91E-03 | 4.84E-05 |
| cg03531754 | FAM83A;FAM83A | TSS1500;TSS1500 | NA | -1.70E-02 | 6.76E-14 |
| cg03534662 | LIMD1 | TSS200 | S_Shore | -1.24E-02 | 2.27E-12 |
| cg03545227 | PTPRN | Body | Island | 8.10E-03 | 1.86E-10 |
| cg03548415 | NA | NA | NA | -2.36E-02 | 2.30E-09 |
| cg03553758 | NA | NA | NA | -8.22E-03 | 4.78E-06 |
| cg03556243 | ZBTB20;ZBTB20;ZBT | 5'UTR;1stExon;5'UT | NA | -1.92E-02 | 1.95E-12 |
| cg03568305 | MED24;MED24;SNC | Body;Body;TSS150C | NA | -7.35E-03 | 1.70E-06 |
| cg03572680 | ROBO4 | TSS1500 | NA | -8.80E-03 | 1.15E-06 |
| cg03575969 | STARD10 | Body | N_Shore | -8.73E-03 | 2.88E-06 |
| cg03584351 | NA | NA | NA | -9.57E-03 | 2.87E-05 |
| cg03591499 | RFTN1 | TSS1500 | S_Shore | -1.79E-02 | 6.98E-15 |
| cg03593189 | STX1A;STX1A | Body;Body | Island | 6.33E-03 | 9.43E-06 |
| cg03593280 | FLJ36031;FLJ36031 | 3'UTR;1stExon | N_Shore | -1.68E-02 | 2.74E-08 |
| cg03594078 | PIWIL2;PIWIL2 | TSS1500;TSS1500 | N_Shore | -1.32E-02 | 2.70E-05 |
| cg03607115 | NPNT | 1stExon | Island | 7.30E-03 | 1.32E-05 |
| cg03614193 | ST8SIA3 | Body | Island | 1.52E-02 | 3.62E-11 |
| cg03615565 | FAM65A | Body | Island | -7.32E-03 | 1.49E-07 |
| cg03628000 | PARN;PARN | Body;Body | NA | -2.28E-02 | 1.51E-12 |
| cg03631065 | UGGT2 | Body | NA | -2.04E-02 | 1.85E-14 |

| | | | | | |
|---|---|---|---|---|---|
| cg03634479 | CACNB2;CACNB2;CA | Body;Body;Body;Bo | S_Shore | -1.27E-02 | 8.77E-06 |
| cg03638795 | SIGIRR;SIGIRR | 5'UTR;5'UTR | Island | -1.57E-02 | 1.19E-14 |
| cg03643998 | C1QTNF1;C1QTNF1; | 5'UTR;Body;Body | N_Shore | -1.13E-02 | 5.69E-06 |
| cg03646605 | ME3;ME3;ME3 | TSS1500;TSS200;TS | S_Shore | -1.91E-02 | 4.33E-13 |
| cg03646916 | ZNF167;ZNF167 | TSS200;TSS200 | Island | 1.85E-02 | 2.36E-06 |
| cg03655142 | JTB | TSS1500 | S_Shore | -2.27E-02 | 9.52E-26 |
| cg03660500 | LHX9;LHX9 | Body;Body | Island | 1.00E-02 | 8.55E-05 |
| cg03662545 | NA | NA | NA | -1.52E-02 | 9.47E-09 |
| cg03664992 | BMP8A;BMP8A | 1stExon;5'UTR | Island | 1.28E-02 | 4.26E-10 |
| cg03671597 | SEZ6;SEZ6 | TSS1500;TSS1500 | S_Shore | 8.70E-03 | 3.49E-07 |
| cg03689092 | LIMS2;LIMS2 | 1stExon;5'UTR | NA | -2.22E-02 | 2.96E-09 |
| cg03696974 | SEZ6L2;SEZ6L2;SEZ6 | Body;Body;Body;Bo | S_Shore | 1.25E-02 | 1.13E-05 |
| cg03697308 | NA | NA | S_Shore | 7.76E-03 | 4.14E-05 |
| cg03698343 | ABCA2;C9orf139;AB | Body;TSS200;Body | N_Shore | -5.51E-03 | 1.32E-09 |
| cg03705912 | 11-mar | 1stExon | Island | 1.69E-02 | 7.82E-16 |
| cg03719032 | HAPLN4 | TSS1500 | S_Shelf | -7.62E-03 | 6.47E-05 |
| cg03725309 | SARS | Body | S_Shore | -1.17E-02 | 1.02E-08 |
| cg03727202 | ONECUT1 | Body | N_Shore | 9.55E-03 | 6.18E-09 |
| cg03731828 | NA | NA | N_Shore | -1.38E-02 | 4.89E-08 |
| cg03732087 | AGBL4 | TSS200 | Island | 1.73E-02 | 8.70E-06 |
| cg03734874 | TMEM179 | TSS1500 | Island | 1.13E-02 | 1.73E-08 |
| cg03735592 | NHSL1;NHSL1 | TSS1500;Body | NA | -1.67E-02 | 2.71E-16 |
| cg03738025 | NA | NA | Island | 2.26E-02 | 1.57E-10 |
| cg03738352 | SLC34A2 | TSS200 | Island | 2.59E-02 | 2.90E-09 |
| cg03742763 | APOC3 | TSS1500 | NA | -4.81E-03 | 5.76E-05 |
| cg03746976 | C16orf57 | Body | S_Shore | -1.07E-02 | 5.93E-17 |
| cg03752138 | SOCS3 | TSS1500 | S_Shore | -5.99E-03 | 4.09E-06 |
| cg03754403 | NA | NA | Island | 1.32E-02 | 8.61E-08 |
| cg03758021 | NA | NA | N_Shore | -1.38E-02 | 2.98E-15 |
| cg03759824 | NA | NA | Island | 9.18E-03 | 2.78E-07 |
| cg03762817 | TTC9 | TSS1500 | N_Shore | -2.53E-02 | 1.82E-08 |
| cg03763300 | NA | NA | S_Shelf | -5.12E-02 | 2.78E-06 |
| cg03772384 | CAND2;CAND2 | Body;Body | N_Shore | -7.80E-03 | 6.73E-08 |
| cg03774463 | DLX6AS;DLX6 | Body;Body | S_Shore | 1.01E-02 | 2.80E-05 |
| cg03777083 | WNK4 | TSS1500 | N_Shore | -6.26E-03 | 2.29E-05 |
| cg03779374 | ZBTB20;ZBTB20;ZBT | 5'UTR;TSS1500;5'U | NA | -1.23E-02 | 3.12E-06 |
| cg03781505 | NA | NA | Island | 1.87E-02 | 3.80E-05 |
| cg03788090 | NA | NA | S_Shore | -7.37E-03 | 1.76E-06 |
| cg03811478 | SOX14 | 1stExon | Island | 6.23E-03 | 2.78E-07 |
| cg03817667 | VSTM2A | TSS200 | Island | 2.46E-02 | 3.55E-09 |
| cg03831054 | PCDHGB4;PCDHGA4 | 1stExon;Body;Body | Island | 1.17E-02 | 3.12E-05 |
| cg03841376 | TMEM105 | TSS200 | NA | -6.47E-03 | 1.01E-05 |
| cg03844506 | NA | NA | Island | 1.27E-02 | 4.58E-06 |
| cg03850957 | SNRPB2;SNRPB2 | Body;Body | S_Shelf | -1.09E-02 | 1.12E-06 |
| cg03851401 | NA | NA | S_Shelf | -4.57E-03 | 9.30E-06 |
| cg03854796 | PAX6;PAX6;PAX6 | Body;Body;Body | N_Shore | 8.25E-03 | 4.52E-06 |
| cg03873281 | PDLIM4;PDLIM4 | 3'UTR;3'UTR | S_Shore | -1.71E-02 | 3.77E-09 |
| cg03880407 | VIPR2 | Body | NA | 6.12E-03 | 1.25E-08 |
| cg03884592 | HIVEP3;HIVEP3;HIVE | 1stExon;1stExon;5'U | S_Shore | -2.18E-02 | 1.91E-10 |
| cg03885664 | BBS2 | TSS1500 | S_Shore | -1.21E-02 | 3.44E-05 |

| | | | | | |
|---|---|---|---|---|---|
| cg03886699 | NA | NA | NA | 1.21E-02 | 1.35E-05 |
| cg03890680 | CDC42BPA;CDC42BI | 1stExon;1stExon | N_Shore | -1.69E-02 | 4.76E-05 |
| cg03890691 | DOC2A | TSS1500 | S_Shore | 1.83E-02 | 3.49E-05 |
| cg03894892 | NA | NA | Island | 7.13E-03 | 2.08E-06 |
| cg03900072 | DEFB124 | TSS200 | N_Shelf | -7.76E-03 | 5.40E-05 |
| cg03900143 | ZIC4;ZIC4;ZIC4;ZIC4; | Body;TSS1500;Body | Island | 1.71E-02 | 3.86E-06 |
| cg03901431 | NA | NA | N_Shore | -8.55E-03 | 3.40E-05 |
| cg03906115 | LTBP1;LTBP1;LTBP1; | TSS200;TSS200;Bod | NA | -1.51E-02 | 7.42E-14 |
| cg03915012 | GAK | Body | S_Shelf | -2.78E-02 | 1.56E-28 |
| cg03916277 | ARHGEF16 | Body | NA | -7.83E-03 | 2.29E-05 |
| cg03916421 | SCLT1;C4orf33 | Body;TSS1500 | N_Shore | -1.31E-02 | 2.78E-10 |
| cg03925294 | NA | NA | Island | 6.10E-03 | 3.28E-06 |
| cg03925970 | NA | NA | NA | -1.16E-02 | 6.58E-11 |
| cg03927133 | ITPKA | Body | Island | 1.07E-02 | 2.96E-08 |
| cg03929796 | ALAS1;ALAS1 | TSS1500;TSS1500 | N_Shore | -8.53E-03 | 4.43E-05 |
| cg03932201 | DOCK1 | Body | NA | -7.17E-03 | 3.28E-06 |
| cg03935116 | FAM60A;FAM60A;FI | 5'UTR;5'UTR;TSS15( | N_Shore | -7.29E-03 | 4.68E-05 |
| cg03936663 | NA | NA | NA | 1.56E-02 | 1.78E-05 |
| cg03940098 | MIR663 | Body | Island | 1.29E-02 | 1.55E-08 |
| cg03940484 | SV2B;SV2B | 5'UTR;5'UTR | NA | -1.50E-02 | 6.05E-08 |
| cg03943218 | NA | NA | Island | 1.63E-02 | 1.11E-07 |
| cg03944122 | NA | NA | Island | 5.49E-03 | 1.71E-06 |
| cg03950655 | ROR1;ROR1 | Body;Body | NA | -1.24E-02 | 1.99E-14 |
| cg03956042 | NA | NA | N_Shore | -2.23E-02 | 1.36E-17 |
| cg03957108 | OTX1 | TSS1500 | Island | 1.53E-02 | 2.01E-09 |
| cg03958308 | PLEC1;PLEC1;PLEC1; | Body;Body;Body;Bo | N_Shore | -1.21E-02 | 4.90E-12 |
| cg03959186 | NA | NA | NA | -1.52E-02 | 5.99E-05 |
| cg03962200 | INPP5J | TSS1500 | NA | -8.71E-03 | 2.49E-08 |
| cg03967327 | ALDH5A1;ALDH5A1 | TSS1500;TSS1500 | N_Shore | -2.10E-02 | 6.54E-16 |
| cg03968755 | NKX6-1 | 1stExon | Island | 8.86E-03 | 9.06E-07 |
| cg03969725 | UBE2C;UBE2C;UBE2 | 5'UTR;1stExon;5'UT | S_Shore | -6.97E-03 | 1.49E-06 |
| cg03970615 | IRX3 | Body | Island | 7.48E-03 | 2.04E-06 |
| cg03970849 | ODZ4 | 5'UTR | N_Shore | 9.83E-03 | 7.19E-08 |
| cg03975694 | ZNF540;ZNF540 | 5'UTR;1stExon | S_Shelf | 1.29E-02 | 7.62E-08 |
| cg03981954 | FEZF1;FEZF1 | 3'UTR;3'UTR | N_Shore | 1.58E-02 | 3.35E-06 |
| cg03984009 | PDE7B | TSS1500 | NA | -1.45E-02 | 2.62E-10 |
| cg03989260 | TBX15 | 5'UTR | N_Shore | 7.52E-03 | 1.42E-07 |
| cg03989567 | LGALS8;LGALS8;LGA | TSS1500;5'UTR;TSS: | N_Shore | -1.34E-02 | 7.37E-08 |
| cg03995156 | PPT2;PPT2 | Body;Body | S_Shore | -6.43E-03 | 4.77E-05 |
| cg04006143 | NDUFC1 | TSS1500 | S_Shore | -3.27E-02 | 1.45E-06 |
| cg04006520 | CACNA1C;CACNA1C | Body;Body;Body;Bo | NA | -8.07E-03 | 6.52E-08 |
| cg04019522 | BIN1;BIN1;BIN1;BIN | Body;Body;Body;Bo | NA | -8.50E-03 | 1.40E-08 |
| cg04021562 | SLC44A4 | Body | NA | -5.44E-03 | 1.58E-08 |
| cg04022561 | NA | NA | NA | 1.50E-02 | 8.77E-09 |
| cg04024095 | VSTM2A;VSTM2A | 5'UTR;1stExon | Island | 1.04E-02 | 2.99E-08 |
| cg04028695 | NA | NA | NA | -2.10E-02 | 2.94E-09 |
| cg04037228 | LIMD1 | 1stExon | S_Shore | -1.15E-02 | 4.54E-13 |
| cg04043742 | PLA2G7;PLA2G7;PL/ | TSS200;1stExon;5'U | Island | 7.63E-03 | 2.67E-05 |
| cg04044664 | ANKRD43 | 1stExon | Island | 9.24E-03 | 8.63E-09 |
| cg04051152 | LRP6 | Body | N_Shore | -2.17E-02 | 2.03E-23 |

| | | | | | |
|---|---|---|---|---|---|
| cg04053392 | DACT2 | TSS200 | S_Shore | 1.69E-02 | 1.96E-06 |
| cg04055490 | NA | NA | N_Shore | -7.98E-03 | 6.50E-09 |
| cg04058752 | B3GNT6 | Body | Island | 1.38E-02 | 5.45E-07 |
| cg04061117 | LHFPL4 | 5'UTR | N_Shore | 1.63E-02 | 6.74E-10 |
| cg04061411 | SH2D7 | Body | NA | -9.64E-03 | 1.36E-08 |
| cg04064028 | BBS5 | TSS1500 | N_Shore | -7.84E-03 | 2.72E-08 |
| cg04064644 | NA | NA | Island | 6.02E-03 | 3.03E-05 |
| cg04064963 | SNX9 | TSS1500 | N_Shore | -1.82E-02 | 8.81E-14 |
| cg04079139 | NA | NA | N_Shelf | 1.50E-02 | 3.00E-14 |
| cg04079301 | ZIC4;ZIC4;ZIC4;ZIC4; | 3'UTR;Body;3'UTR;E | N_Shore | 1.36E-02 | 1.77E-09 |
| cg04080625 | KIAA1026 | Body | NA | -6.00E-03 | 6.30E-08 |
| cg04084354 | NA | NA | NA | -1.52E-02 | 2.81E-05 |
| cg04084892 | VSNL1 | 5'UTR | S_Shore | 1.24E-02 | 6.88E-09 |
| cg04090392 | BNC1 | Body | Island | 1.02E-02 | 5.95E-09 |
| cg04098052 | NA | NA | NA | -1.41E-02 | 4.90E-11 |
| cg04101806 | AFF3;AFF3 | TSS1500;5'UTR | S_Shore | -1.22E-02 | 5.94E-07 |
| cg04103761 | NA | NA | N_Shore | 1.42E-02 | 1.84E-11 |
| cg04109092 | IQCE;IQCE | Body;Body | S_Shelf | -9.03E-03 | 8.29E-07 |
| cg04123409 | SDS;SDS | 5'UTR;1stExon | NA | -1.22E-02 | 2.43E-11 |
| cg04124526 | FAM171A2 | Body | S_Shelf | -8.19E-03 | 3.61E-08 |
| cg04127342 | PENK;PENK | Body;Body | Island | 1.06E-02 | 1.07E-05 |
| cg04140663 | CPEB1 | Body | Island | 1.63E-02 | 6.31E-05 |
| cg04141164 | NTSR2 | 1stExon | Island | 1.60E-02 | 1.73E-05 |
| cg04148285 | TCF25 | Body | N_Shore | -7.16E-03 | 1.63E-05 |
| cg04158698 | NA | NA | NA | -1.18E-02 | 1.90E-12 |
| cg04161365 | DHRS13 | TSS1500 | S_Shore | -6.81E-03 | 2.83E-09 |
| cg04168853 | NA | NA | Island | 1.27E-02 | 8.54E-06 |
| cg04172497 | OSBP | Body | N_Shore | -8.64E-03 | 3.76E-08 |
| cg04176122 | BCL9L | Body | N_Shore | -5.50E-03 | 9.83E-05 |
| cg04184232 | C14orf23;C14orf23 | Body;Body | N_Shore | 2.10E-02 | 3.15E-11 |
| cg04191427 | RPTOR;RPTOR | Body;Body | S_Shore | -2.07E-02 | 1.23E-08 |
| cg04193015 | ACACA;ACACA;ACAC | Body;Body;Body;Bo | NA | -1.73E-02 | 1.42E-20 |
| cg04194821 | EGLN3 | Body | NA | -1.07E-02 | 9.28E-11 |
| cg04199354 | PLXNC1 | Body | NA | -1.28E-02 | 2.76E-15 |
| cg04206967 | TRHR | TSS1500 | NA | -1.21E-02 | 2.00E-07 |
| cg04208403 | ZNF423 | Body | N_Shore | -6.58E-03 | 1.24E-09 |
| cg04208743 | NA | NA | NA | -1.05E-02 | 5.53E-12 |
| cg04208928 | LIN9 | TSS1500 | S_Shore | -1.18E-02 | 1.25E-06 |
| cg04211115 | HS3ST3A1 | 1stExon | Island | 7.10E-03 | 1.44E-05 |
| cg04225775 | HMHB1;HMHB1 | 1stExon;5'UTR | NA | 9.37E-03 | 1.95E-05 |
| cg04228468 | SLITRK3 | 5'UTR | NA | 1.17E-02 | 7.13E-10 |
| cg04229059 | SLC38A7 | TSS1500 | S_Shore | -5.46E-03 | 5.34E-05 |
| cg04231319 | MLLT10;MLLT10 | Body;Body | S_Shore | -6.63E-03 | 1.15E-06 |
| cg04232972 | NA | NA | S_Shore | -1.06E-02 | 7.72E-09 |
| cg04234016 | PTPN6;PTPN6;PTPN | Body;Body;Body | NA | -9.03E-03 | 8.84E-07 |
| cg04243368 | NA | NA | N_Shore | -1.49E-02 | 3.56E-06 |
| cg04256466 | PALLD;PALLD;PALLD | Body;Body;Body;TS | N_Shore | -1.89E-02 | 3.27E-11 |
| cg04267101 | EPN3 | Body | N_Shore | -7.38E-03 | 1.77E-07 |
| cg04267526 | NA | NA | Island | 1.08E-02 | 1.39E-09 |
| cg04275362 | RCSD1 | TSS1500 | N_Shore | -9.61E-03 | 2.60E-08 |

| | | | | | |
|---|---|---|---|---|---|
| cg04290427 | NA | NA | S_Shelf | -1.57E-02 | 1.91E-15 |
| cg04293085 | ADRA1D | Body | Island | 2.33E-02 | 6.84E-06 |
| cg04300115 | PRIC285;PRIC285 | Body;TSS1500 | Island | -6.62E-03 | 2.45E-05 |
| cg04305399 | NA | NA | Island | 9.06E-03 | 1.55E-06 |
| cg04307853 | WSCD1 | Body | N_Shelf | -8.30E-03 | 1.08E-06 |
| cg04311403 | EDARADD;EDARADD | Body;Body | S_Shore | -1.40E-02 | 6.55E-11 |
| cg04319659 | NA | NA | Island | 1.69E-02 | 4.26E-08 |
| cg04326808 | C15orf54;C15orf54 | 1stExon;5'UTR | NA | -1.53E-02 | 3.67E-05 |
| cg04329478 | LOC400696 | TSS200 | NA | -2.60E-02 | 1.33E-19 |
| cg04334016 | CNKSR3 | Body | NA | -1.43E-02 | 4.12E-18 |
| cg04339613 | EBF3 | Body | Island | 2.69E-02 | 5.00E-06 |
| cg04354805 | ZDHHC18 | TSS1500 | N_Shore | -7.41E-03 | 6.56E-05 |
| cg04360793 | ELTD1;ELTD1 | 5'UTR;1stExon | Island | 2.50E-02 | 2.00E-12 |
| cg04361926 | NA | NA | N_Shore | -1.38E-02 | 2.43E-13 |
| cg04365452 | NA | NA | Island | 6.97E-03 | 1.94E-06 |
| cg04370106 | ACCN1;ACCN1 | Body;Body | NA | -1.29E-02 | 2.27E-10 |
| cg04370983 | RAD21 | 5'UTR | N_Shore | -2.06E-02 | 2.81E-05 |
| cg04381957 | RFTN1 | 5'UTR | N_Shelf | -1.60E-02 | 1.47E-13 |
| cg04388666 | CCDC85C | TSS1500 | S_Shore | -6.56E-03 | 3.84E-05 |
| cg04391048 | NT5C1B;NT5C1B | Body;Body | Island | 1.66E-02 | 2.65E-08 |
| cg04398978 | CBX4 | TSS1500 | Island | 8.00E-03 | 1.66E-06 |
| cg04400972 | TRIM45;TRIM45 | TSS1500;TSS1500 | Island | 2.36E-02 | 6.17E-10 |
| cg04401986 | FGF5;FGF5 | Body;Body | S_Shelf | 2.18E-02 | 5.52E-06 |
| cg04404381 | SHANK2 | Body | N_Shore | -9.17E-03 | 3.71E-07 |
| cg04405704 | NA | NA | N_Shore | -1.61E-02 | 8.41E-12 |
| cg04409030 | SOCS2 | TSS1500 | N_Shore | -1.04E-02 | 2.13E-11 |
| cg04415599 | NA | NA | Island | 1.04E-02 | 2.52E-07 |
| cg04416734 | ALDOA;ALDOA;ALDO | TSS1500;TSS1500;5 | N_Shore | -8.99E-03 | 7.12E-09 |
| cg04422802 | NA | NA | S_Shore | -1.22E-02 | 1.20E-09 |
| cg04427254 | NA | NA | S_Shelf | -1.76E-02 | 1.76E-12 |
| cg04427651 | ZDHHC18 | TSS1500 | N_Shore | -6.15E-03 | 1.97E-07 |
| cg04433322 | SLC4A1 | TSS1500 | NA | -1.05E-02 | 8.44E-10 |
| cg04436528 | BAI1 | Body | N_Shore | -4.29E-03 | 7.50E-05 |
| cg04439458 | SIL1;SIL1 | 5'UTR;5'UTR | NA | -1.50E-02 | 2.17E-14 |
| cg04448477 | PDGFRA | TSS200 | N_Shore | -1.04E-02 | 7.74E-08 |
| cg04450459 | NA | NA | Island | 8.73E-03 | 8.12E-07 |
| cg04467589 | RNF39;RNF39 | TSS1500;TSS1500 | S_Shore | -7.73E-03 | 6.22E-05 |
| cg04467618 | TCF21;TCF21 | 1stExon;1stExon | Island | 1.23E-02 | 8.36E-08 |
| cg04468081 | MCF2L2;B3GNT5 | Body;5'UTR | NA | -1.43E-02 | 8.85E-05 |
| cg04470072 | PSKH2 | TSS200 | Island | 1.37E-02 | 9.26E-07 |
| cg04474049 | CAV1 | TSS1500 | N_Shore | -8.25E-03 | 6.76E-05 |
| cg04474832 | ABHD14B;ABHD14B | 1stExon;Body;5'UTR | N_Shore | -8.23E-03 | 2.77E-11 |
| cg04479212 | ITGA9 | Body | S_Shore | -9.08E-03 | 1.50E-05 |
| cg04481603 | NA | NA | S_Shore | -7.10E-03 | 5.40E-06 |
| cg04483701 | NA | NA | NA | -1.17E-02 | 6.92E-09 |
| cg04485799 | IL17REL | Body | Island | 1.02E-02 | 2.82E-10 |
| cg04486778 | NA | NA | Island | 1.50E-02 | 3.89E-05 |
| cg04486873 | C1orf94;C1orf94 | Body;5'UTR | S_Shore | -1.06E-02 | 3.82E-06 |
| cg04489548 | NA | NA | S_Shelf | -1.58E-02 | 6.83E-14 |
| cg04500377 | DDHD2;DDHD2;DDH | 5'UTR;5'UTR;5'UTR | S_Shore | -1.72E-02 | 1.11E-22 |

| | | | | | |
|---|---|---|---|---|---|
| cg04501188 | FOXD2 | 1stExon | Island | 2.08E-02 | 6.75E-07 |
| cg04503319 | ANKRD11 | Body | N_Shelf | -1.07E-02 | 2.58E-11 |
| cg04504043 | ELF5;ELF5 | 5'UTR;TSS1500 | NA | -1.46E-02 | 1.22E-10 |
| cg04505750 | AADACL3;AADACL3 | TSS1500;TSS1500 | NA | -9.45E-03 | 1.95E-06 |
| cg04507775 | NID1 | 1stExon | Island | 9.62E-03 | 3.43E-05 |
| cg04510153 | DDR2;DDR2 | 5'UTR;5'UTR | NA | 1.12E-02 | 4.64E-09 |
| cg04519462 | HRNBP3 | 5'UTR | NA | -1.42E-02 | 1.12E-07 |
| cg04521004 | SHOX2;SHOX2;SHO› | Body;Body;Body | Island | 1.07E-02 | 4.16E-06 |
| cg04525757 | FOXG1 | TSS1500 | N_Shore | 2.16E-02 | 9.79E-06 |
| cg04526087 | NA | NA | NA | -1.29E-02 | 9.03E-09 |
| cg04528819 | KLF14 | 1stExon | Island | 1.16E-02 | 2.51E-07 |
| cg04536704 | PPT2;PPT2 | Body;Body | S_Shore | -9.48E-03 | 1.83E-11 |
| cg04536829 | SEC61A1 | Body | S_Shore | -1.30E-02 | 5.03E-11 |
| cg04543289 | CACNG3;CACNG3 | 1stExon;5'UTR | S_Shore | 8.63E-03 | 4.92E-07 |
| cg04546041 | ZNF304 | Body | Island | 1.23E-02 | 5.25E-05 |
| cg04550712 | NA | NA | NA | -1.35E-02 | 3.38E-10 |
| cg04552470 | VWA5B1 | Body | Island | 1.96E-02 | 1.89E-08 |
| cg04553690 | PCDHGA4;PCDHGA6 | Body;Body;Body;Bo | Island | 9.83E-03 | 3.54E-06 |
| cg04567334 | CDH23 | Body | NA | -5.31E-03 | 1.01E-05 |
| cg04571163 | ST6GAL2;ST6GAL2;S | 5'UTR;5'UTR;5'UTR | Island | 6.51E-03 | 4.00E-05 |
| cg04573078 | INF2;INF2;INF2 | TSS1500;TSS1500;T | N_Shore | -1.99E-02 | 6.38E-13 |
| cg04573702 | MYH6;MIR208A | Body;Body | N_Shore | -5.78E-03 | 6.66E-05 |
| cg04578903 | NA | NA | Island | 7.17E-03 | 2.01E-05 |
| cg04581938 | NA | NA | N_Shelf | -1.18E-02 | 4.06E-06 |
| cg04596004 | NA | NA | Island | 6.53E-03 | 8.00E-05 |
| cg04596060 | RBM19;RBM19;RBM | TSS1500;TSS1500;T | S_Shore | -1.06E-02 | 4.08E-07 |
| cg04597545 | PTBP1;PTBP1;PTBP1 | Body;Body;Body;Bo | S_Shore | -6.42E-03 | 4.72E-07 |
| cg04598121 | PENK;PENK | Body;1stExon | Island | 8.95E-03 | 4.56E-06 |
| cg04598517 | SLC6A3;SLC6A3 | 5'UTR;1stExon | Island | 1.17E-02 | 6.34E-08 |
| cg04601137 | ADAMTSL5 | 5'UTR | N_Shore | -7.98E-03 | 7.55E-06 |
| cg04602284 | C3orf15 | Body | Island | 1.31E-02 | 1.08E-06 |
| cg04604946 | LRRC23;ENO2;LRRC2 | 3'UTR;TSS1500;3'U | Island | -7.57E-03 | 3.91E-09 |
| cg04606861 | GALNT9 | 1stExon | Island | 8.55E-03 | 1.50E-06 |
| cg04614290 | LSM7;SPPL2B;SPPL2 | Body;TSS1500;TSS1 | N_Shore | -8.82E-03 | 4.48E-05 |
| cg04621728 | NA | NA | NA | -1.03E-02 | 2.25E-06 |
| cg04621893 | NA | NA | Island | 1.22E-02 | 2.30E-08 |
| cg04622024 | EPHA10 | Body | Island | 7.26E-03 | 6.01E-05 |
| cg04622802 | FIBIN;FIBIN | 5'UTR;1stExon | NA | 1.13E-02 | 3.62E-05 |
| cg04631994 | THSD4 | Body | NA | -1.23E-02 | 2.27E-05 |
| cg04636834 | C16orf13;C16orf13; | Body;Body;Body;Bo | Island | -9.41E-03 | 4.76E-10 |
| cg04640528 | NA | NA | S_Shelf | -1.07E-02 | 3.49E-10 |
| cg04651240 | LOC494141;LOC494 | TSS1500;TSS1500;T | N_Shore | -1.47E-02 | 2.75E-17 |
| cg04652943 | NA | NA | N_Shore | 1.49E-02 | 3.27E-05 |
| cg04657183 | NA | NA | NA | -1.21E-02 | 5.19E-10 |
| cg04659729 | VARS | Body | N_Shore | -8.91E-03 | 2.11E-06 |
| cg04662594 | EPB49;EPB49;EPB49 | 5'UTR;5'UTR;5'UTR; | S_Shelf | -1.08E-02 | 7.91E-10 |
| cg04665046 | NA | NA | Island | -6.39E-03 | 4.46E-05 |
| cg04666029 | KCNQ1;KCNQ1 | Body;Body | N_Shore | -5.42E-03 | 3.49E-05 |
| cg04669407 | ABLIM3 | 5'UTR | Island | -9.90E-03 | 3.00E-06 |
| cg04670802 | NA | NA | N_Shore | 1.78E-02 | 3.24E-05 |

| | | | | | |
|---|---|---|---|---|---|
| cg04671739 | ASB10;ASB10;ASB10 | TSS1500;TSS200;TS: | NA | -8.07E-03 | 2.54E-09 |
| cg04671742 | LAG3;LAG3 | 1stExon;5'UTR | N_Shore | -1.48E-02 | 6.17E-20 |
| cg04673446 | MGAT3 | 5'UTR | N_Shelf | -7.78E-03 | 6.63E-06 |
| cg04674508 | HLA-J;NCRNA00171 | Body;Body | Island | 7.03E-03 | 7.07E-05 |
| cg04674529 | NA | NA | NA | -1.78E-02 | 1.36E-07 |
| cg04682133 | USP24 | TSS1500 | S_Shore | -5.67E-03 | 1.93E-05 |
| cg04685387 | PARD3 | Body | NA | -1.17E-02 | 7.99E-08 |
| cg04692403 | TCF21;TCF21 | 1stExon;1stExon | Island | 9.65E-03 | 1.06E-08 |
| cg04695027 | CATSPER1;CATSPER: | 1stExon;5'UTR | S_Shelf | -1.07E-02 | 2.39E-09 |
| cg04700814 | HEXIM1 | TSS1500 | N_Shore | -7.50E-03 | 5.28E-05 |
| cg04714110 | NA | NA | Island | 9.16E-03 | 5.95E-06 |
| cg04721098 | CACNG3 | TSS1500 | N_Shore | -1.59E-02 | 9.01E-10 |
| cg04726821 | MLH1;MLH1;MLH1; | TSS1500;TSS1500;T | N_Shore | -1.37E-02 | 7.58E-06 |
| cg04731988 | OXT | TSS1500 | N_Shore | -9.39E-03 | 3.62E-09 |
| cg04732840 | NA | NA | NA | -4.68E-02 | 3.02E-05 |
| cg04733838 | IRAK2 | TSS1500 | N_Shore | -9.10E-03 | 1.28E-08 |
| cg04738237 | GLI2 | Body | N_Shore | -7.49E-03 | 6.61E-06 |
| cg04739647 | HOXD9;HOXD9 | 5'UTR;1stExon | Island | 2.63E-02 | 6.49E-07 |
| cg04740205 | PCF11 | Body | S_Shelf | -4.10E-02 | 6.80E-10 |
| cg04742397 | NA | NA | S_Shelf | -1.02E-02 | 1.75E-12 |
| cg04747322 | SNCAIP | TSS1500 | N_Shore | 1.07E-02 | 1.96E-05 |
| cg04748834 | CHL1 | 5'UTR | Island | 9.15E-03 | 3.36E-05 |
| cg04749646 | MARCH4;MARCH4 | 5'UTR;1stExon | Island | 1.03E-02 | 6.93E-06 |
| cg04753583 | NA | NA | Island | 1.24E-02 | 1.71E-11 |
| cg04756597 | NA | NA | S_Shelf | -4.89E-03 | 2.71E-05 |
| cg04760021 | WNT16;WNT16 | Body;Body | Island | 1.18E-02 | 9.80E-05 |
| cg04766005 | NA | NA | S_Shore | -6.55E-03 | 7.03E-05 |
| cg04766061 | NA | NA | N_Shelf | -1.10E-02 | 4.21E-25 |
| cg04771285 | NA | NA | NA | -9.95E-03 | 5.93E-16 |
| cg04774505 | NRL | 3'UTR | N_Shore | -1.24E-02 | 6.01E-13 |
| cg04775627 | NA | NA | NA | -7.32E-03 | 4.11E-06 |
| cg04779720 | NPPB | TSS1500 | Island | 2.37E-02 | 1.25E-09 |
| cg04788390 | ARHGEF10L;ARHGEI | Body;Body | NA | -1.19E-02 | 1.14E-13 |
| cg04791601 | NA | NA | N_Shore | -1.27E-02 | 4.66E-12 |
| cg04792712 | MTMR7 | TSS200 | Island | 7.44E-03 | 1.17E-06 |
| cg04795774 | NA | NA | Island | 1.60E-02 | 1.59E-05 |
| cg04806942 | NA | NA | Island | 7.98E-03 | 5.68E-06 |
| cg04812351 | ADAMTS3 | Body | NA | -1.78E-02 | 2.03E-16 |
| cg04816013 | KALRN;KALRN | Body;Body | N_Shore | -8.66E-03 | 2.40E-08 |
| cg04820362 | LRRC43 | TSS1500 | NA | -1.03E-02 | 8.83E-08 |
| cg04826368 | NA | NA | NA | -3.61E-02 | 5.42E-07 |
| cg04830316 | OPTC | Body | NA | -9.94E-03 | 3.88E-10 |
| cg04833898 | SNORA10;RPS2 | Body;Body | N_Shore | -1.49E-02 | 4.71E-09 |
| cg04834097 | LOC728927 | 5'UTR | NA | -9.06E-03 | 6.45E-09 |
| cg04834502 | N4BP1 | TSS1500 | S_Shore | -1.10E-02 | 1.63E-06 |
| cg04839289 | JPH4;JPH4 | Body;Body | Island | 1.73E-02 | 2.71E-07 |
| cg04841371 | SPRR1A | 3'UTR | NA | -9.72E-03 | 8.54E-08 |
| cg04844693 | EPB41;EPB41;EPB41 | 5'UTR;5'UTR;5'UTR; | N_Shore | -1.14E-02 | 1.70E-14 |
| cg04848925 | TTLL6 | Body | Island | 8.54E-03 | 2.59E-07 |
| cg04851268 | GHSR;GHSR | TSS1500;TSS1500 | N_Shore | 1.17E-02 | 9.36E-05 |

| | | | | | |
|---|---|---|---|---|---|
| cg04856022 | PPT2;PPT2 | Body;Body | S_Shore | -8.49E-03 | 2.13E-10 |
| cg04858155 | ALX4 | TSS1500 | Island | 1.18E-02 | 3.28E-09 |
| cg04859466 | SOX2OT | Body | Island | 1.52E-02 | 4.52E-05 |
| cg04861640 | ZNF187;ZNF187;ZNF | TSS200;TSS200;TSS. | N_Shore | -3.18E-02 | 1.35E-13 |
| cg04865491 | NA | NA | Island | 2.06E-02 | 6.73E-07 |
| cg04865692 | KCNC3 | 1stExon | Island | 1.13E-02 | 1.09E-05 |
| cg04865976 | NA | NA | NA | -1.48E-02 | 2.00E-06 |
| cg04872610 | AP4B1;DCLRE1B | TSS1500;Body | S_Shore | -8.62E-03 | 2.29E-07 |
| cg04872612 | ADRA1D | 3'UTR | Island | 9.34E-03 | 3.75E-06 |
| cg04875128 | OTUD7A | Body | Island | 3.79E-02 | 1.38E-17 |
| cg04885881 | NA | NA | S_Shelf | -2.09E-02 | 3.02E-23 |
| cg04890576 | NA | NA | S_Shore | -2.57E-02 | 1.42E-18 |
| cg04891961 | ANKRD13B | Body | Island | 1.15E-02 | 2.04E-07 |
| cg04894642 | LOC100302401;RAS. | Body;TSS200 | Island | 9.89E-03 | 1.50E-05 |
| cg04895616 | ZNF283 | TSS1500 | NA | 1.19E-02 | 2.25E-07 |
| cg04896437 | NA | NA | NA | -6.47E-03 | 1.58E-07 |
| cg04899492 | NRSN1 | TSS200 | Island | 1.14E-02 | 5.95E-05 |
| cg04908625 | ADCY5 | 1stExon | Island | 1.82E-02 | 4.52E-14 |
| cg04911680 | AHDC1 | 5'UTR | Island | -8.13E-03 | 5.33E-10 |
| cg04913979 | SHOX2;SHOX2;SHOX | Body;Body;Body | N_Shore | 1.59E-02 | 1.06E-09 |
| cg04927004 | MIR124-3 | TSS1500 | Island | 9.52E-03 | 3.26E-06 |
| cg04928670 | NA | NA | NA | -1.33E-02 | 4.18E-11 |
| cg04931184 | KCNJ4;KCNJ4 | 5'UTR;TSS1500 | NA | -5.92E-03 | 2.87E-06 |
| cg04931216 | DMRTA2 | Body | Island | 6.44E-03 | 7.39E-06 |
| cg04931256 | NA | NA | Island | 1.11E-02 | 9.52E-06 |
| cg04931539 | KCNQ2;KCNQ2;KCN | Body;Body;Body;Bo | N_Shore | -1.94E-02 | 1.54E-16 |
| cg04935434 | NA | NA | Island | 1.11E-02 | 5.04E-05 |
| cg04940570 | TEAD1 | 5'UTR | Island | 2.41E-02 | 5.75E-13 |
| cg04943986 | NR2F2;NR2F2 | TSS1500;Body | Island | 9.80E-03 | 6.58E-06 |
| cg04945379 | CIITA | TSS1500 | N_Shelf | -1.31E-02 | 4.88E-15 |
| cg04950301 | PHACTR1 | Body | Island | 7.33E-03 | 1.54E-06 |
| cg04955333 | IQCE;IQCE | Body;Body | S_Shore | -7.18E-03 | 1.28E-05 |
| cg04955914 | C2orf24 | Body | N_Shore | -1.47E-02 | 2.97E-20 |
| cg04956949 | STX1A;STX1A | Body;Body | S_Shore | -8.32E-03 | 7.21E-13 |
| cg04957377 | NA | NA | NA | -1.32E-02 | 4.32E-12 |
| cg04959790 | NR1H3;NR1H3;NR1H | 5'UTR;TSS1500;TSS. | NA | -9.71E-03 | 3.60E-12 |
| cg04965987 | DSCR6 | TSS1500 | Island | 8.70E-03 | 2.64E-07 |
| cg04970434 | GP1BA | 3'UTR | NA | -9.04E-03 | 6.99E-09 |
| cg04976151 | POLR2C | TSS1500 | N_Shore | -1.46E-02 | 7.63E-13 |
| cg04977602 | PRLR;PRLR | 1stExon;5'UTR | NA | -9.32E-03 | 7.36E-06 |
| cg04980928 | LRTM2;LRTM2;CACN | Body;Body;Body;Bo | NA | 6.37E-03 | 5.63E-06 |
| cg04983142 | GFRA1;GFRA1;GFRA | 5'UTR;TSS1500;5'U | Island | 6.16E-03 | 1.69E-08 |
| cg04986004 | MIR874;KLHL3 | TSS1500;Body | NA | -8.63E-03 | 7.21E-05 |
| cg04986276 | TCP10L | 5'UTR | NA | -8.13E-03 | 2.00E-09 |
| cg04986304 | NA | NA | S_Shelf | -9.58E-03 | 4.12E-07 |
| cg04986579 | UNC13A | TSS1500 | S_Shore | 7.16E-03 | 6.75E-05 |
| cg04999352 | RARRES3 | Body | NA | -1.32E-02 | 6.94E-10 |
| cg04999945 | CIITA | Body | S_Shore | -8.68E-03 | 2.94E-07 |
| cg05007163 | CBX6 | Body | N_Shore | -6.94E-03 | 5.55E-07 |
| cg05009601 | FEZF1;FEZF1;FEZF1;I | 1stExon;1stExon;5'U | Island | 1.40E-02 | 3.07E-09 |

| | | | | | |
|---|---|---|---|---|---|
| cg05016408 | LOC134466 | TSS200 | Island | 1.59E-02 | 3.91E-06 |
| cg05018513 | INF2;INF2 | Body;Body | NA | -1.04E-02 | 1.96E-10 |
| cg05024939 | NA | NA | N_Shore | 1.12E-02 | 3.76E-10 |
| cg05030953 | HLA-C | TSS1500 | S_Shore | -3.40E-02 | 2.62E-06 |
| cg05041351 | C16orf73;C16orf73; | 5'UTR;TSS200;1stEx | Island | 1.44E-02 | 9.87E-08 |
| cg05045027 | NA | NA | NA | -8.95E-03 | 3.26E-06 |
| cg05046306 | NA | NA | N_Shore | -1.10E-02 | 8.07E-11 |
| cg05048976 | WNT5A | Body | Island | 1.09E-02 | 6.37E-07 |
| cg05053440 | NA | NA | Island | 1.33E-02 | 9.16E-06 |
| cg05061804 | TTF1 | TSS200 | S_Shore | -9.06E-03 | 5.72E-06 |
| cg05062889 | C16orf13;C16orf13; | TSS1500;TSS1500;T | S_Shore | 1.02E-02 | 6.57E-08 |
| cg05076755 | LRRC32;LRRC32 | 5'UTR;TSS200 | N_Shore | -1.16E-02 | 4.17E-08 |
| cg05081167 | NA | NA | NA | -1.26E-02 | 7.33E-10 |
| cg05081614 | STRN3;STRN3;AP4S1 | Body;Body;TSS1500 | N_Shore | -1.24E-02 | 6.83E-07 |
| cg05083128 | WDR1;WDR1 | Body;Body | N_Shore | -1.44E-02 | 2.06E-11 |
| cg05093315 | SAAL1 | TSS1500 | S_Shore | -1.05E-02 | 4.29E-05 |
| cg05101437 | CDK6;CDK6 | Body;Body | NA | -1.48E-02 | 7.29E-06 |
| cg05104897 | NA | NA | NA | -5.57E-02 | 1.71E-06 |
| cg05114898 | NA | NA | S_Shore | -1.11E-02 | 9.33E-10 |
| cg05121182 | KALRN;KALRN;KALR | TSS1500;TSS1500;T | NA | -7.22E-03 | 1.28E-06 |
| cg05121480 | SST | Body | N_Shore | 1.77E-02 | 1.05E-13 |
| cg05135521 | RBMS1;RBMS1 | Body;Body | NA | -1.55E-02 | 6.62E-08 |
| cg05139788 | TFAP2D | Body | N_Shore | 8.89E-03 | 2.02E-06 |
| cg05147578 | PEX26;PEX26 | TSS1500;TSS1500 | N_Shore | -2.44E-02 | 5.51E-10 |
| cg05156137 | RCAN1;RCAN1;RCAN | 5'UTR;Body;1stExon | NA | -2.11E-02 | 1.20E-15 |
| cg05157098 | THAP2 | Body | S_Shore | -4.13E-02 | 2.18E-10 |
| cg05157541 | NA | NA | S_Shore | -7.28E-03 | 8.10E-06 |
| cg05158615 | NPY | TSS1500 | Island | 1.05E-02 | 3.30E-06 |
| cg05163057 | SPPL2B;SPPL2B;LSM | Body;Body;TSS1500 | S_Shore | -6.79E-03 | 3.83E-06 |
| cg05163510 | RPS6KB2 | Body | N_Shelf | -9.39E-03 | 1.28E-08 |
| cg05166490 | ANK1;ANK1 | 1stExon;5'UTR | Island | 2.07E-02 | 2.01E-05 |
| cg05167251 | HOXD9;HOXD9 | 5'UTR;1stExon | Island | 2.11E-02 | 6.41E-05 |
| cg05168491 | NA | NA | Island | 9.22E-03 | 8.27E-14 |
| cg05178291 | SAA1;SAA1 | TSS200;TSS200 | NA | -5.22E-03 | 9.47E-05 |
| cg05194346 | NA | NA | NA | -1.42E-02 | 1.58E-16 |
| cg05195452 | NA | NA | NA | -8.72E-03 | 8.72E-07 |
| cg05200313 | SLC24A4;SLC24A4;S | 3'UTR;3'UTR;3'UTR | NA | -1.29E-02 | 1.43E-09 |
| cg05201185 | HLA-E | Body | S_Shore | -8.09E-03 | 3.89E-07 |
| cg05205351 | NOP56;SNORD110;N | Body;TSS200;Body;T | S_Shore | -8.76E-03 | 1.76E-07 |
| cg05207465 | GEMIN5 | TSS1500 | S_Shore | -6.71E-03 | 1.16E-06 |
| cg05211868 | NA | NA | N_Shore | -2.30E-02 | 4.64E-05 |
| cg05213896 | IL4I1;IL4I1 | Body;Body | Island | 1.77E-02 | 1.68E-07 |
| cg05215925 | OPRM1;OPRM1;OPI | 1stExon;1stExon;1s | Island | 1.12E-02 | 1.22E-08 |
| cg05223210 | DSCAML1 | Body | NA | -6.41E-03 | 4.85E-07 |
| cg05228408 | CLCN6;CLCN6;MTHF | TSS1500;TSS1500;5 | N_Shore | -8.77E-03 | 5.54E-15 |
| cg05230054 | CHST8;CHST8;CHST8 | 5'UTR;TSS200;5'UTI | Island | 2.14E-02 | 4.93E-09 |
| cg05235151 | TCP11;TCP11 | Body;5'UTR | N_Shore | -2.00E-02 | 4.48E-05 |
| cg05240166 | TMEM159;DNAH3 | 5'UTR;TSS1500 | S_Shore | -7.48E-03 | 3.59E-05 |
| cg05242244 | CSAD | Body | NA | -9.02E-03 | 4.85E-16 |
| cg05248321 | KLHL33 | Body | NA | 9.54E-03 | 1.01E-06 |

| | | | | | |
|---|---|---|---|---|---|
| cg05253759 | FLJ25006 | TSS200 | NA | 4.91E-03 | 2.25E-05 |
| cg05255522 | TFAP2D | Body | Island | 2.15E-02 | 7.50E-13 |
| cg05261596 | MYRIP | Body | NA | -7.29E-03 | 4.64E-09 |
| cg05262877 | GUCA2A | TSS1500 | NA | -7.43E-03 | 6.10E-07 |
| cg05265596 | LCP2 | Body | NA | -1.02E-02 | 1.60E-07 |
| cg05266781 | IRX5 | Body | Island | 8.54E-03 | 3.99E-07 |
| cg05267955 | VARS2;VARS2;VARS: | TSS1500;TSS1500;T | Island | -9.05E-03 | 5.73E-05 |
| cg05296057 | TLE2;TLE2;TLE2 | TSS1500;Body;TSS1 | S_Shore | -8.63E-03 | 7.75E-07 |
| cg05299836 | BCKDK;BCKDK | TSS1500;TSS1500 | N_Shore | -5.46E-03 | 2.18E-05 |
| cg05301866 | TBR1 | Body | Island | 8.28E-03 | 5.72E-06 |
| cg05302489 | VARS | Body | N_Shelf | -2.03E-02 | 5.44E-17 |
| cg05303901 | CREB5 | TSS1500 | NA | -2.70E-02 | 9.41E-09 |
| cg05316627 | NA | NA | N_Shore | -2.40E-02 | 1.06E-20 |
| cg05317396 | RAMP1 | Body | NA | -1.82E-02 | 3.47E-13 |
| cg05324516 | NA | NA | NA | -1.72E-02 | 8.75E-17 |
| cg05329352 | ADRA2A | 1stExon | Island | 1.29E-02 | 8.01E-08 |
| cg05331498 | CHDH;IL17RB | TSS1500;Body | Island | 6.44E-03 | 6.66E-05 |
| cg05331856 | NA | NA | NA | -8.15E-03 | 3.87E-06 |
| cg05338672 | HLA-C | TSS1500 | S_Shore | -2.32E-02 | 4.29E-05 |
| cg05339605 | TTC7B | Body | NA | -1.40E-02 | 8.53E-12 |
| cg05339727 | SLC38A11 | TSS200 | NA | -1.37E-02 | 4.68E-06 |
| cg05346491 | GRIN2D | Body | N_Shore | -7.52E-03 | 4.92E-10 |
| cg05350798 | NA | NA | NA | 7.77E-03 | 6.50E-05 |
| cg05352500 | WTIP | TSS1500 | Island | 1.74E-02 | 4.57E-06 |
| cg05354479 | MAP3K9 | TSS1500 | S_Shore | -1.37E-02 | 1.91E-08 |
| cg05358170 | HLA-F;HLA-F;HLA-F | Body;Body;Body | S_Shore | -1.06E-02 | 1.00E-06 |
| cg05360577 | NA | NA | Island | 7.90E-03 | 1.20E-05 |
| cg05360958 | MGP | Body | NA | -1.31E-02 | 8.82E-07 |
| cg05371578 | ZIC1 | 1stExon | Island | 1.42E-02 | 3.22E-08 |
| cg05373251 | NA | NA | S_Shore | -2.27E-02 | 1.46E-12 |
| cg05376937 | CNIH3 | Body | NA | -1.03E-02 | 2.30E-08 |
| cg05377527 | PTPMT1;PTPMT1 | TSS1500;TSS1500 | N_Shore | -4.83E-03 | 1.67E-05 |
| cg05379350 | GIT1;GIT1 | TSS1500;TSS1500 | N_Shore | -1.14E-02 | 3.99E-12 |
| cg05381960 | JTB;CREB3L4 | 3'UTR;3'UTR | N_Shelf | -7.58E-03 | 6.98E-06 |
| cg05386493 | FOXL2;C3orf72;FOXl | 1stExon;TSS1500;5' | Island | 9.00E-03 | 2.45E-05 |
| cg05396212 | KRT19 | Body | N_Shore | 7.90E-03 | 1.72E-06 |
| cg05398903 | NA | NA | N_Shore | 1.68E-02 | 1.50E-05 |
| cg05401965 | PCDH10;PCDH10 | TSS1500;TSS1500 | Island | 1.06E-02 | 1.39E-06 |
| cg05405914 | NA | NA | NA | -1.51E-02 | 1.95E-18 |
| cg05412028 | ABCC4;ABCC4 | Body;Body | N_Shore | -2.71E-02 | 6.22E-05 |
| cg05417162 | NA | NA | Island | 1.79E-02 | 1.84E-06 |
| cg05425754 | CCNO | Body | Island | 9.09E-03 | 1.22E-07 |
| cg05442902 | MGC16703;P2RX6;P | TSS1500;TSS1500;T | S_Shore | -4.91E-03 | 5.92E-07 |
| cg05452406 | GCM2 | Body | Island | 2.90E-02 | 1.19E-11 |
| cg05454635 | CYP11A1;CYP11A1 | Body;5'UTR | N_Shore | -1.23E-02 | 2.15E-11 |
| cg05469614 | ZSCAN10;ZSCAN10 | 1stExon;5'UTR | S_Shelf | -5.66E-03 | 2.09E-05 |
| cg05470166 | HRCT1 | TSS1500 | NA | -7.67E-03 | 3.24E-08 |
| cg05471730 | PID1;PID1;PID1 | 5'UTR;1stExon;1stE: | Island | 7.62E-03 | 1.07E-05 |
| cg05471845 | CCK | TSS200 | Island | 1.05E-02 | 8.15E-06 |
| cg05476568 | SGEF | Body | Island | 1.12E-02 | 1.04E-05 |

| | | | | | |
|---|---|---|---|---|---|
| cg05488632 | EPHX3;EPHX3;EPHX: | 1stExon;5'UTR;5'UT | Island | 5.40E-03 | 4.30E-05 |
| cg05490029 | IL7 | TSS1500 | S_Shore | -1.81E-02 | 1.13E-13 |
| cg05500840 | HOXD11 | TSS200 | Island | 2.03E-02 | 5.94E-05 |
| cg05517711 | ST6GALNAC6 | TSS1500 | S_Shore | -9.11E-03 | 1.99E-11 |
| cg05533615 | PTTG1IP | Body | N_Shore | -5.55E-03 | 7.65E-06 |
| cg05541640 | ABHD14B;ABHD14B | TSS200;TSS200;TSS: | N_Shore | -9.36E-03 | 1.89E-08 |
| cg05547200 | NA | NA | S_Shore | 1.42E-02 | 1.61E-09 |
| cg05547260 | RSPH9 | TSS1500 | N_Shore | -1.71E-02 | 2.90E-12 |
| cg05547853 | MGAT1;MGAT1 | 5'UTR;5'UTR | N_Shore | -1.03E-02 | 1.22E-08 |
| cg05553502 | NR2F1 | Body | Island | 7.21E-03 | 3.80E-05 |
| cg05555111 | NA | NA | Island | 1.25E-02 | 6.27E-10 |
| cg05557932 | CREBBP;CREBBP | Body;Body | N_Shore | -1.08E-02 | 8.62E-11 |
| cg05561193 | DCLK2;DCLK2 | TSS1500;TSS1500 | N_Shore | -2.15E-02 | 1.99E-14 |
| cg05575054 | ADRB1 | 1stExon | Island | 1.77E-02 | 2.85E-09 |
| cg05575505 | NA | NA | NA | -1.05E-02 | 4.18E-07 |
| cg05576845 | PPP2R2B;PPP2R2B;I | Body;Body;Body;Bo | NA | -1.67E-02 | 3.98E-13 |
| cg05586607 | NA | NA | N_Shelf | -1.93E-02 | 2.53E-13 |
| cg05592292 | NA | NA | NA | -9.21E-03 | 1.44E-05 |
| cg05601974 | SMARCD3;SMARCD: | Body;Body;Body | N_Shore | -6.56E-03 | 1.43E-05 |
| cg05619598 | NA | NA | N_Shelf | -1.36E-02 | 2.98E-14 |
| cg05638174 | NA | NA | Island | 1.88E-02 | 1.71E-07 |
| cg05651778 | MRC2 | Body | Island | 2.40E-02 | 3.36E-06 |
| cg05655457 | OR5B17 | TSS1500 | NA | -2.03E-02 | 1.74E-12 |
| cg05657427 | LPPR5;LPPR5 | TSS1500;TSS1500 | Island | 1.46E-02 | 4.30E-05 |
| cg05657656 | NA | NA | NA | -1.17E-02 | 1.02E-05 |
| cg05658793 | DGCR6 | TSS1500 | Island | -7.54E-03 | 5.29E-06 |
| cg05663558 | MAG;MAG | TSS1500;TSS1500 | NA | -7.54E-03 | 6.91E-07 |
| cg05666120 | EGR4 | 1stExon | Island | 1.93E-02 | 3.72E-05 |
| cg05666820 | FZD9 | 1stExon | Island | 2.19E-02 | 2.05E-06 |
| cg05669853 | BEND3;BEND3 | 1stExon;5'UTR | N_Shore | -1.55E-02 | 6.58E-14 |
| cg05681670 | KCTD5 | Body | NA | -8.89E-03 | 2.99E-07 |
| cg05684266 | NA | NA | NA | -1.36E-02 | 6.93E-14 |
| cg05687719 | NA | NA | NA | -8.54E-03 | 5.95E-06 |
| cg05691543 | NA | NA | NA | -1.40E-02 | 6.57E-10 |
| cg05694021 | NA | NA | NA | -1.09E-02 | 1.70E-06 |
| cg05694250 | GPRIN2 | 5'UTR | S_Shelf | -7.54E-03 | 4.18E-06 |
| cg05697866 | NA | NA | S_Shore | -1.79E-02 | 8.32E-06 |
| cg05700079 | ZIC1 | TSS1500 | N_Shore | 9.29E-03 | 8.37E-06 |
| cg05705813 | ME3;ME3;ME3 | TSS1500;TSS200;TS: | S_Shore | -1.52E-02 | 2.53E-11 |
| cg05713794 | GP5 | 5'UTR | S_Shore | -2.58E-02 | 1.39E-14 |
| cg05714732 | NA | NA | N_Shore | -1.87E-02 | 1.28E-05 |
| cg05718255 | HMOX1 | 3'UTR | NA | -8.03E-03 | 6.17E-11 |
| cg05721773 | FAIM3;FAIM3;FAIM: | Body;Body;Body | S_Shore | -6.17E-03 | 7.87E-11 |
| cg05722931 | NA | NA | N_Shore | -1.37E-02 | 1.20E-09 |
| cg05724065 | PHKG1;PHKG1 | 1stExon;5'UTR | NA | -1.09E-02 | 1.37E-11 |
| cg05725703 | MYL3 | TSS1500 | NA | -9.10E-03 | 1.02E-10 |
| cg05733135 | BDNF;BDNF;BDNF;B | Body;5'UTR;5'UTR;5 | Island | 1.60E-02 | 2.91E-05 |
| cg05747555 | MARCH10;MARCH1 | 3'UTR;3'UTR | N_Shelf | -1.02E-02 | 2.63E-07 |
| cg05758434 | ZDBF2 | TSS200 | Island | 3.64E-02 | 6.83E-07 |
| cg05763097 | C14orf73 | Body | Island | 1.37E-02 | 3.85E-05 |

| | | | | | |
|---|---|---|---|---|---|
| cg05764628 | SPTBN4 | Body | Island | 9.23E-03 | 1.48E-10 |
| cg05767421 | MAL2 | Body | Island | 8.91E-03 | 1.40E-05 |
| cg05787556 | TLX3 | TSS1500 | Island | 6.31E-03 | 5.83E-05 |
| cg05790084 | NA | NA | S_Shelf | -9.07E-03 | 5.24E-05 |
| cg05797770 | EPHA2 | Body | NA | -1.36E-02 | 1.91E-08 |
| cg05798429 | C6orf27 | Body | NA | -5.76E-03 | 5.96E-05 |
| cg05802386 | SLC2A1 | Body | N_Shore | -1.50E-02 | 8.80E-15 |
| cg05809481 | ISG20L2 | Body | N_Shore | -1.15E-02 | 1.80E-11 |
| cg05810129 | FAM107B | Body | NA | -1.49E-02 | 1.20E-08 |
| cg05810428 | SLC14A1;SLC14A1;S | TSS1500;TSS1500;T | NA | -9.52E-03 | 1.80E-05 |
| cg05817709 | RARRES3 | TSS200 | NA | -1.45E-02 | 4.69E-09 |
| cg05818824 | SCN4A | TSS1500 | NA | -1.00E-02 | 1.65E-07 |
| cg05839709 | ZC3H12C | TSS1500 | N_Shore | -1.55E-02 | 3.83E-06 |
| cg05844247 | PAX1 | Body | N_Shore | 9.02E-03 | 6.84E-06 |
| cg05850205 | ADHFE1 | Body | N_Shore | -1.13E-02 | 2.92E-17 |
| cg05855039 | FLRT2;FLRT2 | 1stExon;5'UTR | Island | 2.80E-02 | 1.07E-10 |
| cg05860723 | MYF6 | Body | Island | 1.05E-02 | 2.58E-06 |
| cg05860978 | IL17B | TSS200 | NA | -7.33E-03 | 2.74E-05 |
| cg05862438 | TAS2R40 | 1stExon | NA | -6.53E-03 | 7.28E-05 |
| cg05863502 | CACNA1B | TSS1500 | Island | 1.23E-02 | 5.93E-05 |
| cg05864140 | RESP18 | Body | Island | 1.28E-02 | 5.77E-08 |
| cg05864261 | ABCG8;ABCG5 | TSS200;TSS200 | NA | -8.69E-03 | 6.93E-07 |
| cg05887421 | ATPBD4;ATPBD4 | TSS1500;TSS1500 | S_Shore | -1.32E-02 | 7.19E-12 |
| cg05892674 | C11orf70;C11orf70 | 1stExon;5'UTR | Island | 8.34E-03 | 1.67E-05 |
| cg05894970 | CDGAP | Body | N_Shore | -1.08E-02 | 1.10E-07 |
| cg05895545 | NA | NA | Island | 9.89E-03 | 7.58E-07 |
| cg05897963 | PLEKHF1 | 5'UTR | S_Shore | -8.07E-03 | 2.89E-05 |
| cg05898246 | HERC3;NAP1L5 | Body;TSS1500 | NA | -1.33E-02 | 1.48E-10 |
| cg05898524 | LMNA;LMNA;LMNA | TSS1500;TSS1500;T | N_Shore | -1.11E-02 | 1.49E-13 |
| cg05905988 | FAM155A | TSS1500 | Island | 1.12E-02 | 3.86E-06 |
| cg05915866 | ZFHX3 | 5'UTR | N_Shore | -1.68E-02 | 2.58E-07 |
| cg05923226 | CCDC105 | TSS200 | N_Shore | 1.31E-02 | 8.56E-12 |
| cg05925497 | FLJ32810 | Body | NA | -2.49E-02 | 2.19E-16 |
| cg05928053 | NA | NA | S_Shore | 1.24E-02 | 6.86E-05 |
| cg05933904 | SOX8 | 1stExon | Island | 2.63E-02 | 9.62E-10 |
| cg05934592 | NA | NA | NA | -9.85E-03 | 3.96E-07 |
| cg05936059 | EGFL7;EGFL7 | 5'UTR;5'UTR | Island | -8.13E-03 | 2.28E-05 |
| cg05937737 | SLC7A14 | 5'UTR | Island | 9.76E-03 | 5.17E-05 |
| cg05941376 | WWC1;WWC1;WW( | Body;Body;Body | NA | -1.38E-02 | 1.65E-09 |
| cg05941575 | MAPK4 | Body | Island | 1.08E-02 | 8.87E-05 |
| cg05942128 | HOXD11 | TSS200 | Island | 8.79E-03 | 2.93E-07 |
| cg05942459 | GRIK2;GRIK2;GRIK2 | TSS200;TSS200;TSS: | Island | 2.64E-02 | 1.50E-11 |
| cg05951860 | CTTNBP2 | Body | Island | 1.62E-02 | 2.52E-05 |
| cg05954192 | NA | NA | NA | -1.22E-02 | 3.53E-12 |
| cg05955036 | PLEKHO1 | Body | NA | 5.24E-03 | 9.04E-05 |
| cg05963604 | APPL1 | Body | S_Shore | -1.14E-02 | 8.14E-07 |
| cg05967403 | GSX1 | Body | Island | 9.66E-03 | 1.05E-07 |
| cg05972185 | ZFHX3;ZFHX3 | Body;Body | NA | -1.87E-02 | 3.13E-17 |
| cg05974498 | ZNF841 | TSS1500 | S_Shore | -1.22E-02 | 4.17E-08 |
| cg05979020 | HOXD10 | TSS200 | Island | 1.51E-02 | 1.90E-11 |

| | | | | | |
|---|---|---|---|---|---|
| cg05981033 | MTHFSD;MTHFSD;M | Body;Body;Body;Bo | N_Shore | -8.93E-03 | 1.64E-09 |
| cg05981335 | MYF6 | Body | Island | 6.46E-03 | 2.02E-05 |
| cg05991454 | NA | NA | Island | 2.69E-02 | 3.83E-15 |
| cg06002638 | ACACB | Body | N_Shore | -2.13E-02 | 9.40E-12 |
| cg06005169 | HOXD10 | 3'UTR | N_Shore | -3.59E-02 | 2.22E-05 |
| cg06005318 | TUBB | Body | S_Shore | -9.20E-03 | 7.93E-07 |
| cg06020207 | NA | NA | Island | 1.12E-02 | 5.78E-12 |
| cg06027843 | NA | NA | Island | 9.10E-03 | 6.95E-06 |
| cg06029095 | NA | NA | S_Shore | -8.09E-03 | 7.95E-07 |
| cg06029905 | INS-IGF2;IGF2;IGF2;I | Body;Body;Body;TS | S_Shore | -8.67E-03 | 5.36E-09 |
| cg06050495 | NA | NA | N_Shore | 8.22E-03 | 9.05E-06 |
| cg06056169 | CELSR1 | 1stExon | Island | 8.72E-03 | 3.12E-05 |
| cg06060874 | PRDM16;PRDM16 | Body;Body | NA | -1.23E-02 | 9.67E-07 |
| cg06077305 | PRSS22 | Body | Island | 3.05E-02 | 1.66E-05 |
| cg06082883 | DPEP1;DPEP1 | 5'UTR;TSS1500 | NA | -7.99E-03 | 4.68E-07 |
| cg06083330 | POU4F2 | Body | Island | 2.64E-02 | 1.57E-09 |
| cg06084210 | NA | NA | NA | -1.02E-02 | 1.50E-07 |
| cg06092815 | SPHKAP;SPHKAP | TSS200;TSS200 | Island | 7.41E-03 | 7.86E-05 |
| cg06097994 | C1QTNF1;C1QTNF1; | Body;Body;Body | N_Shore | -7.78E-03 | 8.67E-05 |
| cg06126421 | NA | NA | NA | -2.11E-02 | 1.15E-14 |
| cg06127256 | NA | NA | NA | -3.63E-02 | 2.18E-10 |
| cg06132069 | SLC12A5;SLC12A5 | Body;Body | Island | 8.38E-03 | 7.88E-06 |
| cg06155229 | PMPCB | TSS1500 | N_Shore | -1.31E-02 | 5.20E-05 |
| cg06161600 | BAIAP3 | Body | Island | -1.58E-02 | 3.71E-06 |
| cg06170683 | TRAP1 | Body | NA | -9.69E-03 | 1.26E-06 |
| cg06177599 | CLCN6 | Body | Island | -1.36E-02 | 1.47E-06 |
| cg06180869 | NA | NA | NA | -1.20E-02 | 9.39E-12 |
| cg06183244 | NA | NA | NA | -9.20E-03 | 6.62E-05 |
| cg06183338 | COL23A1 | Body | Island | 2.47E-02 | 6.96E-08 |
| cg06195193 | NA | NA | NA | -1.51E-02 | 9.93E-12 |
| cg06197616 | MYO7B | Body | N_Shore | -9.26E-03 | 5.63E-08 |
| cg06200092 | NA | NA | Island | 7.40E-03 | 7.57E-05 |
| cg06204735 | TMEM90A | TSS200 | Island | 1.63E-02 | 7.91E-05 |
| cg06207201 | SNX20;SNX20 | 3'UTR;3'UTR | NA | -5.28E-03 | 1.72E-06 |
| cg06217736 | DUSP8;HCCA2 | Body;Body | S_Shore | 1.10E-02 | 3.10E-05 |
| cg06223834 | ADCY9 | Body | NA | -6.35E-02 | 2.90E-08 |
| cg06239726 | FIGNL1;FIGNL1 | 5'UTR;5'UTR | N_Shore | -1.23E-02 | 9.29E-09 |
| cg06240854 | LIPT1;LIPT1;LIPT1;LI | TSS1500;TSS1500;T | N_Shore | -1.74E-02 | 3.85E-18 |
| cg06247406 | GRIK2;GRIK2;GRIK2 | TSS200;TSS200;TSS2 | Island | 1.18E-02 | 1.29E-06 |
| cg06272038 | ASCL2 | TSS1500 | Island | 3.12E-02 | 2.26E-06 |
| cg06275859 | NA | NA | Island | 1.25E-02 | 1.55E-07 |
| cg06279471 | TCIRG1 | TSS1500 | N_Shore | -9.05E-03 | 3.75E-10 |
| cg06285727 | ATG16L2 | TSS1500 | N_Shore | -8.29E-03 | 7.29E-06 |
| cg06291867 | HTR7;HTR7;HTR7 | 1stExon;1stExon;1s | Island | 8.90E-03 | 1.30E-06 |
| cg06294470 | TBC1D22B | Body | NA | -3.03E-02 | 4.40E-05 |
| cg06321596 | XYLT1 | Body | NA | -1.10E-02 | 1.13E-06 |
| cg06330558 | SCUBE2;SCUBE2 | Body;Body | NA | -1.17E-02 | 3.64E-09 |
| cg06335143 | ZYG11A | Body | Island | 1.57E-02 | 5.57E-18 |
| cg06346307 | COMT;COMT;COMT | 5'UTR;5'UTR;5'UTR; | NA | -6.12E-03 | 4.65E-05 |
| cg06353259 | NA | NA | S_Shore | -9.57E-03 | 1.32E-06 |

| | | | | | |
|---|---|---|---|---|---|
| cg06362313 | GAPDH | Body | S_Shore | -6.89E-03 | 5.42E-07 |
| cg06367459 | RPL28;RPL28;RPL28 | 3'UTR;Body;Body;B | S_Shore | -9.07E-03 | 8.12E-08 |
| cg06370069 | CHST3 | 5'UTR | S_Shelf | -1.33E-02 | 1.16E-13 |
| cg06390651 | NA | NA | N_Shore | 1.47E-02 | 5.37E-10 |
| cg06399164 | ZBTB47 | TSS200 | N_Shore | -5.95E-03 | 2.34E-07 |
| cg06400319 | SPRR2B | TSS1500 | NA | -2.10E-02 | 1.86E-14 |
| cg06401021 | HMGCLL1;HMGCLL1 | 1stExon;5'UTR;1stE | Island | 1.88E-02 | 1.96E-05 |
| cg06401614 | TSPAN18;TSPAN18 | Body;1stExon | NA | -5.41E-03 | 3.51E-05 |
| cg06405206 | NXPH3 | TSS1500 | Island | -5.50E-03 | 4.81E-05 |
| cg06413398 | DDO;DDO | TSS200;TSS200 | NA | -1.18E-02 | 2.65E-07 |
| cg06417752 | ATP5L | TSS1500 | N_Shore | -1.23E-02 | 5.68E-10 |
| cg06424065 | NA | NA | Island | 1.31E-02 | 1.05E-05 |
| cg06439941 | GNG11 | TSS1500 | NA | -1.66E-02 | 3.10E-09 |
| cg06440373 | TSPAN17;TSPAN17;T | 3'UTR;3'UTR;3'UTR | NA | -5.70E-03 | 2.33E-06 |
| cg06441398 | SHANK2;SHANK2 | 3'UTR;3'UTR | NA | -1.10E-02 | 3.56E-07 |
| cg06460691 | FAM169A | TSS1500 | S_Shore | -6.75E-03 | 1.46E-07 |
| cg06470626 | CDR2 | Body | NA | -8.25E-03 | 2.83E-05 |
| cg06474661 | NA | NA | S_Shore | -1.74E-02 | 1.38E-15 |
| cg06483432 | KIT;KIT | TSS1500;TSS1500 | N_Shore | -1.19E-02 | 2.80E-06 |
| cg06487082 | ZNF532;ZNF532 | 1stExon;5'UTR | N_Shore | -9.25E-03 | 4.10E-06 |
| cg06488443 | TBR1 | Body | Island | 9.64E-03 | 9.91E-06 |
| cg06492796 | NA | NA | N_Shore | -1.34E-02 | 1.89E-08 |
| cg06493994 | SCGN;SCGN | 1stExon;5'UTR | Island | 1.68E-02 | 6.47E-18 |
| cg06497848 | VAX1;VAX1 | Body;Body | Island | 7.18E-03 | 9.91E-06 |
| cg06498267 | HCN1 | 1stExon | Island | 8.01E-03 | 3.70E-05 |
| cg06520331 | LOC100188947;HEC | Body;Body;Body | S_Shore | -1.13E-02 | 4.85E-06 |
| cg06526721 | PRR16 | TSS1500 | N_Shore | -1.44E-02 | 3.83E-07 |
| cg06533408 | SLC35E3 | TSS1500 | N_Shore | -1.53E-02 | 4.23E-07 |
| cg06540876 | ZBTB12 | TSS1500 | S_Shore | -1.30E-02 | 2.13E-12 |
| cg06543221 | NA | NA | NA | -1.17E-02 | 9.43E-08 |
| cg06546202 | TSNARE1 | Body | NA | -5.12E-03 | 9.26E-05 |
| cg06552810 | NA | NA | NA | -2.61E-02 | 2.25E-12 |
| cg06558502 | NA | NA | Island | 1.08E-02 | 4.59E-11 |
| cg06567373 | NA | NA | N_Shore | 1.40E-02 | 9.78E-10 |
| cg06567855 | NA | NA | N_Shelf | -8.16E-03 | 9.51E-06 |
| cg06568783 | ITGB3 | 3'UTR | NA | -6.20E-03 | 2.91E-05 |
| cg06570224 | NA | NA | Island | 1.02E-02 | 3.79E-13 |
| cg06572160 | KCNC3 | 1stExon | Island | 2.08E-02 | 1.68E-08 |
| cg06578111 | HDAC11;HDAC11 | 3'UTR;3'UTR | NA | -7.29E-03 | 6.30E-07 |
| cg06594404 | NA | NA | Island | 1.44E-02 | 4.26E-06 |
| cg06599169 | RPL18AP3;SNORA68 | Body;TSS200;Body | S_Shelf | -5.74E-03 | 1.30E-05 |
| cg06601581 | NA | NA | N_Shelf | -9.37E-03 | 4.80E-08 |
| cg06602847 | DBC1;DBC1 | 5'UTR;1stExon | Island | 8.10E-03 | 4.54E-08 |
| cg06620896 | NA | NA | S_Shore | 3.23E-02 | 3.87E-08 |
| cg06621358 | CELSR3 | 1stExon | Island | 5.63E-03 | 7.89E-06 |
| cg06623151 | ASCL2 | TSS1500 | S_Shore | 7.39E-03 | 1.82E-05 |
| cg06629130 | NA | NA | Island | 2.42E-02 | 1.19E-07 |
| cg06633061 | NA | NA | Island | 6.49E-03 | 1.47E-05 |
| cg06639320 | FHL2;FHL2;FHL2;FHI | TSS200;TSS200;5'U | Island | 1.21E-02 | 3.95E-12 |
| cg06641153 | ST8SIA3 | Body | S_Shore | 1.77E-02 | 6.99E-17 |

| | | | | | |
|---|---|---|---|---|---|
| cg06645033 | NA | NA | Island | 1.11E-02 | 1.57E-05 |
| cg06655796 | NA | NA | Island | 8.77E-03 | 7.68E-06 |
| cg06661266 | NA | NA | N_Shelf | -1.78E-02 | 8.62E-17 |
| cg06664357 | DLL3;DLL3 | Body;Body | Island | 1.41E-02 | 1.08E-06 |
| cg06665453 | TSEN54;LLGL2;LLGL2 | 3'UTR;TSS1500;TSS1 | N_Shore | -4.09E-03 | 5.95E-05 |
| cg06666466 | NUB1 | Body | NA | -1.46E-02 | 1.88E-13 |
| cg06667761 | PCDH10;PCDH10 | 1stExon;1stExon | Island | 8.11E-03 | 8.44E-07 |
| cg06669752 | SIL1;SIL1 | TSS1500;TSS1500 | S_Shore | -1.49E-02 | 1.85E-08 |
| cg06673536 | CEACAM16 | 3'UTR | S_Shelf | 8.58E-03 | 4.77E-05 |
| cg06685111 | HCG18;TRIM39;TRIM | TSS1500;5'UTR;5'UT | S_Shore | -1.00E-02 | 1.17E-12 |
| cg06694734 | NA | NA | S_Shore | 1.93E-02 | 1.13E-07 |
| cg06700226 | NA | NA | NA | -6.57E-03 | 4.05E-05 |
| cg06700877 | PSD | Body | N_Shore | -7.04E-03 | 1.60E-06 |
| cg06707978 | ZIK1 | TSS1500 | Island | 1.65E-02 | 7.00E-26 |
| cg06708720 | ERC1;ERC1;ERC1;ER | TSS1500;TSS1500;T | N_Shore | -1.34E-02 | 1.84E-19 |
| cg06716182 | MAML2 | 1stExon | NA | -2.00E-02 | 2.65E-06 |
| cg06719900 | C11orf87;C11orf87 | 5'UTR;1stExon | N_Shore | 1.48E-02 | 3.98E-06 |
| cg06737494 | GHSR;GHSR | TSS1500;TSS1500 | Island | 1.27E-02 | 6.08E-07 |
| cg06757405 | PCDHGA4;PCDHGA5 | Body;Body;Body;Bo | Island | 6.40E-03 | 1.69E-05 |
| cg06761203 | 09-mar | TSS1500 | N_Shore | -8.94E-03 | 4.90E-09 |
| cg06762457 | ZC3H12D | TSS1500 | NA | -1.60E-02 | 7.13E-12 |
| cg06763054 | MTMR7;MTMR7 | 5'UTR;1stExon | Island | 1.62E-02 | 7.20E-05 |
| cg06769994 | NA | NA | NA | -8.34E-03 | 5.43E-05 |
| cg06774893 | DPEP3;DPEP3 | Body;Body | N_Shelf | -7.15E-03 | 8.03E-10 |
| cg06775930 | NA | NA | Island | 1.30E-02 | 4.33E-05 |
| cg06782035 | 11-mar | Body | Island | 2.51E-02 | 6.62E-15 |
| cg06783737 | NA | NA | NA | 3.03E-02 | 7.73E-13 |
| cg06784991 | ZYG11A | Body | Island | 1.82E-02 | 4.88E-19 |
| cg06785999 | SIX6;SIX6 | 1stExon;5'UTR | Island | 2.09E-02 | 2.20E-05 |
| cg06787724 | RASAL1 | Body | N_Shelf | -6.61E-03 | 1.11E-05 |
| cg06804433 | TFAP2D | Body | Island | 1.18E-02 | 2.86E-06 |
| cg06819357 | TECPR2 | Body | NA | -1.52E-02 | 8.68E-20 |
| cg06819923 | ZP2 | Body | NA | -1.45E-02 | 5.13E-14 |
| cg06825039 | SP8;SP8 | Body;Body | Island | 8.53E-03 | 1.16E-05 |
| cg06825166 | OPALIN;OPALIN;OPA | 1stExon;5'UTR;1stE | NA | -8.39E-03 | 1.25E-06 |
| cg06826457 | NA | NA | N_Shelf | -9.55E-03 | 1.40E-06 |
| cg06829299 | NA | NA | Island | 1.05E-02 | 5.47E-05 |
| cg06829686 | NA | NA | Island | 2.42E-02 | 7.45E-11 |
| cg06829788 | TRIM11 | 3'UTR | N_Shore | -1.80E-02 | 4.39E-12 |
| cg06832677 | NA | NA | NA | -1.77E-02 | 9.00E-13 |
| cg06833732 | CPLX2;CPLX2 | 5'UTR;5'UTR | Island | 1.33E-02 | 1.63E-06 |
| cg06834507 | C1orf106;C1orf106 | Body;Body | N_Shelf | -4.79E-03 | 7.26E-05 |
| cg06844526 | MYO1B;MYO1B;MYO | Body;Body;Body | NA | -2.51E-02 | 9.56E-08 |
| cg06853416 | NA | NA | S_Shore | -1.50E-02 | 1.26E-07 |
| cg06854015 | NA | NA | Island | 1.62E-02 | 7.77E-06 |
| cg06856687 | NA | NA | N_Shore | -1.07E-02 | 1.35E-07 |
| cg06861736 | MED15;MED15 | Body;Body | NA | -8.37E-03 | 1.84E-08 |
| cg06865451 | NA | NA | NA | 6.61E-03 | 1.72E-05 |
| cg06867863 | NA | NA | NA | -1.05E-02 | 2.80E-05 |
| cg06868473 | SHMT2;SHMT2;SHM | TSS1500;TSS1500;T | N_Shore | -1.09E-02 | 2.44E-08 |

| | | | | | |
|---|---|---|---|---|---|
| cg06868946 | XKR4 | 1stExon | S_Shore | 5.67E-03 | 2.69E-05 |
| cg06873452 | NA | NA | Island | 1.14E-02 | 4.36E-10 |
| cg06874016 | NKIRAS2;NKIRAS2;N | 3'UTR;3'UTR;3'UTR; | NA | -1.23E-02 | 2.23E-14 |
| cg06875162 | NA | NA | NA | -5.15E-03 | 6.61E-05 |
| cg06878361 | ZNF577;ZNF577;ZNF | Body;5'UTR;5'UTR | N_Shore | 1.00E-02 | 1.16E-09 |
| cg06881421 | NA | NA | NA | -1.27E-02 | 4.83E-20 |
| cg06882544 | NA | NA | Island | 6.76E-03 | 1.82E-10 |
| cg06885782 | KCNQ4;KCNQ4 | TSS1500;TSS1500 | N_Shore | -1.06E-02 | 2.64E-11 |
| cg06899799 | ANKRD52 | Body | N_Shore | -7.98E-03 | 1.79E-11 |
| cg06907680 | C14orf159;C14orf15 | TSS1500;TSS1500;T | NA | -9.91E-03 | 2.45E-07 |
| cg06911110 | CROCC | Body | S_Shore | -1.03E-02 | 5.77E-11 |
| cg06914505 | NA | NA | NA | -1.22E-02 | 8.56E-11 |
| cg06915667 | NA | NA | N_Shore | -7.56E-03 | 9.02E-05 |
| cg06928887 | ZFPM1 | Body | NA | -9.12E-03 | 5.96E-06 |
| cg06929324 | C14orf115 | TSS1500 | NA | -6.92E-03 | 1.41E-07 |
| cg06931612 | NA | NA | S_Shore | -6.89E-03 | 7.81E-12 |
| cg06933824 | NEURL1B | Body | Island | 2.40E-02 | 8.96E-14 |
| cg06934523 | KIAA0247 | TSS1500 | N_Shore | -9.26E-03 | 4.10E-09 |
| cg06938264 | PCDHA6;PCDHA2;PC | Body;Body;Body;1s | Island | 1.29E-02 | 8.88E-06 |
| cg06942685 | ZNF542;ZNF542;ZNF | TSS1500;TSS1500;T | Island | 7.43E-03 | 1.46E-12 |
| cg06942701 | TBR1 | Body | Island | 1.21E-02 | 3.55E-06 |
| cg06951627 | NA | NA | N_Shore | -1.74E-02 | 8.93E-14 |
| cg06953773 | LOC134466 | TSS1500 | S_Shore | 9.16E-03 | 7.21E-05 |
| cg06963109 | SYT7 | Body | N_Shore | 7.06E-03 | 5.34E-05 |
| cg06966811 | OTX2 | 5'UTR | Island | 1.58E-02 | 1.35E-13 |
| cg06971092 | NA | NA | N_Shore | 7.48E-03 | 2.88E-05 |
| cg06976250 | ANKK1 | 1stExon | Island | 1.92E-02 | 1.10E-05 |
| cg06991495 | CSNK1E;CSNK1E | TSS1500;TSS1500 | S_Shore | -5.02E-03 | 2.82E-05 |
| cg07003407 | LOC284837 | TSS200 | NA | -8.36E-03 | 4.96E-07 |
| cg07006564 | C2CD4A | Body | Island | 9.33E-03 | 3.63E-09 |
| cg07009376 | NA | NA | NA | 2.79E-02 | 4.96E-06 |
| cg07012926 | GLRA1;GLRA1 | TSS200;TSS200 | Island | 1.05E-02 | 1.99E-05 |
| cg07022048 | KRT7 | Body | N_Shore | -9.26E-03 | 1.43E-13 |
| cg07027613 | C1RL;LOC283314 | Body;TSS1500 | NA | -1.18E-02 | 1.64E-09 |
| cg07028914 | TBX18 | TSS200 | Island | 2.29E-02 | 7.65E-07 |
| cg07029287 | PRDM16;PRDM16 | Body;Body | NA | -9.01E-03 | 7.01E-05 |
| cg07029873 | ESRRG;ESRRG | 1stExon;5'UTR | Island | 1.55E-02 | 8.10E-08 |
| cg07033624 | NA | NA | N_Shore | 8.34E-03 | 5.16E-05 |
| cg07057579 | NA | NA | Island | 1.41E-02 | 8.33E-17 |
| cg07060551 | SHANK1 | Body | Island | 1.58E-02 | 4.82E-12 |
| cg07062262 | CLPB | Body | N_Shelf | -8.22E-03 | 2.24E-05 |
| cg07063351 | SPTBN4;SPTBN4 | Body;Body | Island | 1.36E-02 | 1.95E-07 |
| cg07065666 | RPS6KA2;RPS6KA2 | Body;Body | S_Shelf | -1.30E-02 | 4.50E-10 |
| cg07067710 | NA | NA | S_Shore | -5.52E-03 | 2.95E-05 |
| cg07068674 | NA | NA | N_Shore | 1.57E-02 | 2.80E-08 |
| cg07071449 | RAMP1 | Body | NA | -2.25E-02 | 6.99E-13 |
| cg07071764 | FAT3 | Body | NA | -2.73E-02 | 1.13E-05 |
| cg07073120 | DCXR | Body | Island | -8.15E-03 | 1.52E-06 |
| cg07077735 | FUK | 5'UTR | NA | -1.44E-02 | 3.22E-05 |
| cg07080372 | SLC25A22 | TSS1500 | N_Shore | -1.74E-02 | 4.64E-19 |

| | | | | | |
|---|---|---|---|---|---|
| cg07082267 | NA | NA | NA | -7.50E-03 | 1.57E-16 |
| cg07098391 | KDR | 1stExon | Island | 9.12E-03 | 4.72E-07 |
| cg07099331 | NA | NA | Island | 1.41E-02 | 1.52E-11 |
| cg07101980 | NDUFC2 | 3'UTR | NA | -1.90E-02 | 1.48E-25 |
| cg07103129 | TFAP2B | Body | Island | 1.67E-02 | 1.02E-06 |
| cg07105285 | MTNR1B | TSS200 | N_Shore | 1.05E-02 | 4.06E-05 |
| cg07115542 | NKX2-1;NKX2-1 | Body;1stExon | Island | 2.03E-02 | 1.25E-05 |
| cg07115626 | NA | NA | S_Shelf | 1.58E-02 | 1.09E-08 |
| cg07116631 | NA | NA | Island | 2.01E-02 | 2.37E-07 |
| cg07125666 | NA | NA | NA | -2.61E-02 | 2.79E-19 |
| cg07126263 | CCBE1 | TSS1500 | Island | 2.88E-02 | 2.42E-06 |
| cg07127410 | ZNRF3 | Body | S_Shore | -7.98E-03 | 6.37E-06 |
| cg07131210 | CYP2W1 | Body | N_Shore | -8.34E-03 | 7.50E-06 |
| cg07131742 | B3GNT1;BRMS1;BRI | 3'UTR;TSS1500;TSS: | N_Shore | -1.07E-02 | 1.28E-08 |
| cg07132038 | PRKAG2 | TSS1500 | S_Shore | -8.33E-03 | 9.99E-07 |
| cg07134316 | NAB2 | Body | NA | -1.48E-02 | 9.70E-08 |
| cg07146984 | NA | NA | S_Shelf | -4.45E-02 | 6.86E-09 |
| cg07148207 | NA | NA | NA | -8.97E-03 | 1.83E-05 |
| cg07149296 | KIAA1755 | TSS1500 | Island | 1.87E-02 | 3.88E-07 |
| cg07149609 | NA | NA | Island | 2.20E-02 | 1.70E-08 |
| cg07150045 | KCNH7;KCNH7 | TSS1500;TSS1500 | NA | 7.18E-03 | 4.65E-05 |
| cg07152216 | NA | NA | N_Shore | 1.83E-02 | 6.08E-08 |
| cg07158339 | FXN;FXN;FXN | TSS1500;TSS1500;T | N_Shore | -1.62E-02 | 2.76E-11 |
| cg07164639 | DDO;DDO | TSS1500;TSS1500 | NA | -1.30E-02 | 5.64E-09 |
| cg07169873 | MIR1253 | TSS1500 | Island | 8.63E-03 | 2.96E-07 |
| cg07171111 | NA | NA | Island | 2.58E-02 | 4.37E-07 |
| cg07178825 | TP73;TP73;TP73;TP7 | Body;Body;3'UTR;3' | Island | 8.78E-03 | 3.73E-06 |
| cg07181952 | ACAP3 | Body | N_Shelf | -8.46E-03 | 3.11E-11 |
| cg07186576 | NA | NA | N_Shore | -7.90E-03 | 2.91E-07 |
| cg07191594 | SLC12A5 | TSS200 | N_Shore | 5.66E-03 | 2.55E-05 |
| cg07191791 | SUSD4;SUSD4 | Body;Body | NA | -7.24E-03 | 8.95E-05 |
| cg07193553 | DIO3 | TSS1500 | Island | 1.28E-02 | 1.34E-05 |
| cg07197831 | DNAJC5G | 3'UTR | NA | -1.63E-02 | 1.60E-11 |
| cg07203024 | OBSCN | Body | S_Shore | -8.68E-03 | 1.12E-05 |
| cg07206442 | NA | NA | N_Shelf | -8.90E-03 | 6.24E-06 |
| cg07211259 | PDCD1LG2 | TSS200 | NA | -1.01E-02 | 3.90E-05 |
| cg07230786 | RPH3A;RPH3A | 3'UTR;3'UTR | NA | -9.00E-03 | 9.20E-09 |
| cg07231955 | NA | NA | Island | 2.27E-02 | 8.20E-09 |
| cg07234388 | TMEM79;TMEM79 | Body;3'UTR | S_Shore | -1.16E-02 | 3.51E-13 |
| cg07236840 | DIP2C | Body | Island | 1.07E-02 | 3.98E-05 |
| cg07237326 | NA | NA | Island | 1.11E-02 | 1.70E-06 |
| cg07240873 | PCDHA6;PCDHA2;P( | Body;Body;Body;1s' | Island | 6.18E-03 | 1.32E-05 |
| cg07249459 | FOXC2 | TSS1500 | Island | 1.47E-02 | 1.46E-05 |
| cg07252200 | NA | NA | Island | 1.82E-02 | 4.96E-05 |
| cg07254032 | HCN1 | 1stExon | Island | 6.49E-03 | 2.18E-08 |
| cg07258474 | NTSR2 | 1stExon | Island | 8.44E-03 | 5.33E-06 |
| cg07262873 | SLC38A9 | Body | NA | -1.74E-02 | 6.33E-06 |
| cg07271264 | MYOD1 | TSS200 | Island | 7.97E-03 | 3.17E-06 |
| cg07279955 | NA | NA | Island | 7.43E-03 | 4.18E-06 |
| cg07280206 | BBS2 | TSS1500 | S_Shore | -1.12E-02 | 3.58E-05 |

| | | | | | |
|---|---|---|---|---|---|
| cg07281879 | WIT1 | Body | Island | 2.39E-02 | 6.11E-08 |
| cg07292140 | NA | NA | NA | -1.58E-02 | 1.24E-06 |
| cg07297777 | ITGA7;ITGA7;ITGA7 | Body;Body;Body | N_Shelf | -5.40E-03 | 4.49E-05 |
| cg07310671 | NA | NA | NA | -1.80E-02 | 4.04E-09 |
| cg07312601 | MRTO4 | Body | NA | -9.10E-03 | 1.26E-08 |
| cg07313319 | CSNK1E;CSNK1E | TSS1500;TSS1500 | S_Shore | -1.18E-02 | 9.07E-13 |
| cg07318398 | KLHL10;NT5C3L | TSS1500;TSS1500 | S_Shore | -1.21E-02 | 1.96E-11 |
| cg07321467 | BMP3 | Body | NA | -1.55E-02 | 1.36E-05 |
| cg07323825 | NA | NA | N_Shore | 1.75E-02 | 2.43E-09 |
| cg07326413 | NA | NA | NA | -1.25E-02 | 6.45E-09 |
| cg07330212 | PCDHA2;PCDHA1;P( | Body;Body;Body;Bo | Island | 1.45E-02 | 1.56E-06 |
| cg07330489 | NA | NA | NA | -1.39E-02 | 4.12E-16 |
| cg07333191 | NA | NA | Island | 1.03E-02 | 9.60E-10 |
| cg07336350 | NA | NA | Island | 1.56E-02 | 2.87E-09 |
| cg07341290 | NA | NA | NA | 1.68E-02 | 1.03E-05 |
| cg07349899 | GALNTL4 | Body | NA | -9.56E-03 | 9.22E-12 |
| cg07362800 | NA | NA | NA | -9.44E-03 | 1.43E-07 |
| cg07364657 | NA | NA | NA | -1.69E-02 | 4.51E-10 |
| cg07365960 | GRIN2C | Body | Island | 1.58E-02 | 9.95E-06 |
| cg07367302 | NA | NA | N_Shelf | -4.23E-03 | 2.45E-05 |
| cg07369190 | PCP4L1 | Body | Island | 9.63E-03 | 1.07E-05 |
| cg07375851 | PGLYRP2 | TSS1500 | NA | -1.24E-02 | 1.35E-11 |
| cg07377098 | CSNK2A2 | Body | N_Shore | -1.50E-02 | 1.14E-19 |
| cg07381930 | GOLGA7B | TSS1500 | N_Shore | -8.20E-03 | 8.18E-06 |
| cg07382132 | NA | NA | N_Shore | -1.33E-02 | 1.86E-09 |
| cg07387591 | PCSK2 | Body | Island | 1.00E-02 | 1.15E-05 |
| cg07388347 | NA | NA | N_Shelf | 1.09E-02 | 1.79E-06 |
| cg07388493 | NDUFS5 | TSS1500 | NA | -2.51E-02 | 7.82E-27 |
| cg07394446 | ADAMTS17 | Body | Island | 1.64E-02 | 1.54E-05 |
| cg07397616 | SH3BP1 | TSS1500 | N_Shore | -8.50E-03 | 4.26E-09 |
| cg07409200 | C13orf30 | TSS1500 | NA | -9.42E-03 | 2.03E-05 |
| cg07416237 | CLYBL | 3'UTR | Island | 1.61E-02 | 4.76E-12 |
| cg07416733 | RHOD | Body | NA | -6.86E-03 | 6.63E-08 |
| cg07418114 | NTRK1;NTRK1;NTRK | Body;Body;Body | NA | -8.45E-03 | 2.49E-08 |
| cg07422880 | LASS2;LASS2 | 5'UTR;5'UTR | N_Shore | -7.85E-03 | 4.22E-07 |
| cg07433178 | SYNPO;SYNPO | Body;Body | NA | -8.79E-03 | 1.37E-07 |
| cg07437546 | NA | NA | NA | -1.78E-02 | 5.60E-20 |
| cg07442409 | NA | NA | Island | 9.74E-03 | 3.42E-05 |
| cg07457252 | NA | NA | N_Shore | -8.01E-03 | 7.70E-08 |
| cg07462292 | ALX4 | TSS1500 | Island | 6.28E-03 | 1.55E-07 |
| cg07462756 | MSX1 | TSS1500 | Island | 1.09E-02 | 6.17E-08 |
| cg07463541 | PCDHA2;PCDHA1;P( | Body;Body;1stExon, | Island | 1.60E-02 | 9.32E-07 |
| cg07464571 | LEP | TSS1500 | Island | 9.45E-03 | 2.01E-05 |
| cg07485775 | NEURL1B | Body | Island | 2.35E-02 | 6.32E-18 |
| cg07488141 | TNS3 | 5'UTR | NA | -6.74E-03 | 2.30E-07 |
| cg07489048 | FOXG1 | 1stExon | Island | 1.85E-02 | 2.60E-10 |
| cg07495704 | NA | NA | N_Shelf | -6.55E-03 | 6.75E-08 |
| cg07502389 | NEFM;NEFM | TSS200;TSS1500 | Island | 1.63E-02 | 2.37E-13 |
| cg07506510 | PHLDA2 | TSS1500 | S_Shore | -7.90E-03 | 7.66E-07 |
| cg07511014 | PAOX;PAOX;PAOX | TSS1500;TSS1500;T | Island | -1.26E-02 | 3.18E-05 |

| | | | | | |
|---|---|---|---|---|---|
| cg07521929 | SLC4A9 | Body | N_Shore | -1.11E-02 | 4.83E-06 |
| cg07529654 | TGIF1;TGIF1 | 5'UTR;TSS1500 | N_Shore | -3.50E-02 | 1.70E-07 |
| cg07530172 | NA | NA | NA | -8.27E-03 | 2.37E-07 |
| cg07539798 | PCDHGA4;PCDHGA1 | Body;Body;Body;Bo | Island | 1.11E-02 | 1.39E-05 |
| cg07541160 | FGD2 | Body | NA | -9.40E-03 | 9.88E-10 |
| cg07544187 | CILP2 | Body | Island | 2.97E-02 | 3.30E-08 |
| cg07547549 | SLC12A5;SLC12A5 | Body;Body | Island | 1.49E-02 | 4.89E-21 |
| cg07553761 | TRIM59 | TSS1500 | Island | 2.45E-02 | 3.62E-32 |
| cg07554478 | ATP6V1A | 5'UTR | NA | -2.99E-02 | 9.37E-05 |
| cg07557423 | ATP8A1;ATP8A1 | Body;Body | N_Shore | -1.18E-02 | 2.87E-05 |
| cg07560948 | KCNQ4;KCNQ4 | Body;Body | N_Shelf | -6.70E-03 | 9.66E-06 |
| cg07565551 | OSBPL6 | 5'UTR | S_Shelf | -2.03E-02 | 5.81E-19 |
| cg07568296 | MAD1L1;MAD1L1;M | Body;Body;Body | N_Shore | -9.46E-03 | 1.76E-06 |
| cg07568430 | CSF1;CSF1;CSF1;CSF | TSS1500;TSS1500;T | N_Shore | -1.33E-02 | 1.17E-13 |
| cg07568841 | ZNRF2 | Body | NA | -2.24E-02 | 5.19E-05 |
| cg07578695 | SLC34A2 | TSS200 | Island | 1.37E-02 | 3.31E-05 |
| cg07581732 | KCNJ6 | TSS200 | Island | 1.82E-02 | 1.33E-08 |
| cg07583137 | CHMP4C | TSS1500 | N_Shore | -2.14E-02 | 1.73E-15 |
| cg07589968 | KCNS1 | TSS200 | S_Shore | -5.44E-03 | 1.57E-08 |
| cg07599133 | LOC643719 | TSS200 | Island | 1.56E-02 | 3.91E-05 |
| cg07601967 | PAK7;PAK7 | Body;Body | NA | -1.37E-02 | 6.54E-10 |
| cg07612562 | CNTNAP2;CNTNAP2 | 1stExon;5'UTR | Island | 1.76E-02 | 7.43E-08 |
| cg07622493 | NA | NA | Island | 1.19E-02 | 9.65E-08 |
| cg07629125 | PCDH17 | Body | NA | -1.28E-02 | 2.73E-10 |
| cg07639287 | NA | NA | S_Shore | 1.03E-02 | 8.71E-11 |
| cg07640648 | DLL3;DLL3 | Body;Body | Island | 1.80E-02 | 4.72E-05 |
| cg07642566 | MIR7-3;C19orf30 | TSS1500;Body | NA | 1.20E-02 | 5.28E-05 |
| cg07650504 | COL2A1;COL2A1 | Body;Body | N_Shore | 9.78E-03 | 1.27E-09 |
| cg07668993 | SLPI | TSS200 | NA | -9.12E-03 | 3.91E-06 |
| cg07676002 | CDH22;CDH22 | 1stExon;5'UTR | Island | 7.32E-03 | 4.83E-05 |
| cg07677446 | PADI6 | Body | NA | -9.37E-03 | 3.83E-10 |
| cg07682515 | NA | NA | S_Shelf | -1.22E-02 | 4.53E-09 |
| cg07698523 | NA | NA | NA | 5.30E-03 | 8.64E-05 |
| cg07698783 | SPI1;SPI1 | Body;Body | N_Shelf | -5.31E-03 | 1.97E-05 |
| cg07703358 | NA | NA | NA | -9.86E-03 | 3.39E-05 |
| cg07706352 | COL5A3 | Body | Island | 7.11E-03 | 5.55E-05 |
| cg07707641 | CST4 | TSS1500 | NA | -8.25E-03 | 5.21E-07 |
| cg07716052 | WT1;WT1;WT1;WT1 | Body;Body;Body;Bo | N_Shore | 8.85E-03 | 1.81E-09 |
| cg07717903 | PCDHGA4;PCDHGA1 | Body;Body;TSS200;I | N_Shore | 1.28E-02 | 8.51E-07 |
| cg07722360 | BANF2;BANF2;BANF | 5'UTR;5'UTR;Body | NA | -2.08E-02 | 9.53E-12 |
| cg07724670 | NA | NA | Island | 1.08E-02 | 9.92E-05 |
| cg07737781 | MLXIPL;MLXIPL;MLX | 1stExon;1stExon;5'U | Island | 9.88E-03 | 8.26E-07 |
| cg07739205 | SNTG1 | TSS1500 | S_Shore | 2.42E-02 | 1.08E-07 |
| cg07739478 | C6orf25;C6orf25;C6 | Body;Body;Body;Bo | S_Shore | -1.18E-02 | 4.48E-05 |
| cg07743805 | NA | NA | S_Shore | -7.05E-03 | 1.08E-06 |
| cg07747220 | OXT | TSS200 | Island | -1.77E-02 | 3.32E-07 |
| cg07748854 | NA | NA | Island | -1.10E-02 | 7.07E-07 |
| cg07750560 | NA | NA | S_Shore | 8.79E-03 | 4.35E-05 |
| cg07778983 | ZNF582 | TSS1500 | S_Shore | 8.70E-03 | 6.65E-05 |
| cg07788865 | NA | NA | NA | -7.39E-03 | 9.02E-05 |

| | | | | | |
|---|---|---|---|---|---|
| cg07795325 | CAMK2D;CAMK2D;C | Body;Body;Body;Bo | NA | -7.30E-03 | 7.91E-09 |
| cg07804076 | PCDHGA4;PCDHGA2 | Body;Body;1stExon; | Island | 1.21E-02 | 2.04E-08 |
| cg07806886 | STXBP5L | TSS200 | Island | 2.56E-02 | 9.08E-17 |
| cg07810884 | TPM4 | TSS1500 | N_Shore | -1.45E-02 | 7.46E-08 |
| cg07820189 | NA | NA | NA | -2.00E-02 | 9.34E-21 |
| cg07824177 | ISCA1 | Body | N_Shore | -1.08E-02 | 3.93E-08 |
| cg07826255 | SGCA;SGCA | Body;Body | NA | -1.10E-02 | 1.12E-09 |
| cg07839457 | NLRC5 | TSS1500 | N_Shore | -2.45E-02 | 5.63E-12 |
| cg07839627 | GABBR1;GABBR1 | Body;Body | N_Shore | -4.64E-03 | 2.61E-05 |
| cg07842403 | EPHB1 | Body | Island | 1.03E-02 | 4.36E-08 |
| cg07850527 | NA | NA | Island | 1.96E-02 | 4.31E-05 |
| cg07852825 | GHSR;GHSR | 1stExon;1stExon | Island | 8.02E-03 | 2.78E-06 |
| cg07858728 | DMBT1;DMBT1;DM | TSS1500;TSS1500;T | NA | -8.23E-03 | 3.09E-05 |
| cg07858908 | NA | NA | Island | 6.74E-03 | 9.08E-05 |
| cg07861564 | HSPB8;HSPB8 | 5'UTR;1stExon | NA | 9.67E-03 | 1.89E-06 |
| cg07861790 | NA | NA | S_Shore | 1.11E-02 | 5.50E-05 |
| cg07862554 | MME;MME;MME;M | TSS1500;TSS1500;T | N_Shore | 1.14E-02 | 1.99E-07 |
| cg07869023 | PCSK2 | Body | S_Shelf | -1.52E-02 | 6.67E-10 |
| cg07870999 | NA | NA | Island | 1.23E-02 | 7.60E-05 |
| cg07871633 | NA | NA | N_Shore | -1.05E-02 | 4.91E-10 |
| cg07892422 | HOXC13 | 1stExon | Island | 8.46E-03 | 3.05E-07 |
| cg07899681 | NA | NA | Island | 8.82E-03 | 1.92E-07 |
| cg07902673 | LMO4 | TSS1500 | N_Shore | -2.31E-02 | 2.28E-05 |
| cg07906193 | NA | NA | NA | -1.86E-02 | 4.63E-11 |
| cg07910488 | MYT1L | 5'UTR | NA | -7.50E-03 | 1.32E-06 |
| cg07912789 | PRMT8 | TSS200 | Island | 1.70E-02 | 2.24E-07 |
| cg07914457 | NR0B2 | TSS1500 | NA | -6.28E-03 | 3.54E-08 |
| cg07915206 | AVEN;CHRM5 | Body;TSS1500 | NA | -1.23E-02 | 1.06E-09 |
| cg07922154 | ARG2 | Body | S_Shore | -9.61E-03 | 5.97E-11 |
| cg07924380 | NA | NA | NA | -1.19E-02 | 2.69E-07 |
| cg07925587 | KRT80;KRT80 | Body;Body | NA | -8.95E-03 | 2.55E-06 |
| cg07927379 | C7orf13;RNF32 | Body;TSS1500 | Island | 3.30E-02 | 1.26E-10 |
| cg07928642 | SGK269 | 3'UTR | NA | -1.75E-02 | 3.10E-11 |
| cg07931391 | NA | NA | Island | 2.41E-02 | 2.67E-06 |
| cg07931844 | NR2E3;NR2E3 | TSS1500;TSS1500 | N_Shore | -8.07E-03 | 1.06E-07 |
| cg07941108 | NA | NA | NA | -1.47E-02 | 1.93E-12 |
| cg07948143 | NA | NA | NA | 8.47E-03 | 5.15E-05 |
| cg07949989 | NA | NA | NA | 7.91E-03 | 1.29E-07 |
| cg07950397 | OLFM2 | Body | N_Shore | -5.44E-03 | 2.86E-05 |
| cg07951083 | NA | NA | NA | 1.07E-02 | 2.09E-06 |
| cg07952813 | LOC100271715;LOC | Body;TSS1500 | Island | 9.66E-03 | 5.09E-05 |
| cg07955995 | KLF14 | TSS1500 | Island | 4.09E-02 | 1.22E-18 |
| cg07960624 | SAMD12 | 3'UTR | NA | -1.03E-02 | 9.77E-06 |
| cg07962315 | OSTF1;C9orf95;C9or | Body;TSS1500;TSS1 | S_Shore | -1.56E-02 | 1.01E-06 |
| cg07964833 | FCAR;FCAR;FCAR;FC | Body;Body;Body;Bo | NA | 1.92E-02 | 6.03E-11 |
| cg07967679 | KRT7 | TSS200 | Island | 9.46E-03 | 3.56E-06 |
| cg07972458 | NA | NA | NA | -1.34E-02 | 4.05E-07 |
| cg07972954 | TUBB | Body | S_Shore | -7.85E-03 | 7.89E-06 |
| cg07979752 | CUL5 | TSS200 | N_Shore | -1.24E-02 | 5.50E-07 |
| cg07986943 | NA | NA | Island | 7.46E-03 | 1.22E-05 |

| | | | | | |
|---|---|---|---|---|---|
| cg07987449 | NA | NA | S_Shelf | -6.76E-03 | 1.85E-09 |
| cg07989568 | TEKT1 | TSS200 | NA | 1.06E-02 | 6.65E-09 |
| cg08021945 | NA | NA | N_Shore | -1.80E-02 | 2.68E-10 |
| cg08022502 | UNC45A;UNC45A | Body;Body | S_Shore | -7.09E-03 | 1.73E-05 |
| cg08023265 | NA | NA | Island | 5.80E-03 | 1.03E-06 |
| cg08024029 | VWA5B1 | Body | Island | 8.02E-03 | 2.45E-06 |
| cg08065657 | HAS1 | Body | Island | 1.06E-02 | 2.66E-06 |
| cg08065733 | INHBB | Body | N_Shore | 1.18E-02 | 6.79E-07 |
| cg08081424 | NA | NA | NA | -7.94E-03 | 3.63E-06 |
| cg08084860 | NA | NA | Island | 1.25E-02 | 9.48E-07 |
| cg08090640 | IFI35 | Body | NA | -1.78E-02 | 3.30E-23 |
| cg08097417 | KLF14 | TSS1500 | Island | 2.00E-02 | 2.23E-22 |
| cg08101174 | NA | NA | NA | -9.21E-03 | 1.37E-08 |
| cg08105529 | HFM1 | Body | NA | 5.54E-03 | 2.50E-10 |
| cg08116915 | CPZ;CPZ;CPZ | 5'UTR;Body;Body | S_Shelf | -1.60E-02 | 3.60E-17 |
| cg08119452 | OLFM4 | TSS1500 | NA | -1.59E-02 | 7.21E-10 |
| cg08122899 | NA | NA | NA | -8.75E-03 | 2.33E-09 |
| cg08144042 | NA | NA | N_Shelf | -1.90E-02 | 3.20E-07 |
| cg08146256 | HNRNPC;HNRNPC;H | 5'UTR;5'UTR;5'UTR; | N_Shelf | -1.40E-02 | 3.68E-06 |
| cg08146483 | LHX8 | Body | Island | 1.67E-02 | 2.26E-05 |
| cg08149193 | ALX4 | TSS1500 | S_Shore | 1.66E-02 | 2.41E-06 |
| cg08153621 | NA | NA | Island | 1.51E-02 | 7.27E-09 |
| cg08154348 | RIPPLY2 | TSS200 | Island | 3.03E-02 | 2.56E-05 |
| cg08157575 | NA | NA | NA | -1.10E-02 | 1.44E-07 |
| cg08160331 | KLHL35 | 1stExon | Island | 1.14E-02 | 2.75E-15 |
| cg08161931 | PLEC1;PLEC1;PLEC1 | TSS1500;Body;Body | S_Shore | -7.48E-03 | 3.98E-07 |
| cg08162372 | BMP4;BMP4 | 5'UTR;5'UTR | Island | 2.50E-02 | 2.03E-06 |
| cg08164233 | RFXAP | Body | S_Shore | -1.38E-02 | 6.91E-10 |
| cg08169949 | RPA2 | TSS1500 | Island | 2.89E-02 | 4.69E-14 |
| cg08179817 | NA | NA | NA | -1.48E-02 | 3.19E-12 |
| cg08182454 | NA | NA | S_Shelf | -1.73E-02 | 1.31E-07 |
| cg08199758 | CLDN9 | TSS200 | N_Shore | -1.46E-02 | 7.32E-05 |
| cg08200404 | C2CD4A | 5'UTR | N_Shore | -9.64E-03 | 9.14E-08 |
| cg08209133 | SLC10A4 | 1stExon | Island | 8.99E-03 | 1.02E-06 |
| cg08212545 | NA | NA | S_Shelf | -1.04E-02 | 5.35E-07 |
| cg08217285 | GIT1;GIT1 | TSS1500;TSS1500 | N_Shore | -1.33E-02 | 8.89E-13 |
| cg08220649 | GPR85;GPR85;GPR8 | TSS1500;5'UTR;TSS: | NA | 8.71E-03 | 8.00E-06 |
| cg08224159 | AKAP12;AKAP12 | TSS1500;Body | N_Shore | -1.33E-02 | 1.58E-10 |
| cg08225986 | PATZ1;PATZ1;PATZ1 | Body;Body;Body;Bo | N_Shelf | -8.38E-03 | 2.56E-08 |
| cg08231709 | KCNS2 | TSS1500 | Island | 1.09E-02 | 3.18E-11 |
| cg08231710 | MMP23A;MMP23B | TSS1500;TSS1500 | Island | 1.32E-02 | 2.25E-06 |
| cg08231730 | OTUB1;OTUB1 | TSS200;TSS200 | N_Shore | -1.12E-02 | 7.80E-06 |
| cg08234504 | NA | NA | N_Shelf | -1.20E-02 | 2.40E-25 |
| cg08235161 | NA | NA | Island | 1.23E-02 | 2.02E-06 |
| cg08239858 | NA | NA | Island | 1.32E-02 | 4.75E-08 |
| cg08247806 | NA | NA | S_Shelf | -6.89E-03 | 5.63E-05 |
| cg08248579 | KIF13A;KIF13A;KIF1: | TSS1500;TSS1500;T | S_Shore | -2.63E-02 | 1.14E-13 |
| cg08248751 | NA | NA | S_Shore | -5.13E-03 | 3.43E-06 |
| cg08255475 | CDT1 | Body | N_Shore | -1.82E-02 | 2.45E-14 |
| cg08262002 | LDB2;LDB2 | Body;Body | NA | -2.21E-02 | 2.04E-38 |

| | | | | | |
|---|---|---|---|---|---|
| cg08262220 | PRDM16;PRDM16 | Body;Body | N_Shore | -8.94E-03 | 1.53E-06 |
| cg08267148 | CFLAR;CFLAR | Body;Body | NA | -1.55E-02 | 3.05E-18 |
| cg08274011 | CPAMD8 | TSS1500 | NA | -1.02E-02 | 1.20E-06 |
| cg08277369 | KIAA1609 | TSS1500 | Island | -2.25E-02 | 1.81E-10 |
| cg08280468 | SHISA9;SHISA9 | Body;Body | Island | 7.19E-03 | 2.37E-07 |
| cg08285646 | NA | NA | NA | -1.48E-02 | 9.40E-10 |
| cg08286181 | NA | NA | N_Shore | 1.24E-02 | 1.97E-07 |
| cg08288130 | DOK2 | TSS1500 | S_Shore | -1.01E-02 | 3.98E-05 |
| cg08288223 | SHANK2 | Body | N_Shore | -1.08E-02 | 4.16E-08 |
| cg08288811 | PCDHA2;PCDHA1;P( | Body;Body;1stExon, | Island | 2.50E-02 | 8.97E-22 |
| cg08291506 | NA | NA | NA | -7.57E-03 | 8.50E-09 |
| cg08295410 | ADAM19 | Body | NA | -1.50E-02 | 2.81E-14 |
| cg08305575 | NA | NA | NA | -1.38E-02 | 5.77E-05 |
| cg08310842 | NA | NA | NA | -9.52E-03 | 1.37E-05 |
| cg08312412 | NA | NA | NA | -9.47E-03 | 7.62E-09 |
| cg08313406 | NA | NA | N_Shore | -1.89E-02 | 3.18E-12 |
| cg08315174 | NKX2-1 | TSS1500 | Island | 1.24E-02 | 5.57E-08 |
| cg08316825 | GLRA1;GLRA1 | TSS200;TSS200 | Island | 5.85E-03 | 1.11E-05 |
| cg08331163 | OR4D11 | 1stExon | NA | -1.14E-02 | 4.03E-10 |
| cg08337633 | VOPP1 | Body | NA | -7.30E-03 | 1.67E-05 |
| cg08343644 | GPR56;GPR56;GPR5 | TSS1500;TSS1500;T | NA | -1.76E-02 | 1.43E-09 |
| cg08349804 | GPR162;CD4;GPR16 | TSS1500;3'UTR;TSS: | N_Shelf | -5.76E-03 | 1.21E-05 |
| cg08355099 | PLEKHA6 | Body | N_Shore | -7.43E-03 | 4.64E-10 |
| cg08360457 | MIR1289-2;FSTL4 | TSS1500;Body | NA | -7.77E-03 | 1.13E-05 |
| cg08363794 | HOXD9 | TSS1500 | Island | 1.05E-02 | 9.79E-07 |
| cg08370546 | PHKG1 | TSS200 | NA | -1.51E-02 | 4.76E-10 |
| cg08370996 | NR2F2;NR2F2 | TSS200;Body | Island | 1.28E-02 | 4.54E-07 |
| cg08372315 | HTR3A;HTR3A | TSS1500;TSS1500 | NA | -1.04E-02 | 7.98E-06 |
| cg08382072 | GCNT2 | TSS1500 | NA | -1.31E-02 | 4.92E-10 |
| cg08386692 | SHISA2 | TSS200 | Island | 9.01E-03 | 4.54E-06 |
| cg08398371 | CCDC97 | TSS1500 | N_Shore | -1.18E-02 | 3.81E-16 |
| cg08404354 | HABP2 | TSS1500 | NA | -8.25E-03 | 4.25E-08 |
| cg08412537 | NA | NA | N_Shelf | -1.16E-02 | 6.51E-09 |
| cg08415592 | APOL1;APOL1;APOL | TSS200;TSS200;TSS: | NA | -1.04E-02 | 9.29E-09 |
| cg08418279 | ZNF423 | Body | S_Shore | -1.64E-02 | 3.70E-09 |
| cg08425187 | NA | NA | N_Shore | -5.69E-03 | 1.50E-05 |
| cg08426733 | VWA1;VWA1 | 3'UTR;3'UTR | S_Shore | -5.41E-03 | 2.96E-08 |
| cg08428188 | NA | NA | Island | -1.75E-02 | 4.03E-08 |
| cg08443717 | NA | NA | N_Shore | 3.77E-03 | 9.86E-05 |
| cg08445802 | NA | NA | Island | 1.30E-02 | 1.15E-05 |
| cg08448711 | NA | NA | NA | -1.07E-02 | 9.41E-08 |
| cg08448812 | PCDHA6;PCDHA2;P( | 1stExon;Body;Body, | Island | 1.33E-02 | 2.82E-06 |
| cg08453194 | CCND3;CCND3;CCNI | Body;Body;Body;Bo | NA | -7.90E-03 | 2.92E-07 |
| cg08456051 | NA | NA | N_Shelf | -5.34E-02 | 6.44E-11 |
| cg08468401 | NA | NA | NA | -2.74E-02 | 1.57E-16 |
| cg08468689 | GHDC;GHDC;GHDC;( | TSS200;TSS1500;TS: | NA | -9.64E-03 | 1.59E-05 |
| cg08470639 | NTRK2;NTRK2;NTRK | 5'UTR;5'UTR;5'UTR; | Island | 8.39E-03 | 5.88E-08 |
| cg08474859 | NA | NA | Island | 1.53E-02 | 8.25E-10 |
| cg08476244 | EPB41L5 | Body | NA | -9.11E-03 | 6.15E-05 |
| cg08486875 | NA | NA | Island | 8.78E-03 | 8.90E-06 |

| | | | | | |
|---|---|---|---|---|---|
| cg08493959 | OBSL1;INHA | TSS1500;TSS200 | S_Shore | -8.73E-03 | 1.82E-05 |
| cg08495239 | LOC619207 | Body | Island | 1.42E-02 | 2.85E-05 |
| cg08497772 | NA | NA | N_Shore | -1.31E-02 | 2.47E-12 |
| cg08499881 | AANAT | 5'UTR | NA | -5.61E-03 | 4.37E-07 |
| cg08505243 | NA | NA | N_Shore | 9.03E-03 | 2.81E-07 |
| cg08512167 | PPP1R13L;PPP1R13I | Body;Body | Island | 9.57E-03 | 1.05E-05 |
| cg08512702 | LOC285548 | Body | Island | 8.57E-03 | 2.19E-05 |
| cg08515264 | ALDH1L1 | Body | NA | -9.52E-03 | 1.18E-08 |
| cg08517286 | LRP12;LRP12 | 1stExon;1stExon | Island | 2.53E-02 | 3.31E-05 |
| cg08519191 | NA | NA | NA | -8.23E-03 | 2.57E-10 |
| cg08521995 | ANK1;ANK1;ANK1;A | Body;Body;Body;Bo | S_Shelf | -1.14E-02 | 2.73E-12 |
| cg08535938 | USP21;USP21 | TSS1500;TSS1500 | N_Shore | -4.88E-03 | 5.99E-05 |
| cg08541155 | NA | NA | NA | -1.63E-02 | 8.02E-07 |
| cg08550421 | PCDHA7;PCDHAC1;F | Body;TSS1500;Body | Island | 9.76E-03 | 3.44E-09 |
| cg08555657 | SPRR2E | TSS1500 | NA | -1.92E-02 | 1.10E-12 |
| cg08558886 | NA | NA | NA | -1.72E-02 | 2.58E-13 |
| cg08566455 | NA | NA | Island | 2.70E-02 | 1.14E-13 |
| cg08567760 | C20orf79 | TSS1500 | NA | -8.76E-03 | 1.81E-07 |
| cg08572611 | ACTL6B | Body | Island | 1.41E-02 | 5.84E-05 |
| cg08578520 | POLR2C | TSS1500 | N_Shore | -1.07E-02 | 7.46E-06 |
| cg08587864 | C1orf105;PIGC;PIGC | Body;TSS1500;TSS1 | S_Shore | -9.04E-03 | 4.93E-05 |
| cg08593157 | SMCR5;RAI1 | TSS1500;5'UTR | N_Shore | -7.34E-03 | 1.61E-06 |
| cg08597761 | HYDIN;HYDIN;HYDIN | 1stExon;1stExon;5'U | Island | 1.37E-02 | 7.37E-05 |
| cg08599909 | NA | NA | NA | -7.02E-03 | 8.63E-06 |
| cg08604596 | CCDC101 | 5'UTR | S_Shore | -7.47E-03 | 1.37E-06 |
| cg08613144 | LYPD1;LYPD1;LYPD1 | 1stExon;5'UTR;TSS1 | Island | 2.03E-02 | 3.61E-09 |
| cg08616061 | PCDHGA4;PCDHGA1 | Body;Body;TSS200;I | N_Shore | 1.76E-02 | 1.70E-13 |
| cg08619515 | CLEC16A | Body | NA | -1.76E-02 | 1.53E-19 |
| cg08621843 | PROC | Body | Island | 1.10E-02 | 7.72E-06 |
| cg08622934 | NA | NA | S_Shore | -1.20E-02 | 1.64E-07 |
| cg08640634 | NR2E1 | TSS1500 | Island | 1.26E-02 | 7.87E-05 |
| cg08644498 | NA | NA | NA | -6.26E-03 | 2.23E-10 |
| cg08647730 | NA | NA | NA | -6.64E-03 | 8.88E-05 |
| cg08648727 | ZNF418 | 5'UTR | Island | 7.63E-03 | 1.08E-07 |
| cg08659357 | HRASLS;HRASLS;MG | 1stExon;5'UTR;TSS1 | Island | 1.83E-02 | 2.76E-06 |
| cg08665930 | APOBEC3D | Body | NA | -1.26E-02 | 6.40E-14 |
| cg08668316 | NA | NA | Island | 1.14E-02 | 6.58E-10 |
| cg08672140 | LHFPL2 | 5'UTR | NA | -9.09E-03 | 1.33E-05 |
| cg08681855 | ZNF578 | 5'UTR | Island | 7.78E-03 | 2.02E-06 |
| cg08682153 | PSD2 | 3'UTR | N_Shelf | -1.92E-02 | 1.08E-11 |
| cg08685909 | TH1L | Body | S_Shelf | -7.15E-03 | 1.12E-09 |
| cg08686462 | INS-IGF2;IGF2;INS-IG | Body;TSS1500;Body | NA | -6.70E-03 | 5.59E-08 |
| cg08688773 | NA | NA | Island | 1.46E-02 | 2.05E-10 |
| cg08692733 | RBM20 | Body | S_Shore | 1.42E-02 | 2.08E-05 |
| cg08695830 | CLIP2;CLIP2 | TSS1500;TSS1500 | Island | -9.28E-03 | 1.85E-06 |
| cg08699512 | OTOA;OTOA;OTOA;( | 5'UTR;1stExon;Body | NA | -9.17E-03 | 9.31E-11 |
| cg08702941 | BEND4;BEND4 | TSS1500;TSS1500 | S_Shore | -2.01E-02 | 3.16E-13 |
| cg08713098 | ZCWPW1 | Body | NA | -8.17E-03 | 6.52E-05 |
| cg08718490 | SPATA18 | Body | Island | 1.31E-02 | 2.01E-06 |
| cg08734740 | ISL1;ISL1 | 5'UTR;1stExon | Island | 1.72E-02 | 2.78E-06 |

| | | | | | |
|---|---|---|---|---|---|
| cg08737116 | SLC22A15 | Body | NA | -1.16E-02 | 1.37E-06 |
| cg08739433 | WNT2B;WNT2B | Body;1stExon | Island | 1.24E-02 | 1.03E-08 |
| cg08748308 | LCP2 | Body | NA | -8.18E-03 | 8.59E-05 |
| cg08761208 | IGDCC4 | Body | S_Shelf | -9.34E-03 | 4.49E-06 |
| cg08770647 | ROBO4 | TSS1500 | NA | -1.04E-02 | 4.63E-15 |
| cg08802053 | NA | NA | S_Shore | 6.64E-03 | 2.40E-06 |
| cg08809361 | NA | NA | N_Shore | -9.64E-03 | 9.02E-10 |
| cg08815403 | HSD17B13;HSD17B1 | TSS200;TSS200 | NA | -1.34E-02 | 2.75E-10 |
| cg08816023 | FGF1;FGF1;FGF1;FG | 5'UTR;Body;Body;5' | NA | -8.54E-03 | 2.81E-08 |
| cg08820801 | FBXO17 | 5'UTR | N_Shore | -1.10E-02 | 4.25E-09 |
| cg08832906 | CLEC2L | 1stExon | Island | 1.51E-02 | 8.53E-06 |
| cg08844900 | PLXND1 | TSS1500 | S_Shore | -5.56E-03 | 5.19E-05 |
| cg08849574 | ZNF134 | 5'UTR | S_Shore | 8.91E-03 | 2.59E-08 |
| cg08850461 | NA | NA | NA | 1.61E-02 | 3.01E-06 |
| cg08857994 | NA | NA | Island | 1.44E-02 | 4.05E-09 |
| cg08868213 | ZNF385A;ZNF385A;Z | Body;Body;Body | N_Shore | -7.94E-03 | 1.03E-06 |
| cg08869955 | PTPN3;PTPN3 | TSS1500;TSS1500 | Island | 9.95E-03 | 9.23E-08 |
| cg08876103 | MACF1 | Body | S_Shore | -1.30E-02 | 3.25E-12 |
| cg08878177 | NKAIN4 | Body | N_Shore | -6.30E-03 | 1.66E-05 |
| cg08880817 | TXNDC5;TXNDC5 | Body;5'UTR | N_Shore | -5.81E-03 | 4.65E-05 |
| cg08885800 | NA | NA | Island | 8.72E-03 | 7.76E-05 |
| cg08888956 | NTS | TSS1500 | NA | -1.17E-02 | 1.19E-08 |
| cg08893692 | NA | NA | Island | 1.81E-02 | 1.68E-05 |
| cg08897044 | MAST1 | Body | N_Shore | -6.86E-03 | 8.63E-05 |
| cg08908135 | ATP11A;ATP11A | Body;Body | NA | -2.54E-02 | 4.90E-06 |
| cg08913523 | NA | NA | NA | -1.00E-02 | 1.53E-06 |
| cg08918749 | LPL | Body | Island | 1.40E-02 | 8.99E-10 |
| cg08928145 | TSSK6;TSSK6 | 3'UTR;1stExon | Island | 1.49E-02 | 2.41E-11 |
| cg08928882 | LOC100192378;ZFH | Body;TSS200 | N_Shore | 4.12E-02 | 8.40E-11 |
| cg08941853 | NA | NA | NA | -1.81E-02 | 1.75E-06 |
| cg08943116 | NA | NA | NA | -1.32E-02 | 1.18E-15 |
| cg08947084 | SNORD47;GAS5;SNC | TSS1500;Body;TSS1 | N_Shelf | -1.38E-02 | 6.15E-07 |
| cg08947167 | CA12;CA12 | Body;Body | N_Shelf | -8.65E-03 | 5.47E-08 |
| cg08947774 | PALLD;PALLD;PALLD | Body;Body;Body | NA | -1.05E-02 | 2.78E-06 |
| cg08949164 | BAIAP3 | TSS1500 | Island | 7.87E-03 | 7.21E-05 |
| cg08951271 | DDR1 | TSS1500 | N_Shore | -8.72E-03 | 5.95E-06 |
| cg08958294 | GRM1;GRM1 | 5'UTR;5'UTR | N_Shore | 2.00E-02 | 3.39E-07 |
| cg08964780 | MIR196B | TSS1500 | Island | 2.93E-02 | 5.56E-06 |
| cg08975803 | KRT7 | TSS200 | Island | 6.21E-03 | 5.17E-05 |
| cg08992872 | CALB1;CALB1 | 1stExon;5'UTR | NA | 1.99E-02 | 3.91E-13 |
| cg08993878 | NA | NA | NA | -3.67E-02 | 4.01E-07 |
| cg08996521 | CISH;CISH | TSS1500;TSS1500 | S_Shore | -9.64E-03 | 1.02E-07 |
| cg09001514 | ARSI | Body | S_Shore | -2.37E-02 | 7.08E-25 |
| cg09003833 | NA | NA | S_Shore | 8.44E-03 | 4.36E-06 |
| cg09010707 | NA | NA | NA | -4.85E-03 | 8.03E-05 |
| cg09012633 | LOC100130015;LOC | TSS200;Body | NA | 2.08E-02 | 2.73E-08 |
| cg09017434 | 11-mar | 1stExon | Island | 2.58E-02 | 1.57E-12 |
| cg09029085 | IGF2BP1;IGF2BP1 | Body;Body | S_Shore | -1.72E-02 | 9.71E-16 |
| cg09029624 | PCDHGA4;PCDHGA1 | Body;Body;1stExon, | Island | 6.89E-03 | 6.27E-05 |
| cg09032973 | PCDHA1;PCDHA1;P( | 1stExon;1stExon;1s | Island | 1.63E-02 | 4.59E-13 |

| | | | | | |
|---|---|---|---|---|---|
| cg09039751 | CHST1 | Body | Island | 1.29E-02 | 4.58E-05 |
| cg09064482 | CCDC64B | Body | S_Shore | -4.95E-03 | 2.33E-05 |
| cg09076010 | ARHGEF10L;ARHGEI | Body;Body | N_Shore | -6.52E-03 | 3.50E-05 |
| cg09088834 | NINL | 5'UTR | Island | 5.75E-03 | 8.48E-05 |
| cg09091424 | ANKRD13D;ANKRD1 | Body;Body | S_Shore | -6.23E-03 | 1.92E-06 |
| cg09098195 | BEND4;BEND4 | TSS1500;TSS1500 | S_Shore | -1.76E-02 | 5.11E-05 |
| cg09098707 | WSCD2 | 5'UTR | S_Shore | -1.05E-02 | 2.52E-08 |
| cg09105193 | CPM;CPM;CPM | TSS1500;TSS1500;5 | Island | 1.52E-02 | 4.59E-07 |
| cg09107240 | NA | NA | NA | -7.38E-03 | 1.11E-09 |
| cg09108532 | NA | NA | NA | -7.66E-03 | 2.89E-10 |
| cg09115485 | TNXB;TNXB | Body;TSS1500 | NA | -9.24E-03 | 4.74E-07 |
| cg09124496 | LOC285954;LOC285 | Body;Body;Body | NA | -1.30E-02 | 4.03E-06 |
| cg09125127 | NA | NA | NA | -1.03E-02 | 1.19E-08 |
| cg09126659 | CLEC18C | TSS1500 | NA | -8.36E-03 | 4.16E-07 |
| cg09135529 | NA | NA | S_Shelf | -1.55E-02 | 4.96E-15 |
| cg09137453 | CLSTN3;RBP5 | TSS1500;TSS1500 | NA | -7.08E-03 | 6.23E-07 |
| cg09137533 | NA | NA | S_Shore | -9.58E-03 | 3.63E-05 |
| cg09138671 | TMCO4 | 5'UTR | N_Shore | -1.04E-02 | 8.49E-10 |
| cg09161455 | NR1D1 | Body | Island | 1.16E-02 | 1.91E-05 |
| cg09168222 | HERC6;HERC6 | TSS200;TSS200 | N_Shore | -1.54E-02 | 7.95E-13 |
| cg09168805 | ABCA4 | Body | NA | -1.33E-02 | 9.33E-10 |
| cg09168808 | NA | NA | N_Shore | 1.22E-02 | 9.22E-06 |
| cg09168997 | ATP1A2 | TSS1500 | NA | -8.52E-03 | 1.81E-08 |
| cg09175289 | TM7SF2;C11orf2 | TSS1500;3'UTR | Island | 4.49E-03 | 8.75E-05 |
| cg09183450 | AHNAK;AHNAK | 5'UTR;5'UTR | NA | -2.02E-02 | 3.35E-15 |
| cg09191036 | DRD5;DRD5 | 1stExon;5'UTR | Island | 8.51E-03 | 1.44E-05 |
| cg09192086 | NA | NA | NA | -6.60E-03 | 7.80E-07 |
| cg09198200 | NA | NA | NA | -9.51E-03 | 4.43E-12 |
| cg09219547 | RPS6KA2;RPS6KA2 | Body;Body | S_Shore | -1.31E-02 | 3.40E-09 |
| cg09222982 | NA | NA | NA | -1.29E-02 | 1.46E-09 |
| cg09232937 | IRX1 | TSS200 | Island | 9.93E-03 | 3.67E-09 |
| cg09244244 | ARSK;TTC37 | Body;TSS1500 | S_Shore | -1.03E-02 | 3.42E-05 |
| cg09247060 | TFAP2B | Body | Island | 7.31E-03 | 2.83E-06 |
| cg09253473 | MAFG;MAFG;SIRT7 | 3'UTR;3'UTR;TSS15( | S_Shore | -8.60E-03 | 5.07E-08 |
| cg09253581 | LYPD5;LYPD5 | Body;Body | Island | 1.60E-02 | 2.49E-05 |
| cg09259081 | KIAA1609 | TSS1500 | Island | -2.34E-02 | 6.77E-08 |
| cg09262442 | C10orf18 | 5'UTR | S_Shore | -2.38E-02 | 7.61E-05 |
| cg09267051 | NA | NA | NA | 2.37E-02 | 2.86E-07 |
| cg09270285 | PKNOX2 | Body | NA | 6.97E-03 | 8.54E-06 |
| cg09275667 | PYGO1 | TSS200 | Island | 9.64E-03 | 5.59E-07 |
| cg09283154 | SLC35F1 | Body | Island | 6.46E-03 | 1.61E-05 |
| cg09287933 | CUTA;CUTA;CUTA;C | Body;Body;Body;Bo | N_Shore | -7.39E-03 | 2.44E-05 |
| cg09289148 | NA | NA | Island | -8.52E-03 | 3.45E-05 |
| cg09292354 | NA | NA | S_Shore | -1.14E-02 | 2.99E-11 |
| cg09293786 | NA | NA | Island | 1.98E-02 | 9.37E-05 |
| cg09294739 | NA | NA | NA | -1.35E-02 | 5.72E-05 |
| cg09304357 | NA | NA | NA | -1.75E-02 | 9.30E-12 |
| cg09307530 | NA | NA | Island | 8.64E-03 | 3.14E-06 |
| cg09317239 | NA | NA | S_Shore | -7.16E-02 | 6.99E-15 |
| cg09317508 | NA | NA | NA | -1.37E-02 | 1.54E-07 |

| | | | | | |
|---|---|---|---|---|---|
| cg09318763 | NA | NA | NA | -1.25E-02 | 1.97E-12 |
| cg09322513 | LOC283867 | Body | NA | -1.00E-02 | 3.55E-07 |
| cg09331106 | NA | NA | N_Shore | -5.44E-03 | 8.93E-06 |
| cg09336320 | CPLX2 | 5'UTR | Island | 9.26E-03 | 8.80E-06 |
| cg09340639 | FCRL1;FCRL1;FCRL1 | Body;Body;Body | NA | -1.55E-02 | 6.77E-09 |
| cg09342225 | NA | NA | NA | -1.94E-02 | 5.01E-19 |
| cg09354241 | NA | NA | N_Shelf | 1.22E-02 | 1.99E-05 |
| cg09360654 | MUTYH;MUTYH;MU | TSS1500;TSS1500;T | S_Shore | -8.81E-03 | 1.55E-05 |
| cg09360770 | FOXC2 | TSS1500 | Island | 2.31E-02 | 2.33E-11 |
| cg09384035 | NA | NA | NA | -2.43E-02 | 6.03E-11 |
| cg09391066 | NA | NA | S_Shore | -1.32E-02 | 2.88E-08 |
| cg09395195 | EVI2A;EVI2A;NF1;NF | Body;Body;Body;Bo | NA | -1.54E-02 | 5.89E-14 |
| cg09401099 | NA | NA | Island | 2.68E-02 | 5.32E-13 |
| cg09409484 | LOC400752 | Body | S_Shore | -5.58E-03 | 1.17E-05 |
| cg09410607 | 04-mar | TSS1500 | Island | 3.05E-02 | 5.82E-09 |
| cg09411874 | KDM2B;KDM2B | Body;Body | Island | -5.82E-03 | 7.99E-05 |
| cg09418063 | ZNF208 | Body | Island | 2.01E-02 | 2.00E-05 |
| cg09418701 | PAPSS2;PAPSS2 | Body;Body | NA | -8.14E-03 | 6.68E-05 |
| cg09419102 | NA | NA | S_Shore | -6.07E-03 | 2.49E-05 |
| cg09424759 | SLC35F3;SLC35F3 | 1stExon;5'UTR | Island | 9.01E-03 | 7.67E-05 |
| cg09427311 | ANGPTL2;RALGPS1 | TSS1500;Body | NA | -9.70E-03 | 2.33E-14 |
| cg09438075 | ENOX1 | 5'UTR | N_Shore | -1.03E-02 | 1.83E-06 |
| cg09438147 | UNC80;UNC80 | TSS200;TSS200 | Island | 1.02E-02 | 4.69E-05 |
| cg09471659 | NA | NA | Island | 1.76E-02 | 5.81E-06 |
| cg09481404 | NA | NA | Island | 1.79E-02 | 1.97E-07 |
| cg09484541 | FAM169A | 5'UTR | N_Shore | -1.67E-02 | 7.78E-09 |
| cg09487078 | CCR6;CCR6 | 5'UTR;1stExon | NA | -1.10E-02 | 6.63E-14 |
| cg09492887 | SLC26A5;SLC26A5;S | 5'UTR;5'UTR;5'UTR; | Island | 1.04E-02 | 1.61E-06 |
| cg09499629 | KLF14 | TSS1500 | Island | 2.47E-02 | 1.25E-18 |
| cg09499856 | PLCXD3 | Body | Island | 1.42E-02 | 1.07E-05 |
| cg09507184 | ISG20L2;C1orf66;C1 | 1stExon;TSS1500;TS | N_Shore | -9.92E-03 | 1.67E-08 |
| cg09510752 | NA | NA | N_Shore | -2.68E-02 | 1.00E-24 |
| cg09522056 | NA | NA | Island | 1.72E-02 | 1.72E-08 |
| cg09538452 | GDF15 | TSS1500 | N_Shore | -1.53E-02 | 4.09E-07 |
| cg09539538 | FERMT1 | TSS200 | S_Shore | -1.15E-02 | 3.37E-12 |
| cg09543255 | SLC7A14 | 5'UTR | N_Shore | 1.55E-02 | 4.65E-12 |
| cg09554951 | KCNQ1DN | TSS1500 | Island | 1.67E-02 | 2.46E-07 |
| cg09558425 | NA | NA | NA | -1.86E-02 | 8.30E-17 |
| cg09559189 | EBF2 | Body | N_Shore | 1.18E-02 | 1.18E-05 |
| cg09560590 | HMHB1 | TSS200 | NA | 8.69E-03 | 6.50E-05 |
| cg09563216 | C1orf51;C1orf51 | 1stExon;5'UTR | S_Shore | 1.05E-02 | 3.22E-05 |
| cg09570614 | NA | NA | NA | -8.53E-03 | 2.30E-11 |
| cg09572067 | RRN3P2 | Body | NA | -1.11E-02 | 1.62E-07 |
| cg09577986 | NA | NA | NA | -6.94E-03 | 3.20E-06 |
| cg09578475 | ZNF177 | TSS200 | Island | 2.63E-02 | 1.17E-13 |
| cg09582200 | FXYD6;FXYD6;FXYD6 | TSS1500;TSS1500;T | S_Shore | -6.55E-03 | 6.36E-05 |
| cg09585333 | FAM89B;FAM89B;FA | Body;Body;Body | N_Shore | -8.90E-03 | 1.23E-12 |
| cg09596818 | NPTX2 | TSS1500 | N_Shore | 1.05E-02 | 1.29E-06 |
| cg09604283 | NA | NA | N_Shore | -8.15E-03 | 1.62E-09 |
| cg09604569 | ARMC9 | Body | NA | -1.44E-02 | 4.82E-10 |

| | | | | | |
|---|---|---|---|---|---|
| cg09608765 | LIMD1 | TSS200 | S_Shore | -1.10E-02 | 3.26E-11 |
| cg09609418 | NA | NA | Island | 5.77E-03 | 8.17E-05 |
| cg09615056 | C16orf91 | Body | Island | -1.95E-02 | 1.80E-05 |
| cg09635866 | NA | NA | Island | -1.40E-02 | 2.72E-05 |
| cg09636313 | NA | NA | N_Shore | -9.60E-03 | 1.90E-08 |
| cg09636661 | NQO1;NQO1;NQO1 | 3'UTR;3'UTR;3'UTR | NA | -1.42E-02 | 9.03E-09 |
| cg09639890 | FGF12 | Body | NA | -1.28E-02 | 2.38E-05 |
| cg09643136 | NA | NA | Island | 7.71E-03 | 5.90E-06 |
| cg09643544 | ZNF177;ZNF177 | 1stExon;5'UTR | Island | 1.51E-02 | 1.61E-12 |
| cg09644153 | NA | NA | S_Shore | 1.21E-02 | 2.14E-05 |
| cg09648727 | NA | NA | S_Shore | -1.12E-02 | 1.54E-05 |
| cg09652746 | E2F4;EXOC3L | TSS1500;TSS1500 | NA | -1.18E-02 | 2.04E-11 |
| cg09655666 | NA | NA | Island | 2.00E-02 | 4.57E-11 |
| cg09660365 | SLC6A11 | Body | Island | 1.07E-02 | 1.17E-05 |
| cg09661809 | NA | NA | Island | 7.42E-03 | 7.37E-08 |
| cg09673807 | NA | NA | NA | -7.83E-03 | 9.77E-09 |
| cg09676013 | HLA-DPB2 | Body | NA | -1.40E-02 | 1.39E-12 |
| cg09676630 | FBRSL1 | Body | N_Shore | -5.40E-03 | 3.79E-05 |
| cg09679234 | GABRA2;GABRA2 | TSS1500;TSS1500 | Island | 8.76E-03 | 8.33E-05 |
| cg09684846 | FAM18A | TSS200 | Island | -2.62E-02 | 1.88E-05 |
| cg09692396 | LRRC23;ENO2;LRRC2 | 3'UTR;TSS1500;3'U | Island | -1.37E-02 | 2.43E-08 |
| cg09700701 | GAL3ST3 | TSS200 | S_Shore | 6.00E-03 | 7.46E-06 |
| cg09701700 | MIR146B | TSS1500 | S_Shelf | -1.05E-02 | 2.21E-09 |
| cg09706243 | LOC100130987;POL | Body;TSS1500 | S_Shore | -6.05E-03 | 2.38E-05 |
| cg09719217 | PRRT4 | Body | Island | 7.13E-03 | 1.81E-05 |
| cg09726279 | MYBBP1A;MYBBP1A | Body;Body | N_Shore | -4.97E-03 | 1.53E-05 |
| cg09729387 | ST18 | 5'UTR | NA | -1.58E-02 | 4.40E-09 |
| cg09730640 | NA | NA | S_Shore | 7.46E-03 | 1.15E-05 |
| cg09741713 | FAM19A2 | 5'UTR | NA | -1.77E-02 | 2.77E-21 |
| cg09748749 | ASL;ASL;ASL;ASL | TSS1500;TSS1500;T | N_Shore | -9.29E-03 | 4.70E-07 |
| cg09763644 | PIGR | 3'UTR | NA | 1.26E-02 | 1.62E-06 |
| cg09788352 | CLN6 | TSS1500 | S_Shore | -9.20E-03 | 2.38E-09 |
| cg09788416 | NA | NA | Island | 1.96E-02 | 8.53E-06 |
| cg09789939 | ZFPM1 | Body | NA | -5.04E-02 | 1.28E-15 |
| cg09793172 | KIF17;KIF17 | 1stExon;1stExon | Island | 1.23E-02 | 3.91E-05 |
| cg09796457 | FEM1B | Body | S_Shore | -1.49E-02 | 1.78E-12 |
| cg09797337 | NA | NA | N_Shore | 1.93E-02 | 1.94E-09 |
| cg09799873 | NDUFA7;RPS28 | Body;TSS1500 | N_Shore | -1.35E-02 | 1.10E-08 |
| cg09803002 | NLRX1;NLRX1 | TSS1500;TSS1500 | N_Shore | -7.68E-03 | 3.91E-06 |
| cg09805709 | PLEKHG5;PLEKHG5;I | Body;Body;TSS150C | S_Shore | -1.30E-02 | 1.69E-12 |
| cg09809672 | EDARADD;EDARADD | TSS1500;5'UTR;1stE | N_Shore | -2.12E-02 | 2.59E-23 |
| cg09814231 | PPARGC1B | Body | NA | -8.42E-03 | 5.83E-11 |
| cg09824603 | NA | NA | NA | -1.25E-02 | 9.10E-06 |
| cg09826973 | UBL4B;UBL4B | 3'UTR;1stExon | NA | -6.67E-03 | 5.08E-05 |
| cg09852209 | ITPK1;ITPK1;ITPK1 | Body;Body;Body | N_Shore | -7.65E-03 | 6.81E-07 |
| cg09858674 | NA | NA | N_Shelf | -1.11E-02 | 8.27E-05 |
| cg09869291 | NA | NA | NA | -1.40E-02 | 1.44E-11 |
| cg09872491 | NA | NA | NA | 6.47E-03 | 9.97E-06 |
| cg09879794 | CCDC136;CCDC136 | 1stExon;5'UTR | S_Shore | -8.76E-03 | 9.65E-07 |
| cg09882118 | TREX1;TREX1 | Body;1stExon | NA | -9.61E-03 | 6.68E-06 |

| | | | | | |
|---|---|---|---|---|---|
| cg09883394 | NA | NA | NA | -1.18E-02 | 2.25E-06 |
| cg09884851 | SYT1;SYT1;SYT1 | Body;Body;Body | NA | -1.95E-02 | 7.64E-07 |
| cg09896412 | NA | NA | Island | 1.51E-02 | 8.93E-06 |
| cg09909740 | NA | NA | NA | -2.28E-02 | 4.68E-06 |
| cg09924848 | FAM53B | 3'UTR | NA | -7.36E-03 | 9.16E-06 |
| cg09935271 | YJEFN3 | Body | S_Shore | -1.85E-02 | 2.42E-08 |
| cg09942743 | ALK | Body | NA | -1.17E-02 | 2.68E-05 |
| cg09979478 | NA | NA | Island | 2.23E-02 | 3.46E-09 |
| cg09980058 | COMP | Body | Island | 1.83E-02 | 3.04E-08 |
| cg10000952 | NA | NA | Island | 9.35E-03 | 2.46E-06 |
| cg10005978 | DMRT3 | Body | Island | 5.72E-03 | 5.24E-05 |
| cg10037005 | CD37;CD37 | TSS200;TSS200 | N_Shelf | -7.40E-03 | 5.27E-05 |
| cg10043090 | HCCA2 | Body | Island | -7.49E-03 | 1.49E-05 |
| cg10045354 | C11orf93;C11orf92 | TSS1500;5'UTR | Island | -7.17E-03 | 3.25E-06 |
| cg10047595 | FBXL7 | Body | Island | 1.42E-02 | 4.64E-11 |
| cg10055566 | NKAPL | 1stExon | Island | 1.00E-02 | 1.66E-06 |
| cg10059660 | KCTD8 | 1stExon | Island | 1.61E-02 | 1.45E-07 |
| cg10074727 | GCM2 | TSS1500 | Island | 1.55E-02 | 4.77E-09 |
| cg10075819 | LIMS1 | 5'UTR | NA | -9.69E-03 | 3.00E-12 |
| cg10092779 | NA | NA | NA | -7.93E-03 | 3.14E-06 |
| cg10092878 | MLXIPL;MLXIPL;MLX | 1stExon;1stExon;5'U | Island | 2.09E-02 | 2.96E-09 |
| cg10098021 | KIAA1949;KIAA1949 | Body;1stExon | N_Shore | -1.80E-02 | 4.71E-06 |
| cg10107473 | EPAS1 | Body | NA | -1.65E-02 | 7.96E-20 |
| cg10111115 | PDZRN3 | TSS200 | Island | 6.91E-03 | 9.33E-07 |
| cg10116864 | CDK5R2;CDK5R2 | 3'UTR;1stExon | N_Shore | 1.50E-02 | 6.88E-07 |
| cg10117719 | C1orf216 | 3'UTR | N_Shelf | -7.91E-03 | 9.57E-13 |
| cg10120698 | HYDIN | Body | NA | -9.95E-03 | 1.94E-14 |
| cg10124993 | CDCA4;CDCA4 | 5'UTR;5'UTR | NA | -9.49E-03 | 9.20E-07 |
| cg10129391 | NA | NA | NA | -8.68E-03 | 5.14E-05 |
| cg10130088 | NA | NA | NA | -1.18E-02 | 6.13E-16 |
| cg10134939 | FAM176A | TSS200 | NA | -1.40E-02 | 9.54E-12 |
| cg10137837 | BCL6B | 5'UTR | Island | 8.62E-03 | 1.49E-05 |
| cg10141715 | SLC5A8 | 1stExon | Island | 9.99E-03 | 5.20E-05 |
| cg10142237 | CSNK1E;CSNK1E | TSS1500;TSS1500 | S_Shore | -5.54E-03 | 2.33E-05 |
| cg10145584 | IL10RA;IL10RA | Body;Body | S_Shelf | -7.17E-03 | 1.80E-05 |
| cg10145941 | SPEN | Body | S_Shelf | -1.56E-02 | 6.26E-08 |
| cg10149582 | CMTM5;CMTM5;CM | 1stExon;5'UTR;5'UT | NA | -9.91E-03 | 6.51E-07 |
| cg10150592 | NA | NA | Island | 8.97E-03 | 1.82E-07 |
| cg10151685 | NA | NA | Island | 1.24E-02 | 2.32E-05 |
| cg10152131 | NA | NA | S_Shore | 2.19E-02 | 3.31E-06 |
| cg10152449 | CHST12 | 5'UTR | S_Shore | -1.30E-02 | 4.33E-08 |
| cg10158679 | VARS2;VARS2;VARS | Body;5'UTR;Body | S_Shore | -1.58E-02 | 1.49E-11 |
| cg10162659 | ZFHX3;ZFHX3 | 5'UTR;5'UTR | NA | -9.09E-03 | 1.10E-05 |
| cg10177394 | NA | NA | Island | 2.31E-02 | 3.35E-12 |
| cg10178130 | NA | NA | N_Shelf | -1.09E-02 | 1.70E-11 |
| cg10182317 | CLVS2 | TSS200 | Island | 1.77E-02 | 7.06E-05 |
| cg10189695 | GPR78;GPR78 | 1stExon;5'UTR | Island | 1.64E-02 | 4.11E-19 |
| cg10192893 | PHOX2B | Body | Island | 2.01E-02 | 1.61E-09 |
| cg10195687 | NA | NA | NA | -5.93E-03 | 3.70E-05 |
| cg10198943 | NA | NA | NA | -1.05E-02 | 3.07E-10 |

| | | | | | |
|---|---|---|---|---|---|
| cg10221746 | NA | NA | S_Shore | -8.98E-03 | 2.79E-11 |
| cg10237088 | POFUT1;PLAGL2;PO | TSS1500;5'UTR;TSS: | N_Shore | -7.33E-03 | 4.24E-08 |
| cg10242160 | ZAR1 | Body | Island | 9.32E-03 | 3.04E-07 |
| cg10244503 | NA | NA | NA | -1.06E-02 | 3.06E-10 |
| cg10246448 | ATP8B2;ATP8B2 | Body;Body | Island | -6.11E-03 | 9.85E-06 |
| cg10247711 | WDR25;WDR25 | Body;Body | NA | -4.16E-02 | 5.07E-07 |
| cg10263682 | SOX2OT | Body | Island | 1.43E-02 | 1.98E-06 |
| cg10269365 | CCDC140 | 5'UTR | N_Shore | 8.80E-03 | 3.24E-05 |
| cg10275766 | LOC91149 | Body | NA | -7.52E-03 | 2.91E-07 |
| cg10278102 | PRMT3;PRMT3;PRM | 5'UTR;Body;Body | S_Shore | -1.33E-02 | 3.73E-05 |
| cg10278552 | LOC283867 | Body | NA | -1.78E-02 | 3.69E-08 |
| cg10304534 | ABCC4;ABCC4 | Body;Body | NA | -1.66E-02 | 3.57E-13 |
| cg10316899 | MACF1 | Body | NA | -2.31E-02 | 8.96E-17 |
| cg10319870 | NA | NA | NA | -8.31E-03 | 7.38E-05 |
| cg10320659 | NA | NA | Island | 2.01E-02 | 5.23E-19 |
| cg10323433 | HTR2A;HTR2A | TSS1500;TSS1500 | NA | -3.10E-02 | 7.44E-15 |
| cg10328573 | KRT9 | 1stExon | NA | 7.13E-03 | 7.82E-06 |
| cg10333278 | NA | NA | NA | -1.40E-02 | 3.95E-10 |
| cg10335834 | OPCML | Body | N_Shore | -9.41E-03 | 4.48E-07 |
| cg10356210 | KCNK3 | Body | N_Shelf | -9.21E-03 | 3.01E-09 |
| cg10366797 | HOXC4 | 5'UTR | Island | 8.36E-03 | 4.29E-08 |
| cg10372188 | FXYD6;FXYD6;FXYD6 | TSS1500;TSS1500;T | S_Shore | -7.65E-03 | 1.42E-05 |
| cg10372770 | NA | NA | Island | 1.80E-02 | 2.52E-08 |
| cg10375954 | ACOT7;ACOT7;ACOT | Body;Body;TSS1500 | NA | -1.14E-02 | 5.63E-09 |
| cg10376827 | AGAP11;C10orf116 | TSS200;Body | N_Shore | -1.08E-02 | 1.67E-07 |
| cg10381440 | HOXB2 | TSS1500 | S_Shore | -1.34E-02 | 7.36E-12 |
| cg10383568 | DNHD1 | Body | N_Shelf | -1.13E-02 | 4.38E-12 |
| cg10385089 | OSBPL9;OSBPL9;OSI | TSS1500;TSS1500;B | N_Shore | -1.07E-02 | 2.76E-06 |
| cg10387551 | KCNA4 | TSS200 | Island | 2.05E-02 | 4.80E-06 |
| cg10424681 | C6orf201;C6orf146; | TSS200;5'UTR;1stEx | Island | 1.42E-02 | 2.83E-07 |
| cg10424974 | NA | NA | NA | -1.89E-02 | 2.57E-25 |
| cg10426318 | KIAA1949;KIAA1949 | Body;Body | NA | -1.57E-02 | 3.25E-20 |
| cg10431747 | C1orf94;C1orf94 | 5'UTR;TSS200 | Island | 1.13E-02 | 1.71E-06 |
| cg10460946 | NA | NA | N_Shelf | -1.37E-02 | 2.78E-05 |
| cg10462048 | PCDHGA4;PCDHGA6 | Body;Body;Body;Bo | S_Shore | 6.32E-03 | 1.78E-07 |
| cg10471544 | NME3 | 3'UTR | Island | -1.43E-02 | 6.76E-13 |
| cg10476085 | FGF1;FGF1;FGF1;FG | 5'UTR;5'UTR;5'UTR; | NA | -8.80E-03 | 8.80E-18 |
| cg10477621 | FERD3L | 1stExon | Island | 1.57E-02 | 7.66E-06 |
| cg10480239 | BCR;BCR | TSS1500;TSS1500 | N_Shore | -1.36E-02 | 1.25E-16 |
| cg10493324 | NA | NA | N_Shelf | -1.06E-02 | 2.83E-05 |
| cg10498429 | GPR137;BAD;BAD;G | TSS1500;1stExon;Bo | N_Shore | -5.81E-03 | 2.37E-05 |
| cg10500737 | C21orf34;C21orf34 | Body;TSS1500 | NA | -2.28E-02 | 8.61E-09 |
| cg10501210 | NA | NA | NA | -2.89E-02 | 3.08E-20 |
| cg10521153 | NA | NA | NA | 1.70E-02 | 1.00E-08 |
| cg10521706 | OSR1 | 5'UTR | Island | 8.32E-03 | 1.38E-06 |
| cg10523019 | RHBDD1;RHBDD1 | TSS1500;TSS1500 | Island | 1.09E-02 | 1.41E-06 |
| cg10524576 | PPM1H | Body | NA | -1.20E-02 | 3.31E-07 |
| cg10530883 | IRX1;IRX1 | 1stExon;5'UTR | Island | 1.70E-02 | 6.53E-05 |
| cg10536898 | NA | NA | NA | 1.03E-02 | 3.87E-05 |
| cg10539808 | KCTD1 | TSS1500 | NA | -8.56E-03 | 2.55E-06 |

| | | | | | |
|---|---|---|---|---|---|
| cg10575261 | ZNF454 | TSS200 | Island | 1.44E-02 | 4.30E-08 |
| cg10581837 | LMO7;LMO7 | TSS200;Body | NA | -1.08E-02 | 2.58E-08 |
| cg10583119 | MIR518D | TSS1500 | NA | -1.07E-02 | 1.82E-08 |
| cg10585962 | MOGAT2 | TSS1500 | NA | -1.07E-02 | 2.53E-09 |
| cg10591607 | GRIK2;GRIK2;GRIK2; | 1stExon;1stExon;5'U | Island | 1.51E-02 | 3.53E-11 |
| cg10597359 | MPRIP;MPRIP | Body;Body | S_Shore | -1.37E-02 | 1.62E-14 |
| cg10601943 | HLA-F;HLA-F;HLA-F | Body;Body;Body | S_Shore | -9.22E-03 | 1.33E-09 |
| cg10606059 | TRPA1 | TSS200 | Island | 2.75E-02 | 1.36E-05 |
| cg10606834 | NA | NA | Island | 7.34E-03 | 2.28E-05 |
| cg10608615 | KCNIP1 | TSS200 | NA | -7.54E-03 | 3.89E-08 |
| cg10616795 | NA | NA | NA | -2.28E-02 | 2.15E-14 |
| cg10620680 | NA | NA | NA | -1.26E-02 | 1.12E-08 |
| cg10628205 | NFIA;NFIA;NFIA;NFIA | Body;TSS1500;TSS1 | N_Shore | -1.44E-02 | 1.65E-13 |
| cg10630880 | ABHD14B;ABHD14B | TSS200;TSS200;TSS: | N_Shore | -1.10E-02 | 5.63E-05 |
| cg10635122 | ZNF577;ZNF577;ZNF | Body;5'UTR;5'UTR;1 | Island | 1.49E-02 | 1.05E-05 |
| cg10640845 | ONECUT3 | Body | Island | 1.48E-02 | 5.71E-10 |
| cg10646145 | NA | NA | NA | -1.02E-02 | 1.48E-09 |
| cg10648763 | TGFBI | TSS200 | N_Shore | -4.60E-03 | 6.72E-05 |
| cg10649903 | PCDHA2;PCDHA1;PC | Body;Body;1stExon, | Island | 1.90E-02 | 1.32E-08 |
| cg10658666 | OTX1 | TSS1500 | Island | 1.05E-02 | 2.67E-08 |
| cg10659886 | ZSCAN18;ZSCAN18 | Body;1stExon | Island | 1.11E-02 | 2.99E-05 |
| cg10661163 | ST7OT4;ST7OT1;ST7 | TSS1500;Body;TSS1 | N_Shore | -7.91E-03 | 4.35E-10 |
| cg10661615 | PRLHR | TSS200 | Island | 9.53E-03 | 3.56E-11 |
| cg10664162 | DIP2C | Body | N_Shore | -9.75E-03 | 5.13E-07 |
| cg10667810 | ZNF565;ZNF146;ZNF | 5'UTR;TSS1500;TSS: | N_Shore | -3.88E-03 | 6.11E-05 |
| cg10668363 | ZNF175 | Body | S_Shelf | -1.87E-02 | 2.41E-19 |
| cg10684486 | NA | NA | NA | -8.97E-03 | 8.90E-11 |
| cg10691109 | PRKAR2B | Body | NA | -1.16E-02 | 6.54E-09 |
| cg10691866 | TPST1 | Body | NA | -1.30E-02 | 7.97E-08 |
| cg10711871 | WNT7A | Body | Island | -9.07E-03 | 1.46E-05 |
| cg10721149 | SCGN | TSS200 | Island | 1.58E-02 | 2.31E-11 |
| cg10751726 | LOC150786;LOC150 | 5'UTR;1stExon | Island | 2.91E-02 | 2.79E-12 |
| cg10765336 | NA | NA | N_Shore | -1.10E-02 | 2.70E-07 |
| cg10766585 | SOD3 | 5'UTR | Island | 1.12E-02 | 7.61E-05 |
| cg10772169 | AGL;AGL;AGL;AGL;A | TSS1500;TSS1500;T | N_Shore | -8.09E-03 | 1.20E-07 |
| cg10783469 | ZNF577;ZNF577;ZNF | TSS200;TSS200;TSS: | Island | 1.42E-02 | 1.17E-06 |
| cg10789050 | OSBPL10 | Body | NA | -1.35E-02 | 6.74E-06 |
| cg10796805 | NA | NA | Island | 9.38E-03 | 2.18E-05 |
| cg10796899 | DIP2C | Body | Island | 9.83E-03 | 4.13E-06 |
| cg10798225 | PTDSS2 | TSS1500 | N_Shore | -1.46E-02 | 9.95E-14 |
| cg10799608 | PNPLA3 | 3'UTR | NA | -9.35E-03 | 5.84E-06 |
| cg10804656 | NA | NA | Island | 1.84E-02 | 5.12E-11 |
| cg10805220 | PCDHGA4;PCDHGA1 | Body;Body;Body;Bo | Island | 7.04E-03 | 7.97E-05 |
| cg10805970 | ASAP3;ASAP3 | Body;Body | NA | -8.33E-03 | 2.26E-07 |
| cg10806820 | CELSR3 | 1stExon | Island | 8.45E-03 | 2.80E-06 |
| cg10822172 | CREB5 | TSS200 | NA | -1.29E-02 | 1.87E-14 |
| cg10827253 | PHGDH | TSS1500 | N_Shore | -2.79E-02 | 3.48E-10 |
| cg10836101 | VSTM2B | Body | Island | 2.39E-02 | 7.28E-08 |
| cg10848373 | KCNJ6 | Body | Island | 2.10E-02 | 5.01E-10 |
| cg10855425 | NA | NA | NA | -1.10E-02 | 1.46E-10 |

| | | | | | |
|---|---|---|---|---|---|
| cg10855746 | PRKCH | Body | NA | -7.63E-03 | 3.11E-09 |
| cg10859844 | NA | NA | Island | -1.26E-02 | 4.68E-12 |
| cg10872209 | SFXN2;ARL3 | 5'UTR;TSS1500 | S_Shore | -1.52E-02 | 5.33E-10 |
| cg10874644 | NA | NA | NA | -2.17E-02 | 8.94E-11 |
| cg10877315 | NA | NA | NA | -1.72E-02 | 9.91E-07 |
| cg10890302 | TNXB | Body | Island | 2.03E-02 | 8.68E-05 |
| cg10905495 | EBF2 | Body | NA | 1.94E-02 | 6.22E-10 |
| cg10906284 | AVPR1A | 1stExon | Island | 1.54E-02 | 5.52E-08 |
| cg10912240 | FOXG1 | TSS1500 | Island | 9.35E-03 | 2.23E-07 |
| cg10917602 | HSD3B7;HSD3B7;HS | 5'UTR;5'UTR;5'UTR | NA | -9.66E-03 | 1.76E-07 |
| cg10928466 | DOCK6 | Body | Island | -8.90E-03 | 5.43E-06 |
| cg10941749 | LYSMD2;LYSMD2 | TSS200;5'UTR | S_Shore | -9.24E-03 | 2.99E-12 |
| cg10943359 | 01-mar | TSS200 | Island | 1.11E-02 | 1.19E-11 |
| cg10943458 | STXBP5L;STXBP5L | 1stExon;5'UTR | Island | 1.51E-02 | 7.17E-05 |
| cg10947146 | XKR6 | 1stExon | Island | 1.68E-02 | 4.24E-11 |
| cg10948797 | HIC1 | TSS1500 | Island | -8.45E-03 | 7.27E-05 |
| cg10949430 | ESPN | Body | N_Shelf | -6.34E-03 | 7.91E-06 |
| cg10949576 | C11orf66;C11orf66 | TSS200;TSS200 | NA | -9.82E-03 | 3.58E-09 |
| cg10953487 | NA | NA | NA | 5.47E-03 | 9.54E-05 |
| cg10959950 | LIFR;LIFR | 5'UTR;5'UTR | NA | -9.54E-03 | 9.10E-10 |
| cg10959999 | C3orf36;C3orf36 | 3'UTR;1stExon | S_Shore | -7.85E-03 | 5.92E-08 |
| cg10964160 | INTS10 | TSS1500 | N_Shore | -1.12E-02 | 6.18E-06 |
| cg10964367 | ARHGEF10 | TSS200 | Island | 1.67E-02 | 7.14E-07 |
| cg10967350 | CNTN4 | TSS1500 | Island | 8.23E-03 | 7.03E-11 |
| cg10976961 | ANKRD34A | 3'UTR | S_Shore | -9.96E-03 | 2.88E-09 |
| cg10981651 | NA | NA | Island | 1.47E-02 | 9.02E-08 |
| cg10983206 | NA | NA | NA | -1.89E-02 | 7.10E-11 |
| cg10987126 | COL22A1 | Body | NA | 1.03E-02 | 2.85E-05 |
| cg10989634 | NA | NA | Island | 5.84E-03 | 1.31E-05 |
| cg11009736 | MARCO | TSS200 | NA | -1.34E-02 | 9.87E-11 |
| cg11014079 | SSPO | Body | NA | -5.85E-03 | 7.34E-05 |
| cg11021940 | LVRN | Body | S_Shore | 1.39E-02 | 1.01E-08 |
| cg11024682 | SREBF1;SREBF1 | Body;Body | S_Shelf | 4.64E-03 | 4.06E-05 |
| cg11036041 | LIMCH1;LIMCH1;LIN | Body;Body;Body | S_Shore | -1.40E-02 | 3.61E-05 |
| cg11052535 | OR8U8;OR9G9;OR9 | Body;1stExon;1stEx | NA | -7.53E-03 | 2.13E-05 |
| cg11064966 | NA | NA | NA | -1.36E-02 | 9.00E-18 |
| cg11065458 | UBL4B | TSS1500 | NA | -6.17E-03 | 1.23E-05 |
| cg11071401 | CACNA1G;CACNA1G | TSS1500;TSS1500;T | Island | 1.49E-02 | 1.74E-13 |
| cg11076306 | LIMCH1;LIMCH1;LIN | Body;Body;Body | NA | -1.35E-02 | 1.98E-10 |
| cg11084334 | LHFPL4 | Body | Island | 1.03E-02 | 4.80E-10 |
| cg11095383 | PCDHA1;PCDHA1;PC | Body;Body;1stExon, | Island | 1.38E-02 | 1.59E-06 |
| cg11095743 | NA | NA | S_Shore | -6.84E-03 | 7.44E-07 |
| cg11100804 | PCDHGA4;PCDHGA6 | Body;Body;Body;Bo | S_Shelf | 1.84E-02 | 7.42E-06 |
| cg11117122 | NA | NA | NA | -5.00E-03 | 4.36E-06 |
| cg11119742 | GPR176 | 1stExon | Island | 9.24E-03 | 3.06E-07 |
| cg11120194 | FLT4;FLT4 | Body;Body | NA | -7.35E-03 | 1.70E-05 |
| cg11120551 | CHD1L | TSS1500 | N_Shore | -1.21E-02 | 7.91E-11 |
| cg11128212 | NA | NA | NA | -2.20E-02 | 3.04E-10 |
| cg11139646 | BMP8B | Body | Island | 1.21E-02 | 1.87E-06 |
| cg11142333 | ZBTB37;ZBTB37 | Body;Body | S_Shelf | -1.95E-02 | 4.65E-33 |

| | | | | | |
|---|---|---|---|---|---|
| cg11143148 | TTC7B | TSS1500 | S_Shore | -1.26E-02 | 3.17E-15 |
| cg11145108 | SRRM3 | Body | S_Shore | -7.37E-03 | 1.58E-05 |
| cg11145399 | TP53BP2;TP53BP2 | TSS1500;TSS1500 | S_Shore | -1.40E-02 | 7.27E-12 |
| cg11145826 | HDDC3;UNC45A;UN | TSS1500;5'UTR;TSS: | N_Shore | -9.91E-03 | 9.61E-13 |
| cg11154829 | SYT10 | Body | Island | 7.40E-03 | 4.79E-06 |
| cg11156891 | NA | NA | S_Shelf | -1.66E-02 | 5.69E-05 |
| cg11171221 | NA | NA | S_Shore | 1.47E-02 | 3.24E-07 |
| cg11174088 | NA | NA | NA | -9.57E-03 | 5.53E-10 |
| cg11176990 | LOC375196;LOC100 | TSS200;Body | Island | 2.76E-02 | 2.07E-12 |
| cg11184748 | NA | NA | Island | 7.81E-03 | 9.33E-05 |
| cg11199046 | NA | NA | Island | 1.31E-02 | 3.24E-10 |
| cg11202265 | PTPRN2;PTPRN2;PTI | Body;Body;Body | Island | 1.04E-02 | 9.53E-05 |
| cg11202634 | NA | NA | NA | -1.44E-02 | 5.50E-17 |
| cg11203512 | CCDC67 | 5'UTR | S_Shore | 2.03E-02 | 2.45E-07 |
| cg11204212 | NA | NA | Island | 8.54E-03 | 1.60E-05 |
| cg11206167 | NA | NA | Island | 3.51E-02 | 4.71E-06 |
| cg11208165 | OXNAD1 | 5'UTR | S_Shore | -1.54E-02 | 3.06E-17 |
| cg11214555 | NPHS1 | Body | Island | 5.68E-03 | 1.56E-05 |
| cg11215976 | ACTN2;ACTN2 | 1stExon;5'UTR | Island | 1.59E-02 | 2.75E-06 |
| cg11218561 | NA | NA | NA | -2.14E-02 | 1.86E-08 |
| cg11220950 | SYNGR3 | Body | Island | 1.15E-02 | 1.99E-22 |
| cg11229185 | NA | NA | Island | 1.29E-02 | 4.42E-07 |
| cg11229663 | PRKG1;PRKG1 | Body;Body | NA | -1.88E-02 | 2.76E-07 |
| cg11230112 | SYNJ2 | Body | NA | -1.07E-02 | 9.11E-06 |
| cg11235015 | SASH1 | Body | NA | -1.69E-02 | 5.98E-23 |
| cg11237573 | KPNA6 | Body | S_Shore | -1.05E-02 | 8.84E-08 |
| cg11249283 | JAK3 | TSS1500 | S_Shore | -8.76E-03 | 2.55E-07 |
| cg11253886 | NA | NA | NA | -1.40E-02 | 5.43E-16 |
| cg11254700 | NA | NA | Island | 2.70E-02 | 6.29E-16 |
| cg11260097 | NA | NA | Island | 7.03E-03 | 1.92E-05 |
| cg11279021 | ETV1;ETV1;ETV1;ET\ | 5'UTR;5'UTR;5'UTR; | N_Shore | 1.45E-02 | 2.89E-12 |
| cg11280525 | LOC645323 | Body | Island | 8.33E-03 | 1.83E-05 |
| cg11284842 | NA | NA | S_Shore | 1.87E-02 | 1.36E-06 |
| cg11291003 | NA | NA | N_Shore | -2.50E-02 | 1.71E-11 |
| cg11296759 | CD163L1 | Body | Island | 1.60E-02 | 3.78E-09 |
| cg11300147 | CD163L1 | 1stExon | S_Shelf | -1.87E-02 | 6.87E-10 |
| cg11300777 | MYO16 | Body | NA | -1.64E-02 | 1.28E-12 |
| cg11304682 | PDZRN3 | Body | NA | -1.17E-02 | 1.48E-14 |
| cg11310639 | NA | NA | NA | -1.59E-02 | 4.54E-19 |
| cg11322252 | NA | NA | Island | 8.80E-03 | 7.61E-08 |
| cg11331344 | RECQL5;LOC643008 | Body;TSS200 | S_Shore | -7.98E-03 | 6.02E-09 |
| cg11342437 | LRCH1;LRCH1;LRCH: | TSS1500;TSS1500;T | N_Shore | -1.81E-02 | 8.75E-17 |
| cg11354629 | GSX1 | TSS200 | Island | 1.42E-02 | 3.99E-05 |
| cg11359984 | MYLK;MYLK;MYLK;N | TSS1500;TSS1500;T | Island | 7.90E-03 | 5.76E-05 |
| cg11362013 | NA | NA | NA | 2.00E-02 | 6.79E-06 |
| cg11365617 | MGC14436;MGC14 | Body;Body;5'UTR | NA | 1.56E-02 | 1.39E-07 |
| cg11367633 | CHD6 | TSS1500 | S_Shore | -6.99E-03 | 6.64E-05 |
| cg11375072 | GHRH | TSS200 | NA | -9.71E-03 | 7.90E-08 |
| cg11377213 | FRMD4B | Body | NA | -1.02E-02 | 4.04E-08 |
| cg11382055 | KCNK1 | TSS1500 | Island | 1.57E-02 | 1.48E-07 |

| | | | | | |
|---|---|---|---|---|---|
| cg11386711 | ST6GAL1;ST6GAL1 | 5'UTR;5'UTR | S_Shelf | -1.06E-02 | 1.58E-08 |
| cg11391828 | KCNE4 | TSS1500 | N_Shelf | -7.81E-03 | 3.52E-07 |
| cg11396628 | PSORS1C1 | 5'UTR | NA | -1.24E-02 | 9.30E-10 |
| cg11397301 | SCG5;SCG5 | TSS1500;TSS1500 | NA | -7.72E-03 | 2.62E-05 |
| cg11397370 | GRHL2 | TSS200 | Island | 7.26E-03 | 4.46E-05 |
| cg11412248 | CHRNB4 | Body | N_Shelf | -1.58E-02 | 1.83E-15 |
| cg11413039 | PUS3;DDX25 | TSS1500;1stExon | Island | 1.32E-02 | 9.96E-05 |
| cg11414560 | PHACTR3;PHACTR3 | 5'UTR;1stExon | Island | 1.42E-02 | 1.09E-05 |
| cg11419186 | CMIP | TSS1500 | N_Shore | -5.59E-03 | 2.10E-07 |
| cg11436113 | NA | NA | N_Shore | -9.74E-03 | 1.48E-11 |
| cg11437140 | TIMP2 | Body | S_Shelf | -1.61E-02 | 1.25E-11 |
| cg11439393 | BRD2;BRD2 | Body;Body | S_Shore | -1.67E-02 | 6.44E-11 |
| cg11441704 | PDXK | Body | N_Shelf | 4.82E-03 | 5.87E-07 |
| cg11442608 | NA | NA | NA | -8.78E-03 | 1.09E-07 |
| cg11453058 | PODNL1;PODNL1;D( | 5'UTR;1stExon;Body | S_Shore | -7.64E-03 | 7.12E-06 |
| cg11468819 | NA | NA | NA | 8.65E-03 | 9.37E-06 |
| cg11479156 | SHANK2 | Body | N_Shore | -1.29E-02 | 1.07E-05 |
| cg11494841 | C17orf62;C17orf62; | Body;Body;Body | Island | -1.02E-02 | 6.05E-06 |
| cg11501236 | LOC134466 | Body | Island | 1.45E-02 | 2.28E-11 |
| cg11502555 | NA | NA | S_Shelf | 1.32E-02 | 2.10E-08 |
| cg11526866 | CAMK2N1 | TSS1500 | S_Shore | 7.16E-03 | 1.87E-07 |
| cg11529128 | LHFPL5 | TSS200 | N_Shore | 1.06E-02 | 7.70E-10 |
| cg11545457 | NA | NA | N_Shelf | -1.02E-02 | 2.30E-13 |
| cg11546137 | HOXD11 | 1stExon | N_Shore | 1.97E-02 | 4.26E-05 |
| cg11554650 | KIAA1949;KIAA1949 | Body;1stExon | N_Shore | -7.66E-03 | 1.47E-08 |
| cg11556592 | C17orf55 | TSS1500 | N_Shore | -9.00E-03 | 1.44E-05 |
| cg11573518 | CLIP2;CLIP2 | 5'UTR;5'UTR | S_Shore | -1.82E-02 | 3.92E-23 |
| cg11581706 | CACNA2D1 | Body | Island | 1.71E-02 | 1.16E-09 |
| cg11582456 | SEMA6A | 5'UTR | Island | 5.97E-03 | 4.76E-05 |
| cg11587584 | HLA-F;HLA-F;HLA-F | Body;Body;Body | S_Shore | -8.08E-03 | 3.13E-07 |
| cg11594821 | HLA-E | Body | S_Shore | -1.16E-02 | 5.73E-10 |
| cg11595059 | DOCK6 | Body | S_Shore | -1.04E-02 | 5.08E-06 |
| cg11595270 | SCN10A | Body | NA | -8.34E-03 | 1.71E-06 |
| cg11606570 | CFTR | Body | NA | -1.41E-02 | 2.96E-11 |
| cg11611097 | NA | NA | NA | -1.11E-02 | 1.59E-13 |
| cg11612647 | NA | NA | NA | -1.29E-02 | 5.69E-10 |
| cg11616547 | NA | NA | Island | 7.49E-03 | 9.61E-05 |
| cg11617938 | HLA-F;HLA-F;HLA-F | Body;Body;Body | S_Shore | -1.06E-02 | 6.62E-09 |
| cg11618529 | ZNF132 | TSS1500 | Island | 8.58E-03 | 5.38E-07 |
| cg11624524 | NEURL1B | Body | Island | 2.81E-02 | 4.95E-07 |
| cg11642324 | GOLGA3 | 3'UTR | N_Shore | 1.21E-02 | 2.52E-06 |
| cg11649376 | ACSS3 | Body | S_Shore | -8.85E-03 | 4.09E-08 |
| cg11654813 | NA | NA | N_Shelf | -3.37E-02 | 7.93E-05 |
| cg11659663 | MACROD1 | Body | S_Shore | -7.15E-03 | 7.47E-06 |
| cg11661534 | NA | NA | N_Shore | -1.09E-02 | 2.34E-05 |
| cg11667020 | NKX2-4;NKX2-4 | 5'UTR;1stExon | Island | 3.28E-02 | 2.14E-10 |
| cg11667754 | CDH1 | TSS1500 | N_Shore | 8.27E-03 | 4.49E-05 |
| cg11671688 | GPR6 | 1stExon | Island | 1.44E-02 | 7.78E-06 |
| cg11685394 | PPFIA3 | Body | Island | 1.40E-02 | 7.98E-05 |
| cg11693709 | PAK6;PAK6;PAK6 | 5'UTR;5'UTR;5'UTR | N_Shelf | -1.56E-02 | 1.09E-18 |

| | | | | | |
|---|---|---|---|---|---|
| cg11703632 | NA | NA | Island | 8.49E-03 | 2.32E-07 |
| cg11705975 | PRLHR | Body | Island | 1.03E-02 | 1.15E-09 |
| cg11726250 | COL20A1 | TSS200 | N_Shelf | -1.48E-02 | 1.55E-08 |
| cg11730703 | INF2;INF2;INF2 | 5'UTR;5'UTR;5'UTR | N_Shore | -1.71E-02 | 6.14E-12 |
| cg11736940 | PKLR;PKLR | Body;Body | Island | 5.97E-03 | 3.75E-06 |
| cg11741201 | FJX1 | TSS1500 | N_Shore | -1.27E-02 | 7.93E-08 |
| cg11743349 | KCNIP4 | TSS1500 | Island | 9.66E-03 | 8.20E-08 |
| cg11747183 | NA | NA | Island | 1.92E-02 | 2.71E-13 |
| cg11751101 | C1R | Body | NA | -1.26E-02 | 3.08E-14 |
| cg11784243 | LRRC16A | Body | NA | -2.26E-02 | 2.84E-08 |
| cg11791577 | KCNA6 | 3'UTR | NA | -2.58E-02 | 1.76E-14 |
| cg11797365 | COL4A3;COL4A3;CO | Body;Body;TSS1500 | Island | 1.26E-02 | 3.52E-10 |
| cg11807280 | NA | NA | S_Shore | -3.78E-02 | 1.92E-14 |
| cg11826475 | PXN;PXN | TSS1500;TSS1500 | S_Shore | -5.61E-03 | 3.33E-06 |
| cg11830800 | CNTN1;CNTN1 | 5'UTR;5'UTR | S_Shore | -1.50E-02 | 5.37E-07 |
| cg11831238 | DBX1 | TSS200 | Island | 2.14E-02 | 5.30E-14 |
| cg11831981 | SCG3;SCG3;SCG3;SC | 5'UTR;1stExon;5'UT | Island | 3.35E-02 | 2.11E-07 |
| cg11836829 | NENF;NENF | TSS1500;TSS1500 | N_Shore | -1.15E-02 | 5.17E-06 |
| cg11839291 | NA | NA | Island | 1.08E-02 | 1.21E-05 |
| cg11857704 | NA | NA | Island | 2.07E-02 | 3.34E-08 |
| cg11866756 | NA | NA | N_Shore | -1.08E-02 | 1.71E-11 |
| cg11870100 | ELF5;ELF5 | 5'UTR;TSS1500 | NA | -8.05E-03 | 3.33E-05 |
| cg11883836 | NA | NA | Island | 1.38E-02 | 8.96E-06 |
| cg11891377 | LRRC56 | 3'UTR | Island | -6.17E-03 | 1.03E-06 |
| cg11918450 | LTBP1;LTBP1;LTBP1; | Body;TSS1500;TSS1 | NA | -8.18E-03 | 4.47E-07 |
| cg11919694 | TBC1D10B | 1stExon | N_Shore | -1.02E-02 | 2.03E-07 |
| cg11920122 | GNAL;GNAL;GNAL | TSS200;Body;TSS20 | Island | 8.53E-03 | 4.80E-07 |
| cg11930114 | LOC645323;LOC645 | Body;Body | N_Shore | 2.01E-02 | 7.67E-09 |
| cg11930955 | TPCN1;TPCN1;IQCD | 5'UTR;5'UTR;TSS15( | S_Shore | -8.19E-03 | 7.83E-09 |
| cg11942910 | TTLL2 | TSS200 | NA | -6.38E-03 | 7.58E-06 |
| cg11946449 | SYT13 | TSS1500 | S_Shore | -8.96E-03 | 4.65E-09 |
| cg11946459 | HLA-A | Body | S_Shore | -2.57E-02 | 1.09E-05 |
| cg11949874 | NA | NA | NA | -9.08E-03 | 1.20E-08 |
| cg11961138 | IGFBP4 | TSS1500 | Island | -8.12E-03 | 3.15E-05 |
| cg11961813 | PSME3;PSME3;AOC: | 3'UTR;3'UTR;TSS15( | NA | -1.27E-02 | 2.29E-11 |
| cg11964364 | COL12A1;COL12A1 | Body;Body | NA | -1.58E-02 | 1.36E-10 |
| cg11970349 | GPR78 | TSS200 | Island | 1.19E-02 | 2.65E-17 |
| cg11977605 | RNF212;RNF212 | Body;Body | NA | -5.33E-03 | 9.07E-05 |
| cg11980800 | PIK3CD | 5'UTR | N_Shore | -1.53E-02 | 9.10E-08 |
| cg11982293 | NA | NA | Island | 8.10E-03 | 6.90E-06 |
| cg12001304 | SPHKAP;SPHKAP | Body;Body | N_Shore | 1.27E-02 | 4.91E-05 |
| cg12004641 | TNS1 | Body | NA | -1.11E-02 | 9.67E-09 |
| cg12007399 | ZFR2;ZFR2;ZFR2 | Body;Body;Body | Island | 8.21E-03 | 1.83E-05 |
| cg12014646 | SEMA6D;SEMA6D;S | 5'UTR;5'UTR;5'UTR; | S_Shelf | -1.39E-02 | 4.86E-16 |
| cg12018863 | BBS1 | TSS1500 | NA | -9.48E-03 | 2.33E-07 |
| cg12025243 | ODZ4 | Body | NA | -6.71E-03 | 3.40E-05 |
| cg12034118 | IRF6 | TSS200 | Island | 2.73E-02 | 5.67E-05 |
| cg12036303 | KIAA1949;KIAA1949 | Body;1stExon | N_Shore | -9.85E-03 | 4.72E-10 |
| cg12042187 | NA | NA | N_Shore | -1.26E-02 | 7.32E-06 |
| cg12042952 | AFF3;AFF3 | TSS1500;5'UTR | S_Shelf | -9.82E-03 | 4.30E-08 |

| | | | | | |
|---|---|---|---|---|---|
| cg12049236 | NA | NA | S_Shore | 1.13E-02 | 1.37E-06 |
| cg12052661 | CACNA1B | 1stExon | Island | 8.60E-03 | 1.22E-11 |
| cg12065138 | AJAP1;AJAP1 | Body;Body | N_Shore | 8.81E-03 | 1.72E-06 |
| cg12065799 | RRAGC | Body | N_Shore | -8.35E-03 | 7.71E-05 |
| cg12068280 | HIF3A;HIF3A | 5'UTR;Body | N_Shelf | -8.02E-03 | 1.33E-07 |
| cg12068816 | FAM83F | TSS1500 | Island | 8.67E-03 | 3.85E-05 |
| cg12068916 | MTMR7 | Body | N_Shelf | -1.18E-02 | 5.68E-06 |
| cg12071008 | DNAH17 | Body | S_Shelf | -6.35E-03 | 3.35E-06 |
| cg12071888 | NA | NA | S_Shelf | 1.10E-02 | 2.79E-06 |
| cg12075928 | PTK2;PTK2 | Body;Body | NA | -2.00E-02 | 2.59E-09 |
| cg12079303 | NFIA;NFIA;NFIA;NFI/ | Body;TSS1500;TSS1 | N_Shore | -1.65E-02 | 1.65E-17 |
| cg12081325 | EIF2B3;EIF2B3 | Body;Body | NA | -1.72E-02 | 4.59E-13 |
| cg12089570 | ZNF177;ZNF177 | 1stExon;5'UTR | Island | 1.04E-02 | 3.25E-09 |
| cg12096759 | GLRX5;SNHG10;SNH | Body;TSS1500;TSS1 | S_Shore | -1.28E-02 | 7.03E-10 |
| cg12105190 | KIAA1949;KIAA1949 | Body;1stExon | N_Shore | -8.04E-03 | 5.41E-08 |
| cg12116137 | PRPF8 | Body | NA | 1.01E-02 | 8.03E-05 |
| cg12121318 | GRK4;GRK4;GRK4;N | TSS1500;TSS1500;T | N_Shore | -1.05E-02 | 7.03E-05 |
| cg12138450 | NA | NA | NA | -9.65E-03 | 2.28E-07 |
| cg12145394 | RMND5B | Body | NA | -7.24E-03 | 3.73E-06 |
| cg12146673 | SST | Body | N_Shore | 1.30E-02 | 3.98E-07 |
| cg12147105 | PPA2;PPA2;PPA2;PF | TSS1500;TSS1500;T | S_Shore | -1.14E-02 | 3.32E-08 |
| cg12147622 | NA | NA | S_Shore | -1.43E-02 | 1.55E-13 |
| cg12155547 | NA | NA | S_Shelf | -1.57E-02 | 2.84E-10 |
| cg12159215 | UPK2 | TSS1500 | NA | -5.63E-03 | 1.19E-05 |
| cg12160664 | OLFML3 | TSS200 | NA | -9.05E-03 | 2.41E-05 |
| cg12161228 | NOX4;NOX4;NOX4;N | 1stExon;1stExon;5'U | Island | 2.12E-02 | 1.48E-05 |
| cg12161971 | USP35 | Body | Island | 3.13E-02 | 1.76E-10 |
| cg12163871 | PRKD2;PRKD2;PRKD | Body;Body;Body;Bo | Island | -4.79E-03 | 1.97E-05 |
| cg12164596 | MNX1;MNX1 | Body;Body | Island | 8.30E-03 | 2.97E-10 |
| cg12166476 | LMO7;LMO7;LMO7 | 5'UTR;1stExon;Body | NA | -1.83E-02 | 7.67E-14 |
| cg12168357 | ARHGEF1;ARHGEF1; | 3'UTR;3'UTR;3'UTR | N_Shore | -5.35E-03 | 9.70E-05 |
| cg12174804 | NA | NA | Island | 1.46E-02 | 1.11E-05 |
| cg12177113 | AFAP1L2;AFAP1L2 | Body;Body | NA | -8.79E-03 | 2.42E-06 |
| cg12179661 | ENTPD8;ENTPD8 | 5'UTR;5'UTR | N_Shore | -8.89E-03 | 5.47E-12 |
| cg12184221 | TMEM39A | TSS1500 | NA | -1.42E-02 | 1.93E-06 |
| cg12188986 | CCDC67;CCDC67 | 1stExon;5'UTR | Island | 1.77E-02 | 5.76E-05 |
| cg12189835 | SYT7 | Body | N_Shore | 7.95E-03 | 7.68E-10 |
| cg12190841 | RARA;RARA;RARA;R | Body;Body;Body;Bo | NA | -7.69E-03 | 2.51E-09 |
| cg12190994 | SDK1;SDK1 | Body;3'UTR | Island | 1.38E-02 | 1.17E-08 |
| cg12196232 | NRG1;NRG1;NRG1;N | Body;Body;Body;Bo | NA | 9.02E-03 | 1.77E-07 |
| cg12197457 | SLC22A1;SLC22A1 | Body;Body | Island | 1.22E-02 | 2.43E-05 |
| cg12200038 | NA | NA | Island | 2.88E-02 | 5.94E-06 |
| cg12206199 | LOC375196;LOC100 | TSS200;Body | Island | 4.44E-02 | 4.00E-14 |
| cg12227172 | ZNF577;ZNF577;ZNF | TSS1500;TSS200;TS | S_Shore | 2.22E-02 | 9.25E-07 |
| cg12227907 | PRDM13 | Body | Island | 8.34E-03 | 9.97E-08 |
| cg12233363 | SIM1 | TSS1500 | Island | 1.03E-02 | 5.63E-06 |
| cg12235572 | NA | NA | NA | -6.42E-03 | 1.23E-05 |
| cg12247101 | KIAA1949;KIAA1949 | Body;1stExon | N_Shore | -1.54E-02 | 4.89E-11 |
| cg12248614 | SPTBN4 | Body | Island | 2.89E-02 | 8.39E-07 |
| cg12251075 | NME2;NME2;NME2 | Body;Body;Body;Bo | S_Shore | -1.03E-02 | 6.38E-08 |

| | | | | | |
|---|---|---|---|---|---|
| cg12252865 | HDAC11;HDAC11 | TSS1500;TSS1500 | N_Shore | -1.18E-02 | 7.89E-09 |
| cg12258344 | NA | NA | NA | -6.54E-03 | 1.74E-09 |
| cg12263377 | NA | NA | NA | -1.10E-02 | 2.93E-11 |
| cg12269535 | SRF | Body | Island | -6.24E-03 | 3.01E-05 |
| cg12274898 | S100A16 | TSS1500 | NA | -8.43E-03 | 6.61E-05 |
| cg12278474 | NA | NA | NA | -1.54E-02 | 2.18E-10 |
| cg12284971 | EFHD1 | TSS1500 | N_Shore | -6.32E-03 | 2.01E-05 |
| cg12289553 | ASAH2B | 5'UTR | S_Shore | -1.75E-02 | 1.33E-12 |
| cg12296772 | MTMR7 | TSS200 | Island | 1.62E-02 | 8.00E-06 |
| cg12300353 | KCTD8 | 1stExon | Island | 1.25E-02 | 1.25E-05 |
| cg12303084 | ZMYND8;ZMYND8;Z | TSS1500;TSS1500;T | NA | -7.51E-03 | 2.45E-05 |
| cg12321669 | HLA-C | TSS1500 | S_Shore | -2.56E-02 | 3.56E-06 |
| cg12325536 | LOC645323;LOC645 | Body;Body;Body | Island | 1.60E-02 | 1.42E-07 |
| cg12334759 | ODF3L2 | TSS200 | NA | -8.85E-03 | 2.27E-08 |
| cg12343106 | NA | NA | Island | 1.29E-02 | 3.22E-07 |
| cg12346592 | NA | NA | Island | 2.29E-02 | 3.95E-06 |
| cg12348202 | PTPRN2;PTPRN2;PTI | TSS1500;TSS1500;T | Island | 1.19E-02 | 1.54E-07 |
| cg12349416 | NA | NA | N_Shelf | -9.65E-03 | 6.85E-08 |
| cg12350474 | NA | NA | NA | -1.22E-02 | 9.46E-12 |
| cg12363722 | MTNR1A | TSS200 | Island | 2.85E-02 | 5.85E-05 |
| cg12363903 | NA | NA | N_Shore | -2.66E-02 | 1.71E-14 |
| cg12371924 | BCAT1 | TSS200 | S_Shore | -1.64E-02 | 7.51E-10 |
| cg12373771 | CECR6;CECR6 | 1stExon;5'UTR | Island | 9.00E-03 | 2.57E-07 |
| cg12377139 | SPAG6;SPAG6 | TSS200;TSS200 | Island | 2.23E-02 | 1.87E-12 |
| cg12379383 | NA | NA | Island | 1.40E-02 | 1.33E-09 |
| cg12382398 | ANPEP | TSS1500 | Island | 9.87E-03 | 5.13E-05 |
| cg12393318 | LOC645323 | TSS1500 | Island | 1.22E-02 | 6.85E-05 |
| cg12407057 | NA | NA | S_Shelf | -1.11E-02 | 5.06E-05 |
| cg12412079 | FAM19A4;FAM19A4 | TSS200;TSS200 | Island | 1.92E-02 | 4.99E-07 |
| cg12422450 | CHGA | Body | Island | 1.32E-02 | 1.96E-10 |
| cg12426313 | PLEKHG5;PLEKHG5;I | Body;Body;TSS150C | S_Shore | -8.60E-03 | 1.06E-07 |
| cg12426748 | NA | NA | N_Shore | 1.66E-02 | 8.57E-11 |
| cg12432980 | CRLS1;CRLS1 | Body;TSS200 | S_Shore | -1.26E-02 | 9.47E-10 |
| cg12441967 | CECR6;CECR6 | TSS1500;TSS1500 | Island | 9.78E-03 | 3.84E-07 |
| cg12446246 | PLXNA2 | TSS1500 | S_Shore | -7.43E-03 | 5.98E-07 |
| cg12453631 | NA | NA | Island | 1.32E-02 | 2.19E-12 |
| cg12457773 | NRSN1;NRSN1 | 5'UTR;1stExon | Island | 9.45E-03 | 4.20E-09 |
| cg12467223 | ROR1;ROR1 | Body;Body | NA | -7.28E-03 | 3.90E-06 |
| cg12467749 | TBX20;TBX20 | TSS1500;TSS1500 | Island | 1.17E-02 | 5.09E-06 |
| cg12468478 | TCF21;TCF21;TCF21 | 1stExon;5'UTR;1stE | N_Shore | 1.30E-02 | 4.54E-08 |
| cg12483947 | SGPL1 | 3'UTR | NA | -1.19E-02 | 5.51E-06 |
| cg12486710 | C1orf220;C1orf220 | Body;5'UTR | S_Shore | -5.54E-03 | 2.45E-05 |
| cg12492496 | PIK3R2 | Body | Island | -8.78E-03 | 2.17E-06 |
| cg12500956 | TMEM163 | Body | NA | -1.00E-02 | 7.48E-05 |
| cg12502460 | WWC2 | Body | NA | -9.34E-03 | 3.09E-09 |
| cg12508451 | LOC145845 | Body | Island | 6.94E-03 | 2.51E-07 |
| cg12516875 | NA | NA | N_Shelf | -1.07E-02 | 8.44E-06 |
| cg12523924 | NA | NA | Island | 1.49E-02 | 8.80E-07 |
| cg12530503 | MIR9-3 | TSS200 | Island | 8.00E-03 | 2.96E-09 |
| cg12533658 | STARD3;STARD3;ST/ | Body;Body;Body | NA | -5.84E-03 | 1.82E-05 |

| | | | | | |
|---|---|---|---|---|---|
| cg12534424 | PRRT4 | Body | Island | 8.30E-03 | 3.83E-13 |
| cg12554573 | PARP3;RRP9;PARP3; | 5'UTR;TSS1500;1stE | S_Shore | -6.70E-03 | 7.11E-06 |
| cg12580096 | C19orf57;CC2D1A | TSS1500;Body | S_Shore | -7.74E-03 | 2.12E-05 |
| cg12581769 | CACNA1A;CACNA1A | Body;Body | S_Shelf | -9.32E-03 | 2.24E-14 |
| cg12596182 | BEST3;BEST3;BEST3 | Body;5'UTR;1stExon | NA | -9.96E-03 | 1.07E-08 |
| cg12597389 | NXPH1 | Body | Island | 1.36E-02 | 3.08E-07 |
| cg12598235 | LOC254559 | TSS200 | Island | 1.08E-02 | 5.13E-06 |
| cg12598865 | NA | NA | N_Shelf | -1.02E-02 | 2.36E-11 |
| cg12603632 | NA | NA | NA | -1.42E-02 | 1.95E-07 |
| cg12605148 | HERC2 | 3'UTR | Island | -1.17E-02 | 5.05E-05 |
| cg12608507 | EEF1B2;EEF1B2;SNC | Body;Body;TSS1500 | S_Shore | -1.24E-02 | 3.48E-08 |
| cg12610471 | SPAG6;SPAG6 | TSS200;TSS200 | Island | 1.63E-02 | 3.06E-08 |
| cg12615982 | TERC | TSS1500 | S_Shore | -1.08E-02 | 5.79E-06 |
| cg12616487 | ROM1;EML3 | TSS1500;Body | N_Shore | -5.60E-03 | 2.91E-05 |
| cg12617928 | NA | NA | Island | -1.32E-02 | 1.10E-06 |
| cg12619162 | FXYD4 | 5'UTR | NA | -6.26E-03 | 1.23E-05 |
| cg12619504 | SQSTM1;MGAT4B;S | TSS1500;Body;TSS1 | Island | -7.87E-03 | 1.14E-06 |
| cg12623930 | ABHD14B;ABHD14B | TSS200;TSS200;TSS: | N_Shore | -8.96E-03 | 6.04E-11 |
| cg12626956 | FOXB2 | TSS200 | Island | 7.29E-03 | 3.59E-06 |
| cg12627844 | VPS54;VPS54 | 5'UTR;5'UTR | N_Shore | -2.89E-02 | 1.82E-16 |
| cg12629349 | EML6 | Body | S_Shore | -1.74E-02 | 1.21E-06 |
| cg12636169 | ESRRG | 5'UTR | N_Shore | 8.99E-03 | 1.87E-05 |
| cg12644285 | CHD2 | 3'UTR | NA | 6.90E-03 | 5.59E-05 |
| cg12658720 | C17orf82 | TSS1500 | Island | 2.94E-02 | 7.69E-08 |
| cg12667125 | C1orf216 | Body | N_Shelf | -1.15E-02 | 1.83E-16 |
| cg12686055 | ANO6;ANO6;ANO6 | Body;Body;Body | NA | -1.67E-02 | 1.40E-12 |
| cg12686273 | CCDC64B | TSS1500 | N_Shore | -1.41E-02 | 1.27E-15 |
| cg12688542 | NCOR2;NCOR2 | Body;Body | N_Shelf | -1.41E-02 | 4.93E-13 |
| cg12693641 | SYT7 | TSS1500 | S_Shore | -6.69E-03 | 3.32E-07 |
| cg12711760 | NAV2;NAV2;NAV2;N | Body;Body;Body;Bo | NA | -1.49E-02 | 1.01E-14 |
| cg12715421 | DDC;DDC | 5'UTR;TSS1500 | NA | -1.01E-02 | 2.12E-12 |
| cg12717533 | FAM59B | Body | Island | 7.26E-03 | 6.09E-06 |
| cg12718579 | ST18 | TSS200 | NA | -1.27E-02 | 1.83E-08 |
| cg12723492 | KIRREL3;KIRREL3 | Body;Body | NA | -6.72E-03 | 7.84E-07 |
| cg12738913 | SNTG2 | Body | S_Shelf | -1.01E-02 | 7.71E-06 |
| cg12739664 | C17orf58;C17orf58 | TSS1500;TSS1500 | S_Shore | -1.49E-02 | 1.55E-11 |
| cg12742799 | NA | NA | Island | 9.48E-03 | 1.72E-09 |
| cg12744812 | NA | NA | Island | 1.16E-02 | 2.79E-05 |
| cg12744820 | OLIG3 | 1stExon | Island | 2.07E-02 | 1.33E-08 |
| cg12756396 | DMRTA2 | Body | Island | 1.22E-02 | 3.45E-06 |
| cg12757011 | TBR1 | 3'UTR | S_Shore | 7.99E-03 | 2.15E-08 |
| cg12758687 | DRD2;DRD2 | TSS1500;TSS1500 | Island | 9.96E-03 | 2.34E-11 |
| cg12764034 | LHX8 | Body | Island | 1.37E-02 | 4.20E-06 |
| cg12765028 | NA | NA | Island | 7.64E-03 | 4.27E-06 |
| cg12781700 | C17orf104 | TSS200 | Island | 2.36E-02 | 4.80E-07 |
| cg12793711 | ACTG1 | Body | N_Shore | -1.09E-02 | 4.31E-07 |
| cg12795893 | ATP5B;SNORD59B | Body;TSS1500 | N_Shore | -1.26E-02 | 6.70E-11 |
| cg12798194 | NA | NA | NA | -6.86E-03 | 1.09E-05 |
| cg12807924 | SRC;SRC | Body;Body | S_Shore | -4.32E-03 | 3.98E-05 |
| cg12831076 | ST3GAL1;ST3GAL1 | 5'UTR;5'UTR | N_Shore | -7.52E-03 | 1.39E-05 |

| | | | | | |
|---|---|---|---|---|---|
| cg12834820 | ADAM11 | Body | N_Shore | -5.82E-03 | 4.23E-05 |
| cg12836959 | NA | NA | NA | 2.11E-02 | 3.45E-09 |
| cg12839156 | NA | NA | NA | -1.89E-02 | 2.07E-09 |
| cg12848065 | LOC200726 | 5'UTR | S_Shore | 1.71E-02 | 6.28E-11 |
| cg12848614 | NA | NA | Island | -1.12E-02 | 1.87E-06 |
| cg12854186 | TAP2;TAP2 | Body;Body | N_Shore | -1.68E-02 | 6.54E-19 |
| cg12856708 | NA | NA | NA | -1.37E-02 | 5.03E-13 |
| cg12862231 | CSF1R | 5'UTR | NA | -5.74E-03 | 2.93E-05 |
| cg12873476 | NA | NA | S_Shore | -8.22E-03 | 5.92E-06 |
| cg12873919 | BCL2L1;BCL2L1 | Body;Body | N_Shore | -1.80E-02 | 1.34E-18 |
| cg12878812 | SRRM4 | 1stExon | Island | 1.17E-02 | 3.43E-16 |
| cg12892303 | C17orf104 | TSS200 | Island | 2.27E-02 | 2.38E-09 |
| cg12899747 | NA | NA | NA | -3.03E-02 | 4.68E-18 |
| cg12910906 | ARHGEF4;ARHGEF4 | TSS1500;TSS1500 | Island | 1.08E-02 | 9.45E-06 |
| cg12915947 | TUBB | Body | S_Shelf | -4.21E-03 | 1.66E-05 |
| cg12918213 | MED13L | Body | NA | -2.83E-02 | 3.59E-19 |
| cg12920180 | COCH;COCH | Body;Body | Island | 2.18E-02 | 9.14E-07 |
| cg12928668 | SEMA6A | TSS200 | Island | 1.01E-02 | 3.62E-09 |
| cg12939283 | HLA-DPB1 | Body | NA | -2.80E-02 | 2.58E-17 |
| cg12941369 | PDCD6IP;PDCD6IP;P | TSS1500;TSS1500;T | N_Shore | -1.27E-02 | 8.84E-07 |
| cg12943155 | PODXL2 | TSS200 | Island | 2.36E-02 | 1.72E-11 |
| cg12951726 | SLC24A6 | TSS1500 | S_Shore | -1.22E-02 | 3.33E-07 |
| cg12969644 | RASEF | TSS200 | Island | 1.04E-02 | 2.72E-05 |
| cg12970084 | NA | NA | NA | -8.89E-03 | 5.77E-08 |
| cg12974223 | NA | NA | NA | -1.24E-02 | 2.78E-11 |
| cg12984877 | ZNF827 | Body | N_Shore | -1.82E-02 | 1.24E-19 |
| cg12995800 | GLRA1;GLRA1;GLRA | 1stExon;1stExon;5'U | Island | 2.17E-02 | 3.60E-07 |
| cg13006424 | TMEM131 | Body | NA | -9.05E-03 | 7.33E-06 |
| cg13007871 | HLA-E | Body | S_Shore | -1.16E-02 | 4.78E-10 |
| cg13016408 | LYPD1;LYPD1 | 5'UTR;Body | N_Shore | 7.22E-03 | 3.40E-05 |
| cg13016524 | NA | NA | Island | 7.14E-03 | 1.18E-07 |
| cg13021857 | NA | NA | NA | -1.94E-02 | 2.46E-10 |
| cg13026773 | FAM108C1 | TSS1500 | N_Shore | -1.16E-02 | 4.61E-07 |
| cg13029847 | SEZ6;SEZ6 | TSS200;TSS200 | S_Shore | 7.96E-03 | 3.20E-05 |
| cg13033938 | IP6K1;IP6K1 | TSS1500;TSS1500 | S_Shore | -9.93E-03 | 4.59E-05 |
| cg13036878 | NA | NA | S_Shelf | 1.12E-02 | 4.57E-05 |
| cg13039251 | PDZD2 | Body | NA | -2.56E-02 | 2.01E-22 |
| cg13054640 | KIAA1949;KIAA1949 | Body;Body | NA | -1.19E-02 | 2.63E-12 |
| cg13055199 | ULK3 | TSS1500 | S_Shore | -1.03E-02 | 3.07E-07 |
| cg13056653 | NA | NA | NA | -1.62E-02 | 1.66E-08 |
| cg13056927 | KBTBD5 | 1stExon | Island | 1.88E-02 | 2.20E-05 |
| cg13058623 | NA | NA | NA | -1.60E-02 | 8.45E-11 |
| cg13059495 | TPRG1L | TSS1500 | N_Shore | -1.71E-02 | 6.99E-16 |
| cg13066481 | MYLK;MYLK;MYLK;M | Body;Body;Body;Bo | NA | -8.17E-03 | 1.84E-11 |
| cg13067634 | MUTYH;MUTYH;MU | TSS1500;TSS1500;T | S_Shore | -1.46E-02 | 1.94E-07 |
| cg13069531 | CD86;CD86;CD86 | 5'UTR;Body;1stExor | NA | -1.03E-02 | 9.01E-05 |
| cg13072214 | PARP10 | TSS200 | N_Shelf | -1.03E-02 | 3.50E-08 |
| cg13072940 | MON1A;MON1A | TSS200;TSS200 | S_Shore | -6.86E-03 | 4.82E-06 |
| cg13072943 | RPS6KA2;RPS6KA2 | Body;Body | NA | -1.21E-02 | 1.16E-09 |
| cg13080565 | GRIK2;GRIK2;GRIK2 | TSS200;TSS200;TSS: | Island | 2.28E-02 | 9.32E-12 |

| | | | | | |
|---|---|---|---|---|---|
| cg13081213 | SKAP2 | Body | NA | -1.90E-02 | 2.30E-16 |
| cg13092901 | TYMP;SCO2;SCO2;T | Body;TSS1500;TSS1 | Island | -9.35E-03 | 4.73E-06 |
| cg13104274 | ATP1B1;ATP1B1 | Body;Body | S_Shore | -1.09E-02 | 4.06E-06 |
| cg13126871 | PF4 | TSS1500 | S_Shore | -1.30E-02 | 4.62E-06 |
| cg13127741 | COMMD7;COMMD7 | TSS200;TSS200 | S_Shore | -1.14E-02 | 1.39E-12 |
| cg13134802 | CACNA1G;CACNA1G | Body;Body;Body;3'U | S_Shelf | -6.58E-03 | 8.21E-05 |
| cg13134916 | IFLTD1;IFLTD1;IFLTD | TSS1500;TSS1500;T | NA | -1.04E-02 | 1.00E-05 |
| cg13135455 | NA | NA | S_Shore | -6.73E-03 | 6.24E-07 |
| cg13150596 | GIPC1;GIPC1;GIPC1; | 5'UTR;5'UTR;5'UTR; | N_Shore | -6.66E-03 | 4.36E-05 |
| cg13153540 | NA | NA | NA | -6.22E-03 | 5.55E-05 |
| cg13153834 | SUMF2;SUMF2;SUM | Body;Body;Body;Bo | NA | -8.76E-03 | 2.79E-08 |
| cg13155490 | AQP12B | TSS1500 | NA | -8.50E-03 | 8.63E-05 |
| cg13157483 | PKNOX2 | 5'UTR | NA | -7.24E-03 | 5.38E-07 |
| cg13159037 | NA | NA | NA | -6.98E-03 | 9.08E-06 |
| cg13161658 | KCNA4 | TSS1500 | Island | 1.38E-02 | 6.67E-10 |
| cg13167518 | NA | NA | Island | 9.07E-03 | 2.24E-05 |
| cg13167730 | NA | NA | Island | 7.86E-03 | 1.01E-05 |
| cg13171679 | NA | NA | NA | -7.17E-03 | 3.15E-06 |
| cg13176012 | NA | NA | Island | 8.52E-03 | 2.56E-09 |
| cg13197216 | NA | NA | Island | 9.28E-03 | 5.49E-07 |
| cg13203811 | AGAP2 | TSS1500 | S_Shelf | -7.43E-03 | 3.17E-09 |
| cg13206191 | NA | NA | NA | -1.15E-02 | 7.30E-05 |
| cg13212079 | SCAND3;SCAND3 | 1stExon;5'UTR | Island | 9.22E-03 | 1.65E-06 |
| cg13221458 | SOD2;SOD2;SOD2 | Body;Body;Body | N_Shore | -2.26E-02 | 5.30E-14 |
| cg13226308 | NA | NA | NA | -2.53E-02 | 9.88E-09 |
| cg13229360 | SH3PXD2B | TSS1500 | S_Shore | -8.94E-03 | 2.73E-10 |
| cg13234863 | TMEM132D | TSS1500 | Island | 8.89E-03 | 3.12E-06 |
| cg13246235 | PHACTR1 | Body | Island | 1.50E-02 | 3.70E-06 |
| cg13249876 | ARHGAP12 | TSS200 | Island | -4.87E-03 | 6.50E-05 |
| cg13269555 | KAZALD1 | 5'UTR | Island | -8.10E-03 | 1.15E-05 |
| cg13272108 | NOP56;SNORD110;N | Body;TSS200;Body;T | S_Shore | -1.83E-02 | 2.90E-13 |
| cg13272546 | SEZ6;SEZ6 | Body;Body | Island | 6.33E-03 | 2.32E-07 |
| cg13278052 | NA | NA | NA | -6.69E-03 | 2.87E-05 |
| cg13278478 | PHKG1 | TSS1500 | NA | -1.21E-02 | 3.59E-08 |
| cg13291394 | USP46 | TSS1500 | S_Shore | -1.20E-02 | 1.20E-08 |
| cg13298997 | PDE1C | Body | NA | -1.44E-02 | 1.62E-14 |
| cg13300580 | SLC9A1 | Body | NA | 4.96E-03 | 2.05E-05 |
| cg13319711 | NA | NA | Island | 7.03E-03 | 1.12E-06 |
| cg13320202 | ATXN7;ATXN7 | Body;Body | S_Shore | -1.21E-02 | 8.41E-10 |
| cg13321077 | RESP18 | Body | Island | 1.25E-02 | 1.45E-05 |
| cg13321967 | NA | NA | S_Shore | 1.89E-02 | 3.23E-08 |
| cg13327545 | NA | NA | Island | 1.19E-02 | 1.44E-08 |
| cg13337047 | NA | NA | Island | 2.00E-02 | 2.04E-05 |
| cg13339930 | NA | NA | S_Shelf | -7.86E-03 | 3.60E-06 |
| cg13347071 | UNC80;UNC80;UNC | 1stExon;5'UTR;1stE | Island | 7.03E-03 | 3.42E-07 |
| cg13351161 | SCARA3;SCARA3 | TSS1500;TSS1500 | N_Shore | -5.71E-03 | 1.64E-05 |
| cg13368756 | CTNND2 | TSS200 | Island | 3.25E-02 | 2.51E-05 |
| cg13370427 | ADSL;ADSL | Body;Body | S_Shore | -1.48E-02 | 3.39E-16 |
| cg13378934 | MEIS3;MEIS3 | Body;Body | S_Shore | 1.66E-02 | 3.82E-12 |
| cg13381486 | FAM123C;FAM123C | TSS1500;5'UTR;5'U | Island | 9.60E-03 | 6.52E-08 |

| | | | | | |
|---|---|---|---|---|---|
| cg13386070 | TNN | Body | N_Shore | -6.82E-03 | 1.97E-05 |
| cg13388731 | DNAJB2;DNAJB2 | TSS1500;TSS1500 | N_Shore | -1.55E-02 | 1.23E-17 |
| cg13391675 | CTNNA2;CTNNA2 | Body;Body | NA | -2.44E-02 | 3.46E-13 |
| cg13392029 | ARTN;ARTN;ARTN;A | TSS1500;5'UTR;5'U | N_Shore | -6.03E-03 | 5.51E-06 |
| cg13392141 | AK5;AK5;AK5 | Body;5'UTR;1stExon | S_Shore | 7.00E-03 | 3.86E-05 |
| cg13393785 | LASS4 | Body | NA | -1.27E-02 | 1.12E-15 |
| cg13405782 | DEFB124 | TSS200 | N_Shelf | -9.56E-03 | 2.24E-08 |
| cg13406339 | NA | NA | NA | -6.87E-03 | 5.07E-06 |
| cg13408430 | SV2C | 5'UTR | Island | 7.89E-03 | 6.24E-09 |
| cg13422921 | NA | NA | S_Shore | -1.88E-02 | 8.55E-08 |
| cg13425146 | NA | NA | NA | -1.27E-02 | 4.19E-08 |
| cg13425391 | NA | NA | N_Shore | 7.91E-03 | 4.76E-06 |
| cg13430755 | NA | NA | NA | -1.25E-02 | 3.27E-14 |
| cg13434842 | GATA4 | Body | S_Shore | 1.54E-02 | 1.46E-09 |
| cg13435718 | NA | NA | S_Shore | 1.08E-02 | 4.06E-08 |
| cg13436155 | NA | NA | S_Shore | 1.25E-02 | 3.57E-06 |
| cg13449257 | NA | NA | N_Shore | -1.48E-02 | 9.99E-08 |
| cg13449778 | FAM163A;FAM163A | 1stExon;5'UTR | Island | 1.42E-02 | 6.63E-05 |
| cg13451651 | LOC100129066 | Body | NA | -5.49E-03 | 6.89E-05 |
| cg13455326 | OLFM2 | Body | N_Shelf | -1.23E-02 | 1.16E-11 |
| cg13458211 | B4GALT2 | TSS1500 | N_Shore | -5.77E-03 | 3.66E-09 |
| cg13460168 | NA | NA | NA | -7.30E-03 | 1.06E-06 |
| cg13463639 | SIGIRR;SIGIRR | TSS1500;TSS1500 | S_Shore | -6.48E-03 | 1.82E-05 |
| cg13480898 | C19orf66 | TSS1500 | N_Shore | -1.19E-02 | 3.63E-08 |
| cg13481132 | KCNT1 | Body | Island | 1.70E-02 | 6.88E-06 |
| cg13487312 | TNKS1BP1 | 5'UTR | N_Shore | -1.15E-02 | 6.06E-10 |
| cg13490971 | NDFIP1 | TSS1500 | N_Shore | -9.40E-03 | 3.92E-05 |
| cg13495381 | NA | NA | NA | -1.71E-02 | 1.28E-11 |
| cg13501527 | PHF19;PHF19 | TSS1500;TSS1500 | S_Shore | -8.48E-03 | 5.44E-08 |
| cg13517124 | GRIP2 | Body | NA | -6.53E-03 | 3.76E-05 |
| cg13539545 | NA | NA | Island | 2.12E-02 | 5.63E-07 |
| cg13540171 | NA | NA | Island | 9.83E-03 | 4.18E-05 |
| cg13544075 | LCN6 | TSS1500 | S_Shelf | -1.08E-02 | 5.13E-05 |
| cg13561879 | UNC5D | TSS1500 | Island | 1.03E-02 | 6.31E-07 |
| cg13563634 | TAP2;TAP2 | Body;Body | N_Shore | -1.30E-02 | 2.70E-13 |
| cg13563725 | NA | NA | Island | 1.00E-02 | 1.44E-06 |
| cg13567299 | GDPD5 | 5'UTR | NA | -1.27E-02 | 6.01E-14 |
| cg13573745 | NA | NA | S_Shelf | -6.85E-03 | 1.36E-05 |
| cg13575298 | EIF5A2 | TSS1500 | Island | 7.12E-03 | 9.56E-06 |
| cg13575499 | MIPOL1 | 5'UTR | S_Shore | -1.88E-02 | 2.27E-08 |
| cg13578465 | INF2;INF2;INF2 | 5'UTR;5'UTR;5'UTR | Island | -1.32E-02 | 1.11E-08 |
| cg13586457 | SPTBN5 | TSS1500 | NA | -9.25E-03 | 1.30E-10 |
| cg13591783 | ANXA1 | 5'UTR | NA | -1.13E-02 | 2.41E-05 |
| cg13605327 | NA | NA | NA | -1.16E-02 | 2.12E-18 |
| cg13625026 | NA | NA | NA | -1.02E-02 | 2.63E-05 |
| cg13630131 | NA | NA | N_Shore | -1.25E-02 | 2.37E-10 |
| cg13630871 | ENDOG | Body | S_Shore | -4.34E-03 | 2.24E-07 |
| cg13640414 | AKAP8L | TSS1500 | S_Shore | -2.28E-02 | 1.34E-22 |
| cg13649056 | NA | NA | Island | 1.14E-02 | 2.79E-15 |
| cg13654588 | PRLHR | Body | Island | 1.09E-02 | 7.10E-11 |

| | | | | | |
|---|---|---|---|---|---|
| cg13657200 | DFNB31;DFNB31 | Body;TSS1500 | N_Shore | -1.09E-02 | 1.57E-15 |
| cg13665684 | FAM188B | Body | NA | -1.43E-02 | 3.14E-14 |
| cg13667638 | NA | NA | N_Shore | 1.08E-02 | 9.07E-09 |
| cg13669432 | NA | NA | NA | -1.83E-02 | 6.24E-24 |
| cg13673164 | SYPL2 | 1stExon | Island | 2.57E-02 | 1.55E-05 |
| cg13678973 | ELAVL4 | Body | Island | 2.43E-02 | 8.28E-09 |
| cg13679679 | CMC1 | TSS1500 | N_Shore | -7.97E-03 | 5.41E-05 |
| cg13679804 | NA | NA | NA | -1.03E-02 | 9.29E-06 |
| cg13683194 | C9orf125 | 3'UTR | NA | -1.10E-02 | 2.96E-10 |
| cg13683374 | GPR142 | Body | N_Shelf | -1.10E-02 | 2.18E-12 |
| cg13688769 | PDZRN3 | TSS1500 | Island | 9.48E-03 | 1.76E-07 |
| cg13697715 | LOC202181 | TSS1500 | S_Shore | -1.02E-02 | 4.33E-15 |
| cg13698883 | IGFBPL1 | Body | N_Shore | -1.23E-02 | 7.39E-12 |
| cg13700939 | NA | NA | Island | -6.62E-03 | 3.65E-06 |
| cg13702996 | IQSEC3 | 1stExon | Island | 9.42E-03 | 2.87E-07 |
| cg13708910 | ATPGD1;ATPGD1 | 5'UTR;5'UTR | S_Shore | -4.22E-03 | 4.11E-05 |
| cg13714026 | CDH23;CDH23 | Body;Body | NA | -2.13E-02 | 2.08E-10 |
| cg13719901 | LRRC2;LRRC2 | 5'UTR;TSS200 | S_Shore | -1.58E-02 | 9.95E-09 |
| cg13720022 | CENPP;ECM2 | Body;Body | NA | -1.31E-02 | 3.66E-09 |
| cg13725599 | OXT | TSS200 | Island | -1.99E-02 | 1.54E-07 |
| cg13731106 | NA | NA | Island | 7.51E-03 | 3.37E-07 |
| cg13731636 | NFATC1;NFATC1;NF | Body;Body;Body;Bo | N_Shore | -1.10E-02 | 8.79E-09 |
| cg13734401 | NA | NA | N_Shore | -1.34E-02 | 3.15E-07 |
| cg13740815 | SHANK1 | Body | Island | 1.20E-02 | 2.00E-08 |
| cg13745832 | FAM18B2 | 3'UTR | NA | -1.98E-02 | 4.48E-22 |
| cg13755776 | NA | NA | S_Shore | -1.13E-02 | 3.33E-07 |
| cg13773356 | NA | NA | S_Shelf | -1.44E-02 | 9.46E-08 |
| cg13782884 | FAM46B;FAM46B | 5'UTR;1stExon | Island | 1.78E-02 | 1.22E-05 |
| cg13786089 | ZFR2;ZFR2;ZFR2 | TSS200;TSS200;TSS | Island | 1.74E-02 | 3.09E-11 |
| cg13790576 | LCN6 | Body | Island | -1.07E-02 | 9.72E-07 |
| cg13791254 | FOXE1 | 1stExon | Island | 1.91E-02 | 8.80E-05 |
| cg13794404 | NA | NA | NA | -1.05E-02 | 1.23E-05 |
| cg13798384 | PCDH9;PCDH9 | Body;Body | NA | -2.88E-02 | 7.48E-08 |
| cg13800496 | UTRN | Body | NA | -1.58E-02 | 1.93E-11 |
| cg13806070 | BMP8A;BMP8A | 1stExon;5'UTR | Island | 1.74E-02 | 9.23E-09 |
| cg13806741 | FAM181B;FAM181B | 1stExon;3'UTR | Island | 8.87E-03 | 6.26E-08 |
| cg13816999 | PARVA | TSS1500 | N_Shore | 7.62E-03 | 6.63E-05 |
| cg13823169 | NA | NA | N_Shelf | -1.13E-02 | 7.49E-17 |
| cg13826452 | NA | NA | NA | 1.23E-02 | 2.03E-11 |
| cg13827984 | NA | NA | Island | 6.97E-03 | 1.42E-06 |
| cg13828047 | MPI | TSS1500 | N_Shore | -6.54E-03 | 1.17E-08 |
| cg13829089 | TNFSF13;TNFSF13;T | TSS1500;TSS1500;3 | N_Shelf | -1.52E-02 | 1.81E-06 |
| cg13834623 | SCGN | TSS200 | Island | 1.88E-02 | 3.03E-10 |
| cg13835114 | ASS1;ASS1 | 5'UTR;5'UTR | NA | -1.08E-02 | 8.18E-10 |
| cg13839957 | CHD5 | 3'UTR | NA | -1.19E-02 | 4.15E-10 |
| cg13842240 | NA | NA | S_Shore | 5.97E-03 | 6.26E-05 |
| cg13848598 | ADRB1 | 1stExon | Island | 1.02E-02 | 9.08E-11 |
| cg13851843 | ABCC8;ABCC8 | 5'UTR;1stExon | Island | 9.56E-03 | 1.88E-05 |
| cg13854219 | NA | NA | NA | -1.30E-02 | 4.74E-10 |
| cg13855568 | NA | NA | Island | 7.04E-03 | 4.26E-06 |

| | | | | | |
|---|---|---|---|---|---|
| cg13856810 | SAMD11 | Body | S_Shore | -1.82E-02 | 1.02E-15 |
| cg13856867 | KRT78 | TSS200 | NA | -7.74E-03 | 3.48E-06 |
| cg13876650 | FAM54B;FAM54B;FA | TSS1500;TSS1500;T | N_Shore | -4.01E-03 | 1.22E-05 |
| cg13882284 | MSX1 | TSS1500 | Island | 6.82E-03 | 4.10E-07 |
| cg13898106 | TRIM45;TRIM45 | TSS1500;TSS1500 | Island | 1.42E-02 | 5.59E-07 |
| cg13899108 | PDE4C | 5'UTR | S_Shore | 1.07E-02 | 1.78E-06 |
| cg13900817 | NEURL1B | Body | S_Shore | -1.21E-02 | 4.96E-10 |
| cg13902210 | KCNC4;KCNC4;KCNC | 1stExon;1stExon;1s | Island | 1.95E-02 | 1.95E-08 |
| cg13905757 | FAM171A1 | Body | N_Shore | 7.36E-03 | 3.51E-05 |
| cg13912573 | LOC100129066 | Body | S_Shore | -1.03E-02 | 1.91E-08 |
| cg13915538 | ZNF676 | Body | NA | -8.45E-03 | 1.53E-05 |
| cg13927769 | COL15A1 | Body | NA | -1.29E-02 | 2.62E-07 |
| cg13929658 | NA | NA | NA | -9.67E-03 | 4.09E-08 |
| cg13933080 | SPAG6;SPAG6 | TSS1500;TSS1500 | Island | 1.36E-02 | 3.33E-09 |
| cg13944685 | IL18BP;IL18BP;IL18E | TSS200;5'UTR;TSS1! | NA | -1.04E-02 | 7.95E-12 |
| cg13948857 | C5orf56 | Body | NA | -7.36E-03 | 1.91E-06 |
| cg13949132 | NA | NA | NA | 4.25E-03 | 3.36E-05 |
| cg13954457 | FBLL1 | Body | Island | 2.40E-02 | 2.24E-11 |
| cg13957558 | TSPAN16;RAB3D | Body;3'UTR | NA | 1.60E-02 | 4.43E-07 |
| cg13959344 | NA | NA | NA | -1.41E-02 | 4.91E-14 |
| cg13962212 | NA | NA | NA | 1.74E-02 | 9.59E-07 |
| cg13964105 | LRRC3B | TSS1500 | N_Shore | 1.39E-02 | 2.10E-08 |
| cg13973820 | HMGCLL1;HMGCLL1 | TSS200;TSS200 | Island | 1.96E-02 | 4.02E-10 |
| cg13984563 | SERPINA5 | TSS200 | NA | 8.33E-03 | 6.94E-06 |
| cg13999458 | NA | NA | S_Shore | 1.70E-02 | 1.74E-18 |
| cg14001664 | NA | NA | NA | 2.37E-02 | 5.01E-10 |
| cg14003035 | KIAA1755 | TSS200 | Island | 2.05E-02 | 2.33E-07 |
| cg14007067 | SALL3 | TSS200 | Island | 1.75E-02 | 1.72E-08 |
| cg14009098 | SLC35F3 | Body | Island | 1.28E-02 | 2.05E-08 |
| cg14009632 | TJAP1;TJAP1;TJAP1;T | TSS1500;TSS1500;T | NA | -1.11E-02 | 6.08E-05 |
| cg14010720 | PTK6 | TSS200 | S_Shore | -1.02E-02 | 1.52E-07 |
| cg14018141 | CD300A | 3'UTR | NA | -8.74E-03 | 1.19E-08 |
| cg14020846 | NA | NA | Island | 8.50E-03 | 1.65E-05 |
| cg14021478 | CUTA;CUTA;CUTA;C | Body;Body;Body;Bo | N_Shore | -7.82E-03 | 9.41E-09 |
| cg14024579 | INPP5E | Body | Island | -8.79E-03 | 1.38E-08 |
| cg14030904 | ZC3H12D | TSS1500 | NA | -7.53E-03 | 3.18E-05 |
| cg14034476 | C19orf28;C19orf28; | Body;Body;Body | N_Shore | -1.04E-02 | 1.37E-10 |
| cg14034778 | NA | NA | NA | -6.50E-03 | 2.04E-05 |
| cg14035828 | NA | NA | NA | -1.31E-02 | 7.69E-08 |
| cg14039301 | NA | NA | N_Shelf | -6.68E-03 | 6.15E-08 |
| cg14068328 | TBX18 | TSS200 | Island | 1.01E-02 | 1.14E-07 |
| cg14072219 | MAP1LC3C | TSS1500 | NA | -1.19E-02 | 3.12E-10 |
| cg14074174 | SNAPC2;SNAPC2 | Body;Body | S_Shore | -6.44E-03 | 5.77E-10 |
| cg14089045 | CCDC88C | Body | NA | -1.10E-02 | 1.96E-05 |
| cg14093936 | SEMA7A;SEMA7A;SI | TSS1500;TSS1500;T | S_Shore | -6.78E-03 | 3.23E-11 |
| cg14095048 | NA | NA | NA | -1.04E-02 | 2.28E-14 |
| cg14097568 | NA | NA | NA | -7.43E-03 | 3.47E-05 |
| cg14098299 | NA | NA | N_Shore | -8.45E-03 | 3.59E-05 |
| cg14102128 | SEPT10;SEPT10;SEPT | 5'UTR;1stExon;1stE | Island | 2.28E-02 | 2.19E-05 |
| cg14112601 | GCNT2 | TSS1500 | NA | -7.23E-03 | 1.01E-06 |

| | | | | | |
|---|---|---|---|---|---|
| cg14121185 | PRKCA | Body | NA | -1.11E-02 | 2.68E-08 |
| cg14123992 | APOE | TSS1500 | N_Shelf | -9.35E-03 | 1.00E-07 |
| cg14129473 | DAP | Body | NA | -1.59E-02 | 1.17E-16 |
| cg14131038 | NCK2;NCK2 | TSS1500;TSS1500 | N_Shore | -6.97E-03 | 3.14E-05 |
| cg14132888 | TRIM36;TRIM36;TRI | Body;Body;Body | Island | 1.94E-02 | 1.17E-07 |
| cg14134497 | DTNA;DTNA;DTNA | 5'UTR;5'UTR;5'UTR | Island | 8.61E-03 | 2.38E-05 |
| cg14134616 | NA | NA | Island | -1.44E-02 | 9.41E-06 |
| cg14149172 | PI4KB | TSS200 | N_Shore | -1.36E-02 | 4.37E-11 |
| cg14155831 | NA | NA | NA | -1.16E-02 | 1.31E-10 |
| cg14160151 | MSTO1 | Body | S_Shelf | -8.88E-03 | 5.71E-12 |
| cg14176752 | NA | NA | Island | 1.06E-02 | 1.47E-05 |
| cg14187687 | SAMD4A;SAMD4A;S | Body;Body;Body | NA | -7.98E-03 | 9.53E-05 |
| cg14188401 | NA | NA | S_Shelf | -1.54E-02 | 8.84E-18 |
| cg14200127 | DPYD | Body | NA | -1.94E-02 | 3.88E-12 |
| cg14204433 | PER3;PER3 | 5'UTR;1stExon | Island | 1.26E-02 | 1.48E-05 |
| cg14207114 | NA | NA | N_Shore | -8.23E-03 | 1.79E-05 |
| cg14207210 | PPM1E | TSS200 | Island | 2.66E-02 | 6.02E-05 |
| cg14208102 | TREX1;TREX1 | TSS200;TSS200 | NA | -5.98E-03 | 1.68E-06 |
| cg14209784 | AGAP11;C10orf116 | TSS1500;Body | N_Shore | -9.71E-03 | 2.62E-06 |
| cg14214353 | MFSD2B | Body | S_Shelf | -6.54E-03 | 2.04E-05 |
| cg14217861 | NOTCH1 | Body | Island | -2.55E-02 | 8.86E-06 |
| cg14218480 | NA | NA | N_Shore | -7.86E-03 | 6.55E-07 |
| cg14222574 | NA | NA | NA | -7.33E-03 | 6.28E-05 |
| cg14231297 | ZSCAN18;ZSCAN18 | TSS200;TSS200 | Island | 1.56E-02 | 8.71E-07 |
| cg14237025 | CCL16 | TSS1500 | NA | -9.19E-03 | 6.72E-07 |
| cg14237550 | MST1P2 | Body | Island | 4.96E-03 | 1.62E-07 |
| cg14242557 | ZNF483;ZNF483 | 5'UTR;5'UTR | S_Shore | -1.70E-02 | 1.81E-05 |
| cg14244577 | DDX19B;DDX19B;DI | TSS200;TSS200;TSS: | NA | -4.95E-03 | 1.94E-05 |
| cg14249348 | ZAR1 | TSS200 | Island | 2.09E-02 | 1.84E-05 |
| cg14254534 | SPRED3;SPRED3 | Body;Body | N_Shelf | -1.60E-02 | 1.16E-16 |
| cg14254767 | TJP2;TJP2;TJP2;TJP2 | TSS200;Body;5'UTR | NA | -1.28E-02 | 3.05E-11 |
| cg14255824 | TJP2;TJP2;TJP2;TJP2 | Body;5'UTR;Body;B | NA | -1.62E-02 | 8.13E-05 |
| cg14257429 | NA | NA | NA | -1.18E-02 | 6.28E-14 |
| cg14264496 | LOC100133612 | Body | N_Shore | -1.19E-02 | 3.38E-06 |
| cg14267222 | NA | NA | N_Shelf | -8.98E-03 | 6.76E-07 |
| cg14268223 | DNAJA1 | Body | S_Shore | -1.25E-02 | 1.69E-07 |
| cg14270193 | ASGR1 | Body | S_Shore | -5.21E-03 | 1.04E-07 |
| cg14286292 | NA | NA | N_Shore | -7.50E-03 | 3.36E-09 |
| cg14290170 | NA | NA | S_Shore | -1.49E-02 | 1.39E-12 |
| cg14292522 | UHRF1BP1L;UHRF1E | Body;Body | N_Shore | -2.24E-02 | 5.64E-06 |
| cg14296767 | HLA-L | TSS1500 | N_Shore | -1.40E-02 | 5.62E-06 |
| cg14305711 | COMMD1 | TSS1500 | NA | -2.11E-02 | 2.48E-14 |
| cg14319235 | ELTD1 | Body | Island | 1.62E-02 | 6.88E-14 |
| cg14321284 | NA | NA | NA | -6.29E-03 | 2.19E-05 |
| cg14326805 | TJP2 | 5'UTR | S_Shore | -1.24E-02 | 9.31E-09 |
| cg14330675 | DKFZp434J0226 | TSS200 | NA | -1.37E-02 | 1.90E-07 |
| cg14333542 | NA | NA | NA | -9.40E-03 | 2.17E-09 |
| cg14334310 | NA | NA | S_Shore | -7.92E-03 | 7.15E-07 |
| cg14336011 | LHX3 | Body | Island | 1.17E-02 | 3.02E-08 |
| cg14337844 | NA | NA | Island | 1.38E-02 | 2.91E-06 |

| | | | | | |
|---|---|---|---|---|---|
| cg14341254 | NA | NA | NA | -7.31E-03 | 8.83E-06 |
| cg14345012 | MIR663 | TSS1500 | Island | 1.70E-02 | 6.39E-11 |
| cg14349763 | ADAMTS13;ADAMTS | Body;TSS200;TSS20 | S_Shelf | -7.47E-03 | 3.57E-09 |
| cg14353137 | NA | NA | Island | 3.24E-02 | 9.44E-10 |
| cg14354820 | ANK1 | Body | NA | -6.08E-03 | 8.50E-06 |
| cg14356799 | HLA-DPB1 | Body | NA | -1.82E-02 | 1.22E-11 |
| cg14359680 | GPR132 | TSS1500 | NA | -1.01E-02 | 9.09E-14 |
| cg14361627 | KLF14 | TSS1500 | Island | 1.94E-02 | 1.96E-15 |
| cg14373988 | PEX10;PEX10 | TSS1500;TSS1500 | N_Shore | -2.01E-02 | 9.79E-09 |
| cg14378924 | SLC44A4 | Body | S_Shore | -8.44E-03 | 6.71E-09 |
| cg14385298 | NA | NA | NA | -9.54E-03 | 3.00E-05 |
| cg14391923 | NA | NA | NA | -2.74E-02 | 5.66E-05 |
| cg14392725 | NA | NA | NA | -1.73E-02 | 4.12E-08 |
| cg14394939 | BTG3;BTG3 | 5'UTR;5'UTR | N_Shore | -1.93E-02 | 1.05E-25 |
| cg14398464 | NOL12 | TSS1500 | N_Shore | -6.99E-03 | 1.17E-09 |
| cg14410016 | HOXD9 | TSS1500 | Island | 7.66E-03 | 7.39E-06 |
| cg14412322 | MAGI1;MAGI1;MAG | Body;Body;Body | Island | 7.99E-03 | 4.38E-05 |
| cg14414100 | SLC24A2 | Body | NA | -1.45E-02 | 1.46E-11 |
| cg14414464 | NA | NA | Island | 1.13E-02 | 3.41E-06 |
| cg14416311 | GAS1 | 1stExon | Island | 1.64E-02 | 8.54E-05 |
| cg14420000 | NA | NA | NA | -9.58E-03 | 8.36E-10 |
| cg14425564 | LHX2 | Body | NA | 1.36E-02 | 1.03E-06 |
| cg14431361 | NA | NA | NA | 9.21E-03 | 6.25E-07 |
| cg14438453 | DAAM2 | 5'UTR | NA | -5.54E-03 | 1.20E-05 |
| cg14441491 | WBSCR28 | Body | NA | -6.74E-03 | 1.29E-05 |
| cg14442421 | SLC24A2 | TSS1500 | Island | 1.12E-02 | 1.94E-05 |
| cg14442492 | ELL2 | Body | N_Shore | -1.17E-02 | 6.63E-09 |
| cg14472366 | NA | NA | Island | 1.97E-02 | 2.32E-08 |
| cg14480266 | NA | NA | NA | -1.44E-02 | 3.78E-09 |
| cg14481315 | NA | NA | N_Shelf | -6.51E-03 | 6.63E-09 |
| cg14486857 | AP2A2 | Body | Island | -1.17E-02 | 1.46E-11 |
| cg14489366 | PRUNE2 | TSS200 | Island | 1.11E-02 | 9.15E-05 |
| cg14505439 | RAPGEF1 | Body | NA | -7.34E-03 | 1.34E-10 |
| cg14507845 | NA | NA | NA | -7.64E-03 | 1.31E-06 |
| cg14507891 | KLHDC8B | TSS1500 | N_Shore | -7.04E-03 | 4.77E-05 |
| cg14510359 | MMEL1 | Body | Island | 1.73E-02 | 1.86E-08 |
| cg14511946 | NA | NA | Island | 2.56E-02 | 4.44E-12 |
| cg14514813 | PCDHA6;PCDHA2;PC | Body;Body;Body;Bo | N_Shore | 1.44E-02 | 2.20E-07 |
| cg14518803 | NA | NA | N_Shelf | -1.00E-02 | 4.64E-13 |
| cg14519115 | PLCXD3;PLCXD3 | 1stExon;5'UTR | Island | 9.26E-03 | 1.12E-07 |
| cg14519664 | MLLT3 | Body | NA | -1.06E-02 | 3.19E-05 |
| cg14526718 | NA | NA | N_Shore | 1.63E-02 | 2.09E-05 |
| cg14527110 | P4HA2;P4HA2;P4HA | 5'UTR;5'UTR;5'UTR; | N_Shore | 3.92E-03 | 9.35E-05 |
| cg14532894 | CIZ1;DNM1;DNM1;C | 5'UTR;TSS1500;TSS1 | N_Shore | -7.81E-03 | 1.65E-09 |
| cg14535884 | ATP6V1G1 | TSS1500 | N_Shore | -1.22E-02 | 2.50E-05 |
| cg14536184 | NA | NA | NA | -1.39E-02 | 2.50E-07 |
| cg14543780 | AFAP1;AFAP1;LOC84 | Body;Body;Body | NA | -7.98E-03 | 1.93E-06 |
| cg14548264 | NUP214 | 3'UTR | NA | -7.63E-03 | 2.29E-05 |
| cg14555996 | NA | NA | NA | -1.28E-02 | 3.83E-08 |
| cg14556683 | EPHX3;EPHX3 | 1stExon;Body | Island | 1.16E-02 | 1.04E-08 |

| | | | | | |
|---|---|---|---|---|---|
| cg14568105 | NA | NA | S_Shore | 1.10E-02 | 9.03E-06 |
| cg14570291 | NA | NA | Island | 1.17E-02 | 4.27E-07 |
| cg14571710 | KIAA1949;NRM;KIA/ | TSS1500;3'UTR;TSS: | S_Shore | -5.00E-03 | 1.63E-06 |
| cg14573448 | HOXA3 | 5'UTR | S_Shore | -1.16E-02 | 7.81E-11 |
| cg14577707 | NA | NA | NA | -1.62E-02 | 9.55E-15 |
| cg14579240 | EXD3 | Body | NA | -5.93E-03 | 2.80E-05 |
| cg14582763 | ZNF578 | TSS200 | Island | 1.13E-02 | 5.30E-06 |
| cg14585371 | NA | NA | Island | 8.30E-03 | 9.65E-09 |
| cg14600885 | MYOZ3;MYOZ3 | TSS200;TSS200 | S_Shelf | -1.17E-02 | 7.35E-15 |
| cg14601038 | TPO;TPO;TPO;TPO | Body;Body;Body;Bo | Island | 1.89E-02 | 1.65E-05 |
| cg14601444 | DCTN1;DCTN1 | TSS200;TSS200 | NA | -1.36E-02 | 7.02E-14 |
| cg14602087 | ZNF703 | 3'UTR | N_Shore | -1.42E-02 | 9.13E-07 |
| cg14603098 | ACCN1 | TSS1500 | Island | 3.66E-02 | 6.44E-11 |
| cg14605590 | LOC100128076 | Body | N_Shelf | -1.11E-02 | 2.71E-06 |
| cg14609289 | ENGASE | 3'UTR | NA | -5.34E-03 | 2.51E-07 |
| cg14614094 | EXOSC2 | TSS1500 | N_Shore | 1.60E-02 | 9.91E-11 |
| cg14619249 | NA | NA | NA | -7.79E-03 | 5.69E-06 |
| cg14620572 | GPR144 | Body | Island | 1.13E-02 | 1.10E-07 |
| cg14624546 | ST5;ST5;ST5 | 5'UTR;5'UTR;5'UTR | NA | -7.52E-03 | 5.52E-07 |
| cg14631386 | NA | NA | Island | 1.19E-02 | 3.98E-07 |
| cg14632899 | CHRM1 | 5'UTR | NA | -6.05E-03 | 6.87E-05 |
| cg14650167 | FSTL5;FSTL5;FSTL5 | TSS200;TSS200;TSS: | NA | 6.75E-03 | 7.81E-05 |
| cg14654886 | EVC2;EVC2 | 5'UTR;TSS200 | Island | 2.55E-02 | 5.00E-05 |
| cg14670435 | MIR1258;ZNF385B | TSS1500;TSS1500 | Island | 8.34E-03 | 8.75E-06 |
| cg14671809 | ERC2 | 3'UTR | NA | -1.24E-02 | 3.26E-16 |
| cg14674720 | NA | NA | Island | 1.16E-02 | 8.86E-14 |
| cg14685796 | NA | NA | Island | 1.51E-02 | 2.07E-08 |
| cg14687785 | NA | NA | NA | -1.55E-02 | 6.11E-05 |
| cg14689532 | PNPLA2 | Body | S_Shore | -1.18E-02 | 5.19E-14 |
| cg14692377 | SLC6A4;SLC6A4 | 1stExon;5'UTR | Island | 1.60E-02 | 1.20E-11 |
| cg14694075 | HNF1B;HNF1B | Body;Body | NA | -1.05E-02 | 8.06E-13 |
| cg14698448 | NA | NA | N_Shore | 2.51E-02 | 2.96E-13 |
| cg14715327 | OR4A16 | TSS1500 | NA | -1.98E-02 | 2.19E-08 |
| cg14719352 | FGF21;FUT1 | TSS1500;5'UTR | S_Shore | -9.50E-03 | 1.01E-11 |
| cg14722140 | C6orf141 | 1stExon | Island | 1.83E-02 | 1.52E-05 |
| cg14730524 | FAT3 | Body | S_Shore | -9.25E-03 | 2.98E-13 |
| cg14734340 | YPEL4 | 3'UTR | N_Shore | -7.91E-03 | 7.05E-06 |
| cg14747813 | NA | NA | NA | -2.07E-02 | 8.11E-27 |
| cg14754427 | DERA | Body | NA | -2.11E-02 | 2.23E-15 |
| cg14772925 | PGR | 1stExon | Island | 8.47E-03 | 5.83E-07 |
| cg14775286 | DZIP1L;DZIP1L | 5'UTR;TSS1500 | N_Shore | -1.37E-02 | 1.79E-10 |
| cg14776518 | FBXL22 | Body | N_Shore | -1.31E-02 | 4.55E-08 |
| cg14781189 | KCNH7;KCNH7;KCNI | 1stExon;5'UTR;1stE: | NA | 2.79E-02 | 5.43E-13 |
| cg14789121 | FBLN2;FBLN2;FBLN2 | Body;Body;Body | N_Shelf | -7.99E-03 | 2.50E-05 |
| cg14789818 | NA | NA | Island | 2.38E-02 | 4.33E-08 |
| cg14793137 | TPO;TPO;TPO;TPO | Body;Body;Body;Bo | Island | 2.51E-02 | 1.27E-05 |
| cg14794655 | C2orf73 | Body | S_Shore | -1.10E-02 | 1.08E-10 |
| cg14818621 | NA | NA | NA | -9.43E-03 | 1.46E-07 |
| cg14826456 | ADRB1 | 1stExon | Island | 9.40E-03 | 6.04E-07 |
| cg14837598 | EPB49;EPB49;EPB49 | TSS200;5'UTR;5'UTI | S_Shelf | -1.32E-02 | 1.61E-14 |

| | | | | | |
|---|---|---|---|---|---|
| cg14843632 | ANKH | Body | N_Shore | -2.39E-02 | 5.17E-07 |
| cg14844989 | NA | NA | NA | -1.79E-02 | 3.15E-05 |
| cg14855519 | EBF2 | Body | N_Shore | 1.42E-02 | 1.07E-05 |
| cg14857193 | ALDH1L1 | Body | NA | -7.67E-03 | 3.00E-09 |
| cg14862806 | NA | NA | Island | 9.46E-03 | 2.75E-11 |
| cg14866863 | NA | NA | Island | 1.17E-02 | 2.88E-06 |
| cg14871932 | GCK;GCK;GCK | Body;Body;Body | Island | 2.13E-02 | 7.19E-07 |
| cg14874742 | NA | NA | NA | -2.24E-02 | 3.48E-17 |
| cg14879279 | NA | NA | NA | -1.58E-02 | 1.40E-11 |
| cg14893576 | NA | NA | N_Shelf | -1.33E-02 | 7.42E-08 |
| cg14894848 | FAM19A5 | TSS1500 | Island | 1.30E-02 | 3.51E-06 |
| cg14898223 | NA | NA | S_Shore | -1.62E-02 | 4.63E-10 |
| cg14901671 | NA | NA | Island | 4.76E-03 | 8.95E-06 |
| cg14909325 | HCCA2 | Body | S_Shore | -6.86E-03 | 3.47E-06 |
| cg14909730 | TFAP2A;TFAP2A | Body;TSS200 | N_Shore | 1.69E-02 | 2.32E-07 |
| cg14911856 | NA | NA | S_Shore | -7.91E-03 | 6.97E-06 |
| cg14924160 | SUGT1L1 | TSS1500 | N_Shore | -1.47E-02 | 2.31E-08 |
| cg14932016 | ACTN2 | TSS200 | Island | 7.83E-03 | 5.11E-05 |
| cg14934413 | NA | NA | N_Shore | 1.02E-02 | 3.41E-08 |
| cg14950321 | PLIN5 | Body | N_Shore | -1.31E-02 | 2.56E-14 |
| cg14951193 | PLIN5 | TSS200 | Island | 1.56E-02 | 6.48E-06 |
| cg14956327 | DDO;DDO | TSS1500;TSS1500 | NA | -8.51E-03 | 1.41E-06 |
| cg14958635 | NEUROG1 | 1stExon | Island | 1.01E-02 | 4.42E-06 |
| cg14959729 | T | Body | Island | 8.01E-03 | 2.20E-07 |
| cg14962007 | NA | NA | NA | -9.28E-03 | 3.69E-05 |
| cg14972576 | NA | NA | NA | -1.62E-02 | 1.48E-06 |
| cg14973055 | DNAI2 | Body | NA | -7.25E-03 | 2.93E-07 |
| cg14975410 | NA | NA | S_Shore | -9.19E-03 | 6.92E-05 |
| cg14977938 | ZFYVE21 | Body | N_Shelf | -1.25E-02 | 5.65E-13 |
| cg14979593 | PGAP2;PGAP2;PGAF | Body;Body;5'UTR;B | S_Shore | -8.01E-03 | 1.31E-11 |
| cg14994947 | NA | NA | NA | -1.48E-02 | 2.31E-13 |
| cg14995235 | DOCK1 | Body | NA | -6.27E-03 | 3.10E-06 |
| cg14999189 | RAP1GAP2;RAP1GA | Body;Body | NA | -8.11E-03 | 2.76E-13 |
| cg15000071 | EPHB1 | Body | NA | -8.05E-03 | 1.48E-11 |
| cg15001056 | MED12L | Body | NA | -1.48E-02 | 4.59E-14 |
| cg15001747 | NA | NA | Island | 7.20E-03 | 5.38E-06 |
| cg15007156 | KIAA1026;KIAA1026 | TSS200;TSS200 | Island | 9.43E-03 | 4.78E-09 |
| cg15012161 | NA | NA | NA | -1.09E-02 | 5.29E-06 |
| cg15020894 | SLC22A3 | Body | N_Shelf | -9.67E-03 | 3.37E-05 |
| cg15027907 | NA | NA | Island | 2.57E-02 | 1.29E-10 |
| cg15037004 | ZNF366 | TSS1500 | NA | -1.36E-02 | 3.42E-09 |
| cg15055782 | BARX2 | Body | S_Shelf | -8.25E-03 | 9.46E-07 |
| cg15058464 | C20orf56 | TSS1500 | Island | 2.05E-02 | 1.09E-08 |
| cg15060599 | NA | NA | Island | 1.79E-02 | 1.83E-06 |
| cg15063366 | NA | NA | NA | -1.21E-02 | 4.45E-06 |
| cg15069295 | NA | NA | Island | 9.89E-03 | 2.45E-09 |
| cg15070718 | NR2F2;NR2F2;NR2F2 | TSS200;Body;Body; | Island | 2.15E-02 | 4.14E-05 |
| cg15084543 | ELTD1;ELTD1 | 5'UTR;1stExon | Island | 2.65E-02 | 8.22E-16 |
| cg15084803 | CHCHD4;CHCHD4 | Body;Body | NA | -1.30E-02 | 3.22E-07 |
| cg15090440 | PRDM16;PRDM16 | Body;Body | N_Shelf | -9.42E-03 | 5.81E-06 |

| | | | | | |
|---|---|---|---|---|---|
| cg15092343 | MSX1 | TSS1500 | Island | 1.97E-02 | 6.43E-08 |
| cg15093194 | DDX23 | TSS1500 | S_Shore | -8.57E-03 | 6.70E-05 |
| cg15102821 | CALML6 | TSS1500 | NA | -8.40E-03 | 3.27E-07 |
| cg15110296 | LOH12CR1;LOH12CF | TSS1500;Body | N_Shore | -2.06E-02 | 9.02E-13 |
| cg15113803 | RHO | TSS1500 | NA | -6.62E-03 | 6.04E-05 |
| cg15120333 | NA | NA | N_Shelf | 9.24E-03 | 2.33E-06 |
| cg15121267 | PRL;PRL | Body;Body | NA | -1.17E-02 | 2.48E-06 |
| cg15121420 | RAB17 | Body | NA | -1.09E-02 | 3.39E-11 |
| cg15123519 | LCT | Body | NA | -1.52E-02 | 8.74E-11 |
| cg15123692 | KLHL29 | Body | NA | 2.02E-02 | 1.22E-08 |
| cg15125438 | TPCN1;TPCN1 | Body;Body | NA | -6.72E-03 | 7.88E-06 |
| cg15133719 | PAX3;PAX3;PAX3;CC | TSS200;TSS200;TSS: | Island | 8.89E-03 | 8.59E-07 |
| cg15137369 | LRP12;LRP12 | TSS1500;TSS1500 | S_Shore | 1.04E-02 | 5.02E-09 |
| cg15138614 | SYT8 | Body | NA | -1.25E-02 | 2.09E-09 |
| cg15139254 | TMEM104 | Body | N_Shelf | -8.19E-03 | 4.56E-06 |
| cg15148394 | NA | NA | S_Shelf | -1.44E-02 | 1.48E-12 |
| cg15161667 | LRP4 | TSS1500 | S_Shore | -8.74E-03 | 1.18E-08 |
| cg15168727 | NA | NA | Island | 9.01E-03 | 6.17E-07 |
| cg15172061 | GSDMD | 5'UTR | N_Shore | 8.12E-03 | 9.32E-07 |
| cg15174623 | NA | NA | Island | 9.58E-03 | 5.80E-07 |
| cg15175581 | FMN1 | TSS200 | NA | -1.10E-02 | 1.53E-05 |
| cg15180789 | SCHIP1 | Body | NA | -1.25E-02 | 1.88E-05 |
| cg15189715 | NA | NA | N_Shore | -1.61E-02 | 2.04E-07 |
| cg15195412 | CX3CL1 | Body | NA | -7.85E-03 | 1.18E-08 |
| cg15200445 | PIP5K1C | Body | N_Shore | -6.73E-03 | 1.64E-05 |
| cg15201877 | PTGER3;PTGER3;PT( | 1stExon;Body;1stEx | Island | 7.59E-03 | 7.38E-05 |
| cg15237494 | ADAMTS5;ADAMTS! | 1stExon;5'UTR | Island | 9.73E-03 | 1.49E-05 |
| cg15243034 | USP35 | Body | Island | 2.91E-02 | 3.44E-16 |
| cg15243578 | PITPNM2 | 3'UTR | N_Shore | -1.01E-02 | 1.94E-09 |
| cg15261665 | LTF;LTF | 5'UTR;1stExon | Island | 9.13E-03 | 8.78E-06 |
| cg15270654 | GDF6 | Body | N_Shore | 9.81E-03 | 8.96E-07 |
| cg15272362 | VSX1;VSX1 | TSS200;TSS200 | Island | 1.54E-02 | 1.55E-06 |
| cg15277914 | DHX36;DHX36 | TSS1500;TSS1500 | S_Shore | -1.46E-02 | 1.36E-05 |
| cg15297650 | TMEM163 | TSS1500 | S_Shore | -8.08E-03 | 1.32E-09 |
| cg15298486 | BLOC1S2;BLOC1S2 | TSS1500;TSS1500 | S_Shore | -1.17E-02 | 5.82E-10 |
| cg15302379 | KAZALD1;KAZALD1 | 1stExon;5'UTR | Island | -9.19E-03 | 5.38E-05 |
| cg15312943 | SERGEF | Body | NA | -7.63E-03 | 1.63E-08 |
| cg15319457 | HES6;HES6 | Body;Body | Island | 3.05E-02 | 1.19E-05 |
| cg15333426 | NA | NA | Island | 9.68E-03 | 4.69E-05 |
| cg15341124 | DIO3;DIO3;MIR1247 | 5'UTR;1stExon;TSS1 | Island | 8.50E-03 | 1.43E-09 |
| cg15345741 | NA | NA | Island | 8.30E-03 | 1.66E-05 |
| cg15347189 | SST | 1stExon | Island | 9.92E-03 | 9.93E-06 |
| cg15356553 | MUC2 | Body | Island | -8.10E-03 | 8.27E-06 |
| cg15357821 | HK3 | Body | S_Shelf | -8.74E-03 | 2.60E-07 |
| cg15360181 | SLC9A9 | TSS200 | NA | -3.57E-02 | 5.83E-12 |
| cg15360771 | NA | NA | NA | -9.91E-03 | 3.30E-13 |
| cg15376996 | SBF1 | Body | Island | -7.45E-03 | 7.87E-05 |
| cg15389519 | PCDHGB4;PCDHGA4 | 1stExon;Body;Body, | S_Shore | 7.55E-03 | 7.02E-05 |
| cg15393702 | ANKRD29 | TSS1500 | S_Shore | -2.00E-02 | 1.28E-16 |
| cg15398497 | C2orf70 | Body | S_Shore | -7.74E-03 | 9.30E-08 |

| | | | | | |
|---|---|---|---|---|---|
| cg15408237 | NA | NA | NA | -2.26E-02 | 1.27E-06 |
| cg15425280 | GRIA2;GRIA2;GRIA2 | TSS1500;TSS1500;T | Island | 7.52E-03 | 5.28E-05 |
| cg15448975 | CRMP1 | TSS200 | Island | 9.65E-03 | 4.93E-05 |
| cg15449870 | AGTRAP;AGTRAP;AC | TSS200;TSS200;TSS: | Island | -8.05E-03 | 9.34E-06 |
| cg15451045 | TNFSF9 | TSS1500 | N_Shore | -1.04E-02 | 1.47E-08 |
| cg15452970 | NA | NA | Island | 7.81E-03 | 4.60E-05 |
| cg15457058 | FOXE3 | 1stExon | Island | 4.43E-02 | 6.01E-08 |
| cg15475851 | INA | 1stExon | Island | 3.17E-02 | 3.94E-10 |
| cg15480367 | CHGA;CHGA | 5'UTR;1stExon | Island | 1.05E-02 | 9.35E-17 |
| cg15484033 | NA | NA | NA | -1.33E-02 | 2.72E-11 |
| cg15495372 | ZNF579 | TSS1500 | S_Shelf | -1.50E-02 | 3.96E-09 |
| cg15500259 | NA | NA | N_Shore | -8.01E-03 | 1.08E-06 |
| cg15504461 | NA | NA | N_Shore | 1.18E-02 | 1.52E-08 |
| cg15512289 | NA | NA | NA | -1.05E-02 | 3.27E-06 |
| cg15519786 | PRDM16;PRDM16 | Body;Body | NA | -1.05E-02 | 1.20E-07 |
| cg15529692 | BMP7 | Body | Island | 2.23E-02 | 3.70E-09 |
| cg15538427 | LRRN4CL;LRRN4CL | 5'UTR;1stExon | S_Shore | -1.20E-02 | 5.11E-24 |
| cg15540371 | NA | NA | Island | -8.70E-03 | 2.95E-05 |
| cg15541630 | PSKH2 | 1stExon | Island | 1.58E-02 | 2.90E-09 |
| cg15557036 | NA | NA | Island | 8.44E-03 | 1.18E-05 |
| cg15562220 | SCGN | TSS200 | Island | 1.88E-02 | 1.31E-18 |
| cg15564098 | EVX1 | TSS1500 | N_Shore | 1.53E-02 | 1.09E-06 |
| cg15571353 | LOC284632 | Body | NA | -9.50E-03 | 3.22E-07 |
| cg15576918 | SLC6A20;SLC6A20 | Body;Body | N_Shelf | -7.08E-03 | 1.03E-06 |
| cg15577126 | NA | NA | NA | -9.27E-03 | 2.90E-07 |
| cg15580724 | FLJ32810 | TSS1500 | Island | 2.97E-02 | 2.32E-06 |
| cg15588941 | SLC25A20 | TSS200 | S_Shore | -1.39E-02 | 8.93E-18 |
| cg15594967 | HMGCLL1;HMGCLL1 | TSS200;TSS200 | Island | 1.20E-02 | 2.83E-10 |
| cg15600488 | MTMR7 | TSS200 | Island | 1.20E-02 | 7.06E-10 |
| cg15611112 | CAPN11 | TSS1500 | NA | -1.15E-02 | 6.79E-11 |
| cg15614917 | TM6SF2 | TSS200 | Island | 8.56E-03 | 1.84E-07 |
| cg15616915 | CTDSP1;MIR26B;CTI | Body;TSS1500;Body | S_Shore | -6.66E-03 | 3.74E-07 |
| cg15618978 | TRIM59 | TSS1500 | Island | 1.95E-02 | 5.63E-15 |
| cg15626285 | C1S;C1S | TSS200;TSS200 | NA | -2.79E-02 | 1.60E-12 |
| cg15633035 | IL6R;IL6R | 3'UTR;3'UTR | NA | -1.46E-02 | 5.39E-11 |
| cg15640503 | NA | NA | S_Shore | -1.51E-02 | 8.62E-12 |
| cg15644324 | HIVEP3;HIVEP3 | TSS200;TSS200 | S_Shore | -3.83E-02 | 6.85E-06 |
| cg15644764 | SYT2;SYT2 | 5'UTR;5'UTR | NA | -7.80E-03 | 1.11E-06 |
| cg15650298 | SCRIB;SCRIB;MIR937 | Body;Body;TSS1500 | N_Shore | -5.83E-03 | 2.03E-07 |
| cg15653478 | FAT3 | Body | NA | -1.31E-02 | 5.89E-06 |
| cg15657588 | TNXB | 5'UTR | NA | -5.63E-03 | 2.03E-05 |
| cg15657936 | GPR78 | 1stExon | Island | 1.97E-02 | 2.93E-10 |
| cg15662768 | HRH3 | TSS1500 | Island | 9.89E-03 | 3.02E-05 |
| cg15672768 | PCDHGA4;PCDHGA2 | Body;Body;1stExon, | Island | 1.62E-02 | 3.27E-07 |
| cg15677293 | EDARADD;EDARADD | Body;Body | S_Shore | -1.39E-02 | 9.52E-07 |
| cg15681626 | NA | NA | Island | 2.02E-02 | 8.33E-05 |
| cg15688551 | CHST8;CHST8;CHST8 | 5'UTR;5'UTR;TSS15( | N_Shore | 1.01E-02 | 6.40E-06 |
| cg15694605 | NA | NA | Island | -1.16E-02 | 8.37E-05 |
| cg15695155 | KDM2B;KDM2B | Body;Body | N_Shore | -1.46E-02 | 4.50E-07 |
| cg15700989 | NA | NA | NA | -1.01E-02 | 3.40E-05 |

| | | | | | |
|---|---|---|---|---|---|
| cg15701281 | NA | NA | S_Shore | -8.08E-03 | 4.88E-05 |
| cg15704699 | NLK | Body | NA | -1.99E-02 | 2.60E-26 |
| cg15707093 | NBLA00301;HAND2 | TSS1500;1stExon | Island | 1.85E-02 | 2.43E-05 |
| cg15711288 | NA | NA | S_Shelf | -1.36E-02 | 4.97E-13 |
| cg15718066 | PALLD;PALLD;PALLD | 1stExon;5'UTR;Body | Island | 9.95E-03 | 5.90E-09 |
| cg15722438 | HNRNPM;HNRNPM | Body;Body | S_Shore | 8.06E-03 | 1.26E-09 |
| cg15728320 | NA | NA | NA | -1.38E-02 | 1.89E-17 |
| cg15730857 | ST7OT4;ST7OT1;ST7 | TSS1500;Body;TSS1 | N_Shore | -1.25E-02 | 8.03E-14 |
| cg15736994 | ACCN5 | Body | NA | -2.20E-02 | 2.34E-18 |
| cg15746719 | DAPK1 | 5'UTR | Island | 3.19E-02 | 1.97E-05 |
| cg15754084 | PEX5;PEX5;PEX5;PE) | Body;Body;Body;Bo | S_Shore | -8.78E-03 | 2.60E-07 |
| cg15756114 | NA | NA | NA | -1.23E-02 | 1.67E-10 |
| cg15759721 | MIR21 | Body | NA | -9.40E-03 | 2.41E-07 |
| cg15777760 | C16orf53;MVP;MVP | 3'UTR;5'UTR;5'UTR | S_Shelf | -7.39E-03 | 4.98E-05 |
| cg15789607 | MIR7-3;C19orf30 | TSS1500;Body | NA | 1.46E-02 | 3.38E-08 |
| cg15790214 | HCG11 | Body | Island | 1.63E-02 | 3.95E-05 |
| cg15797286 | NAT1;NAT1;NAT1;N | TSS1500;TSS1500;T | NA | -8.14E-03 | 7.43E-05 |
| cg15802091 | HSPA2 | 1stExon | Island | 1.10E-02 | 4.05E-08 |
| cg15804973 | MAP3K5 | TSS1500 | S_Shore | -1.73E-02 | 5.09E-12 |
| cg15818604 | FAM92A1 | Body | S_Shore | -1.42E-02 | 2.72E-10 |
| cg15826479 | RPTOR;RPTOR | Body;Body | NA | -1.23E-02 | 3.12E-16 |
| cg15829826 | FRMD8 | TSS200 | Island | -1.08E-02 | 3.37E-06 |
| cg15837212 | NA | NA | Island | 6.35E-03 | 3.40E-05 |
| cg15842276 | MTNR1B | TSS200 | N_Shore | 7.20E-03 | 3.64E-06 |
| cg15843567 | MYO3A;MYO3A | 1stExon;5'UTR | Island | 2.56E-02 | 5.88E-05 |
| cg15845821 | NWD1 | TSS200 | NA | -2.49E-02 | 1.46E-37 |
| cg15852312 | NA | NA | Island | 1.10E-02 | 3.05E-05 |
| cg15857661 | MIR146B | TSS200 | S_Shelf | -1.84E-02 | 1.04E-19 |
| cg15871086 | NA | NA | N_Shelf | -1.57E-02 | 9.54E-16 |
| cg15887709 | NA | NA | Island | 1.00E-02 | 1.11E-10 |
| cg15890469 | PGF | 3'UTR | NA | -1.18E-02 | 1.60E-08 |
| cg15893346 | GUSB | TSS1500 | S_Shore | -1.02E-02 | 2.09E-10 |
| cg15895690 | NA | NA | Island | 7.91E-03 | 1.53E-06 |
| cg15931949 | NA | NA | N_Shore | 9.24E-03 | 2.59E-05 |
| cg15937073 | HIVEP3;HIVEP3 | TSS200;TSS200 | S_Shore | -1.44E-02 | 1.26E-06 |
| cg15948836 | LDLRAD3 | Body | NA | -9.37E-03 | 1.94E-08 |
| cg15957394 | AFAP1;AFAP1 | TSS200;TSS200 | Island | 1.04E-02 | 2.45E-07 |
| cg15961920 | MRPS18B;PPP1R10 | Body;TSS1500 | S_Shore | -1.60E-02 | 1.48E-05 |
| cg15962267 | SNHG4;SNORA74A;I | Body;TSS1500;5'UT | S_Shelf | -1.23E-02 | 7.83E-14 |
| cg15979173 | BARHL2 | 1stExon | Island | 9.20E-03 | 6.15E-07 |
| cg15985184 | IGLON5 | Body | Island | 1.40E-02 | 8.24E-06 |
| cg15989810 | GPR133 | Body | NA | -1.05E-02 | 6.38E-08 |
| cg15993345 | NA | NA | N_Shelf | -5.68E-03 | 2.25E-05 |
| cg15996534 | LOC134466 | Body | Island | 1.14E-02 | 1.55E-09 |
| cg16001165 | NA | NA | NA | -1.61E-02 | 8.64E-15 |
| cg16005494 | WBSCR17 | TSS200 | Island | 8.56E-03 | 2.06E-05 |
| cg16006701 | MYLK4 | TSS1500 | NA | -1.63E-02 | 3.82E-18 |
| cg16008966 | NA | NA | NA | -1.68E-02 | 6.59E-26 |
| cg16015113 | KIAA0319L;NCDN;N | TSS1500;Body;5'UT | S_Shore | -1.20E-02 | 3.53E-08 |
| cg16015712 | ZYG11A | 1stExon | Island | 1.56E-02 | 8.45E-07 |

| | | | | | |
|---|---|---|---|---|---|
| cg16025567 | TBCCD1;TBCCD1 | TSS200;5'UTR | Island | -1.02E-02 | 5.69E-07 |
| cg16031065 | MTSS1L | Body | Island | -8.66E-03 | 4.20E-08 |
| cg16031846 | NA | NA | NA | -8.90E-03 | 2.30E-08 |
| cg16038120 | FOXG1 | TSS1500 | Island | 2.18E-02 | 3.32E-11 |
| cg16047471 | BHLHE41 | 3'UTR | N_Shore | -1.36E-02 | 7.94E-12 |
| cg16054275 | F5 | TSS1500 | NA | -1.36E-02 | 3.43E-14 |
| cg16061099 | BRUNOL4;BRUNOL4 | 3'UTR;3'UTR;3'UTR; | N_Shelf | -1.13E-02 | 3.50E-14 |
| cg16074643 | NT5DC1 | Body | S_Shore | -8.77E-03 | 2.00E-05 |
| cg16081388 | NA | NA | Island | 6.19E-03 | 8.58E-06 |
| cg16085531 | NA | NA | Island | 4.89E-03 | 3.09E-05 |
| cg16086559 | NA | NA | Island | 1.56E-02 | 3.30E-07 |
| cg16095155 | TCF19;TCF19 | Body;Body | S_Shore | -9.99E-03 | 4.79E-09 |
| cg16104584 | SLC2A5 | TSS1500 | NA | -7.49E-03 | 2.28E-05 |
| cg16107285 | SYNM;SYNM | TSS1500;TSS1500 | Island | 9.66E-03 | 7.25E-10 |
| cg16109680 | CADM3;CADM3 | Body;Body | S_Shelf | -1.03E-02 | 2.32E-12 |
| cg16169675 | NA | NA | N_Shelf | -2.07E-02 | 8.92E-10 |
| cg16178603 | MEIS1 | Body | NA | 3.21E-02 | 2.23E-05 |
| cg16179952 | SNTG2 | Body | Island | 9.20E-03 | 1.19E-08 |
| cg16180082 | TAGAP;TAGAP;TAG/ | TSS1500;TSS1500;T | NA | -1.41E-02 | 6.66E-07 |
| cg16181383 | COL23A1 | Body | NA | -6.71E-03 | 2.63E-05 |
| cg16185386 | NA | NA | NA | -1.38E-02 | 1.23E-11 |
| cg16185666 | WDFY2 | TSS1500 | N_Shore | -8.58E-03 | 2.08E-06 |
| cg16193295 | FHOD3 | Body | N_Shore | -1.59E-02 | 3.25E-16 |
| cg16201146 | NA | NA | N_Shore | -1.93E-02 | 1.70E-23 |
| cg16204717 | NA | NA | NA | -1.50E-02 | 8.80E-17 |
| cg16205854 | EDN3;EDN3;EDN3;E | TSS200;TSS200;TSS: | Island | 5.81E-03 | 1.19E-05 |
| cg16207263 | NA | NA | NA | -1.09E-02 | 1.79E-09 |
| cg16209444 | ACOX2 | 5'UTR | NA | -8.31E-03 | 4.20E-10 |
| cg16209664 | ZIC1 | Body | Island | 8.90E-03 | 3.52E-06 |
| cg16218721 | HOXD4 | Body | Island | 1.10E-02 | 7.16E-11 |
| cg16219603 | PENK | TSS1500 | Island | 1.04E-02 | 5.87E-13 |
| cg16226962 | TFAP2A;TFAP2A | Body;TSS1500 | N_Shore | 8.07E-03 | 1.24E-08 |
| cg16232126 | SLC5A7;SLC5A7 | 1stExon;5'UTR | Island | 2.14E-02 | 2.37E-07 |
| cg16236328 | NA | NA | NA | -2.21E-02 | 1.98E-06 |
| cg16240162 | ZIC4;ZIC4;ZIC4;ZIC4; | Body;Body;Body;Bo | N_Shore | 1.62E-02 | 1.98E-11 |
| cg16243652 | NA | NA | NA | -1.57E-02 | 3.54E-33 |
| cg16254309 | CNTNAP2 | Body | S_Shore | 1.38E-02 | 5.43E-06 |
| cg16271600 | JUB | 1stExon | Island | 1.36E-02 | 9.97E-07 |
| cg16278496 | C14orf64 | TSS200 | NA | -1.26E-02 | 2.61E-05 |
| cg16288101 | NA | NA | NA | -2.37E-02 | 1.77E-08 |
| cg16290275 | NA | NA | S_Shore | -9.96E-03 | 3.05E-07 |
| cg16313944 | ALDH3B1 | TSS200 | NA | -6.67E-03 | 7.00E-06 |
| cg16323609 | PRKAR2A | TSS1500 | S_Shore | -6.11E-03 | 1.40E-05 |
| cg16340422 | PLD6 | TSS1500 | Island | -4.09E-02 | 2.46E-15 |
| cg16348158 | PRDM16;PRDM16 | Body;Body | N_Shore | -8.66E-03 | 1.64E-05 |
| cg16349774 | C1orf170 | Body | N_Shelf | -1.24E-02 | 1.82E-11 |
| cg16355231 | PEX10;PEX10 | TSS1500;TSS1500 | N_Shore | -2.48E-02 | 4.26E-13 |
| cg16361302 | NA | NA | Island | 1.28E-02 | 4.33E-05 |
| cg16361966 | NA | NA | Island | 6.68E-03 | 2.35E-07 |
| cg16373229 | FEZF1;FEZF1 | Body;Body | N_Shore | 8.24E-03 | 1.92E-08 |

| | | | | | |
|---|---|---|---|---|---|
| cg16376108 | IRX5 | Body | Island | 7.72E-03 | 2.24E-07 |
| cg16385335 | IL18BP;IL18BP;IL18E | TSS200;5'UTR;TSS1! | NA | -1.48E-02 | 3.73E-16 |
| cg16386080 | CDK20;CDK20;CDK2 | Body;Body;Body;Bo | N_Shore | 5.21E-03 | 1.02E-05 |
| cg16392391 | NHEG1 | Body | NA | -1.40E-02 | 2.31E-15 |
| cg16404157 | CLEC14A | 1stExon | Island | 1.54E-02 | 2.75E-05 |
| cg16407998 | MAGI2 | Body | NA | -1.13E-02 | 6.77E-12 |
| cg16408865 | YEATS4 | TSS1500 | N_Shore | -1.51E-02 | 3.45E-09 |
| cg16410171 | RNASE7 | 5'UTR | NA | -7.11E-03 | 2.62E-07 |
| cg16412573 | RGS6 | Body | NA | -6.81E-03 | 7.60E-06 |
| cg16416715 | BRD2;BRD2 | Body;Body | S_Shore | -1.42E-02 | 6.00E-08 |
| cg16417409 | NA | NA | S_Shelf | -1.76E-02 | 2.59E-06 |
| cg16419235 | PENK | TSS1500 | Island | 1.84E-02 | 1.04E-14 |
| cg16428251 | SOX14 | TSS200 | Island | 9.51E-03 | 2.70E-07 |
| cg16428357 | FER1L4 | Body | Island | 2.25E-02 | 8.56E-06 |
| cg16431352 | NA | NA | NA | -1.05E-02 | 2.21E-11 |
| cg16431787 | LOC285768;LOC285 | Body;Body | NA | -4.28E-02 | 7.58E-18 |
| cg16441259 | NA | NA | Island | 7.70E-03 | 5.69E-06 |
| cg16449262 | NA | NA | N_Shore | -1.22E-02 | 6.18E-07 |
| cg16454902 | IL21R;IL21R | TSS200;5'UTR | NA | -1.27E-02 | 6.45E-10 |
| cg16456596 | NA | NA | NA | -7.29E-03 | 1.14E-05 |
| cg16464007 | LPP;LPP;LPP | 5'UTR;5'UTR;5'UTR | NA | -1.13E-02 | 1.20E-11 |
| cg16477091 | PPM1E | TSS1500 | Island | 1.51E-02 | 4.72E-11 |
| cg16494108 | WDR76;WDR76 | 5'UTR;Body | S_Shore | -1.12E-02 | 4.43E-08 |
| cg16498194 | FZD10 | 1stExon | Island | 1.18E-02 | 3.49E-09 |
| cg16498741 | SFRP1 | Body | NA | -1.24E-02 | 8.54E-12 |
| cg16502931 | NA | NA | N_Shelf | -9.27E-03 | 1.33E-05 |
| cg16509239 | AHNAK | Body | S_Shore | -6.20E-03 | 6.04E-06 |
| cg16512661 | NA | NA | S_Shelf | 1.58E-02 | 4.09E-12 |
| cg16519217 | NA | NA | NA | -1.87E-02 | 4.59E-14 |
| cg16519487 | NA | NA | Island | 9.00E-03 | 7.78E-06 |
| cg16521917 | CNTNAP2;CNTNAP2 | 1stExon;5'UTR | Island | 1.74E-02 | 7.75E-05 |
| cg16529477 | PAX3;PAX3;PAX3;PA | TSS1500;TSS1500;T | S_Shore | 1.36E-02 | 2.16E-07 |
| cg16536329 | ZNF454 | TSS200 | Island | 1.31E-02 | 1.45E-08 |
| cg16541026 | P4HTM;P4HTM | TSS1500;TSS1500 | N_Shore | -1.03E-02 | 2.91E-09 |
| cg16541931 | LOC100128811;GPR | Body;TSS1500 | Island | 1.32E-02 | 1.29E-11 |
| cg16554099 | NA | NA | S_Shore | -2.20E-02 | 1.13E-20 |
| cg16556442 | NA | NA | Island | 8.17E-03 | 4.53E-06 |
| cg16563171 | PAX3;PAX3;PAX3;PA | Body;Body;Body;Bo | Island | 9.35E-03 | 8.18E-07 |
| cg16565409 | RPL23A;SNORD4A | Body;TSS1500 | S_Shore | -1.63E-02 | 3.09E-05 |
| cg16569273 | NA | NA | NA | 1.02E-02 | 1.12E-05 |
| cg16572910 | NA | NA | NA | -1.03E-02 | 1.23E-18 |
| cg16577789 | PRIMA1 | 3'UTR | NA | -6.75E-03 | 3.65E-07 |
| cg16578267 | HES4;HES4 | TSS1500;TSS1500 | Island | 1.22E-02 | 4.20E-05 |
| cg16581017 | MYL10 | 3'UTR | NA | -8.20E-03 | 1.71E-06 |
| cg16581347 | NA | NA | N_Shore | 1.16E-02 | 1.61E-08 |
| cg16582779 | FOXG1 | 1stExon | Island | 7.42E-03 | 2.92E-10 |
| cg16584393 | ANKRD30B | TSS1500 | Island | 9.93E-03 | 8.07E-05 |
| cg16590237 | NA | NA | Island | 1.49E-02 | 7.75E-06 |
| cg16593060 | NA | NA | N_Shore | -6.06E-03 | 7.14E-06 |
| cg16598405 | BAT3;BAT3;BAT3;BA | TSS1500;TSS1500;T | S_Shore | -1.40E-02 | 2.79E-07 |

| | | | | | |
|---|---|---|---|---|---|
| cg16599136 | NA | NA | Island | 8.49E-03 | 4.72E-07 |
| cg16604233 | NA | NA | N_Shore | -1.38E-02 | 2.62E-10 |
| cg16606731 | SYNE1;SYNE1 | TSS1500;TSS200 | S_Shore | -1.03E-02 | 6.97E-07 |
| cg16615154 | NA | NA | Island | 1.47E-02 | 1.18E-09 |
| cg16616340 | TSGA10IP | TSS1500 | NA | -7.79E-03 | 4.73E-09 |
| cg16616467 | CPNE7;CPNE7 | Body;Body | Island | 2.37E-02 | 4.58E-07 |
| cg16620537 | PCDHA7;PCDHAC1;F | Body;TSS1500;Body | Island | 8.94E-03 | 9.35E-06 |
| cg16622061 | NA | NA | NA | -1.10E-02 | 2.54E-06 |
| cg16624069 | DOCK9;DOCK9;DOC | Body;5'UTR;1stExon | NA | -1.43E-02 | 6.34E-09 |
| cg16624521 | NA | NA | N_Shelf | -2.08E-02 | 8.72E-19 |
| cg16630369 | MAST4;MAST4 | Body;Body | NA | -1.30E-02 | 7.31E-09 |
| cg16637224 | NA | NA | NA | -1.06E-02 | 1.55E-10 |
| cg16638523 | MARCH4;MARCH4 | 5'UTR;1stExon | Island | 1.07E-02 | 7.05E-05 |
| cg16640358 | NA | NA | S_Shelf | -8.34E-03 | 4.28E-12 |
| cg16644497 | ZNF599 | Body | Island | 2.92E-02 | 2.24E-06 |
| cg16655765 | C6orf186 | TSS1500 | S_Shore | -1.73E-02 | 2.71E-07 |
| cg16655791 | NA | NA | Island | 9.35E-03 | 5.73E-09 |
| cg16664584 | NA | NA | NA | -6.89E-03 | 4.49E-05 |
| cg16664617 | SLC27A1 | Body | N_Shore | -1.37E-02 | 1.77E-18 |
| cg16668176 | PTPRS;PTPRS;PTPRS | 5'UTR;5'UTR;5'UTR; | NA | -1.40E-02 | 2.55E-11 |
| cg16671079 | MGAT5B;MGAT5B | Body;TSS200 | S_Shore | -7.87E-03 | 4.61E-08 |
| cg16671365 | CSMD1 | Body | S_Shelf | -1.19E-02 | 8.68E-12 |
| cg16677955 | ZBTB8B | TSS1500 | N_Shore | 1.03E-02 | 4.22E-06 |
| cg16682903 | ACVR1;ACVR1 | 5'UTR;5'UTR | NA | -1.54E-02 | 5.25E-09 |
| cg16686396 | PRDM16;PRDM16 | Body;Body | NA | -8.62E-03 | 5.23E-06 |
| cg16689232 | NA | NA | S_Shelf | -1.18E-02 | 1.54E-09 |
| cg16690876 | AKAP10 | Body | NA | -2.28E-02 | 7.10E-07 |
| cg16695548 | URGCP;URGCP;URG | 5'UTR;TSS1500;TSS1 | S_Shore | -1.55E-02 | 6.77E-07 |
| cg16705627 | SOX1;SOX1 | 1stExon;5'UTR | Island | 1.34E-02 | 4.16E-07 |
| cg16708465 | ABCC4;ABCC4 | Body;Body | NA | -1.48E-02 | 7.91E-16 |
| cg16717122 | SCG3;SCG3;SCG3;SC | 5'UTR;1stExon;5'UT | S_Shore | 7.21E-03 | 6.78E-07 |
| cg16717225 | GLT1D1 | TSS1500 | N_Shore | 5.08E-03 | 6.87E-05 |
| cg16718445 | PDGFRA | 5'UTR | Island | 1.48E-02 | 4.59E-05 |
| cg16720379 | NA | NA | NA | -1.12E-02 | 2.75E-08 |
| cg16725944 | POLE | Body | NA | -6.02E-03 | 5.61E-07 |
| cg16733705 | NA | NA | NA | 2.44E-02 | 2.03E-10 |
| cg16736964 | NA | NA | NA | -1.26E-02 | 4.17E-08 |
| cg16737749 | MAPK10 | 5'UTR | NA | -1.24E-02 | 6.50E-06 |
| cg16742481 | NA | NA | NA | -5.18E-03 | 5.50E-05 |
| cg16744741 | PRKG2 | 1stExon | NA | -9.13E-03 | 1.48E-07 |
| cg16762735 | ZFP42 | TSS200 | Island | 1.05E-02 | 3.29E-07 |
| cg16765088 | NA | NA | N_Shore | -6.95E-03 | 1.11E-06 |
| cg16766249 | NRXN3;NRXN3;NRXI | Body;TSS1500;TSS1 | Island | 1.31E-02 | 4.62E-06 |
| cg16766914 | CUEDC1 | Body | Island | 2.41E-02 | 9.95E-05 |
| cg16768018 | ZIC4;ZIC4;ZIC4;ZIC4; | Body;Body;Body;Bo | Island | 2.38E-02 | 9.49E-09 |
| cg16776874 | C17orf90;CCDC137 | Body;TSS1500 | N_Shore | 4.76E-03 | 2.50E-06 |
| cg16782084 | NA | NA | NA | -1.39E-02 | 4.47E-17 |
| cg16783186 | NA | NA | NA | -1.08E-02 | 1.56E-05 |
| cg16786458 | PPARGC1B | TSS1500 | N_Shore | -1.19E-02 | 1.17E-07 |
| cg16791210 | ELMOD3;ELMOD3;E | TSS1500;TSS1500;T | N_Shore | -9.42E-03 | 6.16E-08 |

| | | | | | |
|---|---|---|---|---|---|
| cg16803083 | NA | NA | Island | 2.11E-02 | 7.27E-05 |
| cg16810343 | SRRM3 | Body | S_Shelf | -1.58E-02 | 1.05E-14 |
| cg16810559 | PRICKLE2 | Body | NA | -9.54E-03 | 4.67E-06 |
| cg16829924 | PRR5L | 5'UTR | NA | -1.98E-02 | 7.67E-06 |
| cg16832267 | KCNS3 | 5'UTR | Island | 1.98E-02 | 1.31E-05 |
| cg16833797 | TMTC1 | Body | NA | -1.57E-02 | 6.06E-10 |
| cg16850150 | RFTN1 | Body | NA | -1.46E-02 | 4.84E-11 |
| cg16852704 | TLX2 | Body | Island | 7.39E-03 | 1.87E-05 |
| cg16867657 | ELOVL2 | TSS1500 | Island | 2.80E-02 | 6.44E-34 |
| cg16879608 | EMP1 | 5'UTR | NA | -7.81E-03 | 2.30E-06 |
| cg16882373 | NFE2L3 | TSS1500 | N_Shore | -8.07E-03 | 7.42E-06 |
| cg16887334 | OXT | TSS200 | Island | -2.41E-02 | 3.52E-07 |
| cg16894819 | NA | NA | N_Shelf | -8.98E-03 | 5.55E-07 |
| cg16922279 | PHOX2A | TSS1500 | Island | 1.08E-02 | 4.66E-06 |
| cg16932827 | NA | NA | S_Shore | -1.87E-02 | 1.16E-20 |
| cg16933181 | CHST9 | TSS200 | Island | 1.24E-02 | 1.47E-06 |
| cg16936060 | HOPX;HOPX;HOPX;H | 5'UTR;Body;Body;TS | S_Shore | -9.27E-03 | 1.12E-05 |
| cg16952974 | NA | NA | Island | 6.98E-03 | 6.55E-05 |
| cg16960758 | NOSTRIN;NOSTRIN;I | 5'UTR;TSS200;TSS2( | NA | -2.65E-02 | 5.34E-40 |
| cg16962008 | C7orf57 | Body | Island | 1.49E-02 | 9.75E-09 |
| cg16983588 | PRDM10;PRDM10;P | Body;Body;Body;Bo | NA | -1.61E-02 | 1.09E-10 |
| cg16987606 | GPRC5C | TSS1500 | N_Shore | -7.52E-03 | 8.76E-05 |
| cg16987754 | NA | NA | Island | 2.03E-02 | 5.71E-07 |
| cg17003736 | ZIC4;ZIC4;ZIC4;ZIC4; | Body;TSS1500;Body | Island | 8.71E-03 | 4.08E-08 |
| cg17007640 | C14orf23;C14orf23 | Body;Body | Island | 1.39E-02 | 2.74E-08 |
| cg17024199 | MGAT5B;MGAT5B | Body;Body | S_Shelf | -8.85E-03 | 1.61E-09 |
| cg17024482 | ANKFN1 | TSS200 | NA | -9.51E-03 | 3.31E-08 |
| cg17034109 | CYB561D1;CYB561D | TSS1500;TSS1500;T | N_Shore | -7.54E-03 | 5.73E-09 |
| cg17035591 | APOBEC3F;APOBEC3 | Body;Body | NA | -1.06E-02 | 5.58E-21 |
| cg17051321 | BNC1 | Body | Island | 1.01E-02 | 4.65E-08 |
| cg17062829 | NA | NA | N_Shore | 1.01E-02 | 3.65E-05 |
| cg17071446 | NA | NA | N_Shore | 1.07E-02 | 2.79E-06 |
| cg17072494 | NA | NA | NA | -1.00E-02 | 2.15E-05 |
| cg17073733 | WNT1 | Body | Island | 4.87E-03 | 8.41E-05 |
| cg17074353 | PAQR5 | TSS1500 | N_Shore | 8.47E-03 | 5.51E-05 |
| cg17076890 | PTF1A | TSS200 | Island | 9.79E-03 | 1.69E-07 |
| cg17082959 | NA | NA | NA | -9.81E-03 | 3.10E-09 |
| cg17087331 | PPAPDC1A | 1stExon | Island | 9.16E-03 | 9.17E-05 |
| cg17094065 | TENC1;TENC1;TENC | TSS1500;5'UTR;Bod | S_Shore | -9.87E-03 | 3.30E-08 |
| cg17095489 | NA | NA | NA | -2.15E-02 | 1.62E-20 |
| cg17101296 | NA | NA | Island | 2.22E-02 | 4.61E-18 |
| cg17101703 | AAK1 | Body | NA | -1.09E-02 | 1.12E-14 |
| cg17110586 | NA | NA | S_Shelf | 1.93E-02 | 2.86E-35 |
| cg17111166 | NA | NA | NA | -1.84E-02 | 7.52E-14 |
| cg17112975 | SEPT9;SEPT9;SEPT9; | Body;Body;Body;Bo | NA | 4.82E-03 | 6.83E-06 |
| cg17118262 | CCL1 | TSS1500 | NA | -9.76E-03 | 1.60E-06 |
| cg17130251 | NA | NA | S_Shelf | -8.98E-03 | 5.04E-07 |
| cg17136255 | NA | NA | NA | -1.16E-02 | 1.26E-08 |
| cg17139392 | NA | NA | NA | -1.45E-02 | 1.40E-16 |
| cg17144149 | HOXB4 | TSS1500 | S_Shore | -1.34E-02 | 3.63E-09 |

| | | | | | |
|---|---|---|---|---|---|
| cg17145190 | NFIX | Body | N_Shore | -5.94E-03 | 3.96E-05 |
| cg17152981 | GPR6 | TSS1500 | Island | 6.28E-03 | 1.09E-06 |
| cg17156491 | NA | NA | NA | -8.26E-03 | 1.78E-05 |
| cg17165841 | LOC200726 | TSS200 | Island | 8.92E-03 | 4.40E-05 |
| cg17167852 | PCSK9 | TSS200 | Island | 2.01E-02 | 2.32E-05 |
| cg17168836 | GNG12 | 5'UTR | NA | -1.22E-02 | 1.37E-12 |
| cg17173423 | MS4A3;MS4A3;MS4 | TSS200;TSS200;TSS: | NA | -9.52E-03 | 1.62E-06 |
| cg17176609 | ACTG2 | Body | NA | -2.16E-02 | 3.67E-07 |
| cg17177632 | FAM123C;FAM123C | TSS1500;TSS200;TS: | N_Shore | 1.19E-02 | 2.97E-07 |
| cg17179923 | NA | NA | N_Shore | -1.91E-02 | 1.18E-05 |
| cg17180678 | NA | NA | Island | 9.40E-03 | 3.36E-05 |
| cg17192377 | HDHD2 | TSS1500 | S_Shore | -1.57E-02 | 3.09E-10 |
| cg17200920 | NA | NA | Island | 1.53E-02 | 2.29E-06 |
| cg17211261 | C10orf41;ZNF503 | TSS1500;Body | Island | 8.58E-03 | 2.46E-05 |
| cg17214381 | ZFP42 | TSS1500 | Island | 1.49E-02 | 8.36E-07 |
| cg17215278 | CD58;CD58;CD58 | TSS1500;TSS1500;T | S_Shore | -5.79E-03 | 3.52E-05 |
| cg17217996 | OSBPL2;OSBPL2 | Body;Body | S_Shelf | -6.01E-03 | 3.79E-05 |
| cg17228560 | KLHL10;NT5C3L | TSS1500;TSS1500 | S_Shore | -1.89E-02 | 5.69E-17 |
| cg17232357 | SMAD6;SMAD6;SM/ | Body;Body;Body | NA | -7.93E-03 | 7.53E-08 |
| cg17237086 | MKL1 | Body | Island | 5.27E-03 | 3.52E-05 |
| cg17248487 | AHRR | Body | NA | -6.52E-03 | 1.25E-05 |
| cg17261675 | C21orf29;KRTAP12-: | Body;TSS1500 | NA | -1.50E-02 | 7.26E-08 |
| cg17266805 | NA | NA | NA | -6.85E-03 | 3.12E-06 |
| cg17269633 | C17orf104 | TSS200 | Island | 1.54E-02 | 3.80E-07 |
| cg17271585 | BLCAP;BLCAP;BLCAF | 5'UTR;TSS1500;5'U' | N_Shelf | -1.05E-02 | 2.99E-13 |
| cg17280346 | ZIC1 | TSS1500 | N_Shore | 1.81E-02 | 2.09E-08 |
| cg17283453 | ZNF177 | 5'UTR | Island | 9.00E-03 | 2.16E-05 |
| cg17288397 | NA | NA | Island | 1.73E-02 | 5.85E-05 |
| cg17288471 | NA | NA | NA | -6.53E-03 | 6.67E-08 |
| cg17295225 | OLIG3 | 1stExon | Island | 1.10E-02 | 2.93E-05 |
| cg17301902 | GNA14;GNA14 | 1stExon;5'UTR | Island | 7.49E-03 | 1.09E-05 |
| cg17302155 | PRDM13 | Body | Island | 2.24E-02 | 1.45E-06 |
| cg17306747 | SLC6A3 | TSS1500 | Island | 8.44E-03 | 2.96E-06 |
| cg17321954 | STXBP5L | TSS200 | Island | 8.95E-03 | 9.33E-09 |
| cg17326451 | CROT;TP53TG1;CRO | 5'UTR;TSS1500;Bod | S_Shore | -1.80E-02 | 1.31E-13 |
| cg17326555 | NA | NA | Island | 1.29E-02 | 2.57E-08 |
| cg17328514 | NDUFB8 | TSS1500 | S_Shore | -1.72E-02 | 1.04E-18 |
| cg17329534 | ZBTB7B | 5'UTR | NA | -9.56E-03 | 9.45E-09 |
| cg17336354 | NA | NA | NA | -6.15E-03 | 1.91E-05 |
| cg17339521 | RFTN1 | TSS1500 | S_Shore | -7.67E-03 | 2.96E-05 |
| cg17340519 | CNIH3 | Body | Island | 1.22E-02 | 2.59E-09 |
| cg17341765 | NA | NA | NA | -2.21E-02 | 2.02E-08 |
| cg17343374 | GAK | Body | S_Shore | -1.24E-02 | 3.41E-11 |
| cg17344149 | NUP98;PGAP2;NUP! | 5'UTR;TSS1500;5'U' | N_Shore | -1.40E-02 | 1.95E-05 |
| cg17344640 | NA | NA | NA | -3.77E-02 | 2.76E-05 |
| cg17354303 | BARX1 | Body | Island | 5.60E-03 | 5.90E-07 |
| cg17368808 | C20orf103 | Body | Island | 7.40E-03 | 1.34E-05 |
| cg17372101 | CNTNAP2 | Body | NA | -1.15E-02 | 1.30E-10 |
| cg17373345 | DNAJC5B | 5'UTR | NA | -2.18E-02 | 2.11E-12 |
| cg17381377 | ABCC8 | TSS200 | Island | 1.14E-02 | 2.49E-06 |

| | | | | | |
|---|---|---|---|---|---|
| cg17384380 | NA | NA | Island | 1.15E-02 | 3.44E-08 |
| cg17403609 | FLT4;FLT4 | Body;Body | Island | 1.19E-02 | 8.21E-06 |
| cg17410236 | FLRT2;FLRT2 | 1stExon;5'UTR | Island | 2.46E-02 | 8.52E-05 |
| cg17411135 | NA | NA | N_Shore | 6.46E-03 | 4.28E-05 |
| cg17415650 | NA | NA | NA | -1.89E-02 | 1.09E-24 |
| cg17425144 | PEX14 | Body | NA | -1.40E-02 | 1.72E-09 |
| cg17427926 | LOC283999 | TSS200 | N_Shore | -9.12E-03 | 5.73E-07 |
| cg17430979 | JAKMIP1 | Body | NA | -2.05E-02 | 1.94E-22 |
| cg17434901 | NA | NA | NA | -4.97E-03 | 4.08E-06 |
| cg17436656 | RARG | TSS1500 | S_Shore | -1.21E-02 | 1.27E-14 |
| cg17444738 | NKX6-1 | Body | Island | 8.65E-03 | 2.64E-08 |
| cg17451586 | BRD2;BRD2 | Body;Body | S_Shore | -1.27E-02 | 5.68E-08 |
| cg17455088 | NRP2;NRP2;NRP2;N | Body;Body;Body;Bo | S_Shore | 7.19E-03 | 1.88E-05 |
| cg17471102 | FUT3;FUT3;FUT3;FU | 5'UTR;5'UTR;5'UTR; | NA | -1.42E-02 | 3.05E-23 |
| cg17478228 | ELAVL3;ELAVL3;ELA\ | 5'UTR;1stExon;1stE: | Island | 1.36E-02 | 4.66E-07 |
| cg17479956 | EXT2;EXT2 | TSS1500;TSS1500 | N_Shore | -1.37E-02 | 8.42E-09 |
| cg17485681 | CDH23 | Body | Island | 8.93E-03 | 8.22E-05 |
| cg17494199 | NA | NA | NA | -1.33E-02 | 6.79E-10 |
| cg17497271 | GPR176;GPR176 | 5'UTR;1stExon | Island | 1.21E-02 | 2.17E-09 |
| cg17499358 | NA | NA | NA | -1.42E-02 | 1.77E-06 |
| cg17508591 | PCDHA6;PCDHA2;P( | Body;Body;Body;1s | Island | 1.59E-02 | 9.24E-16 |
| cg17514528 | MTHFR | Body | N_Shelf | -1.24E-02 | 3.03E-10 |
| cg17529152 | C2orf76;DBI;DBI;DB | TSS1500;5'UTR;Bod | S_Shore | -6.91E-03 | 1.62E-08 |
| cg17533477 | ASNS;ASNS;ASNS | 5'UTR;5'UTR;5'UTR | N_Shore | -1.27E-02 | 3.52E-08 |
| cg17535550 | NA | NA | Island | 1.19E-02 | 1.30E-08 |
| cg17551891 | MAD1L1;MAD1L1;M | Body;Body;Body | NA | -1.43E-02 | 1.27E-07 |
| cg17563773 | POU2F3 | Body | NA | -8.69E-03 | 1.03E-07 |
| cg17567838 | SLC39A7;RXRB;SLC3 | TSS1500;Body;TSS1 | N_Shore | -8.06E-03 | 8.30E-10 |
| cg17589341 | SLC14A1;SLC14A1;S | TSS200;TSS200;TSS: | NA | -1.67E-02 | 6.60E-14 |
| cg17592231 | NA | NA | S_Shore | 1.15E-02 | 1.10E-22 |
| cg17593342 | NA | NA | NA | -2.49E-02 | 4.76E-23 |
| cg17593472 | FGF1;FGF1;FGF1;FG | 5'UTR;5'UTR;5'UTR; | NA | -9.55E-03 | 6.77E-09 |
| cg17603689 | NA | NA | Island | 1.06E-02 | 3.37E-06 |
| cg17605476 | GRIA2;GRIA2;GRIA2 | Body;Body;Body | Island | 9.92E-03 | 1.49E-07 |
| cg17645528 | NA | NA | S_Shelf | -1.03E-02 | 3.96E-06 |
| cg17662369 | TBC1D9B;TBC1D9B | Body;Body | N_Shore | -1.09E-02 | 3.71E-09 |
| cg17662872 | WFS1;WFS1 | TSS1500;TSS1500 | N_Shore | -1.23E-02 | 6.02E-11 |
| cg17665497 | NA | NA | N_Shelf | -1.13E-02 | 2.95E-11 |
| cg17672614 | C6orf176;C6orf176 | Body;Body | NA | -2.93E-02 | 3.04E-05 |
| cg17674725 | LYPD1;LYPD1 | 5'UTR;Body | N_Shore | 8.33E-03 | 7.33E-07 |
| cg17676618 | NA | NA | NA | -7.13E-03 | 2.44E-05 |
| cg17694795 | NA | NA | Island | 1.95E-02 | 4.06E-14 |
| cg17722002 | NA | NA | Island | 7.25E-03 | 2.46E-06 |
| cg17729667 | NINL | TSS1500 | Island | 1.08E-02 | 6.93E-09 |
| cg17732038 | NA | NA | S_Shelf | -1.01E-02 | 7.84E-11 |
| cg17737681 | DLX1;DLX1 | Body;Body | Island | 1.64E-02 | 2.92E-05 |
| cg17741799 | NA | NA | NA | -8.30E-03 | 1.70E-06 |
| cg17744295 | TPM4 | TSS1500 | N_Shore | -1.36E-02 | 9.66E-09 |
| cg17745234 | TMPRSS6;TMPRSS6 | 1stExon;5'UTR | NA | -1.10E-02 | 1.06E-09 |
| cg17754914 | SCN3A;SCN3A;SCN3 | TSS1500;TSS1500;T | NA | -1.35E-02 | 1.35E-13 |

| | | | | | |
|---|---|---|---|---|---|
| cg17760405 | NA | NA | Island | 1.85E-02 | 2.82E-08 |
| cg17768491 | SPTBN4 | Body | Island | 1.99E-02 | 2.89E-06 |
| cg17775079 | JMJD1C;LOC84989 | Body;TSS1500 | N_Shore | -1.29E-02 | 3.79E-09 |
| cg17775765 | NA | NA | N_Shore | 9.20E-03 | 4.67E-07 |
| cg17777676 | PCDHA2;PCDHA1;P( | Body;Body;Body;1s | Island | 1.66E-02 | 1.23E-09 |
| cg17783401 | RGS20 | Body | Island | 1.38E-02 | 6.17E-06 |
| cg17784808 | PXT1;KCTD20 | 5'UTR;TSS1500 | N_Shore | -1.43E-02 | 2.53E-13 |
| cg17786697 | PRDM6 | Body | N_Shore | 8.30E-03 | 1.00E-06 |
| cg17804071 | PDZRN3 | Body | NA | -1.28E-02 | 5.90E-12 |
| cg17816357 | NA | NA | NA | -2.00E-02 | 1.66E-11 |
| cg17816908 | SFRP1 | TSS200 | Island | 1.30E-02 | 7.14E-05 |
| cg17828689 | TMEM22;TMEM22; | 5'UTR;5'UTR;5'UTR | Island | 8.52E-03 | 6.37E-05 |
| cg17833265 | NA | NA | Island | 1.10E-02 | 5.37E-05 |
| cg17852932 | PDLIM4;PDLIM4 | Body;Body | S_Shelf | -8.04E-03 | 2.95E-06 |
| cg17857094 | DPCR1 | TSS1500 | NA | -2.51E-02 | 1.60E-06 |
| cg17861230 | PDE4C | Body | Island | 1.07E-02 | 7.74E-05 |
| cg17864091 | PRIC285;PRIC285 | Body;TSS1500 | Island | -1.31E-02 | 2.34E-12 |
| cg17877704 | NA | NA | Island | 2.17E-02 | 4.19E-06 |
| cg17879376 | BAI2 | Body | S_Shelf | -6.21E-03 | 1.27E-07 |
| cg17885226 | NA | NA | Island | 9.84E-03 | 1.52E-06 |
| cg17890940 | ELFN1 | 5'UTR | N_Shore | -1.45E-02 | 3.01E-05 |
| cg17892169 | TNFSF12;TNFSF12-T | Body;Body | Island | -6.06E-03 | 2.09E-05 |
| cg17903229 | KRTAP10-1;C21orf2 | 3'UTR;Body;1stExor | NA | -5.40E-03 | 5.52E-06 |
| cg17906168 | EFNB2 | 3'UTR | N_Shore | -2.68E-02 | 1.15E-16 |
| cg17918314 | ARTN;ARTN;ARTN | TSS1500;TSS1500;T | N_Shore | -6.30E-03 | 8.53E-05 |
| cg17920479 | SLC12A5 | TSS200 | N_Shore | 6.32E-03 | 2.01E-05 |
| cg17922283 | RGS6 | Body | NA | -8.14E-03 | 3.84E-07 |
| cg17943663 | CDK3 | 3'UTR | N_Shore | -5.50E-03 | 4.70E-05 |
| cg17959631 | GCK;GCK;GCK | Body;Body;Body | S_Shelf | -7.59E-03 | 6.32E-09 |
| cg17971747 | DUOXA1;DUOXA2 | Body;3'UTR | S_Shore | 7.36E-03 | 3.37E-06 |
| cg17978548 | NA | NA | NA | -1.04E-02 | 1.14E-07 |
| cg17980283 | NA | NA | Island | 1.05E-02 | 8.24E-05 |
| cg18006328 | NA | NA | Island | 1.01E-02 | 1.90E-05 |
| cg18016194 | RALYL;RALYL;RALYL; | 5'UTR;5'UTR;1stExo | Island | 9.60E-03 | 1.16E-07 |
| cg18018027 | NA | NA | N_Shore | 2.09E-02 | 2.04E-11 |
| cg18018313 | IRS1 | 3'UTR | S_Shore | -1.39E-02 | 6.39E-19 |
| cg18018890 | NA | NA | Island | 6.87E-03 | 4.32E-05 |
| cg18026631 | NA | NA | Island | 1.18E-02 | 9.99E-07 |
| cg18034299 | ERGIC1 | Body | NA | -1.17E-02 | 3.78E-16 |
| cg18037834 | TENC1;TENC1;TENC | 5'UTR;Body;Body | N_Shelf | -7.73E-03 | 2.10E-07 |
| cg18041884 | NA | NA | Island | 1.17E-02 | 4.40E-05 |
| cg18042586 | SLC39A14 | Body | NA | -1.30E-02 | 2.15E-10 |
| cg18045859 | CLDN3;CLDN3 | 5'UTR;1stExon | Island | 3.12E-02 | 8.45E-06 |
| cg18047353 | SLC14A2 | 5'UTR | NA | -1.29E-02 | 3.03E-05 |
| cg18054674 | PCDHGA4;PCDHGA1 | Body;Body;TSS200;I | N_Shore | 1.26E-02 | 3.71E-06 |
| cg18055585 | NTRK3;NTRK3;NTRK | Body;Body;Body | NA | -7.49E-03 | 1.89E-08 |
| cg18057109 | NA | NA | Island | 1.07E-02 | 1.24E-06 |
| cg18064714 | SP8;SP8 | Body;Body | Island | 5.75E-03 | 6.82E-05 |
| cg18070593 | MAP3K12 | 5'UTR | N_Shore | -1.37E-02 | 5.51E-17 |
| cg18077307 | TMEM131 | Body | NA | -1.40E-02 | 7.41E-07 |

| | | | | | |
|---|---|---|---|---|---|
| cg18087326 | GPX7 | TSS1500 | N_Shore | -9.16E-03 | 1.26E-05 |
| cg18087520 | CALM1 | TSS1500 | N_Shore | -1.65E-02 | 1.10E-09 |
| cg18091264 | SNORD18A;RPL4;SN | Body;Body;TSS150C | NA | -1.24E-02 | 1.66E-14 |
| cg18096962 | NA | NA | Island | 1.06E-02 | 1.63E-05 |
| cg18097850 | SELV | TSS200 | N_Shore | 6.75E-03 | 4.53E-06 |
| cg18099096 | NA | NA | NA | -1.76E-02 | 2.55E-09 |
| cg18105529 | DNAH2 | Body | N_Shore | -1.44E-02 | 3.87E-15 |
| cg18115040 | HOXD10 | TSS200 | Island | 2.11E-02 | 8.52E-09 |
| cg18121981 | NA | NA | S_Shelf | -1.90E-02 | 2.52E-08 |
| cg18122743 | NA | NA | N_Shelf | -5.61E-03 | 5.74E-05 |
| cg18125836 | LIMCH1;LIMCH1;LIN | TSS1500;TSS1500;T | N_Shore | -6.42E-03 | 2.63E-05 |
| cg18137414 | NA | NA | N_Shore | -1.44E-02 | 7.07E-09 |
| cg18141828 | OPRD1 | 1stExon | Island | 1.70E-02 | 5.31E-05 |
| cg18143296 | NA | NA | Island | 1.15E-02 | 1.91E-08 |
| cg18147543 | ZBTB17 | 5'UTR | NA | -1.11E-02 | 1.43E-05 |
| cg18147605 | IFFO1;IFFO1 | Body;Body | N_Shore | -1.39E-02 | 2.96E-14 |
| cg18151030 | PRDM15;PRDM15 | Body;Body | S_Shore | -1.44E-02 | 2.32E-10 |
| cg18159860 | NA | NA | Island | 9.01E-03 | 4.79E-07 |
| cg18170297 | NA | NA | N_Shelf | -8.85E-03 | 5.47E-08 |
| cg18177731 | IFFO2 | Body | NA | -5.35E-03 | 7.15E-06 |
| cg18183242 | KCNK17;KCNK17 | Body;Body | N_Shelf | -8.89E-03 | 2.18E-08 |
| cg18183701 | MYBPH | Body | NA | -8.48E-03 | 1.59E-06 |
| cg18184411 | ZYG11A | Body | Island | 8.95E-03 | 3.90E-05 |
| cg18186343 | MIR770;MEG3;MEG | TSS1500;Body;Body | NA | -9.73E-03 | 4.40E-06 |
| cg18193094 | GRIK2;GRIK2;GRIK2; | 1stExon;1stExon;5'U | Island | 9.28E-03 | 1.22E-05 |
| cg18194945 | SHOX2;SHOX2;SHOX | Body;Body;Body | N_Shore | 1.15E-02 | 8.33E-08 |
| cg18201351 | NRXN1;NRXN1;NRXI | Body;Body;TSS200 | S_Shore | 1.32E-02 | 3.18E-07 |
| cg18212762 | MAP4K2 | 3'UTR | NA | -1.40E-02 | 2.78E-16 |
| cg18219180 | ICOS | TSS1500 | NA | 1.36E-02 | 2.25E-08 |
| cg18219226 | GUSB | TSS1500 | S_Shore | -8.59E-03 | 5.44E-08 |
| cg18224262 | NA | NA | S_Shore | -9.68E-03 | 4.56E-06 |
| cg18229521 | SLC24A4;SLC24A4;S | 1stExon;5'UTR;1stE | Island | 2.81E-02 | 6.89E-12 |
| cg18234606 | THBS1 | Body | NA | -2.16E-02 | 3.46E-05 |
| cg18240400 | ANUBL1;ANUBL1 | TSS1500;TSS1500 | Island | 6.24E-03 | 7.27E-05 |
| cg18247055 | SPAG6;SPAG6 | TSS200;TSS200 | Island | 3.53E-02 | 5.01E-10 |
| cg18252039 | NA | NA | S_Shelf | 6.99E-03 | 3.98E-06 |
| cg18252633 | ARHGEF17 | Body | S_Shore | -8.34E-03 | 4.62E-05 |
| cg18257814 | CCDC88A;CCDC88A; | 5'UTR;1stExon;1stE | N_Shore | 1.45E-02 | 6.70E-06 |
| cg18267049 | NA | NA | Island | 2.69E-02 | 2.12E-07 |
| cg18267374 | NEFM;NEFM;NEFM | TSS1500;5'UTR;1stE | Island | 1.55E-02 | 8.68E-13 |
| cg18273840 | HCN1 | Body | Island | 2.49E-02 | 2.31E-10 |
| cg18279094 | FOXD3 | 1stExon | Island | 2.43E-02 | 1.61E-07 |
| cg18287310 | DAB1 | 5'UTR | NA | -7.30E-03 | 1.19E-08 |
| cg18294691 | NA | NA | Island | 2.16E-02 | 1.90E-08 |
| cg18294707 | RNASE2 | TSS1500 | NA | -1.41E-02 | 2.57E-19 |
| cg18297437 | ZIC1 | Body | Island | 1.62E-02 | 6.29E-07 |
| cg18299578 | FOXG1 | TSS1500 | N_Shore | 1.03E-02 | 3.41E-09 |
| cg18301891 | DIO3;MIR1247 | TSS1500;TSS1500 | Island | 3.03E-02 | 4.74E-05 |
| cg18304704 | MAST3 | Body | S_Shore | -1.01E-02 | 4.94E-08 |
| cg18310639 | CAPN2;CAPN2 | Body;Body | S_Shelf | -1.14E-02 | 4.59E-16 |

| | | | | | |
|---|---|---|---|---|---|
| cg18311537 | MIR196B | TSS200 | Island | 8.73E-03 | 1.91E-07 |
| cg18311708 | ZNF767;ZNF767 | TSS1500;TSS1500 | S_Shore | -1.35E-02 | 1.16E-05 |
| cg18313790 | PCDHB13 | 1stExon | Island | 7.94E-03 | 2.81E-05 |
| cg18322569 | BARHL2;BARHL2 | 5'UTR;1stExon | Island | 2.34E-02 | 5.59E-11 |
| cg18323236 | NIPAL3 | 5'UTR | Island | -2.58E-02 | 9.88E-08 |
| cg18324824 | GFRA1;GFRA1;GFRA | Body;Body;Body | N_Shore | -1.95E-02 | 2.21E-05 |
| cg18328334 | TNS1;TNS1 | 1stExon;5'UTR | NA | -9.92E-03 | 2.46E-06 |
| cg18333339 | NA | NA | NA | -1.60E-02 | 2.91E-24 |
| cg18335326 | KIAA1949;KIAA1949 | Body;1stExon | N_Shore | -1.73E-02 | 7.55E-12 |
| cg18335991 | SEMA7A;SEMA7A;SI | Body;Body;5'UTR | N_Shore | -8.26E-03 | 1.40E-06 |
| cg18343437 | NA | NA | Island | 1.89E-02 | 5.37E-08 |
| cg18345924 | NCAM2 | Body | S_Shore | -1.76E-02 | 1.69E-07 |
| cg18347442 | NA | NA | NA | -6.77E-03 | 3.62E-05 |
| cg18358723 | LOC645323 | Body | N_Shore | 1.45E-02 | 2.00E-10 |
| cg18363008 | TM4SF5 | TSS200 | NA | -5.06E-03 | 1.69E-06 |
| cg18389639 | PDLIM1 | Body | N_Shore | -9.09E-03 | 6.26E-08 |
| cg18395623 | SCRN1;SCRN1;SCRN | Body;Body;Body;Bo | NA | -1.26E-02 | 9.29E-11 |
| cg18403193 | C6orf176 | Body | N_Shelf | -9.45E-03 | 1.78E-05 |
| cg18404041 | ITIH1;ITIH1;ITIH1;ITI | Body;Body;Body;Bo | NA | -1.14E-02 | 2.00E-08 |
| cg18405341 | ATF4;ATF4 | 3'UTR;3'UTR | S_Shore | -7.58E-03 | 1.99E-08 |
| cg18405719 | NA | NA | NA | -3.13E-02 | 1.62E-25 |
| cg18409985 | ATP11A;ATP11A | Body;Body | NA | -1.34E-02 | 2.07E-07 |
| cg18418479 | MINK1;MINK1;MINK | TSS1500;TSS1500;T | N_Shore | -9.88E-03 | 4.55E-18 |
| cg18419358 | NA | NA | NA | -1.55E-02 | 3.60E-11 |
| cg18427465 | FBXO34;FBXO34 | TSS1500;TSS1500 | N_Shore | -2.83E-02 | 1.63E-08 |
| cg18431951 | PUF60;PUF60;PUF6( | Body;Body;5'UTR | NA | 1.04E-02 | 2.31E-09 |
| cg18437808 | C17orf62;C17orf62; | Body;Body;Body | Island | -1.36E-02 | 1.10E-10 |
| cg18439861 | PCDHGA4;PCDHGA1 | Body;Body;Body;Bo | Island | 2.03E-02 | 9.76E-06 |
| cg18444673 | NFKBID | 5'UTR | N_Shore | -5.65E-03 | 1.86E-07 |
| cg18448426 | APOL1;APOL1;APOL | TSS1500;TSS1500;T | NA | -2.32E-02 | 4.11E-26 |
| cg18450254 | PRICKLE2 | 5'UTR | NA | -1.45E-02 | 5.10E-13 |
| cg18458509 | SLC22A18AS;SLC22A | Body;TSS1500 | N_Shelf | -6.55E-03 | 5.44E-06 |
| cg18468354 | NA | NA | Island | 1.37E-02 | 3.27E-07 |
| cg18471993 | NA | NA | Island | 1.47E-02 | 3.31E-07 |
| cg18474153 | EEF2 | Body | N_Shore | -8.09E-03 | 6.41E-07 |
| cg18480675 | CMC1 | TSS1500 | N_Shore | -3.72E-02 | 1.19E-06 |
| cg18490846 | C17orf73 | TSS1500 | NA | -1.68E-02 | 3.00E-19 |
| cg18501647 | PRRT1 | Body | N_Shore | 9.27E-03 | 1.94E-06 |
| cg18502099 | NA | NA | N_Shore | -7.21E-03 | 4.25E-06 |
| cg18505959 | BAT5 | Body | N_Shore | -9.16E-03 | 2.58E-06 |
| cg18513357 | TRPC7;TRPC7;TRPC7 | Body;Body;Body | S_Shelf | -2.56E-02 | 4.82E-06 |
| cg18525126 | BMPR1A | 5'UTR | NA | -1.63E-02 | 1.17E-20 |
| cg18532215 | PLOD2;PLOD2 | TSS1500;TSS1500 | S_Shore | -1.53E-02 | 1.93E-05 |
| cg18538662 | AFAP1;AFAP1 | TSS200;TSS200 | Island | 2.89E-02 | 3.26E-06 |
| cg18540492 | AGBL4 | Body | Island | 1.45E-02 | 9.39E-07 |
| cg18541254 | NA | NA | N_Shore | 8.77E-03 | 7.29E-10 |
| cg18568067 | DGKZ;DGKZ;DGKZ | Body;Body;Body | S_Shelf | -8.72E-03 | 3.83E-10 |
| cg18568843 | TMEM14A | TSS1500 | N_Shore | -1.44E-02 | 9.44E-14 |
| cg18573383 | KCNC2;KCNC2;KCNC | 1stExon;1stExon;5'U | S_Shore | 1.26E-02 | 5.64E-09 |
| cg18582010 | ASAP1 | TSS1500 | NA | -1.11E-02 | 1.10E-06 |

| | | | | | |
|---|---|---|---|---|---|
| cg18582342 | ELAVL3;ELAVL3 | TSS200;TSS200 | Island | 7.24E-03 | 8.14E-07 |
| cg18596010 | NA | NA | Island | 7.13E-03 | 8.36E-10 |
| cg18612627 | NA | NA | Island | 1.49E-02 | 2.03E-07 |
| cg18618815 | COL1A1 | Body | N_Shore | -1.45E-02 | 2.59E-16 |
| cg18622896 | NA | NA | NA | -4.54E-02 | 1.24E-08 |
| cg18627235 | NA | NA | Island | 1.80E-02 | 4.46E-05 |
| cg18628483 | KIF5A | TSS1500 | N_Shore | -8.91E-03 | 3.00E-08 |
| cg18633600 | LRTM2;LRTM2;CACI | Body;Body;Body;Bo | NA | 7.53E-03 | 1.92E-07 |
| cg18635670 | HMGN4 | 5'UTR | S_Shore | -1.57E-02 | 1.76E-12 |
| cg18651026 | COL11A2;COL11A2;( | Body;Body;Body | NA | -7.55E-03 | 3.57E-10 |
| cg18656708 | NA | NA | S_Shore | 4.69E-03 | 3.37E-05 |
| cg18674643 | NA | NA | Island | 1.28E-02 | 3.35E-06 |
| cg18675610 | ARHGAP12 | 5'UTR | N_Shore | -1.47E-02 | 1.80E-06 |
| cg18679410 | LRRC3B;LRRC3B | 1stExon;5'UTR | Island | 8.10E-03 | 4.18E-05 |
| cg18685561 | 01-mar | TSS1500 | Island | 9.33E-03 | 7.21E-06 |
| cg18688704 | PDGFC | Body | N_Shelf | -1.66E-02 | 1.31E-12 |
| cg18689332 | TBX5;TBX5;TBX5;TB | Body;Body;Body;Bo | N_Shore | 7.39E-03 | 1.53E-06 |
| cg18691434 | STAG3;GPC2 | TSS200;TSS1500 | Island | 1.03E-02 | 7.46E-07 |
| cg18692205 | COBL | Body | NA | -1.49E-02 | 1.69E-10 |
| cg18707417 | PTH1R | Body | N_Shelf | -8.18E-03 | 1.83E-06 |
| cg18708252 | CBX7 | Body | N_Shelf | -9.96E-03 | 1.70E-07 |
| cg18708504 | IP6K3;IP6K3 | TSS1500;TSS1500 | NA | -6.51E-03 | 8.24E-05 |
| cg18719170 | OR6B2 | TSS200 | NA | -9.34E-03 | 2.39E-08 |
| cg18724565 | FSCN1 | 1stExon | Island | 2.32E-02 | 5.71E-06 |
| cg18735473 | BRUNOL4;BRUNOL4 | Body;Body;Body;Bo | NA | -8.42E-03 | 7.64E-05 |
| cg18738190 | CHST3 | 5'UTR | NA | -1.27E-02 | 3.82E-08 |
| cg18760496 | NA | NA | N_Shelf | -7.22E-03 | 2.82E-09 |
| cg18765906 | NID1 | Body | NA | -9.92E-03 | 1.09E-06 |
| cg18770350 | ACTN2;ACTN2 | 1stExon;5'UTR | Island | 3.60E-02 | 3.94E-10 |
| cg18774195 | SLC7A14;SLC7A14 | 5'UTR;1stExon | S_Shore | 8.30E-03 | 9.22E-07 |
| cg18779283 | LIMD1 | TSS200 | S_Shore | -9.15E-03 | 1.05E-07 |
| cg18780412 | PEX5L | TSS1500 | Island | 3.23E-02 | 1.72E-06 |
| cg18786171 | ADK;ADK | Body;TSS1500 | N_Shore | -1.43E-02 | 1.46E-16 |
| cg18791730 | HAO2;HAO2 | TSS1500;TSS1500 | NA | -1.22E-02 | 3.82E-13 |
| cg18795809 | ZNF518B | 5'UTR | Island | 2.75E-02 | 2.85E-09 |
| cg18797590 | CC2D2A;CC2D2A;CC | Body;Body;Body | NA | -1.79E-02 | 8.62E-16 |
| cg18801806 | CCDC84 | Body | S_Shore | -6.08E-03 | 1.48E-07 |
| cg18808904 | BTBD3;BTBD3;BTBD | 1stExon;5'UTR;5'UT | NA | -2.23E-02 | 3.78E-24 |
| cg18809126 | VGLL4;VGLL4;VGLL4 | Body;Body;Body | NA | -2.15E-02 | 5.76E-23 |
| cg18815647 | FAM123A;FAM123A | Body;1stExon | Island | 9.07E-03 | 4.86E-05 |
| cg18815943 | FOXE3 | 1stExon | Island | 3.20E-02 | 4.34E-10 |
| cg18822719 | SLC23A3;SLC23A3;S | TSS1500;TSS1500;T | NA | -1.19E-02 | 2.31E-10 |
| cg18835078 | PCDHGA1;PCDHGA1 | 1stExon;1stExon | Island | 2.80E-02 | 1.92E-07 |
| cg18836626 | ATP8A2 | Body | Island | 1.05E-02 | 1.78E-08 |
| cg18841653 | AKR1C2;AKR1C2;AK | 5'UTR;5'UTR;TSS20( | NA | -6.61E-03 | 7.50E-05 |
| cg18847118 | NA | NA | S_Shelf | -1.95E-02 | 4.81E-13 |
| cg18849102 | MSX1 | TSS1500 | Island | 6.50E-03 | 6.39E-07 |
| cg18862005 | NA | NA | NA | -1.07E-02 | 3.49E-11 |
| cg18866015 | DCC | Body | Island | 1.92E-02 | 5.04E-06 |
| cg18869485 | PCDHGA4;PCDHGA1 | Body;Body;Body;Bo | N_Shore | -1.62E-02 | 7.29E-05 |

| | | | | | |
|---|---|---|---|---|---|
| cg18873909 | PARG | Body | N_Shelf | -1.13E-02 | 1.13E-22 |
| cg18876270 | NA | NA | N_Shore | -9.93E-03 | 7.80E-09 |
| cg18877361 | NA | NA | NA | -1.87E-02 | 1.30E-13 |
| cg18879160 | ZNF844 | TSS200 | Island | 1.18E-02 | 1.49E-06 |
| cg18879828 | NA | NA | S_Shelf | -1.15E-02 | 2.40E-13 |
| cg18891762 | PCDHGA4;PCDHGA2 | Body;Body;1stExon, | S_Shore | 1.07E-02 | 4.34E-05 |
| cg18912541 | DCAF5 | Body | NA | -1.08E-02 | 1.61E-10 |
| cg18914962 | MAP3K5 | Body | NA | -1.12E-02 | 2.93E-13 |
| cg18915128 | UNC119;UNC119 | Body;Body | N_Shore | -4.49E-03 | 2.79E-05 |
| cg18920097 | GJD2 | Body | N_Shore | 9.38E-03 | 8.11E-06 |
| cg18928900 | NA | NA | Island | 8.13E-03 | 6.13E-08 |
| cg18929842 | NA | NA | N_Shore | -9.46E-03 | 3.08E-08 |
| cg18933331 | NA | NA | S_Shore | -8.76E-03 | 1.04E-12 |
| cg18935108 | NA | NA | Island | 1.76E-02 | 1.16E-05 |
| cg18935453 | LRP5 | Body | NA | -8.49E-03 | 8.57E-07 |
| cg18952506 | RIN2 | Body | NA | -1.55E-02 | 1.67E-19 |
| cg18952796 | NPTX2;NPTX2 | 5'UTR;1stExon | Island | 3.38E-02 | 5.44E-08 |
| cg18953784 | NA | NA | Island | 1.60E-02 | 3.78E-09 |
| cg18954388 | SLC6A5 | Body | N_Shore | 7.47E-03 | 4.24E-08 |
| cg18956933 | NA | NA | Island | 9.49E-03 | 6.20E-05 |
| cg18961681 | NA | NA | Island | 1.72E-02 | 8.08E-08 |
| cg18964582 | EDARADD | TSS1500 | N_Shore | -2.70E-02 | 2.53E-18 |
| cg18969008 | HPCAL1;HPCAL1 | 5'UTR;5'UTR | S_Shore | -1.55E-02 | 6.16E-09 |
| cg18969232 | NA | NA | Island | 6.80E-03 | 3.56E-05 |
| cg18978531 | NA | NA | NA | -1.05E-02 | 5.62E-11 |
| cg18987410 | HEPACAM | Body | Island | 2.83E-02 | 2.83E-06 |
| cg18993949 | CC2D2A;CC2D2A;CC | Body;Body;Body | NA | -1.01E-02 | 3.45E-15 |
| cg19008597 | BCAT1 | TSS200 | S_Shore | -9.48E-03 | 6.85E-06 |
| cg19019214 | NA | NA | Island | 1.26E-02 | 1.42E-08 |
| cg19021738 | NA | NA | Island | 6.43E-03 | 2.69E-08 |
| cg19025497 | SIL1;SIL1 | TSS1500;TSS1500 | S_Shore | -9.42E-03 | 1.02E-05 |
| cg19029181 | ZIC1 | Body | Island | 1.43E-02 | 3.15E-07 |
| cg19038027 | NA | NA | S_Shelf | -6.59E-03 | 4.82E-06 |
| cg19038230 | LRCH1;LRCH1;LRCH1 | TSS1500;TSS1500;T | N_Shore | -1.07E-02 | 4.61E-06 |
| cg19056004 | LRRC23;ENO2;LRRC2 | 3'UTR;TSS1500;3'UT | Island | -1.55E-02 | 3.95E-17 |
| cg19057899 | IGF2BP1;IGF2BP1 | Body;Body | S_Shore | -1.36E-02 | 1.73E-10 |
| cg19075717 | CACNA1G;CACNA1G | Body;Body;Body;Bo | NA | -7.62E-03 | 4.62E-05 |
| cg19077806 | PDE1A;PDE1A | Body;Body | NA | -2.29E-02 | 1.47E-12 |
| cg19078576 | BASP1;LOC285696;E | 5'UTR;TSS1500;1stE | Island | 2.37E-02 | 4.36E-05 |
| cg19079194 | ERBB4;ERBB4;ERBB4 | 5'UTR;5'UTR;1stExo | Island | 1.65E-02 | 1.76E-06 |
| cg19093370 | PLD6 | TSS1500 | Island | -3.63E-02 | 1.14E-14 |
| cg19099050 | LHFPL4 | 5'UTR | N_Shore | 2.36E-02 | 8.64E-12 |
| cg19100996 | FBRSL1 | Body | N_Shore | -5.49E-03 | 6.46E-06 |
| cg19103609 | PKN1 | TSS1500 | N_Shore | -9.84E-03 | 5.98E-08 |
| cg19117322 | ARHGAP23 | Body | S_Shore | -5.42E-03 | 1.43E-05 |
| cg19123345 | NA | NA | NA | -9.76E-03 | 1.21E-10 |
| cg19134347 | NA | NA | Island | 7.38E-03 | 1.13E-06 |
| cg19134568 | POU4F2 | Body | Island | 6.31E-03 | 3.35E-05 |
| cg19141644 | ZNF513 | Body | N_Shore | -6.63E-03 | 3.54E-08 |
| cg19149020 | MEIG1 | TSS1500 | N_Shore | -1.09E-02 | 2.27E-05 |

| | | | | | |
|---|---|---|---|---|---|
| cg19154116 | CYP21A2;CYP21A2 | TSS1500;TSS1500 | NA | -9.18E-03 | 1.17E-15 |
| cg19155932 | C20orf85 | TSS200 | Island | 1.74E-02 | 6.11E-06 |
| cg19165390 | NA | NA | Island | 7.13E-03 | 2.11E-08 |
| cg19181162 | NA | NA | Island | 6.90E-03 | 3.08E-06 |
| cg19190519 | NA | NA | S_Shelf | -1.36E-02 | 2.54E-13 |
| cg19203836 | STK19;STK19;DOM3 | Body;Body;TSS1500 | S_Shore | -1.17E-02 | 8.86E-08 |
| cg19211800 | MARCKS | 1stExon | N_Shore | 9.39E-03 | 8.30E-08 |
| cg19226099 | MC3R | 1stExon | N_Shore | -1.25E-02 | 3.70E-08 |
| cg19230755 | NA | NA | Island | 1.81E-02 | 8.18E-09 |
| cg19233663 | NA | NA | S_Shore | -8.15E-03 | 3.77E-08 |
| cg19234983 | NA | NA | N_Shore | -1.14E-02 | 1.22E-07 |
| cg19235307 | IFT122;IFT122;MBD4 | Body;Body;TSS1500 | S_Shore | -1.29E-02 | 8.56E-15 |
| cg19236454 | CIC | 3'UTR | NA | -7.40E-03 | 5.78E-06 |
| cg19237294 | BRUNOL4;BRUNOL4 | Body;Body;Body;Bo | Island | 1.63E-02 | 3.17E-06 |
| cg19261426 | OBSCN | Body | S_Shore | -1.11E-02 | 4.62E-09 |
| cg19268498 | CCDC39 | TSS1500 | Island | 1.27E-02 | 2.25E-06 |
| cg19269473 | NA | NA | Island | -6.85E-03 | 2.18E-07 |
| cg19283806 | CCDC102B | 5'UTR | NA | -3.09E-02 | 1.38E-17 |
| cg19284368 | NA | NA | NA | -9.60E-03 | 5.51E-08 |
| cg19293468 | SMG6;SMG6 | Body;Body | NA | -1.11E-02 | 6.59E-06 |
| cg19296520 | MAPKBP1;MAPKBP1 | 5'UTR;5'UTR | S_Shore | -1.22E-02 | 1.17E-08 |
| cg19306368 | NA | NA | NA | -1.96E-02 | 1.80E-12 |
| cg19320476 | FAM181B | 1stExon | Island | 1.47E-02 | 2.13E-05 |
| cg19329160 | NA | NA | Island | 1.33E-02 | 4.65E-05 |
| cg19332452 | RTBDN;RTBDN | Body;Body | Island | 5.21E-03 | 9.77E-10 |
| cg19334176 | DIP2C | Body | N_Shore | -8.95E-03 | 1.32E-06 |
| cg19335412 | ACTA2;ACTA2 | 3'UTR;3'UTR | NA | -1.42E-02 | 4.55E-14 |
| cg19337279 | MPL | Body | NA | -1.37E-02 | 6.76E-11 |
| cg19356189 | KLK10;KLK10 | TSS1500;TSS1500 | S_Shore | -1.13E-02 | 2.68E-07 |
| cg19356905 | GREM2;GREM2 | 1stExon;5'UTR | NA | 1.07E-02 | 1.01E-09 |
| cg19359550 | NA | NA | NA | -1.49E-02 | 3.93E-12 |
| cg19369022 | NA | NA | Island | 1.98E-02 | 1.52E-05 |
| cg19370054 | CNGA3;CNGA3 | 5'UTR;5'UTR | Island | 1.20E-02 | 1.09E-06 |
| cg19375123 | PLIN5 | TSS200 | S_Shore | 8.77E-03 | 1.33E-09 |
| cg19376664 | PBX2 | 3'UTR | NA | -1.40E-02 | 2.66E-08 |
| cg19384531 | TFAP2D | Body | Island | 1.32E-02 | 3.71E-07 |
| cg19389884 | NA | NA | Island | 1.42E-02 | 4.32E-09 |
| cg19389953 | NA | NA | N_Shore | 1.28E-02 | 1.61E-08 |
| cg19392831 | PRLHR | TSS1500 | Island | 6.54E-03 | 9.28E-11 |
| cg19395441 | C12orf43 | TSS200 | S_Shore | -1.11E-02 | 5.92E-06 |
| cg19400520 | NA | NA | S_Shore | -5.86E-03 | 3.73E-06 |
| cg19404207 | NA | NA | NA | -1.91E-02 | 1.20E-10 |
| cg19405504 | TMCC1;TMCC1 | 5'UTR;5'UTR | N_Shore | -1.20E-02 | 3.21E-06 |
| cg19413066 | NA | NA | NA | -6.43E-03 | 1.65E-06 |
| cg19414741 | PENK;PENK | Body;Body | Island | 8.59E-03 | 3.05E-05 |
| cg19416570 | ZNF274;ZNF274;ZNF | Body;Body;5'UTR | Island | 1.41E-02 | 5.66E-08 |
| cg19416590 | ADARB2 | Body | N_Shelf | -7.34E-03 | 1.19E-05 |
| cg19417526 | NTM;NTM;NTM;NTM | Body;Body;Body;Bo | NA | -1.68E-02 | 1.52E-10 |
| cg19419291 | ELL2 | Body | N_Shore | -1.64E-02 | 7.72E-06 |
| cg19421125 | LAG3 | Body | Island | -1.75E-02 | 9.39E-05 |

| | | | | | |
|---|---|---|---|---|---|
| cg19429281 | ZNF702P | Body | Island | 3.74E-02 | 3.17E-07 |
| cg19433091 | TMEM62 | Body | S_Shore | -1.39E-02 | 1.78E-11 |
| cg19442470 | CLU;CLU;CLU | TSS1500;Body;TSS1 | N_Shore | -8.84E-03 | 6.37E-08 |
| cg19442493 | HRNBP3 | 5'UTR | NA | -6.72E-03 | 1.51E-05 |
| cg19448816 | PLCL2;PLCL2 | Body;TSS1500 | NA | -1.54E-02 | 2.12E-09 |
| cg19451698 | RHBDD1;RHBDD1 | TSS1500;TSS1500 | Island | 1.31E-02 | 4.76E-07 |
| cg19458020 | RARA;RARA;RARA | TSS1500;5'UTR;TSS1 | Island | -1.79E-02 | 7.65E-05 |
| cg19458787 | NA | NA | NA | -1.28E-02 | 7.77E-12 |
| cg19459470 | NA | NA | NA | -7.94E-03 | 3.23E-06 |
| cg19460909 | NA | NA | NA | -1.05E-02 | 1.19E-08 |
| cg19464876 | NA | NA | S_Shelf | -1.05E-02 | 4.16E-10 |
| cg19464917 | ISL2 | TSS1500 | Island | 1.52E-02 | 1.26E-05 |
| cg19465320 | STAT5B | 5'UTR | N_Shore | -1.28E-02 | 1.78E-12 |
| cg19472098 | IQSEC3 | TSS1500 | Island | 1.27E-02 | 7.33E-07 |
| cg19478698 | MUC4;MUC4;MUC4 | Body;Body;Body | NA | -1.33E-02 | 1.42E-07 |
| cg19494100 | RNF144B | TSS1500 | N_Shore | -4.20E-03 | 5.69E-05 |
| cg19496479 | LYPD1;LYPD1 | Body;Body | NA | -1.38E-02 | 3.85E-11 |
| cg19497451 | KY | Body | NA | -6.30E-03 | 4.33E-05 |
| cg19499581 | NA | NA | Island | -6.45E-03 | 1.72E-06 |
| cg19506435 | PPP2R2C | Body | N_Shelf | -1.05E-02 | 1.40E-07 |
| cg19510626 | MAGI3;MAGI3 | Body;Body | S_Shore | -2.10E-02 | 2.69E-17 |
| cg19514721 | NA | NA | NA | -1.15E-02 | 1.81E-14 |
| cg19517476 | GIPC1;GIPC1;GIPC1; | Body;Body;Body;Bo | Island | -1.35E-02 | 3.81E-05 |
| cg19518666 | PDGFC | Body | NA | -1.94E-02 | 2.25E-08 |
| cg19519624 | NA | NA | Island | -1.53E-02 | 1.70E-07 |
| cg19527617 | PCDHGA4;PCDHGA7 | Body;1stExon;Body; | S_Shore | 1.53E-02 | 1.18E-08 |
| cg19529621 | NA | NA | NA | -1.30E-02 | 4.71E-06 |
| cg19542816 | HOXD1 | TSS200 | Island | 1.83E-02 | 1.16E-05 |
| cg19543968 | DNAL4 | TSS1500 | S_Shore | -1.26E-02 | 1.46E-05 |
| cg19544065 | MYT1L | 5'UTR | NA | -1.22E-02 | 4.89E-13 |
| cg19560758 | ERRFI1 | TSS1500 | Island | 1.08E-02 | 8.81E-13 |
| cg19573236 | NA | NA | Island | 3.20E-02 | 1.90E-05 |
| cg19578183 | NA | NA | S_Shore | -1.32E-02 | 1.63E-15 |
| cg19584235 | NA | NA | NA | -1.37E-02 | 3.25E-05 |
| cg19590421 | NA | NA | NA | -2.97E-02 | 5.04E-07 |
| cg19591056 | FMN2 | TSS200 | Island | 1.31E-02 | 1.83E-05 |
| cg19591595 | NA | NA | NA | 8.57E-03 | 1.79E-05 |
| cg19592472 | OXT;OXT | 1stExon;5'UTR | Island | -1.24E-02 | 2.65E-05 |
| cg19593401 | CCDC80;CCDC80 | TSS1500;TSS1500 | NA | -9.87E-03 | 6.14E-08 |
| cg19596509 | NA | NA | N_Shelf | -1.01E-02 | 5.11E-06 |
| cg19605623 | SCHIP1 | Body | NA | -1.32E-02 | 1.16E-06 |
| cg19609242 | OSMR;OSMR | TSS1500;TSS200 | Island | 1.08E-02 | 2.19E-05 |
| cg19612068 | NA | NA | S_Shore | -4.25E-03 | 4.89E-06 |
| cg19619405 | ADAMTS20 | TSS200 | Island | 2.11E-02 | 2.32E-09 |
| cg19621076 | HMGN2 | Body | S_Shore | -1.65E-02 | 1.44E-12 |
| cg19629818 | GPC2 | Body | S_Shore | -8.46E-03 | 5.78E-05 |
| cg19646445 | KCTD3 | TSS200 | Island | 3.66E-02 | 2.65E-05 |
| cg19653594 | SPTBN4;BLVRB | TSS1500;TSS1500 | Island | -1.95E-02 | 4.24E-09 |
| cg19663246 | NA | NA | S_Shore | -1.61E-02 | 5.02E-10 |
| cg19664185 | JAG2;JAG2 | Body;Body | N_Shelf | -1.27E-02 | 1.30E-05 |

| | | | | | |
|---|---|---|---|---|---|
| cg19670290 | HDDC3;UNC45A;UN | TSS1500;5'UTR;TSS1 | N_Shore | -1.51E-02 | 2.49E-16 |
| cg19671120 | CNGA3;CNGA3;CNG | 1stExon;1stExon;5'U | Island | 7.20E-03 | 5.69E-10 |
| cg19679112 | NA | NA | N_Shore | -9.38E-03 | 9.05E-12 |
| cg19681842 | UBR7;UBR7;C14orf1 | TSS1500;TSS1500;B | N_Shore | -1.54E-02 | 3.92E-05 |
| cg19683821 | NA | NA | S_Shore | 1.11E-02 | 1.88E-06 |
| cg19686152 | TMOD3 | TSS1500 | N_Shore | -9.85E-03 | 2.60E-08 |
| cg19699090 | NA | NA | S_Shore | -8.56E-03 | 2.58E-07 |
| cg19702785 | KCNS1 | Body | Island | 1.15E-02 | 6.24E-11 |
| cg19708554 | FAM78B | TSS1500 | S_Shore | -1.36E-02 | 7.76E-10 |
| cg19714279 | GBX2 | TSS1500 | Island | 1.30E-02 | 6.15E-06 |
| cg19719475 | UBE2E1;UBE2E1 | Body;Body | S_Shore | -1.77E-02 | 4.08E-20 |
| cg19722847 | IPO8 | TSS1500 | S_Shore | -1.17E-02 | 1.26E-08 |
| cg19724470 | CD274 | 5'UTR | S_Shore | -1.63E-02 | 6.06E-12 |
| cg19725343 | IGLON5 | Body | Island | 1.13E-02 | 8.99E-05 |
| cg19726711 | NA | NA | N_Shelf | -2.04E-02 | 2.02E-08 |
| cg19729744 | NA | NA | NA | -1.25E-02 | 7.06E-07 |
| cg19753867 | DSN1;DSN1;DSN1;D | Body;Body;Body;Bo | NA | -1.12E-02 | 2.14E-15 |
| cg19754282 | NA | NA | NA | -1.29E-02 | 7.81E-11 |
| cg19755459 | PLEKHA5;PLEKHA5 | Body;Body | S_Shore | -3.59E-02 | 2.11E-05 |
| cg19758448 | PGAP3 | 3'UTR | S_Shelf | -2.00E-02 | 1.41E-16 |
| cg19759064 | PHKG1;PHKG1 | 1stExon;5'UTR | NA | -1.04E-02 | 3.96E-10 |
| cg19766441 | SLC34A2 | TSS200 | Island | 2.01E-02 | 9.76E-07 |
| cg19770281 | NA | NA | NA | -1.65E-02 | 9.79E-06 |
| cg19786988 | TLN2 | Body | NA | -1.11E-02 | 2.80E-12 |
| cg19792599 | DDX25;PUS3;DDX25 | 5'UTR;TSS1500;1stE | Island | 2.30E-02 | 2.50E-05 |
| cg19793904 | TMEM132C | Body | NA | -1.34E-02 | 8.05E-10 |
| cg19803952 | DBNDD2;DBNDD2;D | 5'UTR;5'UTR;TSS150 | S_Shore | -7.24E-03 | 4.30E-06 |
| cg19808643 | SNX2 | TSS1500 | N_Shore | -1.60E-02 | 2.01E-06 |
| cg19809499 | FOXE3 | 1stExon | Island | 5.30E-02 | 1.80E-11 |
| cg19809667 | PRKCG | Body | Island | 6.49E-03 | 4.63E-06 |
| cg19811148 | NA | NA | NA | -1.61E-02 | 8.78E-15 |
| cg19819837 | NA | NA | Island | 1.00E-02 | 1.30E-05 |
| cg19821713 | NA | NA | Island | 1.25E-02 | 9.07E-17 |
| cg19822251 | LHFPL4;LHFPL4 | 5'UTR;1stExon | Island | 1.02E-02 | 6.61E-06 |
| cg19825437 | NA | NA | N_Shelf | -1.41E-02 | 1.09E-12 |
| cg19831403 | LDB3;LDB3;LDB3;LD | Body;Body;Body;Bo | NA | -5.97E-03 | 3.19E-05 |
| cg19832347 | C1QTNF9 | 5'UTR | NA | -3.34E-02 | 2.50E-06 |
| cg19833103 | LRCH1;LRCH1;LRCH1 | TSS1500;TSS1500;T | N_Shore | -1.03E-02 | 1.45E-07 |
| cg19835040 | RTP3 | TSS1500 | NA | -9.73E-03 | 1.96E-09 |
| cg19838043 | ZFYVE21 | Body | S_Shore | -1.21E-02 | 1.13E-12 |
| cg19845136 | NA | NA | N_Shore | -9.10E-03 | 1.52E-05 |
| cg19846389 | KRT9 | 3'UTR | NA | -1.08E-02 | 2.31E-08 |
| cg19847945 | MARCH10;MARCH1 | Body;Body | S_Shore | -1.47E-02 | 2.27E-16 |
| cg19852958 | NKX3-2 | 1stExon | Island | 2.05E-02 | 6.56E-08 |
| cg19856758 | NA | NA | S_Shore | -1.16E-02 | 1.21E-10 |
| cg19861117 | NOVA2 | Body | Island | 2.56E-02 | 8.78E-05 |
| cg19864060 | NA | NA | NA | -2.10E-02 | 3.22E-05 |
| cg19866866 | ZNF648 | Body | Island | 1.82E-02 | 5.91E-05 |
| cg19869443 | SAMD13;SAMD13 | TSS1500;Body | S_Shore | -1.22E-02 | 8.85E-09 |
| cg19873536 | NA | NA | NA | -1.62E-02 | 3.11E-09 |

| | | | | | |
|---|---|---|---|---|---|
| cg19885587 | NA | NA | NA | -6.61E-03 | 2.03E-07 |
| cg19885761 | CPLX2;CPLX2 | 5'UTR;1stExon | Island | 2.12E-02 | 4.84E-13 |
| cg19891728 | ANK1 | TSS1500 | Island | 9.98E-03 | 7.93E-08 |
| cg19908207 | GFRA3 | TSS1500 | S_Shore | -1.38E-02 | 5.11E-15 |
| cg19913291 | HCG18;TRIM39;TRIM | TSS1500;5'UTR;5'UT | S_Shore | -1.58E-02 | 6.03E-12 |
| cg19922137 | SYT14;SYT14;SYT14; | 1stExon;5'UTR;1stE: | Island | 2.21E-02 | 1.90E-05 |
| cg19925849 | CACNG7 | Body | Island | 1.52E-02 | 4.27E-06 |
| cg19926902 | SCN4B;SCN4B;SCN4 | Body;5'UTR;Body;B | N_Shore | -9.72E-03 | 7.85E-10 |
| cg19945554 | NA | NA | Island | 1.46E-02 | 1.13E-06 |
| cg19945840 | SDF4;SDF4;B3GALT6 | TSS1500;TSS1500;1 | Island | 6.68E-03 | 7.99E-07 |
| cg19956914 | SUMF2;SUMF2;SUM | Body;Body;Body;Bo | NA | -8.90E-03 | 3.10E-05 |
| cg19965221 | TJP1;TJP1;TJP1;TJP1 | 5'UTR;1stExon;1stE: | Island | 2.51E-02 | 1.32E-05 |
| cg19990744 | AIFM3;AIFM3;AIFM: | Body;Body;Body;Bo | N_Shelf | 1.62E-02 | 1.17E-06 |
| cg19998073 | ZC3H14;ZC3H14;ZC3 | 3'UTR;3'UTR;3'UTR; | NA | -1.33E-02 | 8.62E-14 |
| cg20008148 | NKX2-8 | TSS1500 | Island | 1.30E-02 | 3.53E-07 |
| cg20010135 | HSD3B7;HSD3B7;HS | 5'UTR;5'UTR;5'UTR | NA | -9.05E-03 | 1.14E-06 |
| cg20011134 | DDO;DDO | Body;Body | NA | -1.20E-02 | 1.99E-10 |
| cg20012601 | MAML3 | Body | NA | -1.73E-02 | 6.30E-19 |
| cg20018782 | NA | NA | S_Shelf | -8.14E-03 | 7.73E-05 |
| cg20023788 | NA | NA | NA | -9.43E-03 | 1.38E-07 |
| cg20024403 | METRNL | Body | Island | -7.92E-03 | 2.83E-06 |
| cg20038219 | NA | NA | N_Shelf | -1.35E-02 | 5.73E-07 |
| cg20038477 | CCDC48 | Body | NA | -6.72E-03 | 3.01E-06 |
| cg20052760 | NA | NA | NA | -1.60E-02 | 7.87E-17 |
| cg20066488 | PPP1R13B | TSS1500 | S_Shore | -7.40E-03 | 9.50E-07 |
| cg20067719 | SPATA20 | TSS1500 | N_Shore | -1.09E-02 | 2.93E-05 |
| cg20069688 | STK19;STK19;DOM3 | Body;Body;TSS1500 | S_Shore | -5.20E-03 | 3.43E-05 |
| cg20086517 | NA | NA | S_Shelf | -2.09E-02 | 2.46E-10 |
| cg20094837 | NBEA | TSS1500 | NA | -1.22E-02 | 3.74E-06 |
| cg20098887 | RAB32 | TSS1500 | Island | 1.12E-02 | 1.57E-06 |
| cg20102280 | HTR2A;HTR2A | 5'UTR;Body | NA | -2.50E-02 | 1.83E-10 |
| cg20107346 | NA | NA | NA | -2.35E-02 | 6.52E-25 |
| cg20107668 | MCF2L | Body | S_Shore | -7.81E-03 | 4.45E-07 |
| cg20110742 | NA | NA | S_Shore | -1.68E-02 | 1.59E-09 |
| cg20116935 | SEMA3B;SEMA3B | TSS1500;TSS1500 | NA | -6.19E-03 | 1.45E-07 |
| cg20119148 | PDE4C | 5'UTR | Island | 1.02E-02 | 8.10E-09 |
| cg20128928 | TACR3 | 1stExon | Island | 7.01E-03 | 1.66E-06 |
| cg20146541 | TRIM58 | 1stExon | Island | 3.48E-02 | 5.15E-06 |
| cg20153322 | PXN;PXN | TSS1500;TSS1500 | S_Shore | -3.80E-03 | 2.45E-05 |
| cg20156659 | LHCGR;LHCGR | 1stExon;5'UTR | Island | 1.72E-02 | 7.89E-12 |
| cg20164887 | TYMP;TYMP;TYMP;T | 5'UTR;5'UTR;5'UTR; | Island | 1.54E-02 | 1.53E-06 |
| cg20168849 | TPRG1 | Body | NA | -2.08E-02 | 5.81E-10 |
| cg20170989 | POM121L12 | TSS1500 | N_Shore | -1.07E-02 | 9.35E-07 |
| cg20176648 | AQP1 | TSS1500 | NA | -4.50E-03 | 2.83E-05 |
| cg20188739 | SNORD87;SNHG6 | Body;Body | N_Shelf | -1.62E-02 | 5.77E-13 |
| cg20189674 | LHX1;LHX1 | 1stExon;5'UTR | Island | 5.57E-03 | 3.03E-05 |
| cg20192747 | NA | NA | Island | 9.92E-03 | 1.06E-06 |
| cg20209308 | GSC2 | Body | Island | 1.16E-02 | 1.01E-06 |
| cg20219381 | RGS22 | Body | Island | 1.36E-02 | 1.72E-05 |
| cg20222376 | AKAP8L | TSS1500 | S_Shore | -1.64E-02 | 1.90E-15 |

| | | | | | |
|---|---|---|---|---|---|
| cg20224885 | KBTBD12 | Body | NA | -8.66E-03 | 1.19E-07 |
| cg20224984 | MAFB;MAFB | 3'UTR;1stExon | Island | 1.51E-02 | 2.91E-06 |
| cg20248093 | NA | NA | Island | 7.78E-03 | 1.65E-05 |
| cg20249566 | NWD1 | TSS200 | NA | -3.44E-02 | 8.70E-40 |
| cg20252022 | CSRP2 | 5'UTR | N_Shore | 1.24E-02 | 1.47E-09 |
| cg20253855 | CUX1;CUX1;CUX1 | Body;Body;Body | NA | 1.04E-02 | 3.59E-05 |
| cg20267101 | BOC;WDR52 | 3'UTR;3'UTR | NA | -1.57E-02 | 5.21E-16 |
| cg20271361 | CACNA1G;CACNA1G | Body;Body;Body;Bo | NA | -1.09E-02 | 2.25E-07 |
| cg20273670 | NA | NA | Island | 1.34E-02 | 9.56E-07 |
| cg20277905 | MIR124-3 | TSS200 | Island | 7.12E-03 | 3.42E-05 |
| cg20279283 | GATA4 | Body | Island | 6.35E-03 | 1.41E-06 |
| cg20286236 | MIR193A | TSS1500 | Island | 1.56E-02 | 1.15E-05 |
| cg20297976 | SCGB1C1;LOC65348 | TSS200;TSS200 | NA | -1.30E-02 | 6.35E-06 |
| cg20300246 | LHX3 | Body | Island | 8.83E-03 | 3.95E-08 |
| cg20301962 | FZD2 | 1stExon | Island | 5.32E-03 | 2.20E-05 |
| cg20303399 | ADRA1A;ADRA1A;AI | TSS200;TSS200;TSS: | Island | 9.63E-03 | 2.77E-09 |
| cg20307125 | NA | NA | NA | -8.76E-03 | 1.37E-06 |
| cg20319926 | C16orf13;C16orf13; | Body;Body;Body;Bo | Island | -1.09E-02 | 3.94E-10 |
| cg20322837 | APLNR;APLNR | TSS1500;TSS1500 | NA | -8.45E-03 | 4.45E-05 |
| cg20324858 | UBE2Q1 | Body | N_Shore | -1.31E-02 | 5.38E-11 |
| cg20329047 | BOP1;HSF1 | Body;TSS1500 | N_Shore | -8.60E-03 | 1.90E-07 |
| cg20329085 | ASXL3 | TSS200 | NA | 2.20E-02 | 2.42E-06 |
| cg20334313 | NA | NA | Island | 1.30E-02 | 3.92E-11 |
| cg20343063 | TNKS1BP1 | 5'UTR | N_Shore | -1.62E-02 | 6.59E-13 |
| cg20344040 | NA | NA | S_Shore | -1.13E-02 | 4.13E-07 |
| cg20346726 | C11orf70;C11orf70 | 1stExon;5'UTR | Island | 1.01E-02 | 1.08E-07 |
| cg20359994 | NA | NA | Island | 1.26E-02 | 5.76E-10 |
| cg20372690 | NA | NA | Island | 6.44E-03 | 1.00E-05 |
| cg20382695 | ATRNL1 | Body | Island | 7.10E-03 | 1.63E-05 |
| cg20392615 | MTSS1 | Body | NA | -1.21E-02 | 6.80E-12 |
| cg20396069 | CALCOCO2 | TSS1500 | N_Shore | -1.41E-02 | 1.34E-12 |
| cg20403938 | HOXC10 | TSS1500 | N_Shore | 1.25E-02 | 1.89E-07 |
| cg20407365 | SART3 | Body | N_Shore | -2.02E-02 | 1.98E-13 |
| cg20408074 | HLA-B | TSS1500 | S_Shore | -2.00E-02 | 5.18E-06 |
| cg20412217 | RGS12;RGS12;RGS1: | Body;Body;Body | S_Shelf | -9.78E-03 | 2.06E-05 |
| cg20426710 | ZBED4 | 5'UTR | N_Shore | -2.06E-02 | 1.60E-18 |
| cg20426994 | KLF14 | 1stExon | Island | 1.83E-02 | 4.58E-08 |
| cg20434926 | GIPR | Body | Island | 1.95E-02 | 7.87E-05 |
| cg20442599 | NA | NA | Island | 2.64E-02 | 5.40E-16 |
| cg20445053 | LASS6 | Body | NA | -1.76E-02 | 2.06E-06 |
| cg20449382 | NA | NA | NA | -1.16E-02 | 3.58E-12 |
| cg20453412 | C1orf127;C1orf127 | Body;Body | NA | 9.72E-03 | 9.10E-08 |
| cg20456243 | SPEG | Body | S_Shelf | -8.17E-03 | 7.87E-09 |
| cg20468415 | ANK1;ANK1;ANK1;A | Body;Body;Body;Bo | NA | -2.02E-02 | 4.10E-15 |
| cg20469744 | ENOX1;ENOX1 | Body;Body | NA | -1.50E-02 | 2.75E-15 |
| cg20485758 | HFE;HFE;HFE;HFE;HI | Body;Body;Body;Bo | NA | -1.50E-02 | 4.48E-12 |
| cg20491914 | KCNK3 | TSS1500 | N_Shore | 9.65E-03 | 1.21E-05 |
| cg20494635 | LOC100133545 | TSS1500 | N_Shore | -8.05E-03 | 9.37E-07 |
| cg20495365 | RGS12;RGS12;RGS1: | TSS1500;Body;Body | N_Shelf | -1.24E-02 | 1.12E-10 |
| cg20495738 | CACNA1C;CACNA1C | Body;Body;Body;Bo | N_Shore | -1.09E-02 | 5.21E-10 |

| | | | | | |
|---|---|---|---|---|---|
| cg20498637 | NA | NA | S_Shore | -9.49E-03 | 9.52E-08 |
| cg20499290 | NA | NA | NA | 1.45E-02 | 8.14E-13 |
| cg20515136 | IL12A | Body | S_Shore | -1.06E-02 | 2.88E-13 |
| cg20536364 | NA | NA | NA | -1.46E-02 | 6.43E-14 |
| cg20544216 | C10orf90 | Body | N_Shelf | -1.05E-02 | 3.71E-10 |
| cg20544651 | SCG3;SCG3 | TSS200;TSS200 | Island | 5.87E-03 | 1.13E-06 |
| cg20555682 | NA | NA | Island | 2.37E-02 | 2.39E-07 |
| cg20585038 | NA | NA | Island | 1.16E-02 | 3.46E-07 |
| cg20585530 | SIX6 | TSS200 | Island | 1.74E-02 | 1.12E-08 |
| cg20585869 | NEFM;NEFM | TSS200;1stExon | Island | 4.42E-02 | 8.82E-08 |
| cg20590797 | SGCA;SGCA | 3'UTR;3'UTR | NA | -7.97E-03 | 1.20E-06 |
| cg20591472 | SYPL2 | TSS200 | Island | 1.79E-02 | 5.80E-18 |
| cg20597382 | NA | NA | Island | 1.21E-02 | 5.64E-05 |
| cg20597403 | SDK1;SDK1 | Body;3'UTR | Island | 1.15E-02 | 1.43E-05 |
| cg20604286 | VRK2;VRK2;VRK2;VF | TSS1500;TSS1500;T | N_Shore | -1.23E-02 | 1.28E-05 |
| cg20617328 | HLA-DPA1 | Body | NA | -2.20E-02 | 1.02E-11 |
| cg20618433 | SPC24 | TSS1500 | S_Shore | -9.26E-03 | 7.65E-07 |
| cg20630887 | ACOT7;ACOT7;ACOT | Body;Body;Body;Bo | NA | -9.29E-03 | 4.57E-10 |
| cg20638525 | NA | NA | NA | -1.01E-02 | 1.55E-08 |
| cg20647467 | FEZF1;FEZF1 | TSS200;TSS200 | N_Shore | 7.18E-03 | 8.42E-06 |
| cg20649017 | HOXD10 | TSS1500 | Island | 1.35E-02 | 3.25E-06 |
| cg20659418 | NA | NA | N_Shelf | 1.43E-02 | 3.99E-08 |
| cg20664247 | PEX10;PEX10 | TSS1500;TSS1500 | N_Shore | -8.71E-03 | 1.17E-07 |
| cg20672981 | NA | NA | S_Shore | 8.00E-03 | 6.14E-06 |
| cg20699586 | NA | NA | Island | 2.66E-02 | 9.43E-09 |
| cg20701901 | MIR29C | TSS200 | NA | -1.26E-02 | 2.24E-08 |
| cg20701926 | NA | NA | N_Shelf | -1.02E-02 | 1.55E-05 |
| cg20702390 | NT5C1B;NT5C1B | Body;Body | Island | 2.12E-02 | 2.90E-05 |
| cg20711030 | NA | NA | N_Shelf | -5.56E-03 | 2.26E-05 |
| cg20711218 | NA | NA | NA | 9.00E-03 | 3.75E-06 |
| cg20716080 | CLN6 | TSS1500 | S_Shore | -8.22E-03 | 6.19E-07 |
| cg20724257 | HLA-DRA | 1stExon | NA | -1.57E-02 | 1.40E-09 |
| cg20731257 | NA | NA | NA | -1.16E-02 | 2.43E-07 |
| cg20734996 | NEURL4;NEURL4 | TSS1500;TSS1500 | S_Shore | -9.04E-03 | 7.24E-06 |
| cg20747266 | NMBR | 1stExon | Island | 1.79E-02 | 1.06E-08 |
| cg20747380 | HOXA2;HOXA2 | 1stExon;5'UTR | N_Shore | 7.23E-03 | 6.68E-06 |
| cg20755170 | POU4F3 | 3'UTR | Island | 2.33E-02 | 2.09E-07 |
| cg20773033 | PDGFB;PDGFB | Body;Body | N_Shelf | -1.11E-02 | 1.04E-07 |
| cg20778451 | ZNF454 | TSS200 | Island | 1.00E-02 | 8.45E-05 |
| cg20792436 | ASPSCR1 | Body | NA | -2.33E-02 | 7.73E-05 |
| cg20795701 | C6orf10 | Body | NA | -9.91E-03 | 1.87E-09 |
| cg20809087 | BRUNOL6;BRUNOL6 | 5'UTR;1stExon | Island | 2.51E-02 | 5.80E-18 |
| cg20809378 | NA | NA | Island | 2.61E-02 | 1.46E-06 |
| cg20810478 | TRIM58 | 1stExon | Island | 2.54E-02 | 6.85E-06 |
| cg20813374 | FKBP5;FKBP5;FKBP5 | 5'UTR;TSS1500;TSS: | S_Shore | -7.45E-03 | 4.27E-07 |
| cg20814718 | NA | NA | N_Shore | -1.05E-02 | 3.87E-07 |
| cg20816447 | CC2D2A;CC2D2A;CC | Body;Body;Body | NA | -2.30E-02 | 3.72E-08 |
| cg20820759 | NA | NA | S_Shore | -9.41E-03 | 2.93E-06 |
| cg20822990 | ATP13A2;ATP13A2;/ | TSS1500;TSS1500;T | S_Shore | -8.00E-03 | 5.87E-13 |
| cg20847733 | CHRM2;CHRM2;CHF | 1stExon;5'UTR;5'UT | Island | 1.06E-02 | 7.46E-06 |

| | | | | | |
|---|---|---|---|---|---|
| cg20874052 | C11orf60;C11orf60 | TSS1500;TSS1500 | NA | -1.31E-02 | 2.08E-05 |
| cg20879085 | TES | TSS200 | Island | 2.57E-02 | 2.00E-08 |
| cg20884984 | THY1 | 5'UTR | Island | 1.35E-02 | 5.16E-06 |
| cg20886049 | TSKU | TSS1500 | N_Shore | -8.70E-03 | 5.14E-06 |
| cg20902783 | NDRG2;NDRG2;NDR | TSS1500;TSS200;TS: | Island | -5.61E-03 | 4.12E-05 |
| cg20922821 | CRADD | TSS1500 | N_Shore | -1.49E-02 | 1.31E-06 |
| cg20942223 | EN2;EN2 | 1stExon;5'UTR | Island | 5.77E-03 | 1.95E-05 |
| cg20951444 | FNDC5 | TSS1500 | NA | -8.39E-03 | 2.84E-08 |
| cg20954460 | NA | NA | NA | -1.02E-02 | 1.44E-09 |
| cg20956815 | NA | NA | Island | 7.05E-03 | 7.78E-06 |
| cg20964856 | HNRNPUL1;AXL;AXL | TSS1500;3'UTR;3'U | N_Shore | -1.40E-02 | 1.57E-23 |
| cg20969424 | NA | NA | NA | -9.92E-03 | 2.02E-08 |
| cg20974196 | CFD | Body | N_Shore | -9.18E-03 | 9.26E-05 |
| cg20979799 | RFX6 | 1stExon | Island | 9.94E-03 | 4.93E-07 |
| cg20979921 | C1QTNF6;C1QTNF6 | Body;Body | NA | -2.56E-02 | 1.68E-08 |
| cg20988565 | ZFPM2 | Body | S_Shelf | -1.31E-02 | 2.38E-10 |
| cg20992114 | PITX2;PITX2;PITX2 | Body;Body;Body | Island | 6.85E-03 | 6.06E-08 |
| cg20992180 | FOLR1;FOLR1;FOLR1 | 5'UTR;TSS1500;5'U | NA | -5.90E-03 | 8.66E-05 |
| cg20998539 | ERN1 | TSS1500 | S_Shore | -7.19E-03 | 1.75E-07 |
| cg21002146 | TC2N;TC2N;TC2N | TSS1500;5'UTR;TSS: | S_Shore | -1.41E-02 | 1.02E-05 |
| cg21009965 | LHX8 | TSS1500 | N_Shelf | 1.21E-02 | 1.84E-05 |
| cg21011139 | CYB5R2 | TSS1500 | Island | 1.90E-02 | 8.27E-11 |
| cg21045464 | NA | NA | N_Shore | 1.40E-02 | 1.32E-07 |
| cg21057494 | CLEC3B | TSS1500 | NA | -9.97E-03 | 5.61E-10 |
| cg21064451 | LGALS1 | TSS200 | N_Shore | -7.97E-03 | 2.61E-08 |
| cg21069176 | THRB;THRB;THRB | 5'UTR;5'UTR;5'UTR | NA | -1.42E-02 | 3.52E-09 |
| cg21079946 | FAM3D | Body | NA | -8.62E-03 | 7.11E-09 |
| cg21082868 | NA | NA | NA | -1.41E-02 | 7.57E-09 |
| cg21084284 | SYNM;SYNM | TSS200;TSS200 | Island | 1.94E-02 | 1.46E-09 |
| cg21091227 | NA | NA | Island | 9.11E-03 | 1.90E-07 |
| cg21091547 | CDKN1A;CDKN1A | TSS1500;TSS1500 | N_Shore | -2.05E-02 | 2.99E-19 |
| cg21099332 | NA | NA | NA | -1.04E-02 | 1.61E-05 |
| cg21114334 | IFITM4P | TSS1500 | N_Shore | -1.27E-02 | 7.71E-07 |
| cg21116457 | SLC14A1;SLC14A1;S | TSS200;TSS200;TSS: | NA | -2.48E-02 | 2.19E-29 |
| cg21117668 | BOK;BOK | 5'UTR;1stExon | Island | 3.13E-02 | 1.43E-10 |
| cg21122474 | FXYD3;FXYD3;FXYD3 | TSS200;TSS200;TSS: | NA | -7.06E-03 | 3.84E-06 |
| cg21126506 | ESRRB | Body | N_Shelf | -1.19E-02 | 4.55E-14 |
| cg21130958 | CRISPLD1 | Body | S_Shore | -1.69E-02 | 6.52E-16 |
| cg21144009 | PLXNA2 | Body | NA | -1.17E-02 | 1.62E-08 |
| cg21150108 | NA | NA | NA | -8.91E-03 | 2.77E-10 |
| cg21161526 | NA | NA | Island | -1.05E-02 | 2.28E-09 |
| cg21165221 | NA | NA | Island | 8.34E-03 | 6.57E-07 |
| cg21166964 | NA | NA | Island | 7.85E-03 | 8.22E-10 |
| cg21171320 | TRPM3;TRPM3;TRPI | Body;Body;Body;Bo | NA | -1.08E-02 | 3.62E-12 |
| cg21179088 | VSTM2A | Body | Island | 1.40E-02 | 4.85E-08 |
| cg21186955 | TRIM56 | 5'UTR | N_Shelf | -1.64E-02 | 7.47E-15 |
| cg21195256 | C14orf23;C14orf23 | Body;Body | S_Shore | 2.24E-02 | 9.10E-08 |
| cg21198219 | NA | NA | N_Shore | -1.66E-02 | 5.76E-09 |
| cg21200656 | NKX2-4 | TSS200 | Island | 1.64E-02 | 1.27E-07 |
| cg21210376 | PBK | TSS1500 | S_Shore | -1.45E-02 | 2.01E-06 |

| | | | | | |
|---|---|---|---|---|---|
| cg21211213 | EBF1 | Body | NA | -1.25E-02 | 5.19E-11 |
| cg21212995 | SLC6A7 | Body | S_Shore | 5.72E-03 | 8.09E-05 |
| cg21214293 | NA | NA | Island | 9.81E-03 | 3.23E-10 |
| cg21217540 | NA | NA | Island | -1.58E-02 | 2.79E-10 |
| cg21220374 | TSTD2;NCBP1 | TSS1500;Body | S_Shore | -1.19E-02 | 3.78E-06 |
| cg21229153 | NA | NA | NA | -5.93E-03 | 2.99E-05 |
| cg21234506 | BCL2A1;BCL2A1 | 1stExon;1stExon | NA | -2.17E-02 | 4.23E-10 |
| cg21236182 | NA | NA | NA | -1.10E-02 | 1.23E-11 |
| cg21239798 | FLJ11235;EPB41L4A | Body;TSS1500 | S_Shore | -8.57E-03 | 3.24E-05 |
| cg21247722 | NA | NA | NA | -1.67E-02 | 5.97E-07 |
| cg21249640 | NA | NA | NA | -9.78E-03 | 1.61E-10 |
| cg21282549 | NA | NA | Island | 2.08E-02 | 1.57E-07 |
| cg21293242 | TDH | Body | N_Shore | 9.02E-03 | 1.76E-08 |
| cg21296230 | GREM1 | 5'UTR | Island | 1.79E-02 | 5.39E-21 |
| cg21298174 | ZNF709 | TSS200 | S_Shore | 1.15E-02 | 1.96E-06 |
| cg21298444 | CBLN2 | 3'UTR | N_Shelf | -1.00E-02 | 9.69E-07 |
| cg21318213 | NR2F2;NR2F2;NR2F2 | TSS1500;1stExon;Bo | Island | 9.32E-03 | 2.24E-05 |
| cg21322248 | PSTPIP1 | Body | NA | -8.99E-03 | 7.05E-06 |
| cg21323720 | NA | NA | NA | -3.54E-02 | 1.80E-12 |
| cg21325715 | NA | NA | NA | -5.73E-03 | 7.64E-05 |
| cg21340845 | LRP1B | TSS1500 | S_Shelf | -1.49E-02 | 3.92E-10 |
| cg21353911 | NA | NA | Island | 1.11E-02 | 1.65E-05 |
| cg21355828 | NA | NA | Island | 2.13E-02 | 4.21E-09 |
| cg21370074 | PDE1B | TSS1500 | NA | -7.26E-03 | 3.52E-08 |
| cg21381594 | NA | NA | NA | 8.32E-03 | 1.02E-05 |
| cg21406967 | TRIP6;SLC12A9 | TSS1500;3'UTR | S_Shore | -9.38E-03 | 6.81E-10 |
| cg21443659 | NA | NA | NA | -6.96E-03 | 6.42E-08 |
| cg21448423 | ACOT11;ACOT11;AC | 1stExon;5'UTR;1stE | NA | -1.59E-02 | 2.11E-09 |
| cg21448513 | PLAG1;PLAG1;PLAG | 5'UTR;5'UTR;5'UTR | NA | -8.08E-03 | 1.01E-08 |
| cg21450829 | MCF2L2;B3GNT5 | Body;TSS1500 | N_Shore | -1.19E-02 | 9.54E-07 |
| cg21469505 | NA | NA | N_Shore | -2.78E-02 | 5.83E-35 |
| cg21473814 | CRTC1;CRTC1 | Body;Body | Island | -8.83E-03 | 1.16E-06 |
| cg21481937 | PTPRR;PTPRR | Body;Body | NA | -2.13E-02 | 5.27E-08 |
| cg21483092 | RGS12;RGS12;RGS12 | TSS1500;Body;Body | N_Shelf | -9.48E-03 | 2.35E-11 |
| cg21484228 | TRIM71 | TSS200 | Island | 1.81E-02 | 1.78E-09 |
| cg21488279 | PRDM6 | Body | Island | 9.05E-03 | 3.06E-06 |
| cg21493505 | ZNF542;ZNF542;ZNF | TSS1500;TSS1500;T | Island | 5.14E-03 | 6.87E-08 |
| cg21496658 | MME;MME;MME;M | TSS1500;TSS200;TS | Island | 1.60E-02 | 9.93E-06 |
| cg21498471 | NUDT19 | Body | S_Shore | -1.03E-02 | 8.79E-07 |
| cg21505886 | TMEM129;TACC3;TI | TSS1500;5'UTR;TSS | S_Shore | -6.00E-03 | 6.43E-08 |
| cg21508639 | NA | NA | N_Shelf | -1.11E-02 | 2.88E-09 |
| cg21512644 | NPY2R;NPY2R | 5'UTR;1stExon | Island | 1.44E-02 | 1.26E-05 |
| cg21517792 | MTA1 | TSS1500 | S_Shelf | -1.03E-02 | 1.39E-06 |
| cg21523251 | GPR78 | TSS200 | Island | 1.42E-02 | 6.10E-13 |
| cg21524899 | NA | NA | N_Shore | -7.71E-03 | 5.35E-07 |
| cg21530890 | SOX8 | 1stExon | Island | 9.48E-03 | 2.63E-05 |
| cg21547095 | KIFC1 | Body | S_Shore | -9.51E-03 | 1.03E-09 |
| cg21550219 | TGFBI | Body | NA | -8.70E-03 | 1.92E-06 |
| cg21553182 | ZNF578 | TSS200 | Island | 1.28E-02 | 2.54E-09 |
| cg21553596 | FAM3B;FAM3B | TSS200;TSS200 | N_Shore | 1.64E-02 | 1.52E-07 |

| | | | | | |
|---|---|---|---|---|---|
| cg21562740 | NA | NA | N_Shelf | -1.08E-02 | 1.45E-08 |
| cg21564242 | PEX5L | TSS1500 | Island | 6.92E-03 | 1.22E-06 |
| cg21565130 | FEZ1;FEZ1 | 5'UTR;5'UTR | N_Shore | 2.44E-02 | 5.95E-06 |
| cg21567504 | OCA2 | TSS200 | Island | 9.94E-03 | 7.60E-07 |
| cg21571060 | NA | NA | NA | -3.62E-02 | 3.40E-27 |
| cg21572722 | ELOVL2 | TSS1500 | Island | 1.40E-02 | 1.38E-32 |
| cg21578351 | NA | NA | NA | -7.55E-03 | 2.35E-05 |
| cg21584691 | NA | NA | NA | -2.11E-02 | 5.82E-08 |
| cg21584710 | DNAI2 | Body | NA | -1.37E-02 | 9.47E-18 |
| cg21585512 | LOC399959 | Body | NA | -2.76E-02 | 4.51E-08 |
| cg21585707 | FBXL18 | Body | S_Shore | -3.76E-02 | 3.15E-14 |
| cg21592158 | FDXACB1;C11orf1 | TSS1500;Body | S_Shore | -1.29E-02 | 2.65E-10 |
| cg21593015 | STOX2 | Body | NA | -2.65E-02 | 2.34E-25 |
| cg21602160 | AOC3;AOC3 | 1stExon;5'UTR | NA | -1.22E-02 | 8.41E-12 |
| cg21608225 | PDE4DIP;PDE4DIP;P | Body;Body;Body | N_Shore | -1.37E-02 | 1.48E-06 |
| cg21621538 | MPP6 | 5'UTR | NA | -9.59E-03 | 1.41E-08 |
| cg21623566 | DCC | Body | Island | 9.98E-03 | 1.35E-07 |
| cg21629394 | SPATA24 | Body | N_Shore | -1.04E-02 | 2.74E-07 |
| cg21629500 | C5orf38 | Body | Island | 2.76E-02 | 1.72E-14 |
| cg21632975 | NOVA2 | Body | Island | 8.70E-03 | 1.40E-11 |
| cg21639287 | COBLL1 | TSS1500 | S_Shore | -1.40E-02 | 1.65E-07 |
| cg21640603 | DOCK6 | 3'UTR | S_Shore | -1.10E-02 | 1.07E-11 |
| cg21640654 | USP40 | Body | S_Shelf | -9.66E-03 | 6.59E-09 |
| cg21657087 | NA | NA | Island | 1.04E-02 | 3.47E-09 |
| cg21657955 | TMEM196 | TSS200 | Island | 1.17E-02 | 5.54E-05 |
| cg21674927 | IL1R2;IL1R2 | Body;Body | NA | -1.28E-02 | 9.04E-11 |
| cg21692159 | PCGF2 | 5'UTR | NA | -1.19E-02 | 3.89E-09 |
| cg21699044 | NA | NA | N_Shore | 1.14E-02 | 7.19E-05 |
| cg21708058 | TACC1 | TSS200 | NA | -1.99E-02 | 5.22E-06 |
| cg21709871 | NRBP2 | 5'UTR | Island | 1.43E-02 | 2.82E-07 |
| cg21721340 | SOX18 | TSS1500 | S_Shore | -7.51E-03 | 9.26E-05 |
| cg21725716 | NA | NA | Island | 1.32E-02 | 8.32E-14 |
| cg21727532 | CLVS2 | TSS1500 | Island | 1.04E-02 | 1.88E-06 |
| cg21733531 | NA | NA | Island | 1.24E-02 | 7.68E-05 |
| cg21746711 | BAP1 | 3'UTR | NA | -6.76E-03 | 8.80E-05 |
| cg21756476 | RORA | Body | NA | -1.39E-02 | 1.92E-13 |
| cg21756806 | KIAA0652;KIAA0652 | TSS1500;TSS1500;5 | N_Shore | -1.73E-02 | 1.51E-10 |
| cg21757368 | C6orf170 | TSS1500 | S_Shore | -8.27E-03 | 9.04E-05 |
| cg21758773 | HLA-E | Body | S_Shore | -1.46E-02 | 1.83E-10 |
| cg21759685 | CCR2;CCR2 | 3'UTR;Body | NA | -1.79E-02 | 2.23E-20 |
| cg21762820 | FAM78B | Body | Island | 7.35E-03 | 3.63E-06 |
| cg21791252 | NA | NA | NA | -1.01E-02 | 6.85E-09 |
| cg21796547 | PAPLN | 5'UTR | N_Shore | -1.18E-02 | 1.72E-07 |
| cg21800232 | ANKRD34B | TSS200 | Island | 2.06E-02 | 2.02E-10 |
| cg21801378 | BRUNOL6 | 1stExon | Island | 2.67E-02 | 2.00E-13 |
| cg21834204 | MTERFD3;MTERFD3 | TSS1500;TSS1500 | S_Shore | -1.21E-02 | 2.11E-07 |
| cg21836275 | NT5DC3 | TSS1500 | Island | 2.61E-02 | 1.43E-06 |
| cg21840211 | USP36 | Body | S_Shelf | -1.18E-02 | 1.50E-06 |
| cg21840875 | SMCR7L | 3'UTR | NA | -7.21E-03 | 6.02E-09 |
| cg21841963 | NA | NA | Island | 1.04E-02 | 1.11E-06 |

| | | | | | |
|---|---|---|---|---|---|
| cg21858380 | C1QL2 | TSS200 | Island | 1.36E-02 | 6.32E-05 |
| cg21858516 | DACT1;DACT1 | TSS1500;TSS1500 | N_Shore | -1.61E-02 | 8.20E-17 |
| cg21860560 | F10 | Body | Island | 9.31E-03 | 5.16E-08 |
| cg21874213 | NRG2;NRG2;NRG2;N | Body;Body;Body;Bo | S_Shore | -7.88E-03 | 5.13E-08 |
| cg21875330 | PAX9 | 5'UTR | Island | 1.02E-02 | 6.29E-08 |
| cg21878650 | ADAMTS6 | Body | NA | -1.98E-02 | 6.42E-07 |
| cg21882300 | NA | NA | N_Shore | 1.11E-02 | 1.87E-06 |
| cg21884231 | SLC7A14;SLC7A14 | 5'UTR;1stExon | Island | 2.23E-02 | 1.37E-05 |
| cg21886186 | NA | NA | N_Shelf | -9.96E-03 | 4.41E-12 |
| cg21887097 | LAMB1 | Body | NA | -1.80E-02 | 1.05E-05 |
| cg21895593 | UBN2 | Body | S_Shore | -2.36E-02 | 1.64E-10 |
| cg21911021 | ZIK1 | TSS1500 | Island | 1.66E-02 | 4.22E-16 |
| cg21915074 | POM121 | 5'UTR | S_Shore | -1.37E-02 | 7.23E-27 |
| cg21917976 | CHD1L | TSS1500 | N_Shore | -1.08E-02 | 7.43E-06 |
| cg21918313 | FBXO41 | Body | Island | 1.79E-02 | 1.34E-09 |
| cg21922810 | NA | NA | N_Shelf | -1.31E-02 | 7.53E-07 |
| cg21927426 | NA | NA | NA | -7.54E-03 | 8.04E-06 |
| cg21927946 | MIR7-3;C19orf30 | TSS1500;Body | NA | 1.56E-02 | 4.60E-12 |
| cg21930668 | PADI3 | Body | NA | -9.37E-03 | 8.24E-08 |
| cg21934230 | WNT3A | Body | NA | -5.73E-03 | 9.34E-05 |
| cg21946195 | ATOH8 | Body | NA | -5.43E-03 | 1.83E-05 |
| cg21948716 | NA | NA | Island | 4.81E-03 | 2.43E-06 |
| cg21962791 | PYROXD1 | TSS1500 | N_Shore | -1.70E-02 | 4.07E-08 |
| cg21990700 | LOC283314;C1RL | TSS200;Body | NA | -1.04E-02 | 1.41E-08 |
| cg21995800 | WDR77 | Body | NA | -1.07E-02 | 2.83E-09 |
| cg22006825 | HNRNPUL1;HNRNPL | Body;5'UTR | S_Shore | -4.76E-03 | 5.27E-05 |
| cg22007809 | TRAK2 | Body | NA | -1.88E-02 | 1.89E-19 |
| cg22016779 | DNER | Body | NA | -1.55E-02 | 2.09E-11 |
| cg22032900 | C13orf35;C13orf35 | 1stExon;5'UTR | N_Shore | -1.08E-02 | 8.58E-09 |
| cg22032961 | NA | NA | NA | -1.36E-02 | 1.23E-15 |
| cg22043168 | BDNF;BDNF;BDNF;B | Body;5'UTR;5'UTR;1 | Island | 1.29E-02 | 1.23E-05 |
| cg22052056 | DNMT3B;DNMT3B;[ | 5'UTR;5'UTR;5'UTR | S_Shore | -6.24E-03 | 4.65E-06 |
| cg22057680 | NA | NA | N_Shelf | -4.91E-03 | 5.39E-05 |
| cg22059073 | CECR6;CECR6 | TSS1500;TSS1500 | Island | 5.67E-03 | 7.98E-06 |
| cg22059812 | HTR6 | 1stExon | Island | 9.77E-03 | 1.19E-06 |
| cg22062984 | KCNJ6;KCNJ6 | 1stExon;5'UTR | Island | 9.20E-03 | 1.16E-05 |
| cg22064451 | TUBA1A | TSS1500 | S_Shore | -3.32E-02 | 5.44E-05 |
| cg22065526 | OBSL1;INHA | TSS1500;TSS200 | S_Shore | -9.33E-03 | 2.58E-07 |
| cg22065923 | NA | NA | Island | 8.50E-03 | 8.09E-05 |
| cg22070406 | ST8SIA3 | TSS200 | Island | 8.49E-03 | 1.57E-06 |
| cg22075924 | AK5;AK5 | Body;Body | NA | -1.04E-02 | 2.27E-14 |
| cg22080436 | NA | NA | S_Shelf | -8.78E-03 | 9.56E-07 |
| cg22085702 | CALCA;CALCA;CALC/ | TSS1500;TSS1500;T | N_Shore | 5.95E-03 | 5.56E-05 |
| cg22087390 | NA | NA | NA | 1.77E-02 | 1.39E-05 |
| cg22094551 | NA | NA | N_Shelf | -6.10E-03 | 8.83E-09 |
| cg22111038 | RAB3C | Body | Island | 9.24E-03 | 5.08E-06 |
| cg22156456 | EIF1 | TSS1500 | N_Shore | -1.22E-02 | 1.06E-08 |
| cg22158769 | LOC375196;LOC100 | TSS200;Body | Island | 6.10E-02 | 1.59E-16 |
| cg22161476 | FSTL5;FSTL5;FSTL5;F | 1stExon;1stExon;5'U | NA | 1.02E-02 | 3.79E-05 |
| cg22161562 | NA | NA | S_Shelf | -2.84E-02 | 1.69E-08 |

| | | | | | |
|---|---|---|---|---|---|
| cg22165106 | ELFN1 | 5'UTR | N_Shore | -9.90E-03 | 6.71E-06 |
| cg22171539 | NA | NA | N_Shelf | 8.81E-03 | 4.01E-05 |
| cg22178238 | PTPRN2;PTPRN2;PTI | Body;Body;Body | Island | 1.92E-02 | 3.12E-10 |
| cg22183706 | CALCA;CALCA;CALC/ | 1stExon;5'UTR;1stE: | N_Shore | 9.76E-03 | 1.91E-06 |
| cg22202689 | NA | NA | N_Shore | -1.49E-02 | 1.21E-14 |
| cg22203237 | NA | NA | NA | -1.18E-02 | 4.25E-08 |
| cg22209918 | NA | NA | N_Shore | -1.33E-02 | 1.74E-10 |
| cg22212691 | OBSL1 | Body | Island | 1.63E-02 | 9.41E-05 |
| cg22215942 | NA | NA | S_Shore | -6.96E-03 | 3.53E-07 |
| cg22221870 | FAM3B;FAM3B;FAM | 1stExon;5'UTR;1stE: | N_Shore | 3.68E-02 | 1.79E-07 |
| cg22234080 | NEFH | 1stExon | Island | 8.55E-03 | 6.91E-08 |
| cg22259765 | SNRPN;SNRPN;SNRF | 5'UTR;5'UTR;5'UTR; | N_Shelf | -1.37E-02 | 2.49E-05 |
| cg22260508 | PTPN6;PTPN6;PTPN | 3'UTR;3'UTR;3'UTR | NA | -9.05E-03 | 1.44E-07 |
| cg22264930 | TEKT3 | 5'UTR | N_Shore | 2.18E-02 | 2.86E-08 |
| cg22268279 | FLJ12825 | Body | N_Shelf | -1.09E-02 | 2.58E-10 |
| cg22273555 | NA | NA | S_Shore | -7.31E-03 | 1.82E-11 |
| cg22282405 | TFAP2B | Body | Island | 2.11E-02 | 2.06E-10 |
| cg22282410 | PTPRN2;PTPRN2;PTI | TSS1500;TSS1500;T | Island | 1.76E-02 | 4.10E-11 |
| cg22285878 | KLF14 | TSS1500 | Island | 3.13E-02 | 2.06E-13 |
| cg22294181 | SLC28A1;SLC28A1 | Body;Body | NA | -6.35E-03 | 3.61E-06 |
| cg22298860 | HLA-F;HLA-F;HLA-F | TSS1500;TSS1500;T | N_Shore | -1.14E-02 | 1.92E-06 |
| cg22310279 | EDNRB;EDNRB;EDNI | TSS1500;5'UTR;TSS: | Island | 1.16E-02 | 9.01E-05 |
| cg22331294 | LIMD1 | Body | NA | -7.73E-03 | 2.40E-05 |
| cg22350070 | CTNNA2;CTNNA2 | TSS1500;TSS1500 | N_Shore | 1.05E-02 | 5.29E-06 |
| cg22353329 | CBX4 | TSS1500 | Island | 1.26E-02 | 2.91E-15 |
| cg22356339 | ASCL1 | 1stExon | Island | 1.50E-02 | 6.21E-06 |
| cg22360649 | CCDC85B | TSS200 | N_Shore | -7.97E-03 | 7.85E-05 |
| cg22363368 | NA | NA | NA | -1.71E-02 | 6.91E-18 |
| cg22367191 | NA | NA | N_Shore | 1.56E-02 | 4.43E-07 |
| cg22369787 | NA | NA | S_Shore | 6.98E-03 | 6.68E-05 |
| cg22396555 | NA | NA | Island | 1.26E-02 | 2.64E-08 |
| cg22402467 | C1S;C1S;C1S;C1S | 1stExon;1stExon;5'l | NA | -1.74E-02 | 4.99E-12 |
| cg22407942 | SNORD93 | TSS1500 | S_Shore | -1.29E-02 | 1.14E-07 |
| cg22416596 | NA | NA | S_Shore | -7.70E-03 | 8.02E-05 |
| cg22419039 | ESRRG;ESRRG;ESRR( | TSS1500;TSS1500;5 | Island | 7.73E-03 | 5.03E-05 |
| cg22441157 | IGSF9;IGSF9 | 3'UTR;3'UTR | S_Shore | -7.68E-03 | 2.21E-08 |
| cg22451356 | KCNAB2;KCNAB2 | 3'UTR;3'UTR | NA | -4.52E-03 | 1.11E-05 |
| cg22454769 | FHL2;FHL2;FHL2;FHI | TSS200;TSS200;5'U' | Island | 1.81E-02 | 3.10E-12 |
| cg22483030 | HLA-DPB1 | 1stExon | NA | -1.93E-02 | 1.67E-10 |
| cg22491141 | NA | NA | Island | 1.12E-02 | 6.90E-05 |
| cg22500518 | TMEM184A | 3'UTR | N_Shore | -8.60E-03 | 2.76E-07 |
| cg22517995 | ZNF382;ZNF529;ZNI | 5'UTR;TSS1500;TSS: | Island | 1.02E-02 | 2.38E-06 |
| cg22530519 | AOC2;AOC3;AOC2 | Body;TSS1500;Body | NA | -1.04E-02 | 2.55E-11 |
| cg22537597 | SSPO | 3'UTR | NA | -6.38E-03 | 1.38E-06 |
| cg22541254 | GRIK2;GRIK2;GRIK2 | TSS200;TSS200;TSS: | Island | 1.01E-02 | 4.64E-05 |
| cg22545206 | NA | NA | Island | 2.32E-02 | 1.08E-06 |
| cg22551065 | NA | NA | S_Shore | -1.35E-02 | 1.86E-11 |
| cg22557662 | PPP1R14A | TSS1500 | Island | 2.76E-02 | 2.29E-09 |
| cg22560214 | NA | NA | Island | 8.37E-03 | 1.38E-08 |
| cg22565914 | NA | NA | NA | -1.01E-02 | 3.84E-11 |

| | | | | | |
|---|---|---|---|---|---|
| cg22575379 | NUDT16P;NUDT16P | TSS1500;TSS1500 | N_Shore | -6.84E-03 | 5.19E-07 |
| cg22580512 | NCOR2;NCOR2 | 5'UTR;5'UTR | N_Shore | -1.85E-02 | 1.25E-18 |
| cg22584802 | AHCYL2;AHCYL2;AH | Body;TSS200;Body; | NA | -1.09E-02 | 1.93E-06 |
| cg22586221 | NA | NA | NA | -1.96E-02 | 2.11E-35 |
| cg22606205 | PKP1;PKP1;PKP1;PK | 5'UTR;5'UTR;1stExo | Island | 2.12E-02 | 1.93E-05 |
| cg22607339 | MPL | TSS1500 | NA | -6.58E-03 | 3.61E-05 |
| cg22609474 | NA | NA | NA | -1.14E-02 | 2.51E-08 |
| cg22617819 | ST3GAL3;ST3GAL3;S | Body;Body;Body;Bo | NA | -9.28E-03 | 3.87E-05 |
| cg22624022 | NA | NA | Island | 8.35E-03 | 8.81E-05 |
| cg22629111 | FAM78B | Body | Island | 1.25E-02 | 8.48E-06 |
| cg22637867 | ZNF709 | 5'UTR | Island | 1.11E-02 | 2.70E-06 |
| cg22639242 | MGAT1;MGAT1;MG | 5'UTR;5'UTR;5'UTR; | N_Shelf | -1.04E-02 | 1.70E-10 |
| cg22646937 | KCNMB1;KCNIP1 | TSS200;Body | NA | -8.69E-03 | 1.10E-13 |
| cg22666373 | NA | NA | N_Shore | 8.04E-03 | 1.62E-05 |
| cg22692362 | LRRC4C | TSS1500 | NA | -1.35E-02 | 7.67E-10 |
| cg22694818 | LHX8 | 5'UTR | Island | 1.29E-02 | 2.94E-07 |
| cg22697325 | MACF1 | Body | NA | -1.22E-02 | 2.52E-10 |
| cg22697825 | CAPN2;CAPN2 | TSS1500;Body | N_Shore | -6.75E-03 | 1.52E-07 |
| cg22706883 | C15orf61 | Body | S_Shore | -1.55E-02 | 3.24E-08 |
| cg22711111 | TRIM15 | Body | Island | 1.30E-02 | 9.67E-05 |
| cg22720041 | ZNF229 | TSS200 | Island | 2.01E-02 | 6.17E-08 |
| cg22730004 | SPTA1 | TSS1500 | NA | -4.11E-02 | 4.45E-06 |
| cg22736354 | NHLRC1 | 1stExon | Island | 1.16E-02 | 7.42E-15 |
| cg22737154 | NA | NA | NA | -7.40E-03 | 8.81E-07 |
| cg22739858 | FGF1;FGF1;FGF1;FG | 5'UTR;5'UTR;5'UTR; | NA | -6.93E-03 | 1.69E-06 |
| cg22757824 | COL4A1;COL4A2 | TSS1500;5'UTR | Island | 1.40E-02 | 1.80E-07 |
| cg22764193 | API5;API5;API5;API5 | TSS1500;TSS1500;T | N_Shore | -1.69E-02 | 1.09E-07 |
| cg22777186 | PKNOX2 | 5'UTR | NA | -8.70E-03 | 3.97E-06 |
| cg22788465 | IL3 | 3'UTR | NA | -9.36E-03 | 1.24E-09 |
| cg22796507 | FOXE3 | 1stExon | Island | 2.04E-02 | 1.06E-07 |
| cg22796572 | ATCAY | TSS200 | N_Shore | 9.01E-03 | 1.92E-05 |
| cg22796704 | ARHGAP22 | Body | N_Shore | -1.15E-02 | 3.38E-16 |
| cg22797031 | NA | NA | N_Shore | 2.31E-02 | 1.10E-05 |
| cg22798977 | PDPN;PDPN | TSS1500;TSS1500 | Island | 7.50E-03 | 1.89E-05 |
| cg22802102 | NTF3 | Body | NA | -8.89E-03 | 4.25E-06 |
| cg22802813 | USP44;USP44 | TSS200;5'UTR | Island | 1.43E-02 | 6.45E-06 |
| cg22815110 | FOXD3 | 1stExon | Island | 1.29E-02 | 1.20E-09 |
| cg22820364 | NA | NA | N_Shore | -2.26E-02 | 1.48E-19 |
| cg22824998 | PNMAL1;PNMAL1 | TSS200;TSS200 | Island | 9.83E-03 | 1.30E-05 |
| cg22830707 | HOXC13 | TSS1500 | N_Shore | 6.95E-03 | 1.92E-07 |
| cg22835712 | NA | NA | NA | -1.65E-02 | 2.32E-11 |
| cg22837726 | C19orf45 | Body | Island | 2.77E-02 | 2.63E-06 |
| cg22848150 | PENK;PENK | Body;Body | Island | 1.13E-02 | 1.87E-07 |
| cg22851420 | HPCAL4 | Body | Island | 1.39E-02 | 4.08E-07 |
| cg22864266 | FKBP11;FKBP11;FKB | TSS1500;TSS1500;T | S_Shore | -9.24E-03 | 6.49E-06 |
| cg22868111 | NA | NA | NA | -1.05E-02 | 8.85E-08 |
| cg22872478 | LYSMD2;LYSMD2 | 5'UTR;TSS1500 | S_Shore | -1.55E-02 | 1.40E-08 |
| cg22879583 | FDXACB1;C11orf1 | TSS1500;Body | S_Shore | -8.92E-03 | 2.53E-05 |
| cg22883332 | CALML3 | TSS1500 | N_Shore | -7.17E-03 | 3.85E-05 |
| cg22883472 | EBF2 | Body | NA | 8.15E-03 | 4.46E-05 |

| | | | | | |
|---|---|---|---|---|---|
| cg22888967 | SLC46A3;SLC46A3 | TSS1500;TSS1500 | S_Shore | -9.56E-03 | 2.35E-06 |
| cg22891191 | PTPRE | 5'UTR | Island | -1.15E-02 | 3.68E-07 |
| cg22891500 | NA | NA | Island | 1.19E-02 | 1.32E-05 |
| cg22892904 | CBX2;CBX2 | TSS1500;TSS1500 | N_Shore | -1.05E-02 | 9.64E-11 |
| cg22894896 | MIR193A | TSS200 | Island | 1.23E-02 | 1.35E-06 |
| cg22900476 | RAPGEF4 | Body | NA | -1.38E-02 | 1.44E-08 |
| cg22912156 | ADARB2 | Body | S_Shelf | -6.82E-03 | 8.60E-05 |
| cg22914338 | NA | NA | Island | -2.79E-02 | 8.62E-06 |
| cg22920258 | MCHR1 | TSS1500 | NA | -8.47E-03 | 1.26E-07 |
| cg22927302 | SEMA3B;SEMA3B | TSS1500;TSS1500 | NA | -5.58E-03 | 8.65E-06 |
| cg22934295 | CCK;CCK | 1stExon;5'UTR | Island | 2.18E-02 | 6.19E-12 |
| cg22940798 | TAP2;TAP2 | Body;Body | N_Shore | -9.42E-03 | 2.95E-13 |
| cg22942576 | CSRP1;CSRP1 | TSS1500;TSS1500 | S_Shore | -8.26E-03 | 4.67E-11 |
| cg22950493 | NA | NA | S_Shelf | -2.35E-02 | 3.68E-21 |
| cg22950598 | GDF11 | Body | S_Shelf | -9.19E-03 | 8.92E-08 |
| cg22962923 | NA | NA | Island | -6.46E-03 | 1.42E-07 |
| cg22975671 | BCL6B | TSS200 | Island | 6.14E-03 | 3.00E-05 |
| cg22986597 | FAM89B;FAM89B;FA | 3'UTR;Body;Body | N_Shore | -1.16E-02 | 2.49E-08 |
| cg22987487 | EBF2 | Body | NA | 3.54E-02 | 1.61E-08 |
| cg22995692 | NA | NA | Island | 1.68E-02 | 2.83E-12 |
| cg23003783 | GRM6;GRM6 | 1stExon;5'UTR | Island | 7.89E-03 | 8.01E-06 |
| cg23005227 | CISH;CISH | Body;Body | N_Shelf | -1.16E-02 | 4.46E-09 |
| cg23012600 | NA | NA | NA | -9.15E-03 | 5.39E-07 |
| cg23014425 | HOXB3 | 5'UTR | NA | -3.20E-02 | 3.59E-09 |
| cg23018448 | QTRT1 | TSS1500 | N_Shore | -1.07E-02 | 4.44E-05 |
| cg23045972 | NA | NA | NA | -8.78E-03 | 1.36E-07 |
| cg23052220 | TMEM132E | Body | S_Shelf | -1.52E-02 | 1.47E-10 |
| cg23052776 | NA | NA | NA | -1.07E-02 | 1.26E-07 |
| cg23053961 | GLIS3 | TSS1500 | S_Shore | -1.50E-02 | 3.90E-09 |
| cg23062198 | FAM84A | 5'UTR | Island | 1.59E-02 | 3.16E-08 |
| cg23065934 | 11-mar | TSS1500 | Island | 1.91E-02 | 2.62E-05 |
| cg23077820 | PAX3;PAX3;PAX3;PA | Body;Body;Body;Bo | N_Shore | 9.66E-03 | 1.53E-05 |
| cg23078123 | GPR177 | Body | NA | -1.77E-02 | 4.65E-26 |
| cg23080427 | CYP4F12 | TSS1500 | NA | -8.94E-03 | 3.48E-12 |
| cg23085281 | FRMPD2;FRMPD2 | Body;Body | NA | -7.38E-03 | 5.90E-05 |
| cg23085662 | NA | NA | Island | 8.13E-03 | 5.40E-07 |
| cg23091758 | NRIP3 | TSS200 | Island | 2.99E-02 | 2.75E-12 |
| cg23092040 | NA | NA | Island | 1.05E-02 | 8.28E-07 |
| cg23093580 | NDUFS5 | TSS1500 | NA | -1.26E-02 | 1.58E-17 |
| cg23093589 | SP8;SP8 | Body;Body | Island | 1.08E-02 | 7.32E-05 |
| cg23095192 | ZEB2;ZEB2;ZEB2 | Body;Body;Body | N_Shelf | -6.04E-03 | 6.77E-05 |
| cg23100720 | NOX3 | Body | NA | -1.27E-02 | 4.87E-14 |
| cg23112821 | NA | NA | N_Shore | -2.84E-02 | 3.31E-18 |
| cg23117999 | C14orf132 | Body | S_Shore | -4.90E-03 | 3.63E-05 |
| cg23119026 | NA | NA | NA | -5.97E-03 | 7.53E-05 |
| cg23124451 | CBX7 | Body | Island | -6.62E-03 | 1.01E-06 |
| cg23130254 | HOXD12 | 1stExon | Island | 1.19E-02 | 8.68E-10 |
| cg23149300 | NA | NA | NA | -1.89E-02 | 5.44E-20 |
| cg23151024 | NA | NA | Island | 1.00E-02 | 6.49E-05 |
| cg23155606 | NA | NA | Island | 7.94E-03 | 1.70E-07 |

| | | | | | |
|---|---|---|---|---|---|
| cg23163653 | NA | NA | NA | -1.49E-02 | 3.15E-11 |
| cg23169957 | MACROD2 | TSS200 | Island | 1.71E-02 | 2.18E-06 |
| cg23175533 | KIAA1467 | TSS1500 | N_Shore | -1.42E-02 | 3.84E-05 |
| cg23181159 | FAM8A1 | TSS1500 | N_Shore | -1.63E-02 | 2.95E-10 |
| cg23184070 | ZNF777 | 5'UTR | Island | 6.39E-03 | 7.96E-06 |
| cg23191801 | NA | NA | S_Shore | -7.81E-03 | 2.22E-06 |
| cg23192824 | GPR126;GPR126;GP | Body;Body;Body;Bo | NA | -1.87E-02 | 6.13E-06 |
| cg23195510 | RLTPR | TSS1500 | Island | -1.16E-02 | 3.40E-11 |
| cg23197460 | SMARCA5 | Body | S_Shore | -1.21E-02 | 3.50E-09 |
| cg23200020 | LOC643719 | Body | Island | 1.79E-02 | 3.70E-05 |
| cg23202913 | RPS20;RPS20 | Body;Body | N_Shore | -1.21E-02 | 7.69E-07 |
| cg23206160 | MAML3 | Body | NA | -7.57E-03 | 1.12E-05 |
| cg23207412 | NA | NA | NA | -9.33E-03 | 5.96E-06 |
| cg23207527 | RBM24;RBM24;RBM | 1stExon;Body;Body | S_Shore | -1.42E-02 | 8.57E-09 |
| cg23211240 | MIR34B;BTG4;MIR3 | TSS200;TSS1500;TS | Island | 1.91E-02 | 3.77E-09 |
| cg23212786 | NA | NA | N_Shelf | 1.67E-02 | 5.82E-14 |
| cg23216612 | RINT1 | TSS1500 | N_Shore | -1.30E-02 | 1.77E-08 |
| cg23217622 | NA | NA | Island | 2.67E-02 | 2.65E-06 |
| cg23229770 | NA | NA | N_Shelf | -1.37E-02 | 7.10E-07 |
| cg23230929 | NA | NA | NA | -8.02E-03 | 2.18E-11 |
| cg23231163 | FUT11 | Body | S_Shore | -1.67E-02 | 2.22E-12 |
| cg23233742 | NA | NA | S_Shore | -6.73E-03 | 1.44E-05 |
| cg23244289 | THBS4 | 5'UTR | Island | 3.64E-02 | 8.78E-10 |
| cg23245711 | FBXW12;FBXW12;FE | TSS200;TSS200;TSS: | NA | -8.90E-03 | 3.20E-08 |
| cg23248150 | TLL1 | Body | S_Shore | 8.99E-03 | 4.63E-05 |
| cg23251200 | NA | NA | NA | -8.54E-03 | 4.28E-09 |
| cg23254497 | NA | NA | S_Shore | -9.74E-03 | 1.51E-08 |
| cg23256579 | PRR4;PRR4 | TSS1500;Body | NA | -1.60E-02 | 7.82E-08 |
| cg23260547 | PROX1 | TSS1500 | Island | 2.69E-02 | 7.41E-09 |
| cg23262897 | PCDHGA2;PCDHGA1 | 1stExon;Body;1stEx | N_Shore | 1.14E-02 | 1.05E-05 |
| cg23274030 | SFXN4 | TSS1500 | S_Shore | -1.28E-02 | 1.46E-08 |
| cg23284720 | NA | NA | Island | 7.34E-03 | 1.44E-06 |
| cg23316648 | NA | NA | NA | -1.07E-02 | 6.80E-05 |
| cg23318063 | NA | NA | Island | 1.71E-02 | 5.78E-06 |
| cg23319477 | TAPBP;TAPBP;RGL2; | 3'UTR;3'UTR;TSS15( | S_Shore | 7.94E-03 | 5.92E-05 |
| cg23320649 | C3orf18 | 5'UTR | N_Shore | -6.18E-03 | 1.01E-09 |
| cg23325364 | DIP2C | Body | NA | -1.02E-02 | 1.07E-13 |
| cg23331484 | SLC7A10 | TSS1500 | S_Shore | 1.20E-02 | 1.33E-07 |
| cg23341182 | BLOC1S2;BLOC1S2 | TSS1500;TSS1500 | S_Shore | -1.76E-02 | 3.29E-18 |
| cg23349517 | NA | NA | N_Shelf | -5.44E-03 | 7.87E-06 |
| cg23368787 | ATP4A | Body | Island | 5.41E-03 | 9.14E-05 |
| cg23371208 | MIR130A | TSS200 | NA | -5.50E-03 | 3.65E-06 |
| cg23386330 | NA | NA | NA | -1.03E-02 | 9.59E-08 |
| cg23397578 | NA | NA | Island | 1.17E-02 | 2.01E-09 |
| cg23400715 | FAM19A5 | Body | NA | -8.37E-03 | 1.51E-05 |
| cg23402821 | NA | NA | Island | 1.70E-02 | 6.50E-10 |
| cg23409168 | NA | NA | NA | -1.66E-02 | 5.02E-07 |
| cg23418219 | CREB5 | TSS1500 | NA | -1.13E-02 | 1.45E-10 |
| cg23435594 | NA | NA | NA | -1.01E-02 | 7.49E-09 |
| cg23436584 | NA | NA | NA | -1.04E-02 | 1.96E-12 |

| | | | | | |
|---|---|---|---|---|---|
| cg23437162 | QRICH1;IMPDH2;QF | 3'UTR;TSS1500;3'U | S_Shore | -6.56E-03 | 2.57E-05 |
| cg23437216 | LMBR1L | Body | S_Shelf | -4.74E-03 | 7.05E-06 |
| cg23445859 | B3GNT4 | 5'UTR | Island | 1.90E-02 | 1.24E-07 |
| cg23449696 | ZIC1 | TSS1500 | N_Shore | 1.55E-02 | 1.36E-12 |
| cg23464360 | MAFA | TSS1500 | S_Shore | -7.98E-03 | 2.77E-06 |
| cg23467079 | FAM83A;FAM83A | Body;Body | Island | 2.31E-02 | 2.77E-06 |
| cg23469878 | LCN6 | Body | Island | -1.15E-02 | 2.22E-06 |
| cg23479922 | 11-mar | 1stExon | Island | 2.91E-02 | 4.37E-14 |
| cg23481795 | PCDHB10 | 1stExon | Island | 4.25E-03 | 4.82E-05 |
| cg23482132 | FMNL2 | Body | NA | -1.20E-02 | 3.20E-06 |
| cg23485738 | SKIV2L | Body | N_Shelf | -6.56E-03 | 2.48E-05 |
| cg23487201 | APCDD1L | TSS200 | Island | 9.70E-03 | 1.65E-07 |
| cg23488804 | RUNDC3A;RUNDC3A | Body;Body;Body | S_Shore | 9.81E-03 | 9.01E-13 |
| cg23489273 | HLA-A | TSS200 | Island | 1.75E-02 | 6.17E-05 |
| cg23492779 | PI16 | Body | N_Shore | -1.19E-02 | 1.27E-05 |
| cg23495748 | CPLX2;CPLX2 | 5'UTR;1stExon | Island | 1.91E-02 | 1.87E-08 |
| cg23495995 | LTBP3;LTBP3;LTBP3 | Body;Body;Body | N_Shelf | -1.27E-02 | 6.66E-18 |
| cg23500122 | GHSR;GHSR | TSS1500;TSS1500 | Island | 1.38E-02 | 1.14E-07 |
| cg23500537 | NA | NA | NA | 1.23E-02 | 8.52E-15 |
| cg23510415 | KIAA1467 | Body | NA | -7.69E-03 | 1.94E-08 |
| cg23531488 | NDUFA5 | TSS1500 | S_Shore | -1.69E-02 | 9.01E-08 |
| cg23534245 | BMP2 | Body | S_Shore | -2.09E-02 | 2.12E-25 |
| cg23543123 | SLC10A4;SLC10A4 | 5'UTR;1stExon | Island | 2.10E-02 | 2.70E-06 |
| cg23549358 | NELL1;NELL1 | Body;Body | S_Shore | 1.25E-02 | 1.77E-07 |
| cg23553442 | NA | NA | Island | 1.14E-02 | 1.15E-08 |
| cg23553762 | GALNTL1;GALNTL1 | Body;Body | NA | -1.16E-02 | 9.52E-20 |
| cg23565821 | CUTA;CUTA;CUTA;C | Body;Body;Body;Bo | N_Shore | -7.10E-03 | 1.69E-05 |
| cg23572163 | psiTPTE22 | Body | S_Shore | 9.48E-03 | 2.57E-06 |
| cg23583920 | CD300LG;CD300LG;( | TSS1500;TSS1500;T | NA | -1.53E-02 | 3.29E-07 |
| cg23585420 | NA | NA | NA | -8.09E-03 | 4.31E-05 |
| cg23592947 | NA | NA | Island | 1.26E-02 | 7.87E-10 |
| cg23597629 | ACTA1 | 5'UTR | Island | 1.04E-02 | 1.51E-06 |
| cg23606718 | FAM123C;FAM123C | 5'UTR;5'UTR;1stExo | Island | 1.39E-02 | 9.87E-08 |
| cg23619217 | IL25;IL25 | TSS1500;TSS1500 | NA | -5.59E-03 | 3.06E-05 |
| cg23625628 | TAPBP;TAPBP;RGL2; | 3'UTR;3'UTR;TSS15( | S_Shore | 1.55E-02 | 6.07E-07 |
| cg23625823 | ODZ3 | Body | NA | -1.38E-02 | 2.38E-13 |
| cg23626733 | PEX10;PEX10 | TSS1500;TSS1500 | N_Shore | -1.22E-02 | 6.52E-09 |
| cg23627335 | NA | NA | S_Shore | 1.21E-02 | 6.20E-05 |
| cg23629166 | PEX10;PEX10 | TSS1500;TSS1500 | N_Shore | -1.37E-02 | 1.15E-07 |
| cg23632393 | NA | NA | S_Shore | -1.05E-02 | 8.41E-10 |
| cg23632849 | TTYH2;MGC16275 | TSS1500;Body | N_Shore | -8.14E-03 | 6.78E-14 |
| cg23635560 | NA | NA | NA | -5.73E-03 | 1.90E-06 |
| cg23635789 | SRC;SRC | Body;Body | Island | -1.15E-02 | 1.33E-05 |
| cg23651356 | RGS17 | TSS1500 | S_Shore | -7.82E-03 | 2.53E-07 |
| cg23677657 | DLX6AS | Body | NA | 8.94E-03 | 1.77E-12 |
| cg23681440 | NA | NA | NA | -1.27E-02 | 1.05E-12 |
| cg23683497 | CHIT1 | Body | NA | -7.35E-03 | 1.10E-05 |
| cg23688719 | LEPR;LEPR;LEPR | 5'UTR;5'UTR;5'UTR | NA | -1.85E-02 | 5.64E-09 |
| cg23693245 | NA | NA | NA | -1.52E-02 | 5.83E-06 |
| cg23715749 | GRIK3 | Body | NA | -1.38E-02 | 4.51E-11 |

| | | | | | |
|---|---|---|---|---|---|
| cg23715830 | NA | NA | Island | 1.26E-02 | 8.39E-06 |
| cg23718736 | L3MBTL4 | 5'UTR | N_Shore | -6.88E-03 | 7.99E-05 |
| cg23726537 | CRCT1 | Body | Island | 9.08E-03 | 9.37E-06 |
| cg23737927 | NA | NA | N_Shelf | -1.29E-02 | 7.80E-07 |
| cg23740491 | NA | NA | Island | 9.94E-03 | 1.52E-07 |
| cg23744638 | NA | NA | N_Shore | -2.81E-02 | 1.17E-21 |
| cg23746497 | NA | NA | Island | 1.62E-02 | 1.26E-08 |
| cg23752828 | DCTN2 | TSS1500 | S_Shore | -1.17E-02 | 1.86E-08 |
| cg23753748 | CALHM2;CALHM2 | TSS1500;TSS1500 | S_Shore | -1.18E-02 | 1.34E-13 |
| cg23762517 | HIVEP3;HIVEP3;HIVE | 1stExon;1stExon;5'U | S_Shore | -1.99E-02 | 7.96E-11 |
| cg23769143 | SYN2;TIMP4;SYN2 | Body;TSS200;Body | S_Shore | -7.83E-03 | 7.47E-07 |
| cg23770904 | GATA5 | TSS1500 | Island | 1.68E-02 | 2.47E-06 |
| cg23779626 | NA | NA | NA | -1.34E-02 | 8.55E-12 |
| cg23786883 | RASSF2;RASSF2 | 3'UTR;3'UTR | NA | -2.95E-02 | 3.72E-07 |
| cg23792308 | COX10 | Body | NA | -9.10E-03 | 1.15E-05 |
| cg23798387 | KIAA0100 | TSS1500 | S_Shore | -1.66E-02 | 2.43E-06 |
| cg23798789 | NA | NA | N_Shelf | -8.93E-03 | 6.62E-06 |
| cg23803337 | NA | NA | N_Shore | 1.26E-02 | 2.12E-05 |
| cg23804481 | PRUNE2 | TSS200 | Island | 8.13E-03 | 5.36E-06 |
| cg23805357 | LOC100128811;GPR | Body;TSS200 | Island | 1.51E-02 | 3.29E-05 |
| cg23836704 | NRAP;NRAP | Body;Body | NA | -8.21E-03 | 4.94E-05 |
| cg23836737 | NA | NA | NA | -1.31E-02 | 6.53E-11 |
| cg23837265 | C10orf116;AGAP11 | 3'UTR;TSS200 | N_Shore | -1.06E-02 | 5.92E-06 |
| cg23845889 | MTMR7 | Body | Island | 7.85E-03 | 1.98E-05 |
| cg23850283 | NA | NA | Island | 1.45E-02 | 4.50E-05 |
| cg23861420 | NA | NA | NA | 1.32E-02 | 3.72E-06 |
| cg23869158 | LRCH1;LRCH1;LRCH1 | TSS1500;TSS1500;T | N_Shore | -1.77E-02 | 2.02E-11 |
| cg23878494 | NA | NA | S_Shore | -7.25E-03 | 2.18E-05 |
| cg23881278 | DRD2;DRD2 | TSS1500;TSS1500 | Island | 2.45E-02 | 4.18E-08 |
| cg23883058 | NA | NA | S_Shore | 1.11E-02 | 1.02E-07 |
| cg23883696 | NETO1;NETO1;NETO | TSS1500;TSS1500;B | Island | 1.83E-02 | 1.94E-05 |
| cg23893898 | NA | NA | Island | 1.17E-02 | 2.35E-07 |
| cg23895722 | C11orf45;KCNJ5 | TSS1500;5'UTR | NA | -9.93E-03 | 2.31E-08 |
| cg23900203 | NA | NA | Island | 1.72E-02 | 4.10E-09 |
| cg23901212 | NA | NA | NA | -1.06E-02 | 4.90E-08 |
| cg23907053 | RAB3IP;RAB3IP;RAB | 3'UTR;3'UTR;3'UTR; | NA | -1.50E-02 | 1.35E-07 |
| cg23913963 | RCC2 | TSS1500 | S_Shore | -1.54E-02 | 1.86E-10 |
| cg23914149 | NA | NA | NA | -1.17E-02 | 7.75E-09 |
| cg23919111 | NA | NA | Island | -7.42E-03 | 3.20E-06 |
| cg23922259 | NA | NA | Island | 1.62E-02 | 4.11E-07 |
| cg23933216 | PDE4DIP | Body | NA | -1.20E-02 | 7.86E-19 |
| cg23934533 | NA | NA | Island | 1.93E-02 | 1.48E-07 |
| cg23934731 | NA | NA | Island | 1.83E-02 | 1.34E-05 |
| cg23950157 | COL1A1 | Body | N_Shore | -1.01E-02 | 1.57E-06 |
| cg23954268 | NA | NA | Island | 9.83E-03 | 9.49E-07 |
| cg23960707 | SFRS1;SFRS1 | Body;Body | N_Shore | -1.03E-02 | 1.33E-06 |
| cg23963802 | NA | NA | Island | 1.08E-02 | 3.04E-05 |
| cg23968154 | CACNG4 | Body | NA | -9.84E-03 | 5.23E-05 |
| cg23973429 | GRHL2;GRHL2 | 1stExon;5'UTR | Island | 1.54E-02 | 4.83E-08 |
| cg23973524 | CRTC1;CRTC1 | Body;Body | Island | -7.67E-03 | 2.71E-05 |

| | | | | | |
|---|---|---|---|---|---|
| cg23982858 | USP44;USP44 | 5'UTR;5'UTR | N_Shore | 7.32E-03 | 2.67E-05 |
| cg23989004 | ZNF709 | TSS200 | S_Shore | 1.12E-02 | 6.66E-06 |
| cg23993425 | FAM43B;FAM43B | 1stExon;3'UTR | Island | 7.29E-03 | 2.86E-05 |
| cg23995459 | ZYG11B | TSS1500 | N_Shore | -1.22E-02 | 1.04E-05 |
| cg23995914 | ZNF518B | TSS200 | Island | 2.23E-02 | 6.36E-13 |
| cg23998119 | ZDHHC22 | Body | NA | 1.34E-02 | 1.35E-06 |
| cg23998240 | INF2;INF2;INF2 | 5'UTR;5'UTR;5'UTR | N_Shore | -9.85E-03 | 1.05E-07 |
| cg23999080 | PRDM16;PRDM16 | Body;Body | N_Shelf | -8.02E-03 | 2.16E-08 |
| cg24008177 | KIAA1370 | TSS1500 | S_Shore | -1.12E-02 | 5.75E-07 |
| cg24024056 | NA | NA | Island | 1.45E-02 | 8.90E-06 |
| cg24028809 | RGS17 | 5'UTR | NA | -1.31E-02 | 2.13E-07 |
| cg24035245 | NA | NA | Island | 1.29E-02 | 1.72E-07 |
| cg24041541 | LOC100192378 | Body | N_Shelf | 2.54E-02 | 1.73E-13 |
| cg24057710 | KIRREL3;KIRREL3 | Body;Body | NA | -7.81E-03 | 1.89E-12 |
| cg24058132 | GALC;GALC;GALC;G/ | 1stExon;5'UTR;1stE: | S_Shore | -9.10E-03 | 2.40E-09 |
| cg24071057 | EZH1 | Body | NA | -1.55E-02 | 1.58E-07 |
| cg24079702 | FHL2;FHL2;FHL2;FHI | TSS200;TSS200;5'U | Island | 1.40E-02 | 2.60E-09 |
| cg24092282 | NA | NA | Island | 1.09E-02 | 1.81E-06 |
| cg24099956 | NA | NA | S_Shore | -9.13E-03 | 2.86E-10 |
| cg24116870 | ADAMTS20 | TSS1500 | Island | 9.27E-03 | 4.61E-05 |
| cg24125710 | NA | NA | NA | -2.55E-02 | 6.56E-06 |
| cg24134897 | TSPAN4;TSPAN4;TSI | 5'UTR;Body;Body;B | S_Shore | -3.07E-02 | 3.49E-12 |
| cg24136205 | ZIC5 | TSS200 | Island | 1.59E-02 | 2.94E-09 |
| cg24139093 | NA | NA | NA | 1.61E-02 | 9.42E-10 |
| cg24139443 | NA | NA | N_Shelf | -1.17E-02 | 3.87E-08 |
| cg24144893 | UROD | Body | S_Shelf | -1.21E-02 | 2.31E-09 |
| cg24150153 | ARHGAP29 | TSS200 | S_Shore | 1.04E-02 | 9.74E-07 |
| cg24159636 | NA | NA | NA | -1.57E-02 | 1.21E-17 |
| cg24168221 | CST9 | TSS1500 | NA | -8.34E-03 | 3.04E-05 |
| cg24176563 | EYA4;EYA4;EYA4;EY/ | 5'UTR;1stExon;1stE: | Island | 1.25E-02 | 3.31E-07 |
| cg24189904 | ZNF177 | 5'UTR | Island | 8.61E-03 | 6.03E-05 |
| cg24195878 | MAST3 | Body | S_Shore | -1.14E-02 | 7.47E-12 |
| cg24198558 | TMEM108;TMEM10 | TSS200;TSS200 | Island | 2.73E-02 | 8.15E-09 |
| cg24202131 | BRUNOL4;BRUNOL4 | Body;Body;Body;Bo | S_Shore | 1.26E-02 | 8.81E-05 |
| cg24212517 | GRN | Body | N_Shelf | -6.69E-03 | 4.71E-05 |
| cg24217948 | SETBP1;SETBP1 | 5'UTR;5'UTR | S_Shore | -2.28E-02 | 2.18E-19 |
| cg24221541 | PNMAL1;PNMAL1;P | 1stExon;1stExon;5'l | Island | 1.78E-02 | 6.45E-07 |
| cg24221648 | NA | NA | Island | 2.48E-02 | 3.50E-07 |
| cg24222546 | STRA6;STRA6;STRA6 | TSS1500;TSS1500;T | NA | -9.92E-03 | 6.84E-08 |
| cg24227728 | ELAVL3;ELAVL3 | Body;Body | Island | 1.48E-02 | 1.72E-06 |
| cg24229750 | UBQLN3 | TSS200 | NA | -9.77E-03 | 4.66E-07 |
| cg24239148 | C10orf107;C10orf1C | 1stExon;5'UTR | Island | 3.10E-02 | 3.31E-08 |
| cg24245418 | NPY | Body | Island | 2.95E-02 | 4.42E-05 |
| cg24253904 | NAV1 | Body | NA | -9.92E-03 | 4.29E-06 |
| cg24259363 | NA | NA | NA | -1.25E-02 | 5.98E-12 |
| cg24293914 | DNTT;DNTT | Body;Body | NA | -1.29E-02 | 1.15E-09 |
| cg24301620 | GRIK2;GRIK2;GRIK2; | 1stExon;1stExon;5'l | Island | 1.98E-02 | 1.85E-17 |
| cg24304919 | KCNN2 | Body | Island | 1.08E-02 | 1.19E-06 |
| cg24308336 | C20orf95 | Body | N_Shelf | -1.23E-02 | 1.39E-10 |
| cg24308389 | FEZF1;FEZF1 | TSS1500;TSS1500 | Island | 9.11E-03 | 3.94E-05 |

| | | | | | |
|---|---|---|---|---|---|
| cg24311382 | CTGF;CTGF | 1stExon;5'UTR | Island | 2.63E-02 | 4.55E-05 |
| cg24312985 | THAP3 | TSS1500 | N_Shore | -7.60E-03 | 1.01E-06 |
| cg24321297 | ST6GALNAC2 | Body | N_Shelf | -7.52E-03 | 3.37E-07 |
| cg24331384 | TCHP;TCHP | TSS1500;TSS1500 | N_Shore | -8.35E-03 | 7.98E-05 |
| cg24333189 | TERC | TSS1500 | S_Shore | -1.31E-02 | 2.77E-07 |
| cg24338780 | CACYBP;CACYBP | TSS1500;TSS1500 | N_Shore | 1.01E-02 | 1.33E-07 |
| cg24350475 | KIAA1462 | Body | NA | -1.26E-02 | 1.10E-11 |
| cg24353443 | PDZRN3 | TSS200 | Island | 1.23E-02 | 2.10E-05 |
| cg24355907 | NA | NA | S_Shore | 8.07E-03 | 4.07E-07 |
| cg24366557 | TFAP2B | Body | Island | 1.88E-02 | 1.15E-12 |
| cg24376689 | TNXB | Body | NA | -1.35E-02 | 2.76E-06 |
| cg24378227 | NA | NA | S_Shelf | -1.00E-02 | 6.18E-08 |
| cg24394631 | CRHR1;CRHR1;CRHF | Body;Body;Body;Bo | S_Shore | 9.15E-03 | 6.24E-05 |
| cg24395452 | SPATA18 | Body | Island | 2.27E-02 | 5.93E-12 |
| cg24407086 | NA | NA | NA | -1.29E-02 | 5.04E-12 |
| cg24427243 | TNFRSF8 | Body | S_Shelf | -2.27E-02 | 2.61E-16 |
| cg24429836 | LDHD;LDHD | TSS200;TSS200 | S_Shore | -1.36E-02 | 2.16E-06 |
| cg24430140 | NA | NA | Island | 2.86E-02 | 2.75E-06 |
| cg24433287 | MANEAL;MANEAL;N | Body;Body;TSS200 | S_Shore | 1.05E-02 | 7.83E-06 |
| cg24434387 | NA | NA | N_Shelf | -1.17E-02 | 2.67E-12 |
| cg24436715 | NA | NA | Island | 1.19E-02 | 5.02E-06 |
| cg24436906 | BOK | TSS200 | Island | 1.32E-02 | 2.43E-09 |
| cg24439334 | PEX10;PEX10 | TSS1500;TSS1500 | N_Shore | -9.86E-03 | 8.26E-05 |
| cg24446586 | HOXA11AS;HOXA11 | Body;TSS1500 | Island | 2.37E-02 | 4.35E-09 |
| cg24446932 | NA | NA | NA | -7.02E-03 | 1.07E-07 |
| cg24452260 | GRIA2;GRIA2;GRIA2 | Body;Body;Body | Island | 1.50E-02 | 2.75E-10 |
| cg24456981 | LOC731789 | TSS1500 | NA | -1.36E-02 | 4.40E-09 |
| cg24466241 | ZYG11A | Body | Island | 2.63E-02 | 1.19E-14 |
| cg24468070 | CDC42EP5 | Body | Island | 2.46E-02 | 1.96E-05 |
| cg24472477 | CHIT1 | 3'UTR | NA | -9.80E-03 | 3.27E-06 |
| cg24474319 | CRABP2 | Body | N_Shelf | -4.48E-03 | 2.69E-05 |
| cg24482747 | NA | NA | N_Shelf | -9.34E-03 | 1.22E-06 |
| cg24488739 | RBM20 | Body | N_Shore | -1.15E-02 | 4.11E-06 |
| cg24488891 | LOC100128811;GPR | Body;TSS200 | Island | 2.09E-02 | 1.28E-09 |
| cg24493446 | GPR176 | 1stExon | Island | 1.14E-02 | 1.75E-05 |
| cg24511404 | NENF;NENF | Body;Body | S_Shore | -1.18E-02 | 7.74E-12 |
| cg24527434 | SDK2 | Body | NA | -8.35E-03 | 4.45E-08 |
| cg24535475 | C1S;C1S | TSS200;TSS200 | NA | -1.91E-02 | 2.00E-21 |
| cg24541550 | MRVI1;MRVI1;MRVI | TSS1500;1stExon;5' | NA | -6.76E-03 | 7.30E-05 |
| cg24543696 | DKK3;DKK3;DKK3 | TSS1500;TSS200;TS: | S_Shore | 1.66E-02 | 1.67E-09 |
| cg24554789 | TINAGL1 | Body | NA | -8.45E-03 | 1.70E-06 |
| cg24575083 | CYB5R2 | TSS1500 | Island | 1.19E-02 | 7.70E-05 |
| cg24575234 | CHRM2;CHRM2;CHF | 1stExon;5'UTR;5'UT | Island | 8.92E-03 | 5.70E-05 |
| cg24578124 | MADCAM1;MADCAI | Body;Body | Island | 5.01E-03 | 3.23E-06 |
| cg24585203 | BBOX1 | Body | NA | -1.07E-02 | 3.81E-10 |
| cg24587080 | NA | NA | NA | -1.74E-02 | 1.44E-16 |
| cg24590353 | UST | Body | NA | -2.04E-02 | 3.54E-21 |
| cg24611446 | NA | NA | Island | 1.40E-02 | 1.01E-11 |
| cg24617352 | IDH3B;IDH3B;IDH3B | 3'UTR;Body;3'UTR | NA | -7.46E-03 | 5.23E-07 |
| cg24622589 | NA | NA | NA | -1.03E-02 | 2.55E-08 |

| | | | | | |
|---|---|---|---|---|---|
| cg24624586 | NA | NA | NA | -8.65E-03 | 1.38E-07 |
| cg24628676 | PDE4D;PDE4D | Body;TSS1500 | Island | 7.64E-03 | 5.15E-06 |
| cg24631162 | BAT1;BAT1 | 5'UTR;5'UTR | N_Shore | -8.62E-03 | 1.70E-07 |
| cg24634060 | ARHGEF3 | 5'UTR | N_Shore | 7.38E-03 | 6.04E-05 |
| cg24639902 | PEBP4 | Body | NA | -9.47E-03 | 4.39E-13 |
| cg24665265 | NA | NA | NA | -7.93E-03 | 1.04E-06 |
| cg24667575 | NEURL1B | Body | Island | 5.14E-02 | 6.00E-09 |
| cg24674215 | NA | NA | NA | -1.29E-02 | 6.70E-10 |
| cg24676612 | KCNA2;KCNA2 | 5'UTR;1stExon | N_Shore | -5.00E-03 | 9.07E-05 |
| cg24679043 | TRIM10;TRIM10 | 1stExon;1stExon | NA | -9.70E-03 | 1.01E-08 |
| cg24679348 | PRRT3 | TSS1500 | S_Shore | -1.61E-02 | 3.27E-15 |
| cg24681086 | TSPAN18 | 5'UTR | NA | -1.18E-02 | 1.01E-09 |
| cg24690071 | ZIC2 | 1stExon | Island | 1.24E-02 | 1.48E-07 |
| cg24690479 | GCNT2 | TSS1500 | NA | -1.16E-02 | 6.53E-09 |
| cg24693287 | SERINC2 | TSS200 | Island | 1.98E-02 | 7.68E-06 |
| cg24694246 | NA | NA | N_Shore | -1.64E-02 | 6.26E-06 |
| cg24702091 | NA | NA | Island | 8.43E-03 | 3.34E-06 |
| cg24704287 | NA | NA | N_Shore | -8.63E-03 | 4.15E-05 |
| cg24707200 | NTRK1;NTRK1;NTRK | Body;Body;Body | S_Shelf | -9.29E-03 | 1.65E-13 |
| cg24708006 | NA | NA | Island | 9.67E-03 | 1.10E-06 |
| cg24711336 | NA | NA | S_Shore | -1.41E-02 | 8.83E-17 |
| cg24719321 | BSX | Body | N_Shore | 9.41E-03 | 3.08E-07 |
| cg24724428 | ELOVL2 | TSS1500 | Island | 2.53E-02 | 1.45E-18 |
| cg24736412 | DSE;DSE | 5'UTR;5'UTR | S_Shore | -1.32E-02 | 2.86E-07 |
| cg24761525 | HOXB3;HOXB3 | 5'UTR;1stExon | N_Shelf | -1.18E-02 | 6.95E-07 |
| cg24774812 | RHOJ | Body | NA | -1.86E-02 | 7.07E-12 |
| cg24789180 | NA | NA | N_Shore | 7.89E-03 | 3.93E-05 |
| cg24794228 | ZNF577;ZNF577;ZNF | Body;5'UTR;5'UTR;1 | Island | 2.69E-02 | 1.17E-09 |
| cg24795867 | WNT5B;WNT5B | Body;Body | N_Shelf | -1.02E-02 | 2.12E-08 |
| cg24803614 | FTH1 | Body | N_Shore | -2.40E-02 | 3.90E-21 |
| cg24806066 | NA | NA | N_Shore | -1.41E-02 | 3.96E-10 |
| cg24807169 | EMCN;EMCN | TSS1500;TSS1500 | NA | -1.29E-02 | 7.57E-08 |
| cg24825722 | ACADVL;DLG4;DLG4 | TSS1500;TSS1500;B | N_Shore | -8.37E-03 | 5.57E-08 |
| cg24826867 | IRF8 | 5'UTR | Island | 2.79E-02 | 1.61E-05 |
| cg24828167 | NA | NA | S_Shelf | -1.60E-02 | 2.00E-09 |
| cg24836946 | NA | NA | N_Shelf | -1.02E-02 | 1.78E-06 |
| cg24837277 | NA | NA | NA | -8.31E-03 | 2.49E-11 |
| cg24840300 | LPPR3 | 3'UTR | Island | 4.31E-02 | 6.36E-06 |
| cg24843346 | NA | NA | S_Shore | -7.90E-03 | 5.23E-06 |
| cg24847230 | UBE2Z | Body | S_Shore | -4.59E-03 | 1.07E-06 |
| cg24848615 | NFIC;NFIC | Body;Body | N_Shore | -9.78E-03 | 5.07E-09 |
| cg24850296 | IFLTD1;IFLTD1;IFLTD | TSS1500;TSS1500;T | NA | -1.34E-02 | 3.97E-10 |
| cg24851859 | NA | NA | NA | -1.44E-02 | 1.18E-08 |
| cg24853724 | TRIL | 1stExon | Island | 7.91E-03 | 8.76E-06 |
| cg24868150 | NA | NA | N_Shelf | -7.23E-03 | 5.05E-08 |
| cg24869601 | TFAP2A;TFAP2A | Body;Body | Island | 2.02E-02 | 5.34E-12 |
| cg24870966 | C17orf55 | 5'UTR | N_Shore | -1.28E-02 | 3.27E-07 |
| cg24872692 | PIWIL3 | Body | N_Shelf | -1.19E-02 | 2.54E-17 |
| cg24883498 | PIK3R2 | 5'UTR | N_Shore | -1.09E-02 | 1.56E-15 |
| cg24884142 | TBX15;TBX15 | 1stExon;5'UTR | Island | 1.19E-02 | 1.43E-09 |

| | | | | | |
|---|---|---|---|---|---|
| cg24885937 | PLEKHA7 | Body | NA | -9.45E-03 | 1.23E-07 |
| cg24892069 | NRP1;NRP1;NRP1 | Body;Body;Body | NA | -2.70E-02 | 7.67E-20 |
| cg24909548 | NA | NA | Island | 1.40E-02 | 1.13E-06 |
| cg24911113 | NA | NA | Island | 1.26E-02 | 2.33E-07 |
| cg24922090 | PCDHGA4;PCDHGB3 | Body;1stExon;Body; | Island | 9.24E-03 | 1.01E-06 |
| cg24923694 | NA | NA | Island | 1.34E-02 | 3.71E-06 |
| cg24924502 | AATK | Body | Island | -2.02E-02 | 8.82E-07 |
| cg24927800 | DES | 1stExon | Island | 2.84E-02 | 7.80E-11 |
| cg24927974 | DPY19L1 | TSS1500 | S_Shore | -1.64E-02 | 2.47E-11 |
| cg24931520 | NA | NA | Island | 7.20E-03 | 5.64E-05 |
| cg24935332 | NA | NA | N_Shore | 1.40E-02 | 1.61E-11 |
| cg24938830 | UNC80;UNC80 | TSS200;TSS200 | Island | 1.82E-02 | 2.76E-05 |
| cg24952158 | FHOD3 | Body | N_Shelf | -7.12E-03 | 1.60E-05 |
| cg24981400 | REV1;REV1 | Body;Body | NA | -4.85E-02 | 5.09E-06 |
| cg24986868 | PKP4;CCDC148;PKP4 | TSS1500;5'UTR;TSS1 | N_Shore | -2.11E-02 | 5.83E-08 |
| cg25008614 | SPG11;SPG11 | TSS1500;TSS1500 | S_Shore | -5.65E-03 | 1.30E-05 |
| cg25010508 | NA | NA | NA | -5.52E-03 | 2.59E-05 |
| cg25022866 | VSTM2A | TSS200 | Island | 1.55E-02 | 1.05E-05 |
| cg25023816 | NA | NA | NA | -1.65E-02 | 1.26E-12 |
| cg25027798 | PCDHA7;PCDHA13;P | Body;1stExon;Body; | Island | 1.66E-02 | 2.61E-08 |
| cg25031476 | PTPRR;PTPRR | Body;Body | NA | -1.68E-02 | 1.62E-08 |
| cg25033364 | KCNC1;KCNC1 | Body;Body | S_Shelf | 1.51E-02 | 3.30E-06 |
| cg25044651 | LVRN | 1stExon | Island | 7.45E-03 | 9.52E-05 |
| cg25047092 | NA | NA | S_Shore | 1.11E-02 | 1.85E-06 |
| cg25053336 | NA | NA | N_Shore | 9.92E-03 | 1.43E-05 |
| cg25057584 | GALNT6 | 3'UTR | NA | -6.45E-03 | 7.01E-05 |
| cg25061755 | STON1-GTF2A1L;STO | Body;1stExon | NA | -1.56E-02 | 2.70E-15 |
| cg25065723 | TCERG1L | Body | N_Shore | 9.45E-03 | 5.93E-07 |
| cg25068253 | NA | NA | NA | -1.66E-02 | 1.99E-05 |
| cg25073967 | KIAA0284;KIAA0284 | Body;Body | S_Shore | -8.64E-03 | 8.09E-08 |
| cg25078444 | FOXG1 | TSS1500 | Island | 3.11E-02 | 1.45E-11 |
| cg25085245 | NA | NA | N_Shore | -8.97E-03 | 2.16E-05 |
| cg25103895 | ADO;ADO | 3'UTR;1stExon | Island | -7.12E-03 | 6.61E-06 |
| cg25109663 | ZBTB20;ZBTB20;ZBT | 5'UTR;5'UTR;5'UTR; | NA | -1.25E-02 | 7.52E-06 |
| cg25112291 | RAX | Body | Island | 6.18E-03 | 6.98E-06 |
| cg25114913 | FUT9;FUT9 | 5'UTR;1stExon | Island | 1.53E-02 | 9.02E-05 |
| cg25123866 | PYGM;PYGM | TSS200;TSS200 | NA | -1.09E-02 | 1.36E-09 |
| cg25124276 | LOC100128811;GPR | Body;TSS1500 | Island | 2.03E-02 | 6.86E-09 |
| cg25124956 | FAT1 | 3'UTR | NA | -1.53E-02 | 2.88E-08 |
| cg25129541 | NA | NA | N_Shore | -7.23E-03 | 3.62E-05 |
| cg25143508 | PRELP;PRELP | 5'UTR;5'UTR | NA | -8.66E-03 | 6.97E-16 |
| cg25143652 | PTK6;PTK6 | 1stExon;5'UTR | Island | -7.55E-03 | 1.16E-08 |
| cg25148589 | GRIA2;GRIA2;GRIA2 | 1stExon;5'UTR;5'UT | N_Shore | 1.04E-02 | 2.32E-08 |
| cg25150721 | CDC14A;CDC14A;CD | Body;Body;Body | NA | -1.52E-02 | 2.88E-14 |
| cg25151916 | YBX2 | Body | Island | 6.07E-03 | 1.54E-05 |
| cg25151919 | ZNF502;ZNF502;ZNF | 5'UTR;5'UTR;5'UTR; | Island | 2.41E-02 | 7.35E-10 |
| cg25165880 | ACOT7 | TSS1500 | S_Shore | -1.46E-02 | 2.97E-12 |
| cg25174438 | NA | NA | Island | 1.09E-02 | 7.02E-05 |
| cg25191850 | KCNK1 | TSS200 | Island | 2.00E-02 | 1.81E-05 |
| cg25196715 | ABCA4 | Body | NA | -1.78E-02 | 1.20E-07 |

| | | | | | |
|---|---|---|---|---|---|
| cg25201047 | NA | NA | Island | 8.49E-03 | 3.54E-06 |
| cg25207248 | NA | NA | Island | 9.62E-03 | 8.45E-05 |
| cg25220781 | ARHGEF19;ARHGEF: | 5'UTR;1stExon | NA | -6.04E-03 | 1.17E-05 |
| cg25234732 | LOC389493 | 1stExon | Island | 1.75E-02 | 1.28E-08 |
| cg25235397 | CCDC50;CCDC50;CC | 5'UTR;1stExon;5'UT | Island | 4.71E-02 | 5.01E-07 |
| cg25236230 | GPR176;GPR176 | 5'UTR;1stExon | Island | 1.55E-02 | 1.37E-09 |
| cg25237016 | SPTBN4 | Body | Island | 8.60E-03 | 1.94E-07 |
| cg25246431 | PARVA | TSS1500 | N_Shore | 1.65E-02 | 1.60E-05 |
| cg25250013 | KANK2;KANK2 | Body;Body | N_Shore | -8.51E-03 | 3.51E-10 |
| cg25254170 | MTCH1 | TSS1500 | S_Shore | -9.77E-03 | 2.66E-05 |
| cg25256723 | F5 | TSS200 | NA | -1.73E-02 | 7.34E-22 |
| cg25264101 | PODNL1;DCAF15;PC | TSS200;Body;TSS20 | S_Shore | -8.28E-03 | 1.10E-09 |
| cg25267072 | NA | NA | Island | 1.87E-02 | 1.22E-08 |
| cg25268718 | PSME1;PSME1 | TSS1500;TSS1500 | N_Shore | -7.81E-03 | 9.06E-09 |
| cg25271525 | NA | NA | NA | -1.09E-02 | 1.35E-08 |
| cg25278941 | NA | NA | NA | -1.37E-02 | 1.09E-10 |
| cg25283626 | NA | NA | Island | 1.23E-02 | 8.16E-06 |
| cg25292309 | NA | NA | NA | -9.06E-03 | 2.23E-05 |
| cg25308086 | PRDM16;PRDM16 | Body;Body | N_Shore | -1.23E-02 | 1.72E-08 |
| cg25308508 | NA | NA | Island | 1.17E-02 | 2.09E-05 |
| cg25311470 | NRCAM;NRCAM | 5'UTR;5'UTR | NA | -1.63E-02 | 2.17E-17 |
| cg25311764 | GCNT3 | 5'UTR | NA | -1.17E-02 | 1.62E-08 |
| cg25316339 | ANKRD34B | TSS200 | Island | 2.22E-02 | 2.62E-12 |
| cg25317585 | FGF14;FGF14 | Body;1stExon | Island | 2.80E-02 | 7.57E-06 |
| cg25320169 | NRP2;NRP2;NRP2;N | 3'UTR;Body;3'UTR;E | NA | -4.42E-03 | 8.00E-05 |
| cg25340674 | PDE4DIP | Body | S_Shore | -1.29E-02 | 8.62E-25 |
| cg25342125 | CCDC50;UTS2D;CCD | TSS1500;5'UTR;TSS: | N_Shore | -1.24E-02 | 2.66E-10 |
| cg25343280 | NA | NA | N_Shore | -9.87E-03 | 6.29E-06 |
| cg25349939 | GTDC1;GTDC1;GTD( | Body;Body;Body | NA | -1.42E-02 | 2.69E-09 |
| cg25351606 | NA | NA | Island | 1.85E-02 | 9.52E-10 |
| cg25352836 | TSSK6;TSSK6 | 3'UTR;1stExon | Island | 1.67E-02 | 1.87E-05 |
| cg25355316 | NA | NA | S_Shore | 8.14E-03 | 2.47E-06 |
| cg25357706 | ADCY5 | Body | NA | -5.75E-03 | 2.28E-05 |
| cg25360385 | GALNT6 | TSS1500 | S_Shore | -2.05E-02 | 2.41E-09 |
| cg25362648 | DAO | TSS200 | NA | -1.04E-02 | 8.12E-06 |
| cg25369553 | NA | NA | S_Shelf | -1.88E-02 | 3.44E-08 |
| cg25371036 | AMOTL1 | TSS1500 | N_Shore | -1.74E-02 | 7.48E-28 |
| cg25371564 | NA | NA | N_Shelf | -7.77E-03 | 9.17E-08 |
| cg25375916 | SLC33A1 | Body | N_Shelf | -9.44E-03 | 1.79E-06 |
| cg25376310 | ZDHHC14;ZDHHC14 | Body;Body | NA | -6.03E-03 | 8.95E-06 |
| cg25382400 | SOX2OT | Body | S_Shore | 8.67E-03 | 6.65E-08 |
| cg25385733 | 04-mar | TSS1500 | Island | 7.77E-03 | 1.12E-07 |
| cg25391162 | FOXD4 | 1stExon | Island | 7.56E-03 | 5.30E-14 |
| cg25393494 | PLD6 | TSS1500 | Island | -3.28E-02 | 3.79E-16 |
| cg25397426 | CTCFL | 5'UTR | Island | -9.86E-03 | 3.32E-05 |
| cg25400422 | GPS1;RFNG;GPS1 | TSS1500;Body;TSS1 | Island | -6.91E-03 | 2.49E-05 |
| cg25403721 | NA | NA | Island | 1.49E-02 | 8.10E-05 |
| cg25406989 | FBN1 | Body | N_Shore | -1.15E-02 | 1.20E-06 |
| cg25408950 | NEK7 | 3'UTR | NA | -1.88E-02 | 2.08E-12 |
| cg25410668 | RPA2 | TSS1500 | S_Shore | 1.03E-02 | 7.56E-07 |

| | | | | | |
|---|---|---|---|---|---|
| cg25425705 | NA | NA | NA | -8.32E-03 | 5.44E-10 |
| cg25427880 | NA | NA | Island | 1.08E-02 | 1.80E-13 |
| cg25428494 | HPSE;HPSE;HPSE | Body;Body;Body | N_Shore | -1.44E-02 | 9.85E-10 |
| cg25436157 | C18orf45 | TSS1500 | S_Shore | -1.29E-02 | 2.39E-14 |
| cg25439496 | NA | NA | Island | 1.37E-02 | 6.40E-09 |
| cg25459450 | NA | NA | Island | 7.81E-03 | 1.19E-05 |
| cg25467652 | AGTRAP;AGTRAP;AC | TSS200;TSS200;TSS | Island | -8.20E-03 | 1.23E-06 |
| cg25471715 | C8orf31 | Body | NA | -1.36E-02 | 1.29E-13 |
| cg25478114 | NA | NA | NA | -5.89E-02 | 2.75E-07 |
| cg25491122 | PCDH9;PCDH9 | Body;Body | N_Shelf | -2.62E-02 | 1.43E-16 |
| cg25497250 | GPR120 | 1stExon | Island | 2.05E-02 | 1.42E-05 |
| cg25502093 | C5orf54 | TSS1500 | S_Shore | -1.70E-02 | 3.14E-12 |
| cg25512537 | NA | NA | N_Shore | -9.85E-03 | 1.11E-05 |
| cg25514273 | KLHL1;ATXN8OS | 1stExon;Body | Island | 1.22E-02 | 7.29E-07 |
| cg25517810 | NA | NA | S_Shore | 1.15E-02 | 7.64E-07 |
| cg25540816 | CSNK1A1;CSNK1A1 | Body;Body | N_Shore | -1.43E-02 | 4.84E-05 |
| cg25544461 | GTF2IRD1;GTF2IRD1 | 5'UTR;5'UTR | N_Shore | -1.71E-02 | 8.57E-16 |
| cg25547580 | ONECUT2 | 1stExon | Island | 1.88E-02 | 2.48E-05 |
| cg25549459 | POU3F3 | TSS1500 | Island | 1.01E-02 | 3.89E-05 |
| cg25556225 | NA | NA | NA | -1.95E-02 | 5.50E-05 |
| cg25561382 | CRTC1;CRTC1 | Body;Body | S_Shore | -1.52E-02 | 2.77E-28 |
| cg25570486 | NA | NA | Island | -6.57E-03 | 3.31E-07 |
| cg25581347 | NA | NA | NA | -1.14E-02 | 4.79E-12 |
| cg25584930 | AP3B2 | Body | NA | 7.64E-03 | 6.05E-08 |
| cg25588844 | TAF1B | Body | NA | -3.42E-02 | 1.72E-10 |
| cg25588852 | MREG | Body | N_Shore | -1.53E-02 | 2.10E-06 |
| cg25590826 | CCDC33 | Body | NA | 7.49E-03 | 4.29E-05 |
| cg25603883 | SHC1;SHC1;SHC1;SH | 5'UTR;5'UTR;TSS15 | N_Shore | -1.19E-02 | 1.14E-06 |
| cg25607920 | HIVEP3;HIVEP3;HIVE | 1stExon;1stExon;5'U | S_Shore | -2.38E-02 | 1.22E-08 |
| cg25608626 | DSCAML1 | Body | NA | -8.47E-03 | 1.00E-08 |
| cg25644224 | PTPRN2;PTPRN2;PTI | Body;Body;Body | N_Shelf | -7.72E-03 | 1.02E-07 |
| cg25644556 | MIR196B | TSS1500 | Island | 2.22E-02 | 6.27E-07 |
| cg25645064 | NA | NA | NA | 1.99E-02 | 7.00E-12 |
| cg25648746 | RGS7BP | Body | S_Shore | 9.75E-03 | 7.32E-06 |
| cg25657261 | PCDHGA2;PCDHGA3 | Body;1stExon;1stEx | Island | 6.93E-03 | 1.43E-06 |
| cg25657713 | NA | NA | Island | 1.12E-02 | 1.60E-06 |
| cg25665697 | LCTL | TSS200 | NA | -6.73E-03 | 5.48E-06 |
| cg25677261 | PCSK6;PCSK6;PCSK6 | Body;Body;Body;Bo | NA | -4.40E-02 | 3.27E-10 |
| cg25678088 | DLX6AS | Body | S_Shore | 9.39E-03 | 8.97E-08 |
| cg25682299 | POU4F1 | TSS200 | Island | 1.00E-02 | 3.39E-05 |
| cg25689193 | NA | NA | N_Shore | 9.64E-03 | 9.64E-06 |
| cg25694915 | NOSTRIN;NOSTRIN;I | 5'UTR;TSS200;TSS2( | NA | -2.94E-02 | 5.14E-36 |
| cg25711003 | PHLDB2;PLCXD2 | 5'UTR;Body | NA | -3.07E-02 | 6.08E-20 |
| cg25712005 | ELN;ELN;ELN;ELN;EL | Body;Body;Body;Bo | NA | -1.54E-02 | 8.12E-08 |
| cg25741259 | RFX2;RFX2 | Body;Body | N_Shore | -6.54E-03 | 6.34E-05 |
| cg25750389 | PITX2;PITX2;PITX2;P | 1stExon;5'UTR;Body | Island | 8.62E-03 | 2.48E-06 |
| cg25754958 | PTPN7 | TSS1500 | NA | -8.57E-03 | 1.41E-05 |
| cg25757869 | MYOZ1;MYOZ1 | 1stExon;5'UTR | NA | -8.47E-03 | 2.95E-10 |
| cg25763393 | ZNF578;ZNF578 | 1stExon;5'UTR | Island | 2.66E-02 | 1.31E-09 |
| cg25773262 | BCAN;BCAN | Body;Body | Island | 1.02E-02 | 4.81E-06 |

| | | | | | |
|---|---|---|---|---|---|
| cg25778262 | CPM;CPM;CPM | TSS1500;TSS1500;5 | Island | 1.18E-02 | 6.02E-10 |
| cg25793051 | NA | NA | NA | -1.45E-02 | 5.64E-11 |
| cg25802093 | SPAG6;SPAG6 | TSS1500;TSS1500 | Island | 9.15E-03 | 7.57E-06 |
| cg25809905 | ITGA2B | TSS1500 | NA | -1.61E-02 | 5.07E-14 |
| cg25818109 | MASP1 | 3'UTR | NA | -1.08E-02 | 3.58E-06 |
| cg25818402 | ZNF273;ZNF273 | TSS200;TSS200 | NA | 1.58E-02 | 1.61E-08 |
| cg25819026 | SPTBN4;SPTBN4 | Body;Body | Island | 1.37E-02 | 2.20E-06 |
| cg25824236 | SPAG16 | Body | NA | -2.29E-02 | 1.62E-05 |
| cg25838150 | NA | NA | NA | -1.57E-02 | 3.01E-09 |
| cg25840237 | NA | NA | Island | 1.05E-02 | 1.31E-05 |
| cg25843346 | VARS | Body | N_Shore | -7.70E-03 | 1.01E-07 |
| cg25865120 | DUT;DUT;DUT | Body;Body;Body | S_Shore | -1.38E-02 | 7.33E-12 |
| cg25865467 | PDZRN3 | Body | Island | 9.56E-03 | 3.82E-05 |
| cg25866895 | GSTP1 | TSS1500 | N_Shore | -2.04E-02 | 3.04E-11 |
| cg25879062 | NA | NA | NA | -7.96E-03 | 1.48E-05 |
| cg25886479 | LOC441601 | Body | Island | 2.64E-02 | 9.15E-07 |
| cg25903363 | HR;HR | 3'UTR;3'UTR | NA | -9.34E-03 | 3.72E-09 |
| cg25907138 | NA | NA | NA | -1.08E-02 | 6.58E-09 |
| cg25907743 | LIPN | TSS1500 | NA | -1.23E-02 | 4.68E-08 |
| cg25909064 | OAF | Body | S_Shore | -5.50E-03 | 8.57E-05 |
| cg25922624 | TRIM2 | 5'UTR | S_Shore | -1.16E-02 | 1.30E-10 |
| cg25930644 | MYH10 | 5'UTR | N_Shore | -1.82E-02 | 9.32E-19 |
| cg25932761 | CUX2 | Body | S_Shelf | -6.77E-03 | 2.76E-07 |
| cg25934371 | SYNPR;SYNPR | Body;TSS1500 | NA | -1.48E-02 | 1.09E-11 |
| cg25936177 | NA | NA | Island | 6.67E-03 | 4.28E-05 |
| cg25950235 | MIR9-3 | TSS1500 | Island | 1.35E-02 | 2.75E-07 |
| cg25960854 | SBNO2 | 5'UTR | S_Shore | 8.45E-03 | 1.43E-07 |
| cg25968437 | NA | NA | NA | -1.31E-02 | 7.37E-10 |
| cg25974380 | NA | NA | NA | -1.58E-02 | 5.51E-06 |
| cg25975805 | NA | NA | Island | 6.21E-03 | 6.06E-05 |
| cg25980484 | KIAA1949;KIAA1949 | Body;1stExon | N_Shore | -9.55E-03 | 1.13E-08 |
| cg25994988 | UBASH3B | Body | NA | -1.39E-02 | 4.33E-22 |
| cg25998745 | NA | NA | NA | -1.23E-02 | 1.87E-12 |
| cg26004710 | PTPN6;PTPN6;PTPN | Body;Body;Body | NA | -8.35E-03 | 6.45E-06 |
| cg26023709 | MRPL43;SEMA4G;M | Body;3'UTR;Body;B | N_Shore | -1.07E-02 | 9.61E-12 |
| cg26023748 | NA | NA | NA | -1.18E-02 | 7.70E-10 |
| cg26025224 | MRPS11;MRPS11 | Body;Body | NA | -1.57E-02 | 6.43E-19 |
| cg26037936 | NA | NA | NA | -7.34E-03 | 1.16E-05 |
| cg26038582 | HIVEP3;HIVEP3;HIVE | 1stExon;1stExon;5'U | S_Shore | -2.56E-02 | 7.97E-08 |
| cg26039298 | KIAA1026;KIAA1026 | Body;Body;Body;Bo | NA | -4.58E-03 | 1.99E-06 |
| cg26049379 | TMEM82 | Body | N_Shore | -7.69E-03 | 1.23E-05 |
| cg26050864 | G0S2 | 3'UTR | S_Shore | 6.27E-03 | 9.72E-05 |
| cg26056703 | NA | NA | Island | 1.59E-02 | 1.14E-15 |
| cg26058502 | LASS2;LASS2 | 5'UTR;5'UTR | N_Shore | -7.80E-03 | 4.55E-10 |
| cg26060489 | NA | NA | Island | 6.20E-03 | 1.00E-06 |
| cg26088662 | ADRA1B | Body | Island | 9.19E-03 | 1.03E-06 |
| cg26094554 | SRCIN1 | Body | Island | 7.57E-03 | 9.14E-06 |
| cg26122129 | RBPMS;RBPMS;RBPI | TSS1500;TSS1500;T | N_Shore | -8.72E-03 | 4.26E-07 |
| cg26122710 | NA | NA | NA | -6.66E-03 | 1.74E-05 |
| cg26140475 | NA | NA | NA | -1.01E-02 | 2.93E-08 |

| | | | | | |
|---|---|---|---|---|---|
| cg26140749 | NA | NA | Island | -1.19E-02 | 8.20E-08 |
| cg26153045 | ELN;ELN;ELN;ELN;EL | TSS1500;TSS1500;T | NA | -1.24E-02 | 2.58E-17 |
| cg26157309 | NA | NA | S_Shelf | -7.41E-03 | 7.20E-05 |
| cg26158279 | NINL | 5'UTR | Island | 1.20E-02 | 1.01E-05 |
| cg26158959 | SYT14;SYT14;SYT14; | TSS1500;TSS1500;T | N_Shore | 9.38E-03 | 3.42E-08 |
| cg26160218 | NA | NA | S_Shore | -1.12E-02 | 7.42E-05 |
| cg26160416 | NA | NA | NA | -8.41E-03 | 7.23E-05 |
| cg26183127 | NA | NA | N_Shore | -8.58E-03 | 2.34E-10 |
| cg26195356 | NA | NA | S_Shelf | 2.46E-02 | 6.51E-11 |
| cg26206396 | FOXG1 | TSS1500 | Island | 8.92E-03 | 7.60E-05 |
| cg26210267 | ATP5I | TSS1500 | S_Shore | -1.41E-02 | 5.46E-17 |
| cg26211349 | HRNBP3 | 5'UTR | NA | -9.98E-03 | 1.27E-07 |
| cg26212163 | LOC134466 | TSS200 | S_Shore | 1.32E-02 | 5.72E-07 |
| cg26220018 | NA | NA | Island | 2.01E-02 | 8.45E-06 |
| cg26229990 | ADCY4 | Body | N_Shore | -1.35E-02 | 1.02E-08 |
| cg26231761 | AJAP1;AJAP1 | Body;Body | NA | -7.16E-03 | 2.25E-05 |
| cg26233209 | ATG12;AP3S1 | TSS1500;Body | S_Shore | -1.34E-02 | 9.97E-14 |
| cg26234644 | TMEM220 | TSS1500 | S_Shore | -2.33E-02 | 2.12E-12 |
| cg26236972 | NA | NA | NA | -1.34E-02 | 1.40E-07 |
| cg26237066 | FRS3 | Body | N_Shelf | -1.11E-02 | 1.22E-08 |
| cg26246342 | BRD2;BRD2 | Body;Body | NA | -9.77E-03 | 8.15E-12 |
| cg26263766 | ZNF544 | TSS1500 | N_Shore | 2.68E-02 | 6.83E-08 |
| cg26264318 | BRD2;BRD2 | Body;Body | S_Shore | -1.20E-02 | 7.29E-07 |
| cg26267561 | OXT | TSS200 | Island | -8.97E-03 | 5.47E-06 |
| cg26267678 | KCNF1;KCNF1 | 5'UTR;1stExon | Island | 8.64E-03 | 3.56E-05 |
| cg26275799 | NA | NA | N_Shelf | -1.43E-02 | 9.44E-07 |
| cg26276120 | TPI1;TPI1;TPI1 | Body;Body;Body | S_Shore | -8.58E-03 | 1.07E-06 |
| cg26280713 | THBS2 | 5'UTR | NA | 1.27E-02 | 8.19E-09 |
| cg26282761 | C10orf90 | Body | N_Shore | -8.94E-03 | 8.90E-05 |
| cg26290632 | CALB1 | 1stExon | NA | 1.93E-02 | 1.25E-14 |
| cg26292150 | ZDHHC19 | Body | Island | 1.74E-02 | 2.01E-08 |
| cg26298273 | SLC22A23;SLC22A23 | TSS200;Body | NA | -7.55E-03 | 1.70E-05 |
| cg26306435 | CHST15;CHST15 | 1stExon;5'UTR | S_Shore | -1.30E-02 | 1.22E-06 |
| cg26307359 | SEZ6;SEZ6 | Body;Body | Island | 1.71E-02 | 2.53E-06 |
| cg26316423 | IL2RA;IL2RA | 1stExon;5'UTR | NA | -8.84E-03 | 6.34E-09 |
| cg26316599 | ATP6V0E1 | 3'UTR | NA | -9.90E-03 | 2.38E-16 |
| cg26320830 | MARCH1;MARCH1 | 1stExon;5'UTR | Island | 1.23E-02 | 5.67E-06 |
| cg26325867 | NA | NA | S_Shelf | -9.36E-03 | 2.67E-14 |
| cg26328951 | SNHG12;SNORD99;S | Body;TSS1500;TSS2 | N_Shore | -1.27E-02 | 1.66E-11 |
| cg26331343 | NA | NA | S_Shore | -1.43E-02 | 1.04E-11 |
| cg26337070 | ATOH8 | Body | NA | -1.38E-02 | 3.29E-08 |
| cg26345105 | NA | NA | S_Shore | 1.07E-02 | 5.34E-06 |
| cg26345203 | NA | NA | NA | -1.49E-02 | 1.36E-05 |
| cg26345916 | NA | NA | NA | -1.01E-02 | 7.58E-11 |
| cg26350754 | HLA-DPB1 | 1stExon | NA | -1.65E-02 | 7.26E-11 |
| cg26351416 | SH3RF2 | Body | S_Shelf | -1.45E-02 | 3.11E-08 |
| cg26354128 | KIAA1409 | 5'UTR | Island | 2.06E-02 | 4.18E-09 |
| cg26355573 | LOC642597 | TSS200 | Island | 1.57E-02 | 2.51E-08 |
| cg26365254 | NA | NA | S_Shelf | -1.66E-02 | 1.17E-13 |
| cg26365690 | NMBR | TSS200 | Island | 2.33E-02 | 1.93E-05 |

| | | | | | |
|---|---|---|---|---|---|
| cg26375057 | ZIC1;ZIC1 | 1stExon;5'UTR | Island | 7.15E-03 | 5.45E-05 |
| cg26390598 | B3GALT5;B3GALT5;I | 5'UTR;5'UTR;5'UTR; | NA | -2.67E-02 | 1.21E-13 |
| cg26394191 | MYLK;MYLK;MYLK;N | Body;Body;Body;Bo | NA | -8.44E-03 | 1.60E-07 |
| cg26395331 | NA | NA | NA | -1.45E-02 | 4.49E-10 |
| cg26403171 | NA | NA | NA | -1.51E-02 | 1.42E-12 |
| cg26403608 | LOC284009;METT1C | TSS1500;3'UTR | NA | -1.56E-02 | 5.94E-12 |
| cg26413683 | NA | NA | Island | 5.35E-03 | 7.41E-05 |
| cg26413855 | NA | NA | NA | -1.09E-02 | 5.09E-05 |
| cg26417912 | SLAMF9;SLAMF9;SL | 3'UTR;Body;Body | NA | -7.34E-03 | 8.76E-08 |
| cg26424147 | NA | NA | S_Shore | -9.66E-03 | 9.80E-09 |
| cg26427498 | NA | NA | NA | -1.35E-02 | 4.27E-05 |
| cg26438705 | IFT140 | 5'UTR | Island | 2.47E-02 | 9.66E-05 |
| cg26445608 | CPXM1 | Body | N_Shore | -1.33E-02 | 1.77E-07 |
| cg26450750 | NA | NA | S_Shelf | -1.37E-02 | 1.43E-06 |
| cg26468891 | PTPRJ;PTPRJ | Body;Body | NA | -1.01E-02 | 2.91E-07 |
| cg26469387 | OTUD7A | Body | NA | -9.94E-03 | 1.96E-06 |
| cg26476852 | HOXA9 | 1stExon | Island | 1.39E-02 | 4.33E-05 |
| cg26483332 | COMTD1 | 3'UTR | N_Shore | -1.50E-02 | 2.49E-14 |
| cg26484813 | AHNAK;AHNAK | 5'UTR;5'UTR | N_Shore | -8.97E-03 | 4.05E-05 |
| cg26485937 | PCDHGA4;PCDHGA9 | Body;Body;Body;Bo | Island | 1.21E-02 | 9.25E-08 |
| cg26497342 | PROZ | TSS200 | NA | -5.38E-03 | 7.42E-05 |
| cg26501046 | DIRC3 | Body | NA | -8.36E-03 | 3.56E-08 |
| cg26503073 | NA | NA | Island | 9.91E-03 | 5.18E-06 |
| cg26506932 | NA | NA | NA | -1.08E-02 | 7.15E-11 |
| cg26513192 | ARHGAP29 | TSS200 | S_Shore | 1.35E-02 | 1.48E-09 |
| cg26517714 | TLX3 | TSS200 | Island | 9.77E-03 | 6.63E-06 |
| cg26529044 | C13orf30 | TSS1500 | NA | -1.07E-02 | 5.39E-06 |
| cg26530942 | ARMC9 | Body | N_Shelf | -1.63E-02 | 2.14E-19 |
| cg26531286 | FARP1;FARP1 | 5'UTR;5'UTR | S_Shelf | -1.26E-02 | 4.87E-05 |
| cg26534677 | CCDC25 | TSS1500 | N_Shore | -1.22E-02 | 4.65E-07 |
| cg26542792 | LOC283404 | TSS1500 | NA | -5.95E-03 | 3.45E-07 |
| cg26543112 | NA | NA | NA | -1.69E-02 | 5.35E-12 |
| cg26547924 | KCNK2;KCNK2;KCNK | Body;Body;Body | S_Shore | 1.85E-02 | 6.88E-05 |
| cg26553501 | NA | NA | S_Shore | -2.47E-02 | 7.38E-09 |
| cg26554353 | NA | NA | N_Shelf | -9.69E-03 | 1.66E-05 |
| cg26555966 | IGDCC3 | Body | S_Shore | -8.16E-03 | 4.16E-08 |
| cg26567423 | FLJ12825 | Body | Island | 1.84E-02 | 2.82E-07 |
| cg26569002 | PCYOX1L | Body | NA | -5.70E-03 | 1.41E-05 |
| cg26571127 | MAST1 | Body | Island | 6.45E-03 | 3.34E-05 |
| cg26578682 | IRX2;IRX2 | Body;Body | Island | 9.79E-03 | 5.20E-10 |
| cg26578856 | ZNF69 | TSS200 | N_Shore | 2.01E-02 | 9.91E-10 |
| cg26580095 | BTBD8 | TSS1500 | N_Shore | -7.54E-03 | 1.24E-07 |
| cg26580869 | HCN2 | Body | Island | -1.27E-02 | 3.31E-05 |
| cg26581729 | NPDC1 | Body | Island | -7.94E-03 | 1.92E-08 |
| cg26588812 | GPRC5C;GPRC5C | TSS1500;Body | Island | -6.00E-03 | 3.19E-05 |
| cg26595643 | VAX1;VAX1 | TSS1500;TSS1500 | Island | 1.60E-02 | 5.50E-07 |
| cg26608718 | AKAP8L | TSS1500 | S_Shore | -1.69E-02 | 5.36E-13 |
| cg26610808 | BLOC1S2;BLOC1S2 | TSS1500;TSS1500 | S_Shore | -1.04E-02 | 4.67E-06 |
| cg26614073 | SCAP | TSS1500 | S_Shore | -1.42E-02 | 5.29E-16 |
| cg26614154 | C1orf92 | Body | Island | 1.25E-02 | 3.42E-07 |

| | | | | | |
|---|---|---|---|---|---|
| cg26619624 | SHFM1 | TSS1500 | S_Shore | -1.58E-02 | 2.01E-06 |
| cg26643914 | NA | NA | NA | -9.59E-03 | 3.11E-06 |
| cg26645655 | NA | NA | NA | -1.92E-02 | 3.49E-06 |
| cg26647566 | PRSS12 | 3'UTR | S_Shelf | -1.92E-02 | 7.51E-15 |
| cg26648818 | TOX3;TOX3 | TSS200;TSS1500 | S_Shore | 9.22E-03 | 8.43E-06 |
| cg26649384 | RPRM;RPRM | 3'UTR;1stExon | Island | 1.36E-02 | 6.47E-05 |
| cg26675682 | RFPL1S;RFPL1;RFPL1 | Body;5'UTR;1stExor | NA | -1.08E-02 | 6.34E-07 |
| cg26682068 | NA | NA | Island | 7.88E-03 | 6.81E-08 |
| cg26685246 | TAP2;TAP2 | Body;Body | N_Shore | -1.40E-02 | 2.69E-12 |
| cg26685941 | ABCC4;ABCC4 | Body;Body | N_Shore | -1.55E-02 | 4.52E-13 |
| cg26697605 | NA | NA | NA | -8.67E-03 | 3.07E-05 |
| cg26700320 | FAM19A5 | Body | NA | -8.07E-03 | 4.43E-05 |
| cg26701785 | SYNJ2 | Body | NA | -2.06E-02 | 2.57E-07 |
| cg26706003 | AVPR1A;AVPR1A | 1stExon;5'UTR | S_Shore | -1.26E-02 | 6.04E-05 |
| cg26712821 | DVWA | Body | N_Shelf | -1.27E-02 | 3.65E-08 |
| cg26718511 | SCGN;SCGN | 1stExon;5'UTR | Island | 1.23E-02 | 1.16E-08 |
| cg26721803 | TUBB | Body | S_Shore | -1.40E-02 | 1.79E-19 |
| cg26725076 | EP400NL | Body | S_Shore | -1.64E-02 | 1.36E-24 |
| cg26728895 | TMTC2 | Body | S_Shore | -1.73E-02 | 6.26E-06 |
| cg26729380 | TNF | 1stExon | NA | -1.88E-02 | 2.73E-09 |
| cg26729956 | NA | NA | S_Shelf | -1.03E-02 | 3.64E-05 |
| cg26734350 | GPR56;GPR56;GPR5 | TSS1500;TSS1500;T | NA | -1.54E-02 | 1.31E-07 |
| cg26739149 | NA | NA | NA | -2.27E-02 | 3.35E-08 |
| cg26748477 | NA | NA | N_Shelf | -1.28E-02 | 1.65E-14 |
| cg26775866 | PTTG1 | 5'UTR | S_Shore | -1.96E-02 | 1.70E-12 |
| cg26785800 | NA | NA | S_Shore | -9.36E-03 | 6.60E-15 |
| cg26801613 | LMO4 | TSS1500 | N_Shore | -2.08E-02 | 2.98E-17 |
| cg26801943 | SEMA4F | TSS1500 | N_Shore | -1.43E-02 | 3.45E-08 |
| cg26803305 | SLC14A1;SLC14A1;S | 5'UTR;5'UTR;5'UTR | NA | -2.04E-02 | 6.94E-08 |
| cg26809372 | LAMC1 | Body | NA | -9.42E-03 | 2.27E-08 |
| cg26844213 | NEDD9;NEDD9;NED | TSS1500;Body;TSS1 | NA | -7.21E-03 | 2.59E-06 |
| cg26844246 | TLX3 | TSS200 | Island | 1.28E-02 | 1.36E-07 |
| cg26856607 | NCAN | 5'UTR | S_Shelf | 9.75E-03 | 7.11E-12 |
| cg26864395 | RUNX3 | Body | S_Shore | -4.56E-03 | 5.78E-07 |
| cg26871350 | F2RL2;IQGAP2 | TSS1500;Body | NA | -2.28E-02 | 8.34E-15 |
| cg26881761 | NA | NA | S_Shore | -1.09E-02 | 4.24E-10 |
| cg26885220 | DNAJC6 | Body | Island | 3.30E-02 | 1.02E-09 |
| cg26888530 | AFAP1;AFAP1 | TSS200;TSS200 | Island | 2.21E-02 | 4.15E-09 |
| cg26890189 | SLC8A2 | Body | Island | 3.13E-02 | 8.48E-14 |
| cg26894354 | FMOD | 3'UTR | NA | -9.36E-03 | 1.61E-09 |
| cg26902127 | NA | NA | Island | -2.41E-02 | 5.64E-08 |
| cg26904049 | DUSP27 | Body | S_Shore | -8.24E-03 | 4.58E-07 |
| cg26905261 | ST18 | TSS200 | NA | -1.20E-02 | 8.73E-09 |
| cg26914205 | ADNP2 | TSS1500 | NA | -1.72E-02 | 2.35E-12 |
| cg26918808 | NA | NA | NA | -9.43E-03 | 5.15E-05 |
| cg26921969 | NA | NA | NA | 9.56E-03 | 2.09E-07 |
| cg26931990 | IFT140 | 5'UTR | Island | 1.31E-02 | 6.50E-07 |
| cg26935102 | POLR3GL;ANKRD34/ | TSS1500;5'UTR | S_Shore | -1.26E-02 | 8.26E-17 |
| cg26938597 | NA | NA | Island | 1.82E-02 | 1.04E-07 |
| cg26949694 | BDNF;BDNF;BDNF;B | Body;5'UTR;TSS150 | Island | 1.38E-02 | 2.99E-16 |

| | | | | | |
|---|---|---|---|---|---|
| cg26953232 | BRD2;BRD2 | Body;Body | S_Shore | -1.54E-02 | 1.30E-11 |
| cg26954138 | C11orf60;C11orf60 | TSS1500;TSS1500 | NA | -1.88E-02 | 2.96E-09 |
| cg26954174 | NOD2 | TSS1500 | NA | -9.17E-03 | 1.44E-05 |
| cg26956007 | VAT1L | Body | NA | -1.42E-02 | 2.03E-11 |
| cg26983177 | NA | NA | Island | 1.55E-02 | 8.46E-06 |
| cg26985711 | DCN;DCN;DCN;DCN; | 1stExon;1stExon;Bo | NA | -1.37E-02 | 1.10E-08 |
| cg26993105 | ACTR3B;ACTR3B | Body;Body | S_Shore | -1.40E-02 | 1.11E-10 |
| cg27004669 | NA | NA | Island | -6.19E-03 | 2.11E-05 |
| cg27018380 | CHGA | Body | Island | 2.31E-02 | 4.51E-09 |
| cg27018424 | HSD17B8 | Body | S_Shore | -5.41E-03 | 3.83E-05 |
| cg27020216 | SGEF | Body | Island | 7.79E-03 | 1.20E-06 |
| cg27021181 | NET1;NET1 | Body;TSS200 | N_Shore | -1.26E-02 | 3.19E-11 |
| cg27042278 | GPR68 | TSS1500 | S_Shore | -6.27E-03 | 1.36E-06 |
| cg27044841 | NA | NA | Island | 1.93E-02 | 5.08E-05 |
| cg27045264 | NA | NA | S_Shelf | -8.98E-03 | 1.35E-06 |
| cg27050153 | NA | NA | Island | 6.60E-03 | 4.12E-06 |
| cg27052113 | SLC8A2 | Body | Island | 1.96E-02 | 3.06E-06 |
| cg27054162 | NA | NA | NA | 9.84E-03 | 3.66E-05 |
| cg27067781 | PRRT1 | 3'UTR | Island | 1.23E-02 | 2.20E-05 |
| cg27068143 | HTR2A;HTR2A | TSS200;TSS200 | NA | -1.84E-02 | 2.82E-16 |
| cg27076669 | CPM;CPM;CPM | TSS1500;TSS1500;5 | Island | 1.23E-02 | 7.00E-09 |
| cg27081230 | SNX20;SNX20;SNX2( | TSS1500;TSS1500;T | NA | -8.45E-03 | 1.29E-06 |
| cg27093871 | NA | NA | NA | -6.31E-03 | 4.39E-05 |
| cg27094244 | SYT7 | Body | Island | 1.13E-02 | 9.13E-05 |
| cg27095222 | NA | NA | NA | -2.69E-02 | 1.41E-06 |
| cg27111678 | CHST8;CHST8;CHST8 | 5'UTR;TSS200;5'UTI | Island | 9.95E-03 | 6.08E-07 |
| cg27113015 | SASH1 | Body | NA | -1.53E-02 | 1.05E-15 |
| cg27121267 | ACRV1;ACRV1;ACRV | Body;Body;Body;Bo | NA | -1.34E-02 | 6.55E-10 |
| cg27130630 | C1RL;LOC283314 | Body;TSS1500 | NA | -9.39E-03 | 7.60E-05 |
| cg27130665 | LRRC4C | TSS1500 | NA | -1.67E-02 | 1.20E-12 |
| cg27134322 | ARHGEF10 | Body | NA | 1.23E-02 | 3.17E-05 |
| cg27151122 | NA | NA | N_Shelf | -6.39E-03 | 1.33E-07 |
| cg27165456 | ELFN1 | 5'UTR | N_Shore | -6.96E-03 | 3.27E-06 |
| cg27166177 | NA | NA | Island | -1.65E-02 | 2.88E-17 |
| cg27167224 | NA | NA | NA | 1.75E-02 | 7.06E-08 |
| cg27171201 | NFIC;NFIC | TSS1500;Body | N_Shore | -1.07E-02 | 4.32E-10 |
| cg27178677 | PLCB1;PLCB1 | Body;3'UTR | NA | -1.71E-02 | 1.48E-15 |
| cg27182923 | ADCY5 | Body | NA | -5.76E-03 | 5.82E-06 |
| cg27187555 | PRDM16;PRDM16 | Body;Body | NA | -6.03E-03 | 5.74E-05 |
| cg27198485 | CHST8;CHST8;CHST8 | 5'UTR;TSS200;5'UTI | N_Shore | 9.72E-03 | 4.88E-13 |
| cg27199827 | DTD1 | TSS1500 | N_Shore | -1.44E-02 | 8.61E-08 |
| cg27209578 | NA | NA | Island | 6.91E-03 | 4.60E-05 |
| cg27209729 | NRXN2;NRXN2 | Body;Body | S_Shore | -1.47E-02 | 5.15E-11 |
| cg27210390 | TOM1L1 | Body | S_Shore | -8.74E-03 | 1.04E-06 |
| cg27211408 | MAD1L1;MAD1L1;M | Body;Body;Body | Island | -8.48E-03 | 4.47E-05 |
| cg27213509 | EVX2 | Body | Island | 1.16E-02 | 1.30E-09 |
| cg27215033 | NA | NA | Island | 1.10E-02 | 1.46E-10 |
| cg27217742 | RGS12;RGS12 | Body;Body | Island | 5.35E-03 | 4.53E-05 |
| cg27228433 | NA | NA | Island | 2.29E-02 | 3.21E-08 |
| cg27230882 | NA | NA | NA | -5.30E-03 | 7.80E-06 |

| | | | | | |
|---|---|---|---|---|---|
| cg27232482 | DARC;DARC | TSS1500;TSS1500 | NA | -4.57E-03 | 2.52E-05 |
| cg27237706 | NA | NA | NA | 4.87E-03 | 7.73E-05 |
| cg27238547 | NA | NA | N_Shelf | -8.67E-03 | 2.24E-07 |
| cg27257955 | SLC44A4 | Body | S_Shelf | -1.03E-02 | 6.02E-09 |
| cg27262717 | TNNC2 | Body | Island | 1.39E-02 | 3.63E-07 |
| cg27263998 | IGF2;INS-IGF2;INS-I( | TSS200;Body;Body | NA | -9.51E-03 | 1.67E-05 |
| cg27275553 | SH2B2 | Body | N_Shelf | -1.06E-02 | 2.75E-10 |
| cg27286011 | INF2;INF2;INF2 | 5'UTR;5'UTR;5'UTR | N_Shore | -9.51E-03 | 4.96E-08 |
| cg27290215 | ABLIM1;ABLIM1;ABI | Body;Body;Body | S_Shore | -2.55E-02 | 4.24E-28 |
| cg27292835 | RASSF3 | Body | NA | -9.98E-03 | 3.26E-05 |
| cg27304332 | SORCS2 | Body | N_Shelf | -8.50E-03 | 3.02E-05 |
| cg27305357 | PCDHGA4;PCDHGA6 | Body;Body;Body;Bo | Island | 6.07E-03 | 7.76E-05 |
| cg27312979 | SORBS1;SORBS1;SO | Body;Body;Body;Bo | NA | -3.13E-02 | 3.04E-24 |
| cg27314569 | BSX | TSS200 | Island | 1.26E-02 | 9.55E-11 |
| cg27314669 | NTRK1;INSRR | Body;Body | S_Shelf | -1.59E-02 | 1.45E-15 |
| cg27319536 | PPP2R2B;PPP2R2B;I | 5'UTR;Body;5'UTR;E | S_Shelf | -1.19E-02 | 4.98E-05 |
| cg27320127 | KCNK12 | TSS1500 | Island | 1.13E-02 | 7.77E-13 |
| cg27333886 | SAE1;SAE1;SAE1;SAI | 3'UTR;3'UTR;Body;3 | NA | -1.65E-02 | 8.40E-15 |
| cg27343616 | PCSK2;PCSK2 | 5'UTR;1stExon | N_Shore | 5.39E-03 | 9.16E-06 |
| cg27353143 | NA | NA | Island | 1.48E-02 | 2.65E-05 |
| cg27355006 | CTF1;CTF1 | Body;Body | N_Shore | -7.30E-03 | 3.51E-08 |
| cg27361370 | NA | NA | S_Shore | 9.56E-03 | 2.47E-05 |
| cg27367526 | STEAP2;STEAP2;STE | 5'UTR;TSS1500;5'U | S_Shore | -1.22E-02 | 1.48E-10 |
| cg27376136 | NA | NA | N_Shelf | 2.78E-02 | 1.26E-13 |
| cg27379915 | ZDHHC13;ZDHHC13 | Body;5'UTR | S_Shore | -1.42E-02 | 5.66E-10 |
| cg27382910 | PTPRE | 5'UTR | NA | -8.22E-03 | 4.24E-06 |
| cg27386529 | SCAP | TSS1500 | S_Shore | -1.01E-02 | 3.07E-07 |
| cg27389185 | ZNF540 | TSS200 | S_Shore | 1.07E-02 | 1.37E-08 |
| cg27389562 | CEACAM8 | TSS200 | NA | -3.27E-03 | 2.67E-05 |
| cg27392792 | PTPRN2;PTPRN2;PTI | TSS1500;TSS1500;T | Island | 2.69E-02 | 1.04E-11 |
| cg27395200 | BRD2;BRD2 | Body;Body | S_Shore | -1.36E-02 | 6.83E-15 |
| cg27401724 | ACBD4;ACBD4;ACBD | Body;Body;Body;Bo | S_Shelf | -7.18E-03 | 5.77E-08 |
| cg27407147 | LOC254559 | TSS1500 | Island | 1.79E-02 | 1.41E-09 |
| cg27409771 | NA | NA | NA | -8.53E-03 | 4.23E-09 |
| cg27425327 | DRAM1 | TSS1500 | N_Shore | -9.58E-03 | 3.27E-09 |
| cg27428520 | ETNK2 | 3'UTR | NA | -9.28E-03 | 4.04E-08 |
| cg27430293 | NA | NA | N_Shore | -9.82E-03 | 3.54E-06 |
| cg27434368 | CILP2 | Body | Island | 1.25E-02 | 3.91E-14 |
| cg27438128 | ZNF487 | Body | S_Shore | -1.01E-02 | 6.66E-15 |
| cg27442164 | ATP8B2;ATP8B2 | Body;Body | S_Shelf | -8.40E-03 | 4.33E-09 |
| cg27465618 | KCNMA1;KCNMA1;k | Body;Body;Body;Bo | NA | -8.43E-03 | 8.58E-08 |
| cg27467282 | NA | NA | NA | -2.41E-02 | 1.77E-24 |
| cg27482605 | C19orf51 | Body | Island | 1.57E-02 | 4.73E-08 |
| cg27492942 | CISD3;MLLT6 | TSS1500;3'UTR | N_Shore | -8.47E-03 | 1.03E-05 |
| cg27499860 | MAPK15;MAPK15 | 1stExon;5'UTR | Island | 1.12E-02 | 4.88E-08 |
| cg27505273 | SOX14 | TSS200 | Island | 1.30E-02 | 5.94E-05 |
| cg27513574 | SLC34A2;SLC34A2 | 1stExon;5'UTR | Island | 2.60E-02 | 9.46E-05 |
| cg27514333 | SMAD6;SMAD6 | Body;Body | S_Shore | -6.46E-03 | 2.97E-05 |
| cg27531496 | NA | NA | NA | -7.76E-03 | 1.83E-05 |
| cg27536559 | NA | NA | S_Shelf | -1.22E-02 | 1.28E-06 |

| | | | | | |
|---|---|---|---|---|---|
| cg27549208 | URGCP;UBE2D4 | 5'UTR;TSS1500 | N_Shore | -1.95E-02 | 2.66E-05 |
| cg27549720 | HTR6;HTR6 | 5'UTR;1stExon | Island | 1.18E-02 | 6.48E-05 |
| cg27552435 | NA | NA | Island | 6.48E-03 | 9.54E-05 |
| cg27553457 | PPP1R13L;PPP1R13I | Body;Body | Island | 2.31E-02 | 1.50E-11 |
| cg27553971 | UNC13C | Body | NA | -1.09E-02 | 4.97E-07 |
| cg27560367 | DCUN1D2;TMCO3 | Body;TSS1500 | N_Shore | -5.82E-03 | 1.23E-05 |
| cg27563048 | ABHD14B;ABHD14B | TSS200;TSS200;TSS: | N_Shore | -9.96E-03 | 1.22E-05 |
| cg27569300 | SYNM;SYNM | TSS1500;TSS1500 | Island | 2.12E-02 | 8.91E-10 |
| cg27578439 | FAM18B | TSS1500 | N_Shore | -2.11E-02 | 1.43E-14 |
| cg27581047 | SLC35B4 | TSS1500 | S_Shore | -1.46E-02 | 3.92E-05 |
| cg27584762 | CCNH | TSS1500 | S_Shore | -1.42E-02 | 3.51E-14 |
| cg27589809 | CISH;CISH | TSS1500;TSS1500 | S_Shore | -1.58E-02 | 3.25E-19 |
| cg27591349 | DKK1 | 1stExon | Island | 1.47E-02 | 2.26E-08 |
| cg27597853 | NA | NA | NA | -2.69E-02 | 6.80E-07 |
| cg27603796 | CTTNBP2 | Body | N_Shore | 8.24E-03 | 1.62E-06 |
| cg27611263 | THUMPD2;THUMPD | TSS1500;TSS1500 | S_Shore | -1.50E-02 | 1.26E-09 |
| cg27614534 | ALPK2 | TSS1500 | NA | -1.76E-02 | 7.02E-18 |
| cg27622625 | CNGB1 | Body | Island | -7.19E-03 | 3.94E-05 |
| cg27630311 | TBX3;TBX3 | 1stExon;1stExon | Island | 2.27E-02 | 8.21E-11 |
| cg27652459 | ARHGAP1 | Body | NA | 6.61E-03 | 7.60E-07 |
| ch.15.677975: | NA | NA | NA | -1.38E-02 | 1.27E-05 |

Supplementary Table S2. Canonical pathways over-represented by genes linked to hyper-methylated CpGs

| Gene Set Name [# Genes (K)] | Description | # Genes in Overlap (k) | p-value | FDR q-value |
|---|---|---|---|---|
| REACTOME_GPCR_LIGAND_BINDING [408] | Genes involved in GPCR ligand binding | 29 | $9.69 e^{-14}$ | $1.29 e^{-10}$ |
| REACTOME_NEURONAL_SYSTEM [279] | Genes involved in Neuronal System | 23 | $1.73 e^{-12}$ | $1.15 e^{-9}$ |
| KEGG_NEUROACTIVE_LIGAND_RECEPTOR_INTER TERACTION [272] | Neuroactive ligand-receptor interaction | 21 | $5.48 e^{-11}$ | $2.37 e^{-8}$ |
| REACTOME_CLASS_A1_RHODOPSIN_LIKE_RECEP CEPTORS [305] | Genes involved in Class A/1 (Rhodopsin-like receptors) | 22 | $7.14 e^{-11}$ | $2.37 e^{-8}$ |
| REACTOME_SIGNALING_BY_GPCR [920] | Genes involved in Signaling by GPCR | 34 | $3.31 e^{-8}$ | $8.79 e^{-6}$ |
| REACTOME_POTASSIUM_CHANNELS [98] | Genes involved in Potassium Channels | 11 | $4.73 e^{-8}$ | $1.05 e^{-5}$ |
| KEGG_CALCIUM_SIGNALING_PATHWAY [178] | Calcium signaling pathway | 14 | $7 e^{-8}$ | $1.33 e^{-5}$ |
| NABA_MATRISOME [1028] | Ensemble of genes encoding extracellular matrix and extracellular matrix-associated proteins | 35 | $1.5 e^{-7}$ | $2.49 e^{-5}$ |
| REACTOME_AMINE_LIGAND_BINDING_RECEPTOR TORS [38] | Genes involved in Amine ligand-binding receptors | 7 | $4.32 e^{-7}$ | $6.38 e^{-5}$ |
| PID_WNT_SIGNALING_PATHWAY [28] | Wnt signaling network | 6 | $1.15 e^{-6}$ | $1.53 e^{-4}$ |
| REACTOME_TRANSMEMBRANE_TRANSPORT_OF_S M _SMALL_MOLECULES [413] | Genes involved in Transmembrane transport of small molecules | 19 | $1.64 e^{-6}$ | $1.98 e^{-4}$ |
| REACTOME_TRANSMISSION_ACROSS_CHEMICAL_ AL_SYNAPSES [186] | Genes involved in Transmission across Chemical Synapses | 12 | $4.81 e^{-6}$ | $5.09 e^{-4}$ |
| REACTOME_GPCR_DOWNSTREAM_SIGNALING [805] | Genes involved in GPCR downstream signaling | 27 | $5.22 e^{-6}$ | $5.09 e^{-4}$ |
| REACTOME_PEPTIDE_LIGAND_BINDING_RECEPT EPTORS [188] | Genes involved in Peptide ligand-binding receptors | 12 | $5.37 e^{-6}$ | $5.09 e^{-4}$ |
| REACTOME_G_ALPHA_I_SIGNALLING_EVENTS | Genes involved in G | 12 | 7.79 | 6.9 e- |

| | | | | |
|---|---|---|---|---|
| [195] | alpha (i) signalling events | | e$^{-6}$ | 4 |
| REACTOME_SLC_MEDIATED_TRANSMEMBRANE_TR _TRANSPORT [241] | Genes involved in SLC-mediated transmembrane transport | 13 | 1.36 e$^{-5}$ | 1.1 e$^{-3}$ |
| REACTOME_DEVELOPMENTAL_BIOLOGY [396] | Genes involved in Developmental Biology | 17 | 1.41 e$^{-5}$ | 1.1 e$^{-3}$ |
| REACTOME_REGULATION_OF_INSULIN_SECRETI ETION [93] | Genes involved in Regulation of Insulin Secretion | 8 | 2.38 e$^{-5}$ | 1.76 e$^{-3}$ |
| REACTOME_TRANSPORT_OF_INORGANIC_CATION IONS_ANIONS_AND_AMINO_ACIDS_OLIGOPEPTI PTIDES [94] | Genes involved in Transport of inorganic cations/anions and amino acids/oligopeptides | 8 | 2.57 e$^{-5}$ | 1.8 e$^{-3}$ |
| REACTOME_GENERIC_TRANSCRIPTION_PATHWAY [352] | Genes involved in Generic Transcription Pathway | 15 | 4.83 e$^{-5}$ | 3.21 e$^{-3}$ |
| REACTOME_NA_CL_DEPENDENT_NEUROTRANSMIT MITTER_TRANSPORTERS [17] | Genes involved in Na+/Cl- dependent neurotransmitter transporters | 4 | 5.17 e$^{-5}$ | 3.27 e$^{-3}$ |
| KEGG_HEDGEHOG_SIGNALING_PATHWAY [56] | Hedgehog signaling pathway | 6 | 7.35 e$^{-5}$ | 4.44 e$^{-3}$ |
| REACTOME_GLYCOSAMINOGLYCAN_METABOLISM [111] | Genes involved in Glycosaminoglycan metabolism | 8 | 8.47 e$^{-5}$ | 4.9 e$^{-3}$ |
| NABA_MATRISOME_ASSOCIATED [753] | Ensemble of genes encoding ECM-associated proteins including ECM-affilaited proteins, ECM regulators and secreted factors | 23 | 1.05 e$^{-4}$ | 5.82 e$^{-3}$ |
| KEGG_TGF_BETA_SIGNALING_PATHWAY [86] | TGF-beta signaling pathway | 7 | 1.09 e$^{-4}$ | 5.82 e$^{-3}$ |
| REACTOME_CLASS_B_2_SECRETIN_FAMILY_REC RECEPTORS [88] | Genes involved in Class B/2 (Secretin family receptors) | 7 | 1.26 e$^{-4}$ | 6.47 e$^{-3}$ |
| WNT_SIGNALING [89] | Genes related to Wnt-mediated signal transduction | 7 | 1.36 e$^{-4}$ | 6.69 e$^{-3}$ |
| REACTOME_INTEGRATION_OF_ENERGY_METABOL BOLISM [120] | Genes involved in Integration of energy metabolism | 8 | 1.46 e$^{-4}$ | 6.94 e$^{-3}$ |
| REACTOME_VOLTAGE_GATED_POTASSIUM_CHANN ANNELS [43] | Genes involved in Voltage gated | 5 | 2.01 e$^{-4}$ | 9.2 e$^{-3}$ |

| | | | | |
|---|---|---|---|---|
| | Potassium channels | | | |
| NABA_CORE_MATRISOME [275] | Ensemble of genes encoding core extracellular matrix including ECM glycoproteins, collagens and proteoglycans | 12 | 2.16 $e^{-4}$ | 9.59 $e^{-3}$ |
| KEGG_FOCAL_ADHESION [201] | Focal adhesion | 10 | 2.55 $e^{-4}$ | 1.09 $e^{-2}$ |
| KEGG_MELANOGENESIS [102] | Melanogenesis | 7 | 3.16 $e^{-4}$ | 1.31 $e^{-2}$ |
| PID_HES_HEY_PATHWAY [48] | Notch-mediated HES/HEY network | 5 | 3.39 $e^{-4}$ | 1.35 $e^{-2}$ |
| REACTOME_AMINE_COMPOUND_SLC_TRANSPORTE RTERS [27] | Genes involved in Amine compound SLC transporters | 4 | 3.45 $e^{-4}$ | 1.35 $e^{-2}$ |
| REACTOME_NUCLEAR_RECEPTOR_TRANSCRIPTIO TION_PATHWAY [49] | Genes involved in Nuclear Receptor transcription pathway | 5 | 3.74 $e^{-4}$ | 1.42 $e^{-2}$ |
| REACTOME_REGULATION_OF_BETA_CELL_DEVEL VELOPMENT [30] | Genes involved in Regulation of beta-cell development | 4 | 5.23 $e^{-4}$ | 1.89 $e^{-2}$ |
| PID_BETA_CATENIN_NUC_PATHWAY [80] | Regulation of nuclear beta catenin signaling and target gene transcription | 6 | 5.27 $e^{-4}$ | 1.89 $e^{-2}$ |
| KEGG_BASAL_CELL_CARCINOMA [55] | Basal cell carcinoma | 5 | 6.41 $e^{-4}$ | 2.24 $e^{-2}$ |
| KEGG_ECM_RECEPTOR_INTERACTION [84] | ECM-receptor interaction | 6 | 6.84 $e^{-4}$ | 2.29 $e^{-2}$ |
| KEGG_WNT_SIGNALING_PATHWAY [151] | Wnt signaling pathway | 8 | 6.89 $e^{-4}$ | 2.29 $e^{-2}$ |
| REACTOME_NEUROTRANSMITTER_RELEASE_CYCL YCLE [34] | Genes involved in Neurotransmitter Release Cycle | 4 | 8.51 $e^{-4}$ | 2.76 $e^{-2}$ |
| BIOCARTA_ALK_PATHWAY [37] | ALK in cardiac myocytes | 4 | 1.18 $e^{-3}$ | 3.65 $e^{-2}$ |
| REACTOME_GABA_SYNTHESIS_RELEASE_REUPTA PTAKE_AND_DEGRADATION [17] | Genes involved in GABA synthesis, release, reuptake and degradation | 3 | 1.18 $e^{-3}$ | 3.65 $e^{-2}$ |

Supplementary Table S3. Canonical pathways over-represented by genes linked to hypo-methylated CpGs

| **Gene Set Name [# Genes (K)]** | **Description** | **# Genes in Overlap (k)** | **p-value** | **FDR q-value** |
|---|---|---|---|---|
| NABA_MATRISOME [1028] | Ensemble of genes encoding extracellular matrix and extracellular matrix-associated proteins | 89 | 7.39 $e^{-27}$ | 9.82 $e^{-24}$ |
| NABA_MATRISOME_ASSOCIATED [753] | Ensemble of genes encoding ECM-associated proteins including ECM-affilaited proteins, ECM regulators and secreted factors | 59 | 3.63 $e^{-16}$ | 2.42 $e^{-13}$ |
| NABA_CORE_MATRISOME [275] | Ensemble of genes encoding core extracellular matrix including ECM glycoproteins, collagens and proteoglycans | 30 | 2.14 $e^{-12}$ | 9.49 $e^{-10}$ |
| REACTOME_TRANSMEMBRANE_TRANSPORT_OF_SM _SMALL_MOLECULES [413] | Genes involved in Transmembrane transport of small molecules | 36 | 1.09 $e^{-11}$ | 3.61 $e^{-9}$ |
| KEGG_FOCAL_ADHESION [201] | Focal adhesion | 23 | 3.02 $e^{-10}$ | 8.03 $e^{-8}$ |
| NABA_ECM_GLYCOPROTEINS [196] | Genes encoding structural ECM glycoproteins | 22 | 1.06 $e^{-9}$ | 2.13 $e^{-7}$ |
| REACTOME_HEMOSTASIS [466] | Genes involved in Hemostasis | 35 | 1.18 $e^{-9}$ | 2.13 $e^{-7}$ |
| REACTOME_PHOSPHOLIPID_METABOLISM [198] | Genes involved in Phospholipid metabolism | 22 | 1.28 $e^{-9}$ | 2.13 $e^{-7}$ |
| REACTOME_METABOLISM_OF_LIPIDS_AND_LIPO IPOPROTEINS [478] | Genes involved in Metabolism of lipids and lipoproteins | 35 | 2.28 $e^{-9}$ | 3.37 $e^{-7}$ |
| KEGG_INSULIN_SIGNALING_PATHWAY [137] | Insulin signaling pathway | 18 | 2.77 $e^{-9}$ | 3.41 $e^{-7}$ |
| KEGG_DILATED_CARDIOMYOPATHY [92] | Dilated cardiomyopathy | 15 | 2.82 $e^{-9}$ | 3.41 $e^{-7}$ |
| REACTOME_SIGNALING_BY_PDGF [122] | Genes involved in Signaling by PDGF | 16 | 2.14 $e^{-8}$ | 2.37 $e^{-6}$ |
| KEGG_VIBRIO_CHOLERAE_INFECTION [56] | Vibrio cholerae infection | 11 | 4.94 $e^{-8}$ | 5.05 $e^{-6}$ |
| KEGG_HYPERTROPHIC_CARDIOMYOPATHY_HCM [85] | Hypertrophic cardiomyopathy (HCM) | 13 | 7.02 $e^{-8}$ | 6.67 $e^{-6}$ |
| REACTOME_NEUROTRANSMITTER_RECEPTOR_BIN BINDING_AND_DOWNSTREAM_TRANSMISSION_IN | Genes involved in Neurotransmitter Receptor Binding And Downstream Transmission In The | 16 | 1.12 $e^{-7}$ | 9.91 $e^{-6}$ |

| | | | | |
|---|---|---|---|---|
| _IN_THE_POSTSYNAPTIC_CELL [137] | Postsynaptic Cell | | | |
| NABA_SECRETED_FACTORS [344] | Genes encoding secreted soluble factors | 26 | 1.38 e$^{-7}$ | 1.15 e$^{-5}$ |
| KEGG_ALLOGRAFT_REJECTION [38] | Allograft rejection | 9 | 1.49 e$^{-7}$ | 1.16 e$^{-5}$ |
| REACTOME_DOWNSTREAM_SIGNAL_TRANSDUCTIO TION [95] | Genes involved in Downstream signal transduction | 13 | 2.69 e$^{-7}$ | 1.91 e$^{-5}$ |
| REACTOME_CA_DEPENDENT_EVENTS [30] | Genes involved in Ca-dependent events | 8 | 2.73 e$^{-7}$ | 1.91 e$^{-5}$ |
| REACTOME_CYTOKINE_SIGNALING_IN_IMMUNE_ NE_SYSTEM [270] | Genes involved in Cytokine Signaling in Immune system | 22 | 3.49 e$^{-7}$ | 2.32 e$^{-5}$ |
| REACTOME_PLATELET_ACTIVATION_SIGNALING ING_AND_AGGREGATION [208] | Genes involved in Platelet activation, signaling and aggregation | 19 | 3.83 e$^{-7}$ | 2.42 e$^{-5}$ |
| REACTOME_DAG_AND_IP3_SIGNALING [32] | Genes involved in DAG and IP3 signaling | 8 | 4.71 e$^{-7}$ | 2.77 e$^{-5}$ |
| KEGG_ECM_RECEPTOR_INTERACTION [84] | ECM-receptor interaction | 12 | 4.79 e$^{-7}$ | 2.77 e$^{-5}$ |
| REACTOME_IMMUNE_SYSTEM [933] | Genes involved in Immune System | 47 | 5.52 e$^{-7}$ | 2.92 e$^{-5}$ |
| KEGG_TYPE_I_DIABETES_MELLITUS [44] | Type I diabetes mellitus | 9 | 5.71 e$^{-7}$ | 2.92 e$^{-5}$ |
| REACTOME_G_ALPHA_Z_SIGNALLING_EVENTS [44] | Genes involved in G alpha (z) signalling events | 9 | 5.71 e$^{-7}$ | 2.92 e$^{-5}$ |
| REACTOME_NGF_SIGNALLING_VIA_TRKA_FROM_ OM_THE_PLASMA_MEMBRANE [137] | Genes involved in NGF signalling via TRKA from the plasma membrane | 15 | 6.43 e$^{-7}$ | 3.17 e$^{-5}$ |
| REACTOME_SIGNALLING_BY_NGF [217] | Genes involved in Signalling by NGF | 19 | 7.32 e$^{-7}$ | 3.47 e$^{-5}$ |
| KEGG_VIRAL_MYOCARDITIS [73] | Viral myocarditis | 11 | 8.29 e$^{-7}$ | 3.8 e$^{-5}$ |
| REACTOME_SIGNALING_BY_GPCR [920] | Genes involved in Signaling by GPCR | 46 | 8.92 e$^{-7}$ | 3.95 e$^{-5}$ |
| KEGG_TYPE_II_DIABETES_MELLITUS [47] | Type II diabetes mellitus | 9 | 1.03 e$^{-6}$ | 4.43 e$^{-5}$ |
| KEGG_ENDOCYTOSIS [183] | Endocytosis | 17 | 1.22 e$^{-6}$ | 4.9 e$^{-5}$ |
| REACTOME_PI_METABOLISM [48] | Genes involved in PI Metabolism | 9 | 1.25 e$^{-6}$ | 4.9 e$^{-5}$ |
| KEGG_PHOSPHATIDYLINOSITOL_SIGNALING_SY _SYSTEM [76] | Phosphatidylinositol signaling system | 11 | 1.25 e$^{-6}$ | 4.9 e$^{-5}$ |
| REACTOME_TRANSMISSION_ACROSS_CHEMICAL_ AL_SYNAPSES [186] | Genes involved in Transmission across Chemical Synapses | 17 | 1.53 e$^{-6}$ | 5.83 e$^{-5}$ |

| | | | | |
|---|---|---|---|---|
| REACTOME_AXON_GUIDANCE [251] | Genes involved in Axon guidance | 20 | 1.66 $e^{-6}$ | 6.12 $e^{-5}$ |
| REACTOME_NEURONAL_SYSTEM [279] | Genes involved in Neuronal System | 21 | 2.29 $e^{-6}$ | 8.22 $e^{-5}$ |
| KEGG_PATHWAYS_IN_CANCER [328] | Pathways in cancer | 23 | 2.56 $e^{-6}$ | 8.97 $e^{-5}$ |
| KEGG_TIGHT_JUNCTION [134] | Tight junction | 14 | 2.65 $e^{-6}$ | 9.02 $e^{-5}$ |
| KEGG_GRAFT_VERSUS_HOST_DISEASE [42] | Graft-versus-host disease | 8 | 4.32 $e^{-6}$ | 1.41 $e^{-4}$ |
| REACTOME_L1CAM_INTERACTIONS [86] | Genes involved in L1CAM interactions | 11 | 4.35 $e^{-6}$ | 1.41 $e^{-4}$ |
| PID_INTEGRIN3_PATHWAY [43] | Beta3 integrin cell surface interactions | 8 | 5.2 $e^{-6}$ | 1.59 $e^{-4}$ |
| REACTOME_PLC_BETA_MEDIATED_EVENTS [43] | Genes involved in PLC beta mediated events | 8 | 5.2 $e^{-6}$ | 1.59 $e^{-4}$ |
| REACTOME_GPCR_DOWNSTREAM_SIGNALING [805] | Genes involved in GPCR downstream signaling | 40 | 5.25 $e^{-6}$ | 1.59 $e^{-4}$ |
| REACTOME_REGULATION_OF_WATER_BALANCE_BE_BY_RENAL_AQUAPORINS [44] | Genes involved in Regulation of Water Balance by Renal Aquaporins | 8 | 6.22 $e^{-6}$ | 1.84 $e^{-4}$ |
| KEGG_CHEMOKINE_SIGNALING_PATHWAY [190] | Chemokine signaling pathway | 16 | 8.84 $e^{-6}$ | 2.55 $e^{-4}$ |
| REACTOME_GLUCAGON_SIGNALING_IN_METABOLIC_REGULATION [34] | Genes involved in Glucagon signaling in metabolic regulation | 7 | 1.01 $e^{-5}$ | 2.84 $e^{-4}$ |
| NABA_ECM_AFFILIATED [171] | Genes encoding proteins affiliated structurally or functionally to extracellular matrix proteins | 15 | 1.03 $e^{-5}$ | 2.84 $e^{-4}$ |
| NABA_ECM_REGULATORS [238] | Genes encoding enzymes and their regulators involved in the remodeling of the extracellular matrix | 18 | 1.11 $e^{-5}$ | 3 $e^{-4}$ |
| REACTOME_OPIOID_SIGNALLING [78] | Genes involved in Opioid Signalling | 10 | 1.16 $e^{-5}$ | 3.07 $e^{-4}$ |

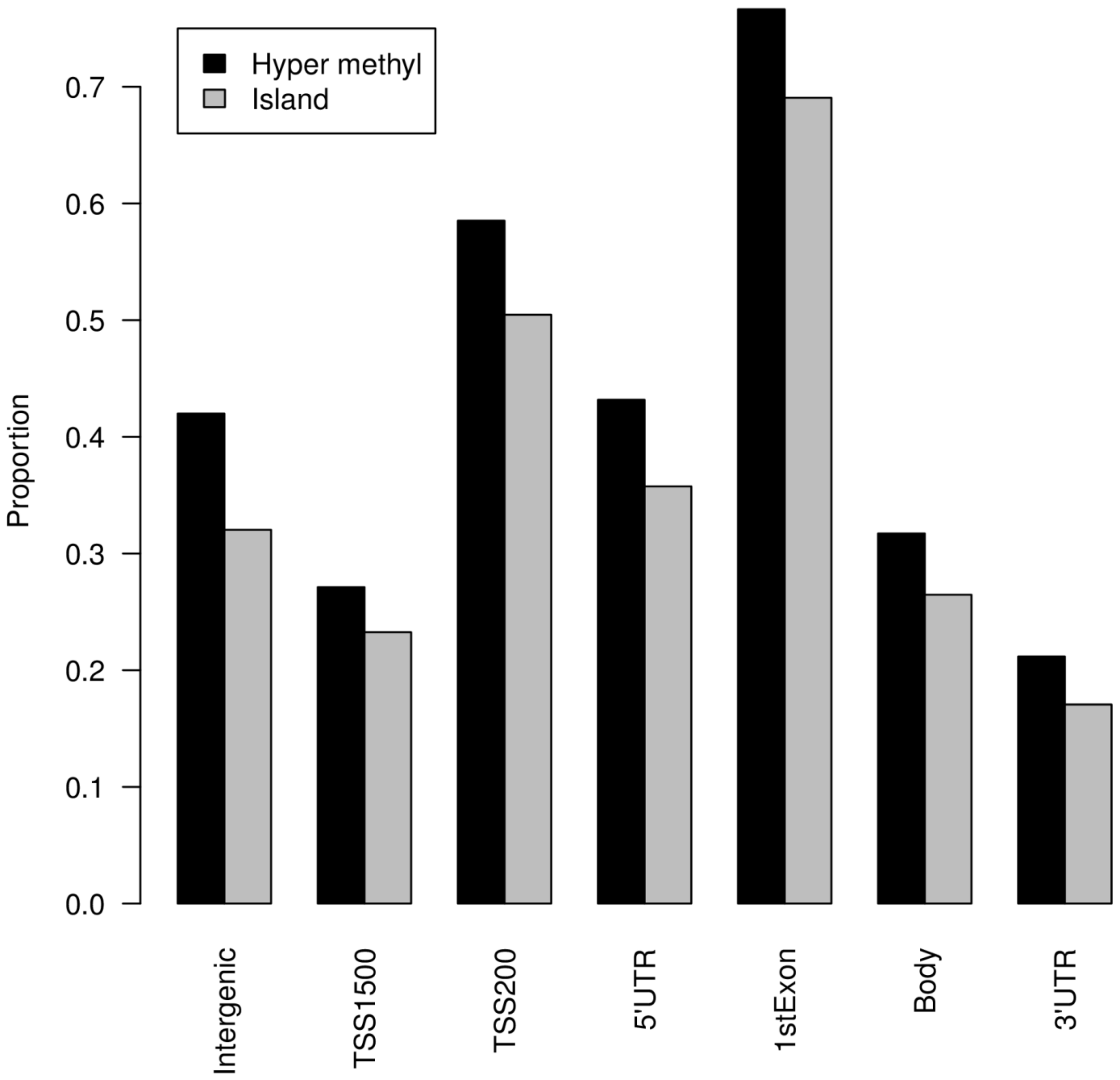


Supplementary_Figure_S1. The proportion of age-methylated CpGs by gene region (black bars) and the proportion of CpGs residing at CGI in the region (grey bar).
